# The relationships between the isoelectric point and: length of proteins, taxonomy and ecology of organisms

**DOI:** 10.1186/1471-2164-8-163

**Published:** 2007-06-12

**Authors:** Joanna Kiraga, Pawel Mackiewicz, Dorota Mackiewicz, Maria Kowalczuk, Przemysław Biecek, Natalia Polak, Kamila Smolarczyk, Miroslaw R Dudek, Stanislaw Cebrat

**Affiliations:** 1 Department of Genomics, University of Wrocław, ul. Przybyszewskiego 63/77, 51-148 Wrocław, Poland; 2 Institute of Physics, University of Zielona Góra, ul. Szafrana 4A, 65-516 Zielona Góra, Poland

## Abstract

**Background:**

The distribution of isoelectric point (pI) of proteins in a proteome is universal for all organisms. It is bimodal dividing the proteome into two sets of acidic and basic proteins. Different species however have different abundance of acidic and basic proteins that may be correlated with taxonomy, subcellular localization, ecological niche of organisms and proteome size.

**Results:**

We have analysed 1784 proteomes encoded by chromosomes of Archaea, Bacteria, Eukaryota, and also mitochondria, plastids, prokaryotic plasmids, phages and viruses. We have found significant correlation in more than 95% of proteomes between the protein length and pI in proteomes – positive for acidic proteins and negative for the basic ones. Plastids, viruses and plasmids encode more basic proteomes while chromosomes of Archaea, Bacteria, Eukaryota, mitochondria and phages more acidic ones. Mitochondrial proteomes of Viridiplantae, Protista and Fungi are more basic than Metazoa. It results from the presence of basic proteins in the former proteomes and their absence from the latter ones and is related with reduction of metazoan genomes. Significant correlation was found between the pI bias of proteomes encoded by prokaryotic chromosomes and proteomes encoded by plasmids but there is no correlation between eukaryotic nuclear-coded proteomes and proteomes encoded by organelles. Detailed analyses of prokaryotic proteomes showed significant relationships between pI distribution and habitat, relation to the host cell and salinity of the environment, but no significant correlation with oxygen and temperature requirements. The salinity is positively correlated with acidicity of proteomes. Host-associated organisms and especially intracellular species have more basic proteomes than free-living ones. The higher rate of mutations accumulation in the intracellular parasites and endosymbionts is responsible for the basicity of their tiny proteomes that explains the observed positive correlation between the decrease of genome size and the increase of basicity of proteomes. The results indicate that even conserved proteins subjected to strong selectional constraints follow the global trend in the pI distribution.

**Conclusion:**

The distribution of pI of proteins in proteomes shows clear relationships with length of proteins, subcellular localization, taxonomy and ecology of organisms. The distribution is also strongly affected by mutational pressure especially in intracellular organisms.

## Background

The abundance of genomic data allows for answering questions concerning the whole genomes' or proteomes' structure and evolution. One of the most intriguing recent findings is the distribution of isoelectric point (pI) of proteins in the whole proteomes. So called virtual 2D gels (i.e. plots where pI of proteins is plotted against their molecular weight) seem to be universal – in all proteomes they are usually bimodal with very low fractions of proteins with pI close to 7.4 [1-8]. More detailed analyses have shown that the distribution is connected with specific properties of basic and acidic residues of amino acids and combinations of their pK values. The probability of constructing a protein with pI close to 7.4 of naturally occurring amino acids is low. This is in agreement with expectations because proteins are most insoluble, least reactive and unstable in pH close to their pI, and pH of the majority of the cell interior compartments is close to 7.5 [5,9]. Thus, this property of proteomes could be the result of selection at the very early steps of evolution.

Different relationships between pI and other phenomena were discovered. Schwartz et al. [6] observed the correlation between the trimodal distribution of pI and the subcellular localization of proteins. Knight et al. [7] characterized more than 100 prokaryotic and eukaryotic proteomes by virtual 2D-gels and observed very little or no relations to phylogeny while significant relationship existed with the ecological niche of organisms. Moreover, they found a negative correlation between proteome size and basicity for the smallest and the most basic proteomes. Also very well documented has been the shift of pI distribution toward acidicity in halophilic bacteria which is probably related to their adaptation to high salt environments [10-12]. Similarly, it has been assumed that the basic proteomes of *Coxiella burnetti *and *Helicobacter pylori *are connected with an adaptation to acidic environment [13] and the basic proteomes of some Archaea are an adaptation to high temperature [14]. On the other hand, Nandi et al. [15] have focused on an evolutionary approach and found that the molecular weight of proteins is a much more conserved feature than their pI value. They concluded that a lot of orthologous proteins change their pI between acidic and basic and only a few stay exclusively acidic or basic in different organisms. Furthermore, Schwartz et al. [6] and Knight et al. [7] found that membrane proteins are larger and more basic than non-membrane ones whereas Nandi et al. [15] found that many orthologous membrane proteins have very variable pI values and may be used as markers to predict the organism's ecological niche.

In this paper we have broadened our analysis to proteomes of organelles, viruses and bacteriophages and applied different methods and parameters to describe quantitatively the pI distribution of proteomes. One of the aims of these studies is uncovering the relationship between the pI and the protein length. In the previous analyses, such correlations even if existing, were neglected. Furthermore, we have analysed the relations between the pI distributions of proteomes and the taxonomy and ecology of corresponding organisms considering different taxonomical levels and ecological signatures such as habitat, relation to the host cell, salinity, oxygen and temperature requirements. Moreover, we have tried to explain the observed relationship between the proteome size and the pI distribution of proteomes [7].

## Results and Discussion

### General properties of isoelectric point distributions in proteomes

**Figure 1:**
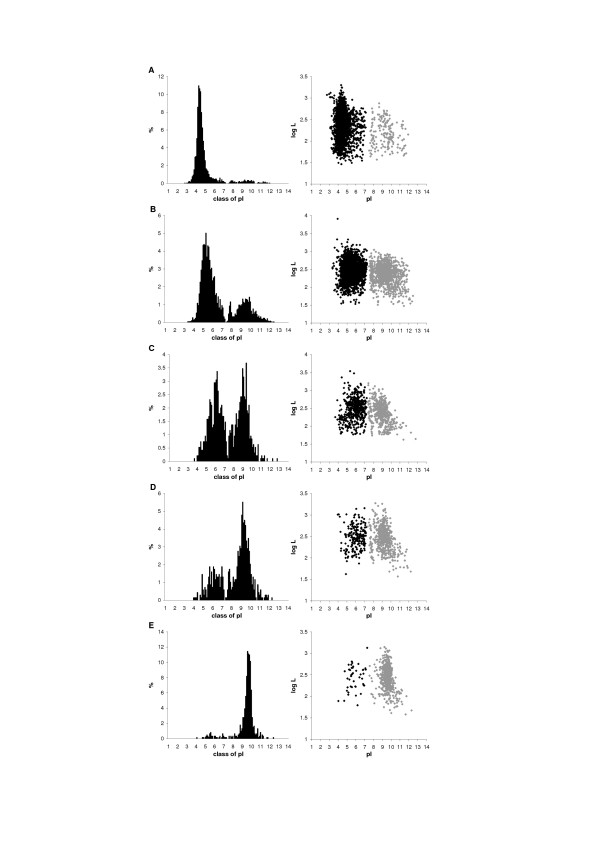
Histograms of pI values at 0.1 unit intervals (left panel) and relationship between length of proteins (log L) and their pI (right panel) for selected prokaryotic proteomes with the different pI bias (b): (A) *Natronomonas pharaonis *DSM 2160, b = -87%. (B) *Silicibacter pomeroyi *DSS-3, b = -44%; (C) *Ehrlichia ruminantium *str. Gardel, b = 0%; (D) *Mycoplasma pneumoniae *M129, b = 43%; (E) *Wigglesworthia glossinidia*, b = 86%. Black points represent the set of acidic proteins while grey ones – the set of basic proteins. Diagrams for all analysed proteomes are available in additional data files 2, 3 and 4.

Some examples of the distributions of isoelectric points of proteins coded by selected proteomes are shown in Fig. 1 (for more proteomes see additional data files 2, 3 and 4). All analysed proteomes show usually bimodal distributions with a smaller third peak between two main peaks, which are in agreement with the results of other authors [7,8] and suggestions that the multimodal distribution of pI corresponds to the pK values of amino acid moieties [8,14].

To simplify comparative analyses of the pI distribution of different proteomes, we have divided the whole sets of proteins into two sets called later the acidic and basic sets. The division point corresponds to the pI value for which the pI distribution reaches the minimum between acidic and basic sets (see the Methods section for details). For most of proteomes the division point was between 7.4 and 7.5. The distribution of the values of division points is very narrow – the range is 7.22 – 7.54 with the average and median 7.41. We have not found any correlation between the pH of these points and the taxonomical or ecological classification of organisms and genome sizes. The universality of the pH value of the minimum in pI distributions supports the conclusion of other authors that the bimodal distribution of pI results from intrinsic chemical properties of amino acids [8].

For each proteome we have calculated the average pI separately for the basic and the acidic sets of proteins, the average length of proteins and the pI bias (see the Methods section). The pI bias simply describes the asymmetry of the bimodal distribution of pI (Fig. 1). It ranges from -100% to 100%. These two extreme values indicate that all proteins in a given proteome are acidic or basic, respectively. Value close to zero means that a given proteome has a similar fraction of acidic and basic proteins. We have found that if a proteome has more acidic proteins, the average pI of proteins of the acidic set is lower and the relationship is statistically significant (correlation coefficient, r = 0.76, p < 0.001) – additional data file 5A. On the other hand, if the basic proteins prevail in the proteome it is not connected with the shift of average pI value of basic proteins with exception of some intracellular microorganisms (additional data file 5B). We have also found that the basic sets have greater variance of their pI than the acidic sets that indicates greater diversity of the basic proteins.

### Relationships between pI value and size of proteins

**Figure 2:**
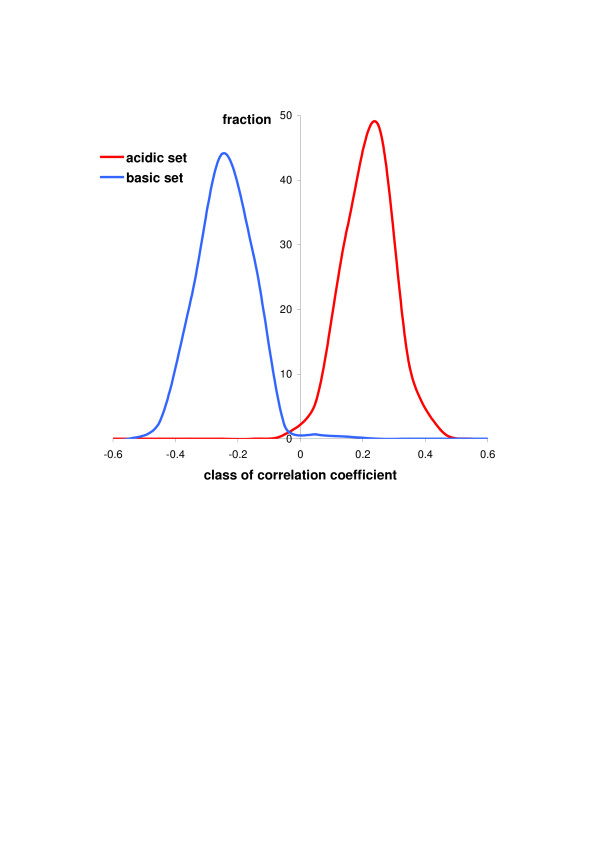
Distributions of the correlation coefficients between pI value and length of proteins calculated separately for acidic and basic sets of proteomes.

The 2D virtual distribution of proteins shows a characteristic pattern that may resemble a "butterfly" [7] or "lungs", and no correlation has been observed between pI and the molecular weight of proteins. Nandi et al. [15] have found that molecular weight of proteins is a much more conserved feature than their pI which would additionally suggest that there should be no correlation between pI and molecular weight. However, after dividing proteomes into acidic and basic sets, the correlation between the pI and the size of proteins for many proteomes can be seen – positive for acidic proteins and negative for basic proteins (Fig. 1). Although the correlation coefficient is not very high, for more than 92% of acidic sets and almost 99% of basic sets of chromosome-encoded proteomes of Archaea, Bacteria and Eukaryota it is statistically significant with Benjamini-Hochberg adjusted p < 0.05. Furthermore, the correlation between the size and pI of proteins is a general feature with very narrow distributions of the correlation coefficients (shown in Fig. 2 separately for acidic and basic proteins).

To show that these correlations are intrinsic properties of proteomes, we have created three artificial proteomes consisting of randomly generated proteins characterized by: (i) the average amino acid composition of *E. coli *proteins and the length of the real *E. coli *proteins; (ii) equal frequencies of amino acids and the length of the real *E. coli *proteins; (iii) the average amino acid composition of the *E. coli *proteins and the uniform length distribution in the range of the *E. coli *proteins. (See additional data file 6 for distributions of pI and relationships between length of proteins and their pI for these proteomes.) The generation of artificial proteomes was repeated 100 times for each set. In each case we got significant correlations between length and pI values (Tab. 1). Each version of artificial proteomes produced the bimodal distribution of pI and stronger correlations between the pI and the length of proteins than in the real *E. coli *proteome. The pI bias of the real proteome does not fall within the range of pI values of generated proteomes and is nearest to the generated proteomes assuming the average amino acid composition and the length of the real *E. coli *proteins. The pI bias was more balanced when proteins were generated from the uniform amino acids distribution, however still without proteins with pI close to 7.4. In addition, the proteome became more acidic when the length of proteins corresponded to the uniform distribution. It suggests that selection for the length of proteins may shape the pI distribution to some extent.

**Table 1:** Properties of artificial and real *E. coli *proteomes.

Analysed proteome	Correlation coefficient for		pI bias [%]
	acidic set	basic set	
the average amino acid composition of *E. coli *proteins; the length of *E. coli *proteins	0.23	-0.28	-32 (-29 to -36)
equal frequencies of amino acids; the length of *E. coli *proteins	0.32	-0.28	-5 (-8 to -2)
the average amino acid composition of *E. coli *proteins; the uniform length distribution in the range of *E. coli *proteins	0.33	-0.48	-50 (-53 to -46)
the real proteome of *E. coli *K12	0.15	-0.25	-27

Each of the first three proteomes was generated 100 times and the resulting correlation coefficients and the pI bias were averaged over 100 values. Correlation coefficients both for all generated artificial proteomes and the real proteome are statistically significant with p < 0.001.

The results mean that the longer proteins can maintain the neutral or nearly neutral pI whereas the most extreme pI values are specific for shorter proteins. It probably results from purely statistical reasons. The shorter proteins show higher fluctuation of amino acid composition, which strongly influences their pI – incorporation of even one charged residue shifts significantly the protein pI to the lower or higher value. On the other hand, long proteins, usually composed of more charged amino acids much better buffer the effect of fluctuation in their composition and can keep their pI even close to 7.4. This 'statistical' explanation of the relationship between proteins size and pI does not exclude the possibility that this relation may have some biological consequences in a diversification of proteins' structure and function. For example, if there exists a selection constraint to generate a very acidic or very basic protein, the easiest way to accomplish that is "to make" it short. Actually, very basic proteins interacting with nucleic acids – ribosomal proteins – are usually very short. We think that more proteins subjected to such selection should be found. These proteins may belong to specific regulatory proteins, transcriptional factors, modulators, signalling proteins, small proteins interacting with other proteins etc.

We have also noticed a difference in the length of acidic and basic proteins. The comparative analyses have shown that acidic proteins are significantly longer than basic ones (t-Student test, Benjamini-Hochberg adjusted p < 0.05) in more than 95% of Archaea, Bacteria and Eukaryota proteomes. The acidic proteins are on average 73 and 107 amino acid residues longer than the basic ones in the case of Prokaryota and Eukaryota, respectively. Probably it is connected with the presence of very short ribosomal proteins in the basic set and long aminoacyl-tRNA synthetases in the acidic one [15].

Nandi et al. [15] observed that the size of orthologous proteins found in closely related organisms is much more conserved than their pI. We compared the length of orthologous proteins of many proteomes whose pI values were changed from acidic to basic or vice versa but we did not observe any statistically significant differences in the length of these proteins, either. We found that the change of pI did not depend on the length of the compared orthologs.

### Relationships between pI values of proteomes and taxonomy

**Figure 3:**
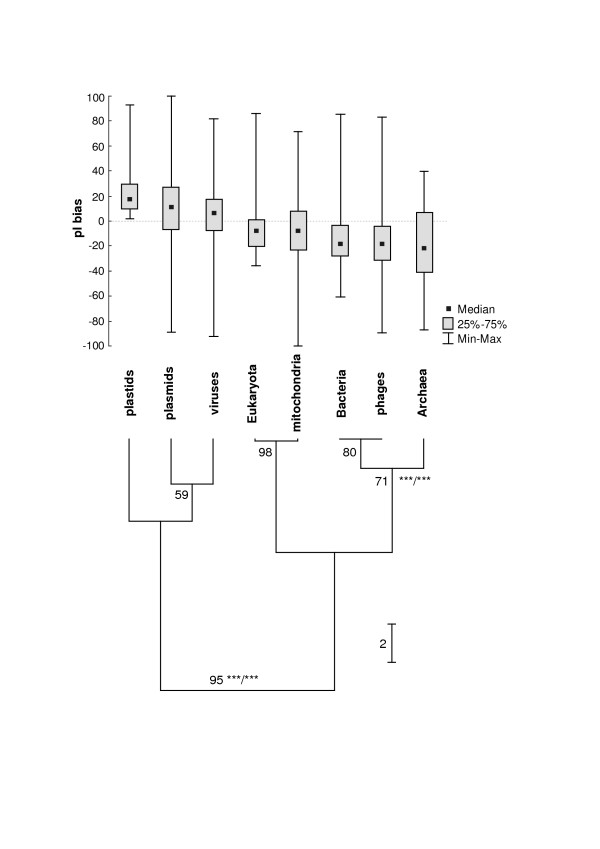
Statistical analysis of the pI bias of different groups of proteomes and their UPGMA-based clustering according to the median of the pI bias. Numbers at nodes mean the percentage support based on subsampling method and asterisks denote results of WLS-LRT/F tests (both with p < 0.001).

In the analysis of potential relationships between pI of proteomes and taxonomy we have used the pI bias that is quite concise but a very informative parameter emphasising the most distinct and basic differences in the pI distributions. Fig. 3 shows the result of statistical analysis of the pI bias of different groups of proteomes and the UPGMA dendrogram classifying the different groups of proteomes according to the median of the pI bias. The analysis allows for dividing proteomes into at least two groups: (1) the more basic proteomes coded by plastids, viruses and plasmids and (2) the more acidic proteomes of Archaea, Bacteria, Eukaryota, mitochondria and phages. This division is supported by 95% (subsampling method), WLS-LRT and F-test (both p < 0.001). Moreover, every comparison between members classified to these different groups made by Kruskal-Wallis test is statistically significant (p < 0.002). Within the acidic group it is possible to recognize some subgroups according to the high percentage support: Eukaryota with mitochondria and Bacteria with phages. The clade containing Prokaryota and phages is rather weakly supported by subsampling analysis but it is very significant according to WLS-LRT and F-test (for both p < 0.001). The observed basicity of plastid, viral and plasmid proteomes is probably connected with the prevalence of basic proteins (e.g. ribosomal in the case of plastids and basic proteins interacting with nucleic acids in the case of viruses) and it may also result from higher rate of accumulation of mutations, especially in viruses and plasmids (see the section 'Relation between pI value of proteomes, their sizes and GC content of genomes' for similar a explanation for intracellular bacteria).

Our analyses of prokaryotic proteomes performed on the lower taxonomical level did not show any relation between the pI bias and phylogeny, which is in agreement with the results of Knight et al. [7]. Many monophyletic or closely related groups (e.g. taxa of Archaea or proteobacteria) were not grouped when the pI bias was used as a criterion. It indicates that the pI distribution has not been conserved during evolution of prokaryotes. We did not observe such a relationship for eukaryotic proteomes, either.

**Figure 4:**
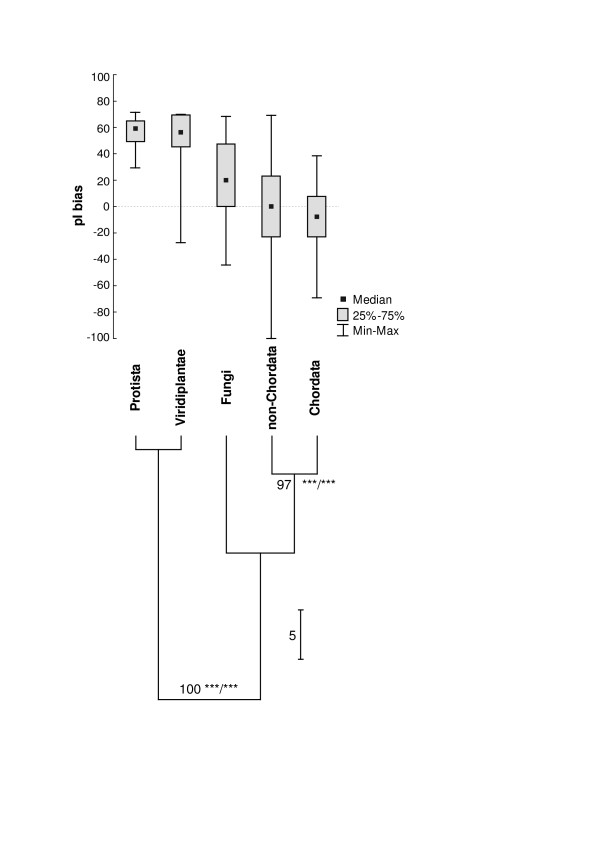
Statistical analysis of the pI bias of mitochondrial proteomes and their UPGMA-based clustering according to the median of the pI bias. Numbers at nodes mean the percentage support based on subsampling method and asterisks denote results of WLS-LRT/F tests (both with p < 0.001).

On the other hand, we have found an interesting grouping related to a phylogenetic signal when analysing mitochondrial proteomes, which are much smaller and more conserved according to their protein content than large chromosome-encoded proteomes (Fig. 4, see also Tab. 2). The analysed groups of proteomes clearly divide into two subgroups: Viridiplantae+Protista and Fungi+Metazoa. This division is strongly supported in 100%, WLS-LRT and F-test (for both p < 0.001). It is in agreement with the opinion that the clade of Fungi+Metazoa called Opisthokonta is a well-established group, which is supported by many molecular data analyses [16,17]. Furthermore, the two subgroups of Metazoa: Chordata and non-Chordata (including the most of phyla of invertebrates) form a well-supported clade (97%; WLS-LRT and F-test: for both p < 0.001). The Viridiplantae+Protista group has much more basic proteomes on average than the Fungi+Metazoa. In the Opisthokonta group Fungi have moderately basic proteomes while Metazoa possess neutral (non-Chordata) or slightly acidic (Chordata) ones.

**Table 2:** Comparison of the pI bias for subsets of organellar proteomes.

Analysed proteome	Nuclear-coded proteins targeted to organelle		Organelle-encoded proteomes		
	
	the whole sequence	the mature protein	quartile 25%	median	quartile 75%
plastid	-5.7	-53.1	9.8	17.4	29.3
mitochondrion – Protista	4.8	-33.3	49.2	59.1	65.0
mitochondrion – Viridiplantae	-9.4	-58.1	45.4	56.4	69.4
mitochondrion – Fungi	44.4	-6.9	0.0	20.0	47.4
mitochondrion – non-Chordata	21.9	-34.4	-23.1	0.0	23.1
mitochondrion – Chordata	46.0	-15.0	-23.1	-7.7	7.7

The observed phylogenetic signal in the pI bias is probably the result of different composition of proteomes. Mitochondrial proteomes of Viridiplantae, Protista and Fungi are usually several times larger than Metazoa proteomes that contain usually 12–13 proteins. Actually, the most of proteins (e.g. ribosomal proteins) that are absent from Metazoa but present in Viridiplantae, Protista and Fungi are very basic (additional data file 7). Therefore, when we performed the same analysis based on the proteomes consisting only of 12 proteins present in the most of mitochondrial proteomes the phylogenetic signal disappeared and the pI bias of basic proteomes became neutral or even acidic (for Fungi). We did not find any relationship between the pI bias and phylogeny on lower taxonomic levels: among metazoan phyla and subgroups of Craniata when analysing both full proteomes and proteomes containing only the 12 common proteins.

Similarly to Knight et al. [7] but analysing a larger set of proteomes, we have found significant correlation between the pI bias of proteomes coded by prokaryotic genomes and their plasmids (N = 63, r = 0.74, p < 0.001). There is no such correlation between the pI bias of eukaryotic nuclear-coded proteomes and proteomes of their organelles (N = 36, r = 0.04, p = 0.81). We have obtained similar results when analysing the average pI values of acidic and basic sets separately and no correlation was found in these two cases when the average length of proteins in these two sets was studied. It seems that organelle genomes are more independent than plasmids to follow the genomic trends in pI, which seems to be not true for plasmids. For example, the organelles are separated by two membranes and posses their own replicational, transcriptional and translational machinery. Plasmids code generally for more basic proteins than chromosomes but a mutational pressure or some selection constraints affect these two proteomes simultaneously and gene transfer between plasmids and chromosomes occurs probably more often than between organellar and nuclear genomes. If more data are available, it would be interesting to analyze such relationships between viral or phagal proteomes and proteomes of infected organisms.

### Relationships between pI values and subcellular localization of proteins

The analysed mitochondrial and plastid proteomes include only proteins coded by the organellar genomes. However, there are many proteins encoded by nuclear genes, which are targeted to the organelles. The nuclear-encoded organellar proteins are usually equipped with N-terminal targeting signals (transit peptides) responsible for their import into organelles. These peptides are cleaved off by organellar peptidases after the proteins are transported. Because these presequences are rich in basic amino acid residues, the pI of premature unprocessed proteins (i.e. the whole sequences) should be more basic than the pI of mature proteins (i.e. without transit peptide). In the Tab. 2 we have compared the pI bias of the premature and mature proteins with proteomes coded by organellar genomes. Because mitochondrial proteomes differ between various taxonomical groups, we analysed them separately. (See additional data file 8 for distributions of pI and relationships between length of proteins and their pI for these proteomes.) As it was expected, the presence of very basic transit peptides (pI bias 93 – 100%) in the premature proteins shifts the pI distribution of these proteins towards basicity (i.e. higher values of pI bias), whereas most mature proteins are acidic. Interestingly, only in plastid and mitochondrial proteomes of plants a weak surplus of acidic premature proteins still exists. The nuclear-encoded organellar proteomes generally differ from the organelle-encoded ones. The pI bias values only of the mitochondrial premature proteins of Viridiplantae and Fungi and mitochondrial mature proteins of Chordata fall within the quartile range of the pI bias of corresponding organelle-encoded proteomes.

Schwartz et al. [6] analyzed three subcellular localizations of proteins and found that cytoplasmic and integral membrane proteomes contain more acidic and basic proteins, respectively, whereas the nuclear proteome has rather equal proportions of the acidic and basic proteins. In this paper we have considered 12 subcellular localizations in analyses (Tab. 3). (See also additional data file 9 for distributions of pI and relationships between length of proteins and their pI for these proteomes.) The results showed that the most acidic proteomes are present not only in cytosol but also in vacuoles and lysosomes and many acidic proteins build cytoskeleton. The most basic is the mitochondrial proteome and the distribution of pI of proteins integral to membrane is slightly shifted towards basicity. The rest of subcellular localizations contain more balanced proportions of acidic and basic proteins. The basic character of integral membrane proteins may be explained by the presence of the basic residues on either side of membrane spanning region, which play a role in a stabilization of proteins in the membrane [6]. Basicity of mitochondrial proteins results from the presence of positively charged transit peptides. The other relationships between the pI and the subcellular localization are probably related to the presence of some specific groups of proteins in particular localizations (e.g. acid hydrolases in lysosomes, degradative enzymes in vacuoles and acidic proteins such as: actins, dyneins, keratins, kinesins, lamins, myosins, tubulins in cytoskeleton). It is interesting to correlate the pI distribution of the particular proteomes with the pH of the compartments in which these proteomes are located. However, we did not observe any statistically significant correlation (r = 0.38, p = 0.27, n = 10).

**Table 3:** Comparison of the pI bias for proteomes of different subcellular localization.

Proteome	number	pI bias
vacuolar	39	-69.2
cytosolic	940	-54.5
cytoskeleton	953	-51.7
lysosomal	143	-45.5
Golgi apparatus	315	-11.7
nuclear	4144	-10.5
peroxisomal	174	-2.3
extracellular	1364	4.1
endoplasmic reticulum	695	4.2
chloroplast	400	5.5
integral to membrane	2191	13.7
mitochondrial	1681	35.8

### Relationships between pI values of proteomes and ecology of their organisms

**Figure 5:**
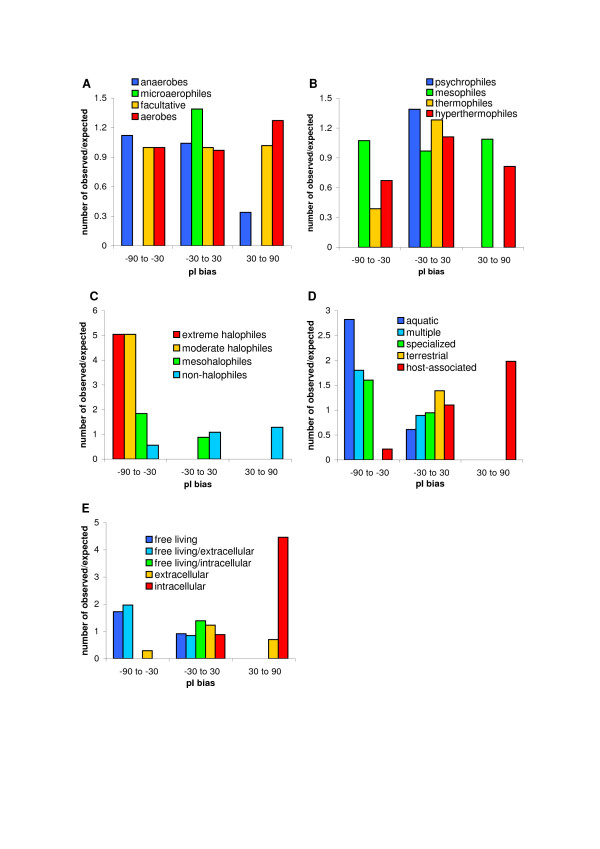
Ratios of the observed to expected number of proteomes in a given class of pI bias for different ecological classifications: (A) oxygen, (B) temperature, (C) salinity, (D) habitat and (E) relation to host cell.

To analyse the relationships between the pI of proteomes and ecology of organisms in details, all analysed prokaryotic proteomes were classified to different ecological subgroups (see the Methods section for details) and then compared in respect to the pI bias (Tab. 4). The Kruskal-Wallis tests revealed that the differences were not statistically significant when organisms were grouped according to their oxygen and temperature requirements (p > 0.1) but they were very significant when classified according to their habitat, relation to host cell and salinity requirements (p < 0.001). The same conclusion we have drawn (on the same significance level) when the χ^2 ^test was performed. Fig. 5 presents the ratios of the observed to expected number of proteomes in a given class of pI bias for different ecological classifications.

**Table 4:** Statistical analysis of the pI bias for different ecological groups.

Ecological group	number	quartile 25%	median	quartile 75%
**oxygen requirements**				
anaerobes	36	-28.4	-15.4	-3.1
microaerophiles	4	-25.5	-8.8	3.3
facultative	96	-26.8	-20.7	-13.3
aerobes	96	-29.8	-12.6	9.8

**temperature requirements**				
psychrophiles	2	-30.6	-21.9	-13.2
mesophiles	202	-29.6	-19.0	-3.3
thermophiles	13	-27.8	-20.8	-13.5
hyperthermophiles	15	-23.4	0.0	12.2

**salinity**				
extreme halophiles	3	-84.4	-79.5	-60.8
moderate halophiles	8	-49.4	-48.3	-44.8
mesohalophiles	41	-38.7	-23.4	-3.4
non-halophiles	180	-24.8	-16.3	-1.3

**habitat**				
aquatic	25	-40.4	-32.7	-3.4
multiple	56	-33.8	-29.4	-21.0
specialized	22	-48.2	-21.2	-9.9
terrestrial	12	-26.0	-22.9	-20.3
host-associated	117	-19.8	-10.6	12.8

**relation to host cell**				
free living	76	-38.4	-25.7	-13.6
free living/extracellular	41	-33.7	-26.5	-20.5
free living/intracellular	4	-12.9	-10.6	-7.8
extracellular	70	-22.1	-17.5	-4.7
intracellular	41	-8.8	14.2	42.8

The analyses showed that salinity is positively correlated with the acidity of proteomes – the more halophilic organisms have more acidic proteomes. It agrees with results of other authors who observed in halophiles predominance of acidic over basic residues [18-22] and low isoelectric point of their proteins [10-12]. Extremely halophilic and moderately halophilic bacteria are present only in the 'acidic' class and mesohalophiles disappear in the 'basic' class (Fig. 5C). This relationship is usually explained by the higher stability and solubility of proteins rich in acidic residues in hypersaline environment [19,23-28].

Considering habitat preferences, host-associated organisms have the least acidic proteomes compared to other groups and aquatic bacteria possess the most acidic ones. In the 'acidic' class aquatic bacteria are the most overrepresented group and host-associated species the most underrepresented one (Fig. 5D). On the other hand, the 'basic' class contains only host-associated microorganisms. Although proteomes of host-associated species are shifted towards more basic proteomes, they are still acidic on average (Tab. 4). However, a more detailed classification of organisms considering their relation to the host cell has revealed that proteomes of intracellular bacteria are on average basic and extracellular and free-living/intracellular species have slightly acidic proteomes (Fig. 5E). More acidic proteomes are characteristic of free-living/extracellular and free-living species. Actually, these two groups are overrepresented in the 'acidic' class whereas intracellular bacteria are strongly overrepresented in the 'basic' class. The results show that the more an organism is related to the host cell the more basic proteome it has. The explanation of this result will be discussed in the next section where the relationship between the pI bias, proteome size and GC content of genome is considered. We have also noticed that all intracellular organisms that have slightly acidic proteomes (Anaplasma, Brucella, Chlamydiae, Ehrlichia) and the majority of them (with only one exception) that have slightly basic proteomes with the pI bias ≤ 20% (Bartonella, Coxiella, Parachlamydia, *Rickettsia conorii*, Tropheryma, Wolbachia) reside and usually replicate in vacuoles or phagosomes. What is interesting, *R. conorii *has the least basic proteome within the Rickettsia genus and as the only representative of its genus was observed in a vacuole [29]. It would imply that environment of vacuoles modifies proteomes of intracellular organisms towards acidicity.

Ecological changes quite strongly and quickly influence the pI bias in the course of evolution of proteomes because the changes in the pI bias are seen even among closely related organisms. We have gathered all analysed species belonging to the same genus and having different ecological assignments (see additional data file 10). In every case the pI bias of host-associated species is more shifted towards higher values (i.e. basicity) than the bias of species living in the multiple environments. The most pronounced examples are two species of Burkholderia of which one lives in a terrestrial habitat and posses the acidic proteome and the other one is associated with a host and has the basic proteome. The proteomes of other host-associated species, although shifted towards basicity, are still acidic probably because these species are still facultative and extracellular parasites. Moreover, the clear shift of the pI bias is visible when species living in different salinity requirements are compared. The proteomes of halophilic and mesohalophilic species have more acidic proteins than non-halophilic ones.

### Relationships between pI values of proteomes, their sizes and GC content of genomes

The relationship between pI value of proteomes, their sizes and GC content of genomes was analysed by Knight et al. [7]. They concluded that there is no correlation between median pI and GC content in small basic proteomes and have opened the discussion about the reasons of basicity of tiny proteomes by giving two unverified potential explanations: selection of proteins in the proteome and selection within particular proteins. To see how general these relations are, we have performed analyses on a larger set of proteomes using the pI bias and considering the relation of organisms to host cell in more detail.

**Figure 6:**
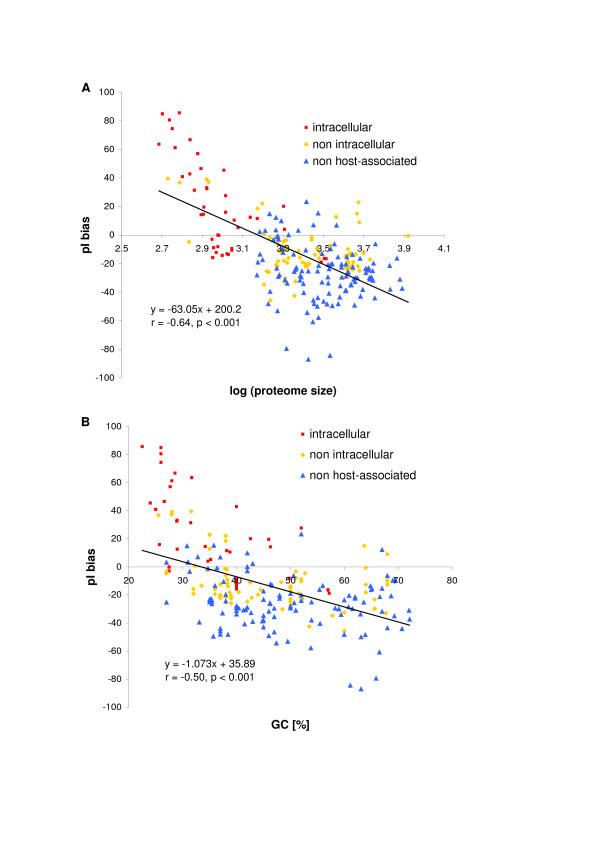
Relationship between the pI bias and: (A) logarithm of proteome size and (B) genomic GC content for different ecological groups of prokaryotes.

Fig. 6A presents the relationship between the pI bias and proteome size (expressed as logarithm of proteins number) for prokaryotic organisms. The plot in additional data file 11 includes also eukaryotic organisms. A clear trend is visible for prokaryotic proteomes – the smaller proteomes contain more basic proteins – whereas, the most of proteomes of Eukaryota are shifted towards larger values of proteome size. The correlation coefficient for the set containing prokaryotic proteomes is r = -0.64 (p < 0.001). The negative correlation has been found also for the pI bias and the GC content of genomes (for the whole set: r = -0.49, for prokaryotic proteomes r = -0.50; for both p < 0.001). Generally, the genomes with lower GC content code for more basic proteomes. Detailed analysis of prokaryotic proteomes, considering relation between organisms and the host cell have shown that the more a bacterial strain relies on its host the higher is AT fraction in its genome and the smaller and more basic proteome it possesses (Fig. 6). Most of these organisms are intracellular parasites or endosymbionts or at least they are in some way associated with their host (extracellular). It seems that the rules are general because the proteome encoded by the nucleomorph of a eukaryote *Guillardia theta *[30] is also AT-rich, reduced and the most basic in the whole set. *G. theta *is a unicellular cryptophyte alga which is a host for another cell with reduced residual nucleus called the nucleomorph. The cell may be considered endosymbiont as well.

The observed correlations are clearer when only species with proteomes smaller than 1000 proteins are considered. The correlation coefficients change from -0.64 to -0.82 for the relationship between the pI bias and proteome size and from -0.49 to -0.58 for the relationship between the pI bias and GC content and are statistically significant. Therefore, the hypothesis that the inefficiency of DNA repairing mechanisms in intracellular microorganisms causes the large AT bias of their small genomes and in consequence greater content of basic lysine [31] can not be completely excluded. It is possible that there are some other reasons of the observed relationships between the basicity of the proteome and the reduction of its size such as: gain or loss of some groups of proteins causing the pI shift of the whole proteome or selection for pI changes of particular proteins, e.g. involved in adaptation to the new environment or host [7]. The former hypothesis explains at least the differences between mitochondrial proteomes (see above).

**Figure 7:**
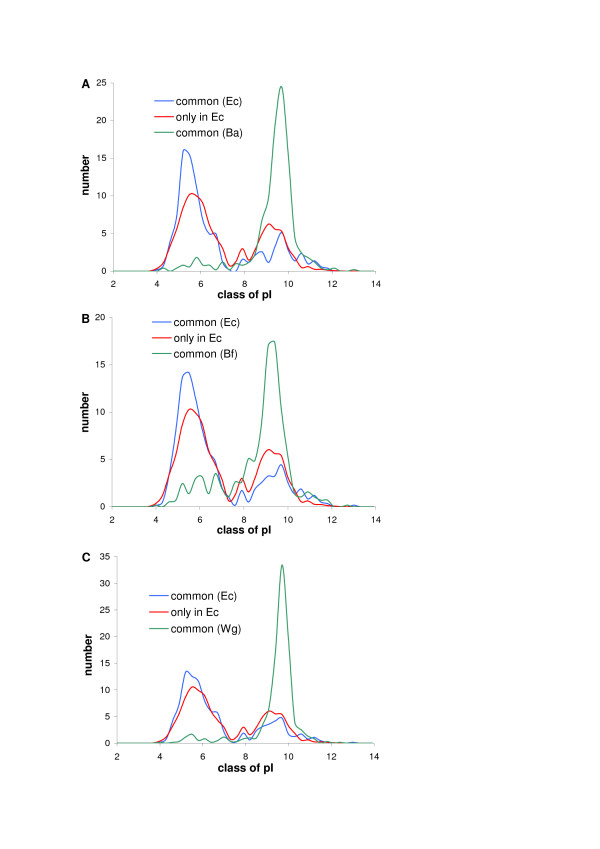
Distribution of pI for three sets of proteins: present in *E*. *coli *K12 only and not in the intracellular endosymbiont (only in Ec), *E. coli *proteins orthologous to the intracellular bacteria [(common (Ec)], and the endosymbiont proteins orthologous to *E. coli *[(common (initials of endosymbiont)]. Comparisons are made for *E*. *coli *K12 with: *Buchnera aphidicola *str. Bp (A), *Candidatus Blochmannia floridanus *(B) and *Wigglesworthia glossinidia *(C).

We have considered if this hypothesis explains the basicity of small prokaryotic proteomes as well. We can exclude acquisition of basic proteins as the explanation because it is difficult to find any new genes gained by intracellular bacteria that would be absent from the closely related free-living relatives [32-34]. Usually, in the intracellular bacteria many genes deteriorate or are eliminated rather, leaving a conserved set of genes (e.g. encoding basic ribosomal proteins). The loss of dispensable genes coding for acidic proteins could lead to the shift of the whole proteome towards basicity. One could assume that conserved orthologous proteins present both, in the intracellular bacteria and in their free-living counterparts did not change their functions in the course of evolution and these sets have the same proportions of different classes of proteins. Then we should expect that these proteomes have similar pI distributions and are more basic than the proteins which are still present in the free-living bacteria but were lost in the intracellular organism. To check this we have analysed distribution of pI for three sets of proteins: present only in the free-living bacteria but not in the intracellular ones, and orthologous proteins common for the intracellular bacteria and their free-living counterparts analysed separately for these two groups of bacteria. The results of comparison of *Escherichia coli *K12 with: *Buchnera aphidicola *str. Bp, *Candidatus Blochmannia floridanus *and *Wigglesworthia glossinidia *are presented in Fig. 7. The set of orthologous proteins of these endosymbionts is very basic. However, the orthologous proteins of *E. coli *are even more acidic than the set of proteins present only in *E. coli *and lost in the endosymbionts. We have obtained similar results for seven other pairs of bacteria. Assuming that the *E. coli *proteome resembles the free-living ancestor of the endosymbionts we can estimate that 300 to 400 of its proteins (i.e. 55–65 percent of the common part of proteomes) have changed their pI from acidic to basic during transition to intracellular way of life. Therefore, in contradiction to the mitochondrial proteomes, the observed shift of small bacterial proteomes towards basicity cannot be explained by the gain or loss of some proteins in the proteome but by the shift of pI of many proteins.

**Figure 8:**
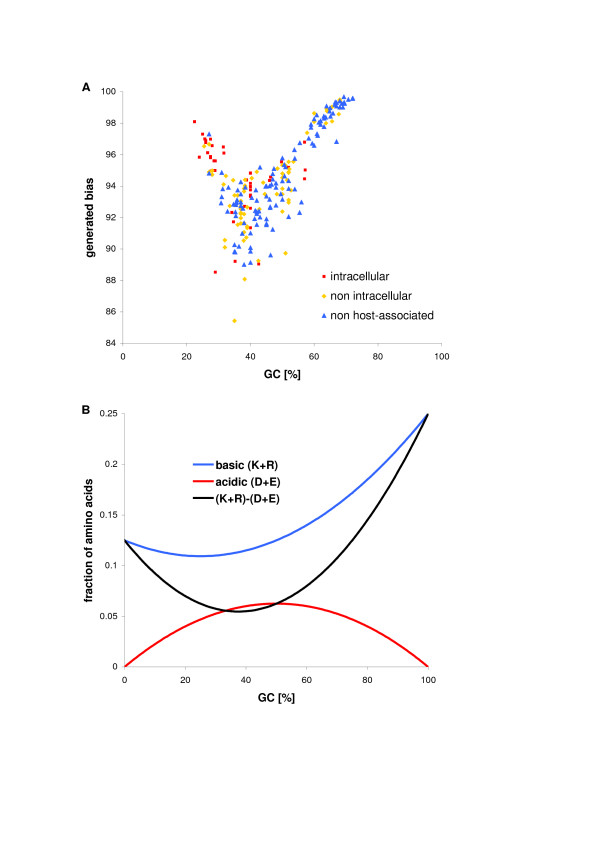
Isoelectric point and GC content. (A) The relationship between the computer-generated pI bias and the genomic GC content of prokaryotic organisms. The generated pI bias is the average calculated for 100 virtual proteomes generated for each organism assuming the same length distribution of proteins as in real proteomes and the amino acid composition calculated from the base composition characteristic for the given genome. (B) The relationship between the fraction of the basic and acidic amino acids and GC content.

To find the explanation of this shift, we have generated for each organism a proteome of the same length distribution of proteins as in its real proteome but with the amino acid composition calculated for the base composition characteristic for the given genome that reflects, to some extent, the composition generated by mutational pressure [35]. We generated 100 such proteomes for each organism and we averaged their pI bias. These proteomes show the equilibrium steady state of proteomes evolving only under mutational pressure without any selection constraints. All generated proteomes showed extremely high (higher than 85%) pI bias with average = 94% (Fig. 8A, see additional data file 6 for the *E. coli *example). Actually, according to the genetic code table, assuming equal frequencies of codons, representation of the acidic residues is lower than the basic ones. The relationship between the fraction of the basic or acidic amino acid and GC content is non-linear but any nucleotide composition favours basic residues especially for extreme GC content (Fig. 8B). The smallest difference between the fraction of coded basic and acidic amino acid is for 37% of GC. It is in agreement with the relationship obtained for the generated proteomes. The lowest pI bias falls between 30% and 40% of GC content. Furthermore, the points bifurcate towards the higher pI bias for the extreme values of GC content showing negative correlation below and positive correlation above 40% of GC content. The negative relationship is probably connected with the increase of basic lysine coded by AT-rich codons whereas the positive one – with the growth of basic arginine coded by GC-rich codons. Since any DNA composition (especially the one in equilibrium with mutational pressure) shifts proteomes towards basicity, we can assume that the acidic proteomes are subjected to strong selection pressure opposite to the mutational pressure. The more acidic a proteome is the stronger selection should be exerted on it. So far, the most acidic proteomes belong to extreme halophiles so we should expect very strong selection constraints on this group exerted by the hypersaline environment in which they live.

The results indicate that AT bias alone cannot explain the observed basicity of tiny proteomes. We have not observed any correlation for these virtual proteomes between the pI bias and proteome size also. It seems that the acceleration of mutation accumulation itself could be responsible for the basicity of tiny proteomes. It is in agreement with the well-documented higher evolutionary rate of intracellular bacteria than their free-living relatives. It has been suggested that the higher rate of evolution results from enhanced mutation rate [36] and/or Muller's ratchet effect or the easier fixation of mutations by genetic drift in small asexual populations [37-46]. Accordingly, the increase of basicity of proteomes and the increase of mutational AT bias would be paralelly ongoing phenomena resulting from the higher evolution rate of genomes of intracellular bacteria. The elevated AT bias probably results from the elimination of genes encoding DNA repair and recombination-associated enzymes or at least from the decrease in their efficiency in intracellular organisms [31,32,37,47-49]. These enzymes would normally correct the error-related tendency toward AT enrichment, for example the deamination of cytosine to uracil, which is then replaced by thymine.

To confirm our explanation we have analysed 39 sets of much conserved orthologous proteins present in each of 100 selected prokaryotic organisms, representing quite uniformly the values of the pI bias. We have assumed that if the increase in substitution rate affects the pI distribution we should observe a positive correlation between the pI of these proteins and the pI bias of the whole proteomes. Actually, for 36 of them we have found such statistically significant correlation (see additional data file 12). Three sets, for which the correlation was not significant, represent the most basic ribosomal proteins and maybe their further shift was improbable. The results indicate that even much conserved proteins subjected to strong selectional constraints follow the global trend in the pI distribution. Such a relationship concerns probably many other proteins because analyses of pI of Clusters of Orthologous Groups (COGs) showed that proteins of only few clusters are conserved and stay in the same acidic or basic set while the majority of them jump between the two sets [15]. Many of these promiscuous proteins are membrane proteins that have direct contact with the external environment and may be considered adaptive proteins. Therefore it would favour the hypothesis about the selection for pI changes of particular proteins. However, it does not fully explain the basicity of small prokaryotic proteomes because the changes of pI concern also many non-membrane and much conserved proteins. (Relationships between pI and other phenomena we discussed in additional data file 13.)

It would be interesting to investigate the relationship between the change of the pI distribution of proteomes and the transition of organisms from the free to intracellular way of life on different stages of genome reduction. Very insightful results concerning this subject would give sequencing and analysis of reduced genomes of bacterial endosymbionts identified in eukaryotic hosts, e.g. a cyanobacterium in amoeba *Paulinella chromatophora*, a cyanobacterium *Cyanothece *in diatom *Rhopalodia gibba*, a Gram-negative bacterium in diatom *Pinnularia nobilis *and many different bacterial endosymbionts found in various species of insects. Additional interesting results would come from analyses of nucleomorphs – small, reduced eukaryotic nuclei found in certain plastids present in some groups of algae such as cryptomonads and chlorarachniophytes. Moreover, it is interesting to estimate how many mutations are required or should be accepted to remodel one proteome to another in the aspect of their pI. However, many factors such as length, amino acid composition, pI of the original proteins, mutation rate and patterns of nucleotide substitutions and resulting patterns of amino acid substitutions should be taken into account.

## Conclusion

Although the distribution of pI of proteins in proteomes is generally bimodal, different species have different abundance of acidic and basic proteins that is correlated with ecology of these species, especially with habitat, relation to the host cell and salinity of the environment. The pI distribution is also related with taxonomy of organism but only on higher taxonomical levels and subcellular localization of some proteomes. The other factor that shapes the distribution is the rate of mutations accumulation. The rate is higher in intracellular organisms than in free-living ones and it is responsible for the basicity of tiny proteomes that explains the observed relationship between the proteome size and pI of proteomes.

## Methods

Proteomic sets were downloaded from different sources (see additional data file 1 for details): National Center for Biotechnology Information, European Bioinformatics Institute, DOE Joint Genome Institute, Broad Institute of MIT and Harvard, The Institute for Genomic Research (TIGR), Welcome Trust Sanger Institute, Ensembl project, Stanford Genomic Resources, Virginia Commonwealth University, National Institute of Genetics, Japan, PlasmoDB, International Fugu Genome Consortium, Genoscope, DictyBase and SilkDB. We have not analysed proteomes containing less than 10 proteins. In sum we have analysed 1784 proteomes grouped in the following sets encoded by: chromosomes in Archaea (22), Bacteria (210) and Eukaryota (63), mitochondria (720), plastids (42), prokaryotic plasmids (319), phages (245) and viruses (163). Note that in the paper, in the proteomes of Archaea, Bacteria and Eukaryota only chromosome-encoded proteins are referred to. Sequences of nuclear-encoded proteins with annotated transit peptides and targeted to mitochondria (2021) or plastids (1173) were downloaded from UniProt database [50]. Proteomes of 12 subcellular localizations (in sum 13,039 proteins) were extracted from non-redundant datasets from DBSubLoc database [51].

Isoelectric points were calculated using the standard iterative algorithm [52,53] that gives relatively precise results of pI calculations for raw protein sequences [1,2]. The algorithm is used in the Compute pI/Mw tool at the ExPASy server [54]. The source code of the algorithm was kindly supplied by Elisabeth Gasteiger.

Each proteome was divided into two sets named the acidic and the basic one according to the pI value of its proteins. To find the point of division of proteomes for the two sets, we ranked proteins according to their pI values, cut off 10% tails of both acidic and basic proteins and the rest of the proteome distribution was scanned for the largest difference in pI between two neighbouring proteins. The point of division between acidic and basic proteins was set in the middle of this distance. Because of statistical reasons, the procedure was applied only to big chromosome-encoded proteomes of Archaea, Bacteria and Eukaryota. Because of the narrow range and the universality of the mid-point, we assumed as the division point of smaller proteomes encoded by plasmids, mitochondria, plastids, viruses and phages the median of the mid point calculated for the big proteomes which equals 7.41.

Proteomes were characterized by the average pI and the average length of proteins separately for the basic and the acidic sets of proteins and by the "pI bias" (b) describing the relation between the number of proteins in the basic set and the number of proteins in the acidic set: b = 100· (N_basic_-N_acidic_)/(N_basic_+N_acidic_), where N_acidic _and N_basic _denote the numbers of proteins in the acidic and the basic sets, respectively.

The different sets of proteomes were clustered by the UPGMA (Unweighted Pair-Group Method Arithmetic Averages) method based on the median of the pI bias. The clustering was performed with the neighbour program from the PHYLIP 3.6 package [55]. To evaluate the reliability of specific clades in UPGMA trees we created 10 000 matrices of median of the pI bias generated by the random sampling of 2/3 members of each group of proteomes (subsampling method). Then we applied the neighbor and consense programs (from the PHYLIP package) to calculate the percent of randomised trees containing a given clade. Moreover, the WeightLESS program [56] was used to perform the WLS-LRT (Weighted least-squares likelihood ratio test) and F-test.

All analysed prokaryotic proteomes were classified according to five ecological signatures: habitat (aquatic, host-associated, multiple, specialized, terrestrial), relation to host cell (extracellular, free living, free living/extracellular, free living/intracellular, intracellular), salinity (extreme halophilic, moderate halophilic, mesohalophilic, non-halophilic), oxygen (aerobic, anaerobic, facultative, microaerophilic) and temperature requirements (hyperthermophilic, mesophilic, psychrophilic, thermophilic). The classification was based on the data published on the NCBI web site, papers related to the sequenced genomes and other sources. A given species was assigned only to one subgroup, the most typical for its ecological property. To analyse the relationship between the pI bias and ecological classification of proteomes the analysed proteomes of particular ecological subgroups were distributed among three classes of the pI bias ('acidic': -90% to -30%, 'neutral': -30% to 30% and 'basic': 30% to 90%). Then the observed numbers of proteomes in the given class were compared to the expected ones by χ^2 ^test.

Sets of orthologous proteins of prokaryotic organisms were downloaded from Microbial Genome Databse – MBGD [57].

The non-parametric Kruskal-Wallis test and t-Student test were applied accordingly to determine statistical significance of tested hypotheses. The Benjamini-Hochberg multiple comparisons procedure for controlling the false discovery rate was used [58].

## Authors' contributions

JK started this work and generated preliminary results, carried out analyses, collected data about ecological classification of organisms and wrote scripts in Perl. MP supervised the project, wrote this manuscript, downloaded the data, carried out analyses and wrote scripts in Perl. DM wrote scripts in Perl and generated sequence data. MK wrote this manuscript and contributed substantially to the final manuscript. PB performed some statistical analyses and wrote scripts in the R environment. NP and KS collected data about ecological classification of organisms and generated sequence data. MRD wrote programs in C++ calculating isoelectric point and generating random amino acid sequences. SC drafted manuscript, provided overall guidance and interpretation of results. All authors read and approved the final manuscript.

## Supplementary Material

Additional file 2 PI distribution for prokaryotic proteomes. Left panel: histograms of pI values at 0.1 unit intervals (X axis: class of pI; Y axis: percent); right panel: relationships between the logarithm of length of proteins (Y axis) and their pI (Y axis). Black points represent the set of acidic proteins while grey ones – the set of basic proteins. Click here for file

Additional file 3 PI distribution for prokaryotic proteomes. Left panel: histograms of pI values at 0.1 unit intervals (X axis: class of pI; Y axis: percent); right panel: relationships between the logarithm of length of proteins (Y axis) and their pI (Y axis). Black points represent the set of acidic proteins while grey ones – the set of basic proteins. Click here for file

Additional file 4 PI distribution for eukaryotic proteomes. Left panel: histograms of pI values at 0.1 unit intervals (X axis: class of pI; Y axis: percent); right panel: relationships between the logarithm of length of proteins (Y axis) and their pI (Y axis). Black points represent the set of acidic proteins while grey ones – the set of basic proteins. Click here for file

Additional file 5 Relationship between the average pI of proteins and the pI bias for acidic (A) and basic sets (B). Click here for file

Additional file 6 The real and artificial proteomes of *Escherichia coli *K12. Left panel: histograms of pI values at 0.1 unit intervals (X axis: class of pI; Y axis: percent); right panel: relationships between the logarithm of length of proteins (Y axis) and their pI (Y axis). Click here for file

Additional file 7 PI distribution of mitochondrial proteomes belonging to Fungi (A), Protista (B) and Viridiplantae (C) prepared separately for proteins common for Metazoa and the analyzed group (present in Metazoa) and absent from Metazoa. Click here for file

Additional file 8 PI distribution for nuclear-encoded proteins targeted to organelles: plastid and mitochondrion. Left panel: histograms of pI values at 0.1 unit intervals (X axis: class of pI; Y axis: percent); right panel: relationships between the logarithm of length of proteins (Y axis) and their pI (Y axis). Black points represent the set of acidic proteins while grey ones – the set of basic proteins. Transit peptides, premature proteins (i.e. the whole sequence) and mature proteins (i.e. without transit peptide) were analysed separately. Mitochondrial proteomes were divided to various taxonomical groups. Click here for file

Additional file 9 PI distribution for proteomes of different subcellular localization. Left panel: histograms of pI values at 0.1 unit intervals (X axis: class of pI; Y axis: percent); right panel: relationships between the logarithm of length of proteins (Y axis) and their pI (Y axis). Black points represent the set of acidic proteins while grey ones – the set of basic proteins. Click here for file

Additional file 10 Ecological changes and their relationships with the pI bias of proteomes in closely related organisms. Click here for file

Additional file 11 Relationship between the pI bias and logarithm of proteome size for archaeal, bacterial and eukaryotic organisms. Click here for file

Additional file 12 Characteristics of 39 sets of conserved orthologous proteins present in all selected 100 prokaryotic organisms. The correlation coefficient was calculated between the pI of proteins and the pI bias of the whole proteomes. Click here for file

Additional file 13 Discussion about relationships between pI and other phenomena Click here for file

Additional file 1 List of analysed proteomes. Click here for file
