# Supplementary figures and images for: The relationships between the isoelectric point and: length of proteins, taxonomy and ecology of organisms
Source: BMC Genomics. 2007 Jun 12;8:163. doi: 10.1186/1471-2164-8-163 (PMC1905920; doi:10.1186/1471-2164-8-163)

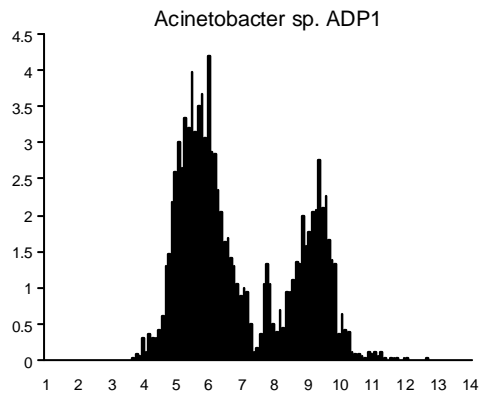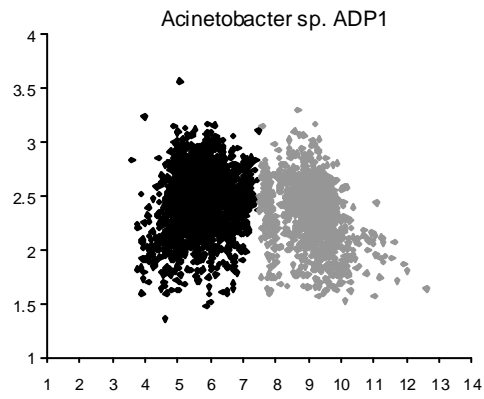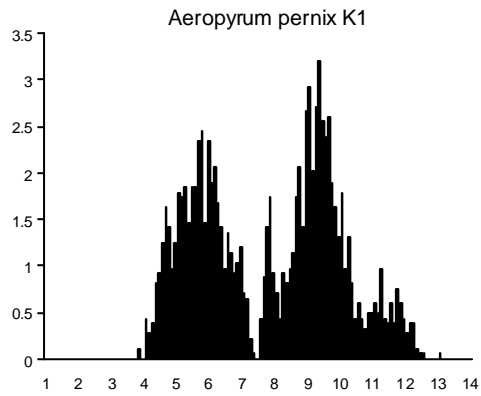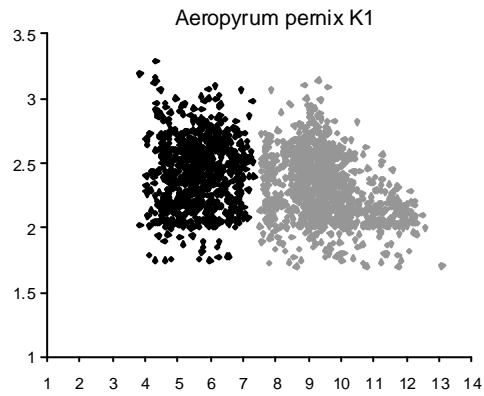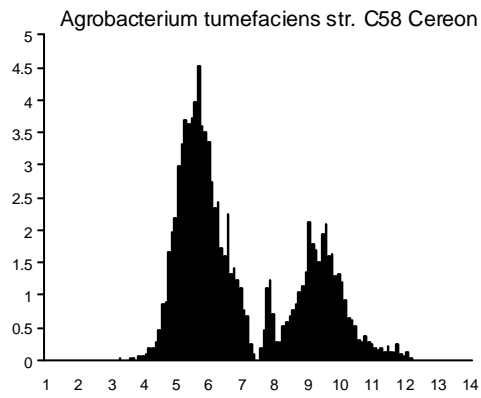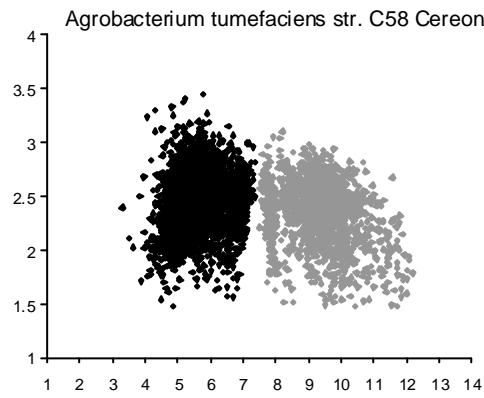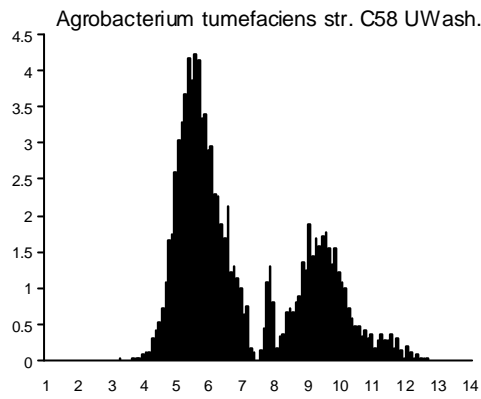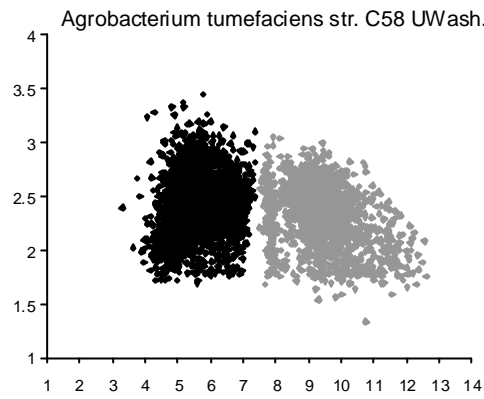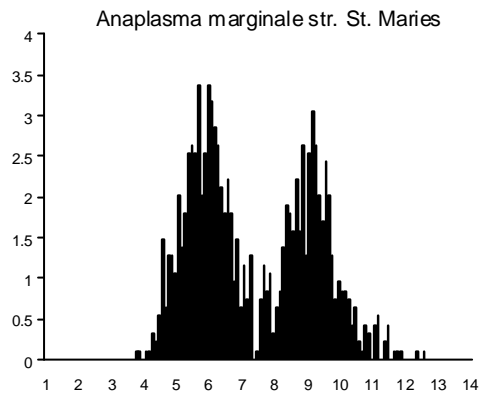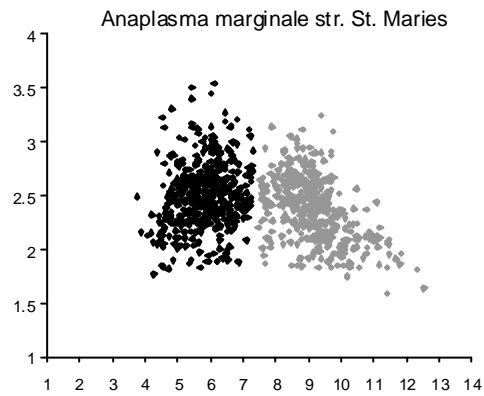

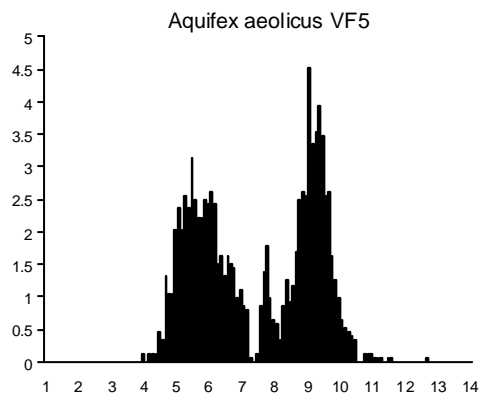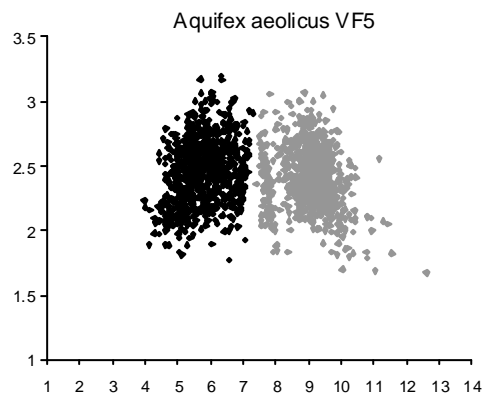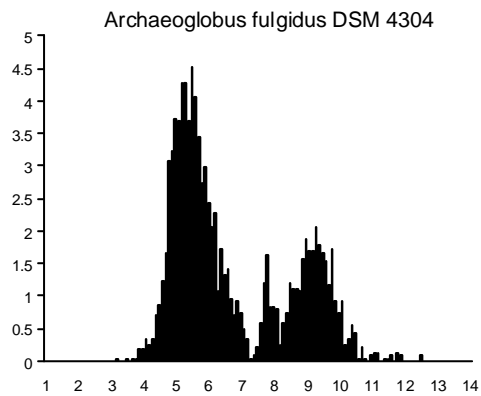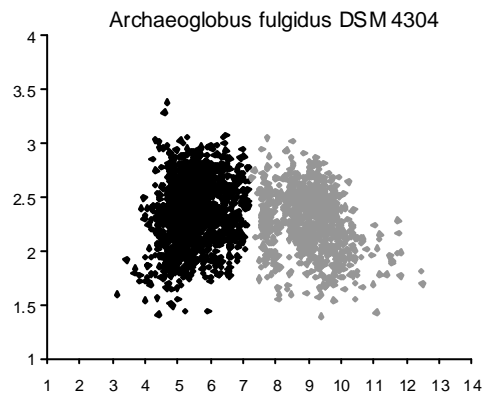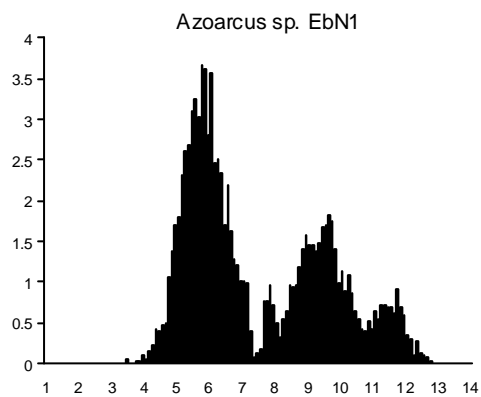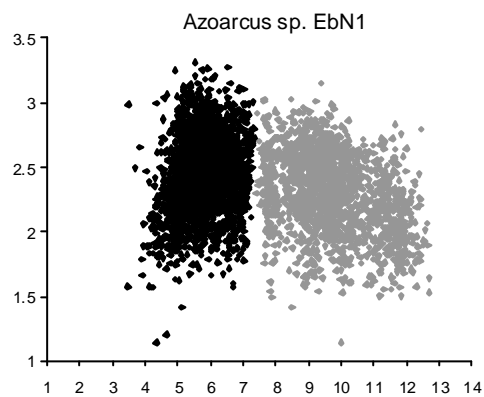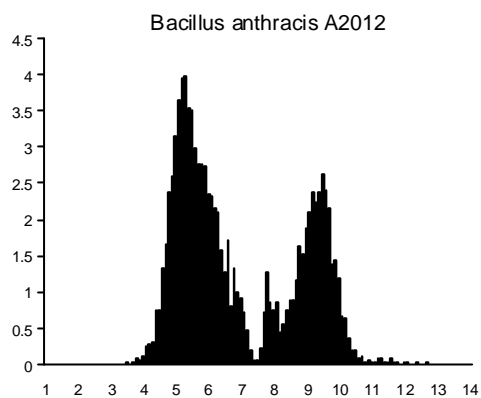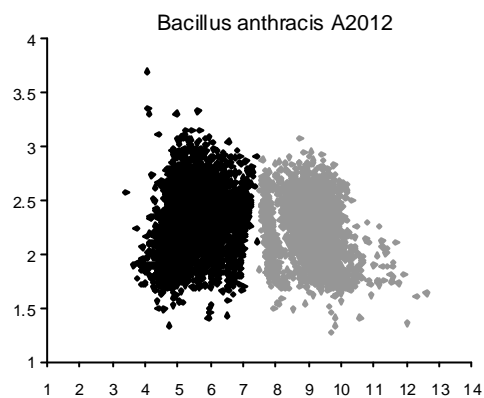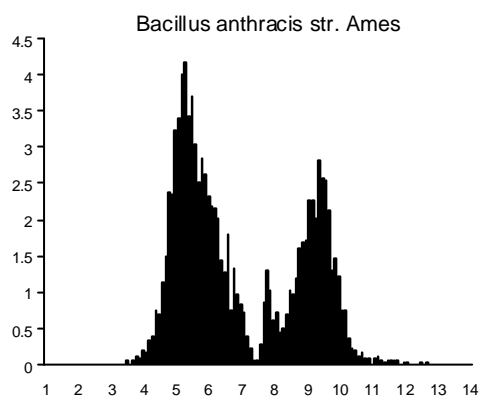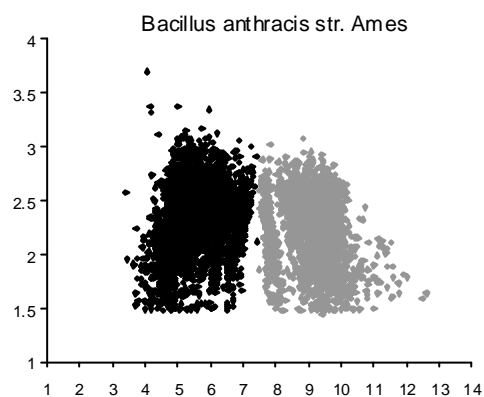

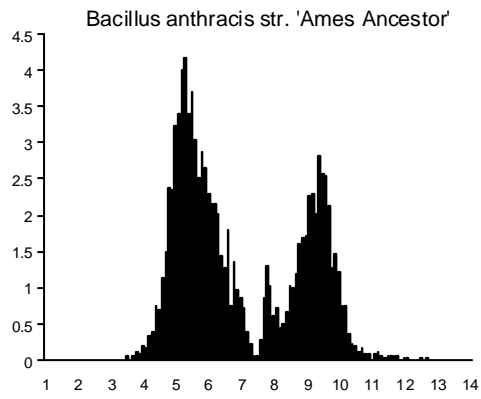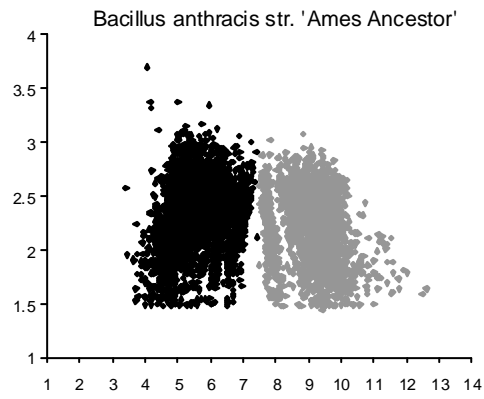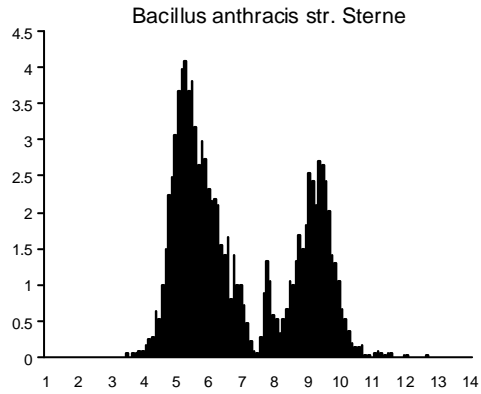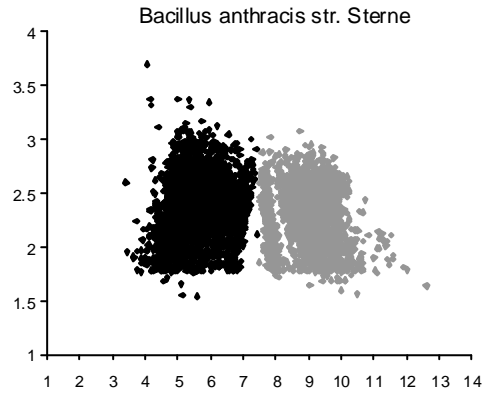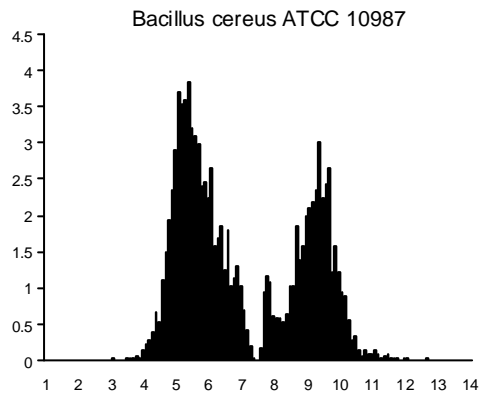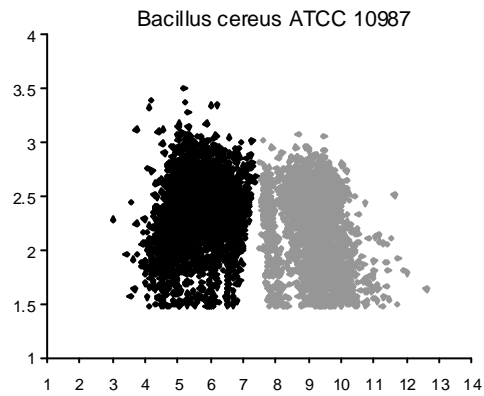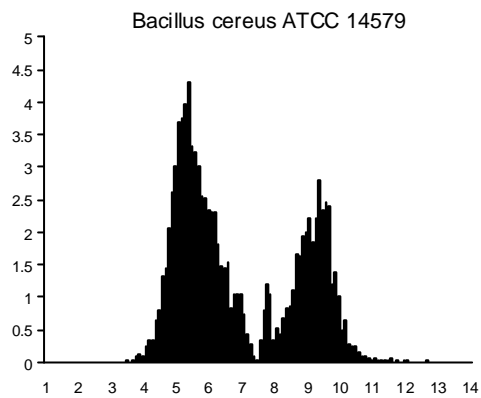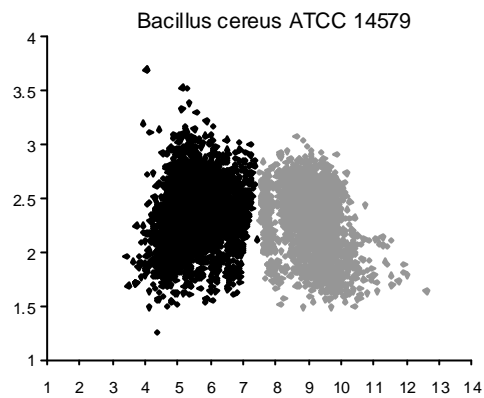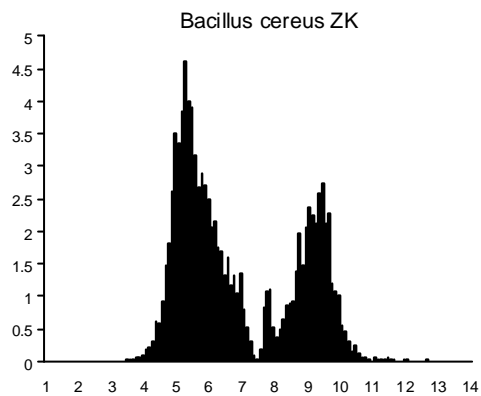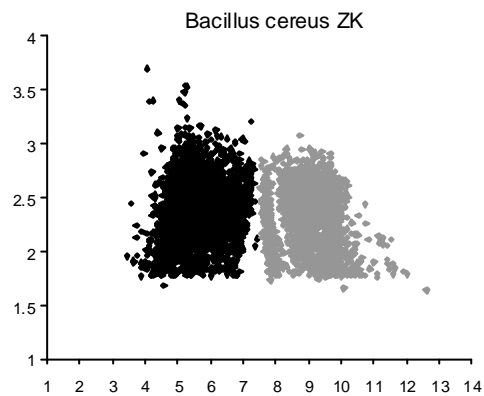

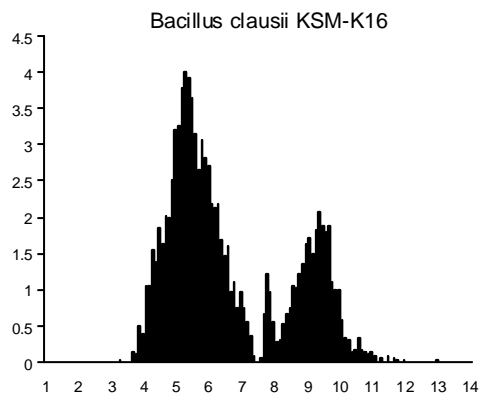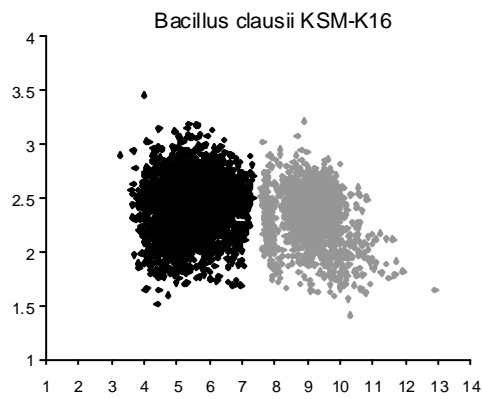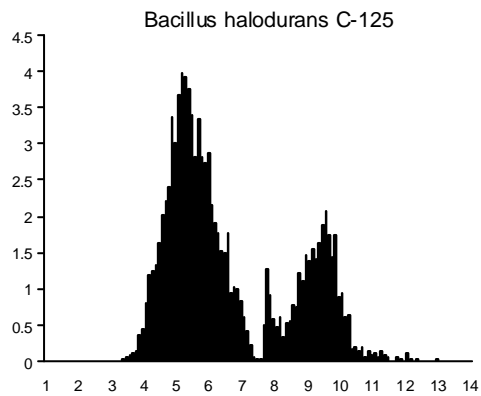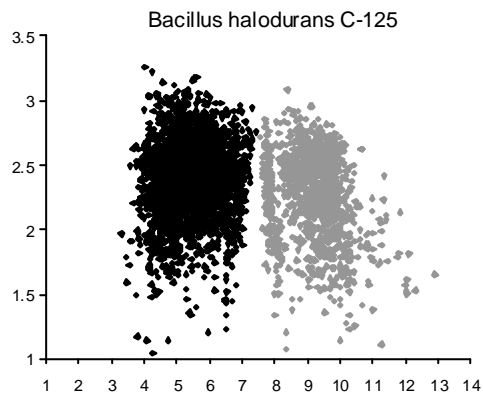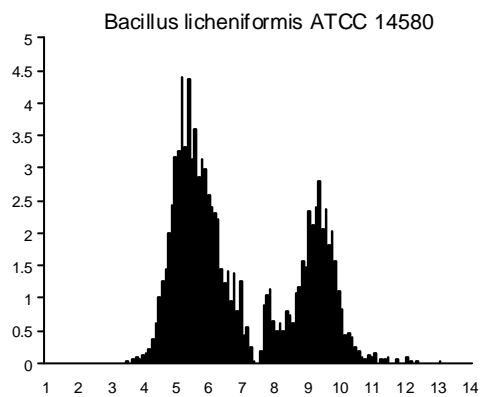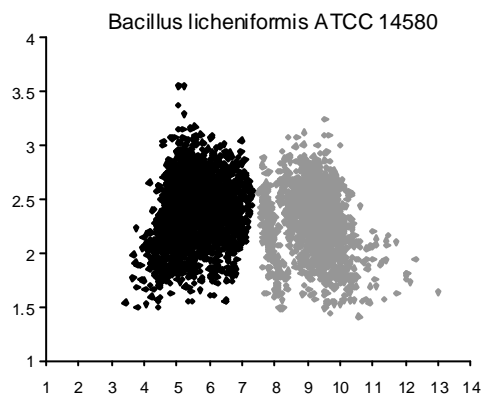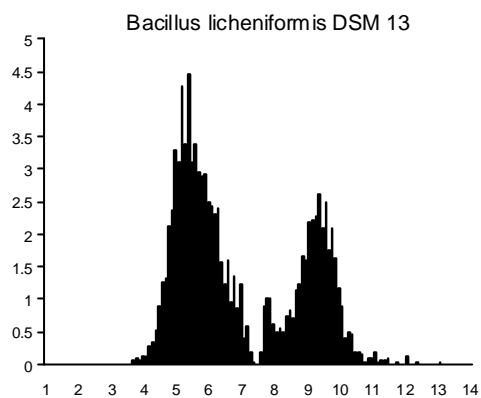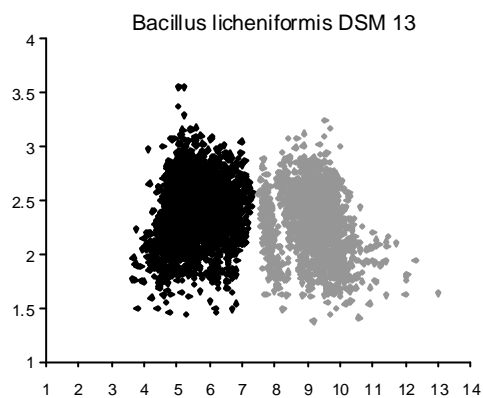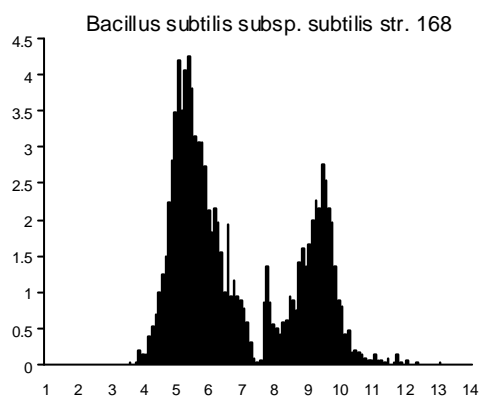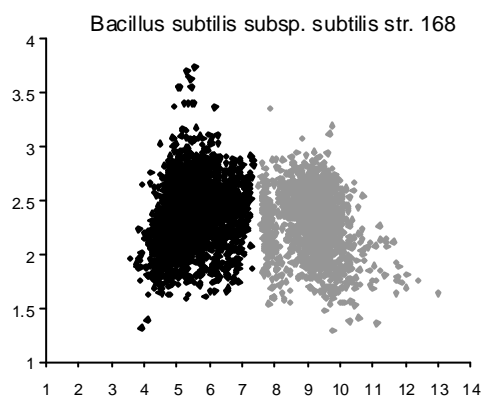

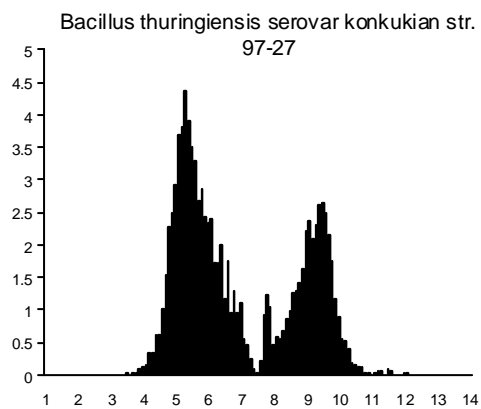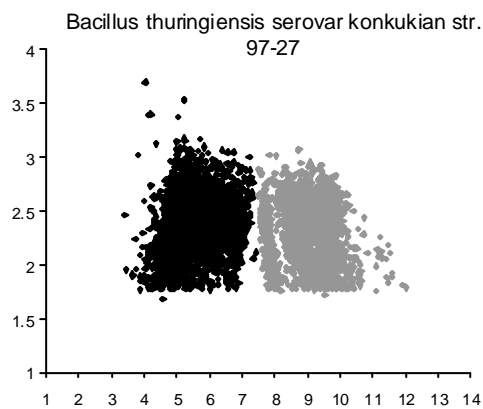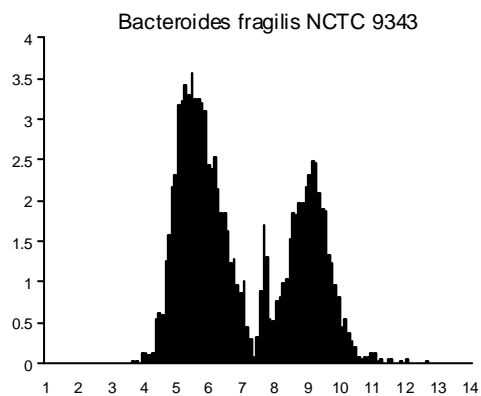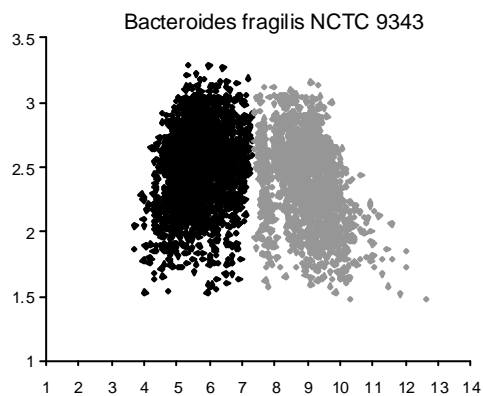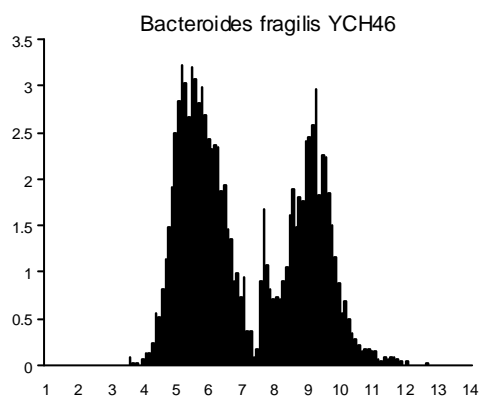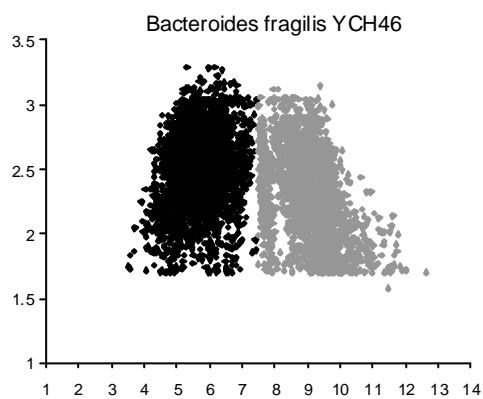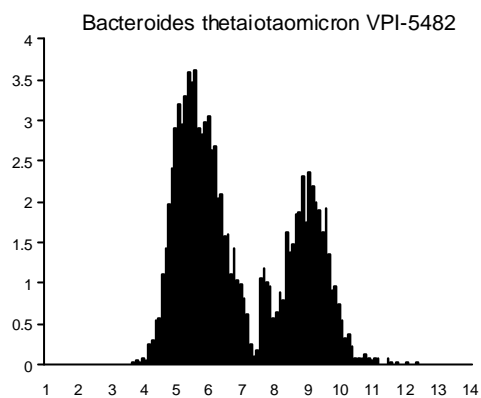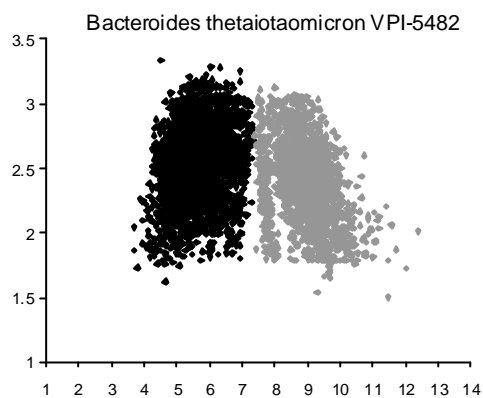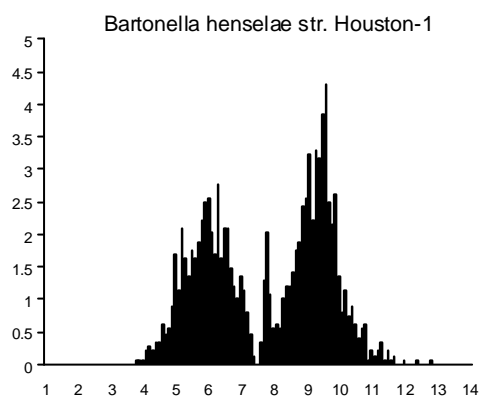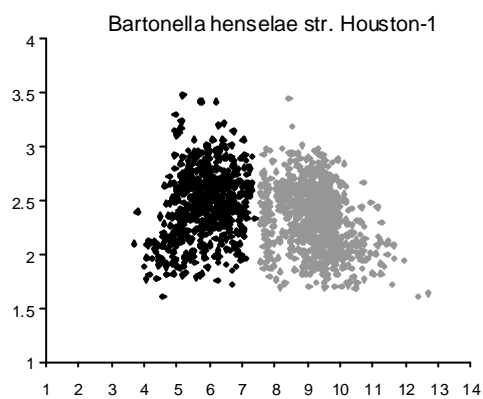

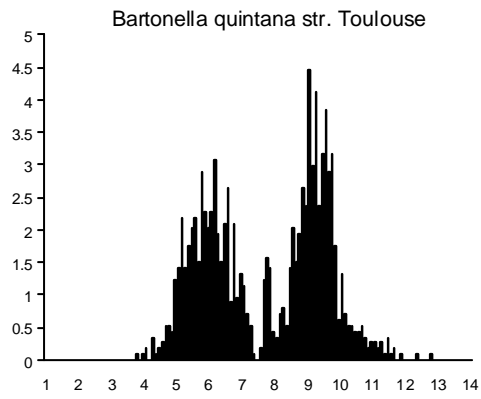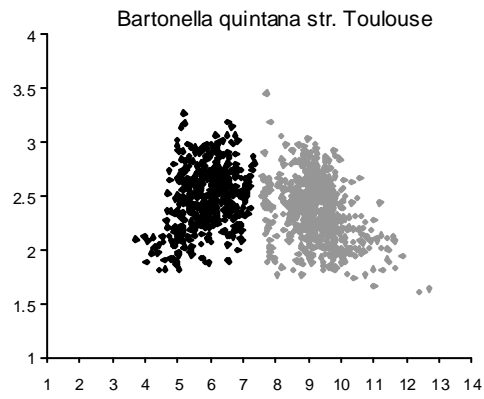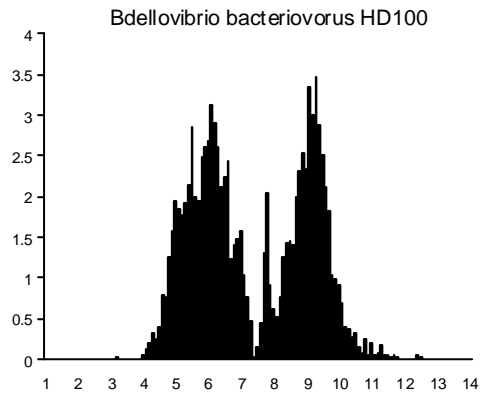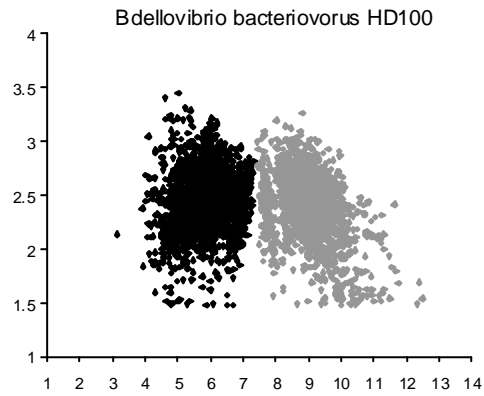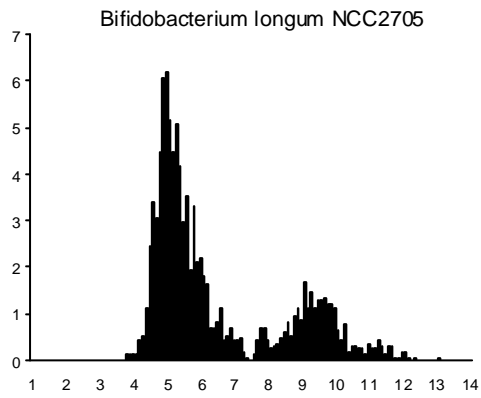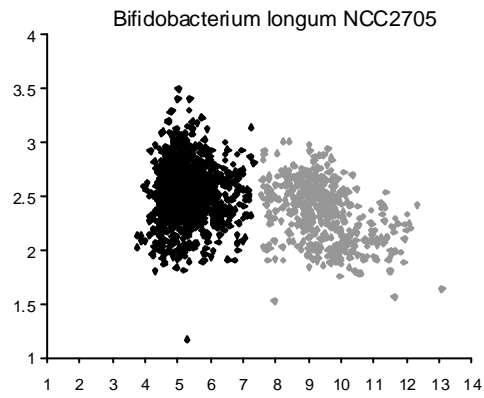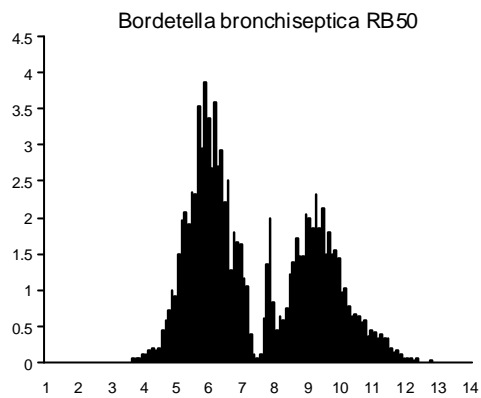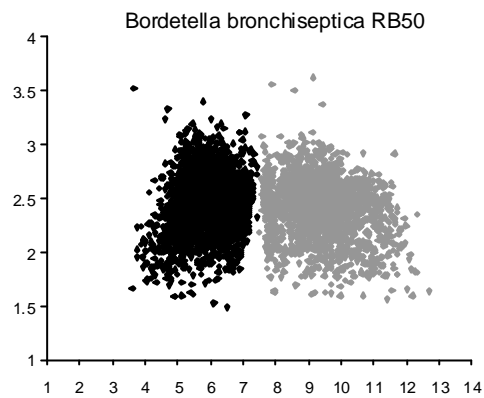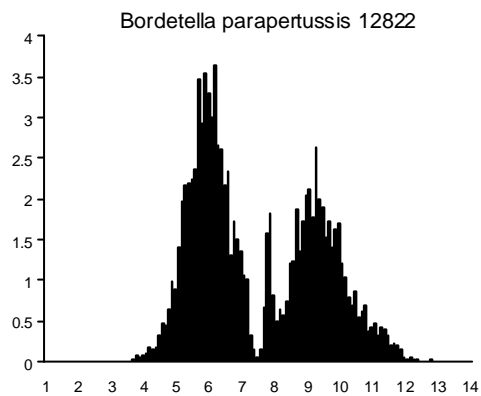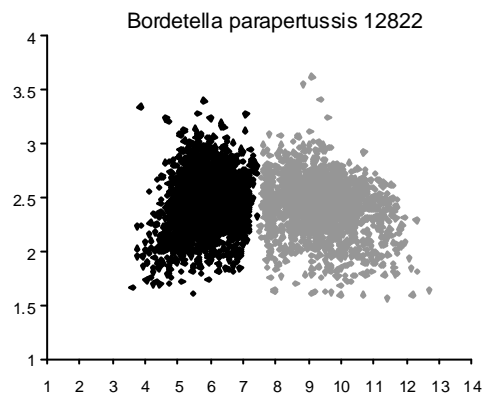

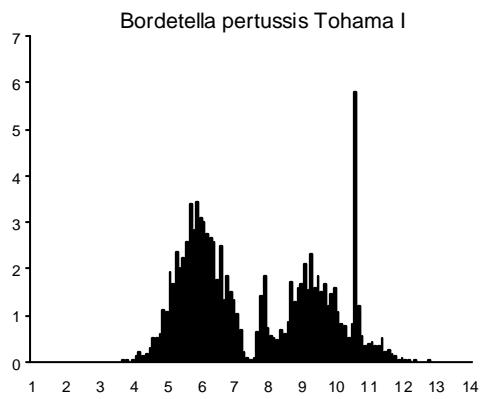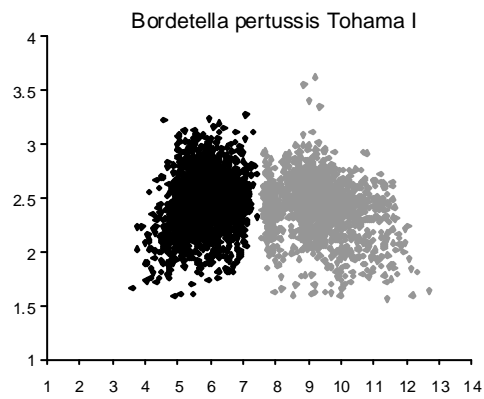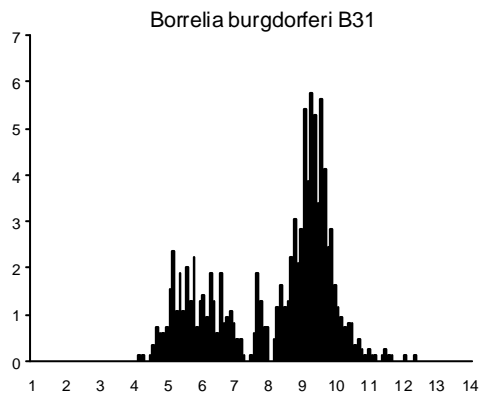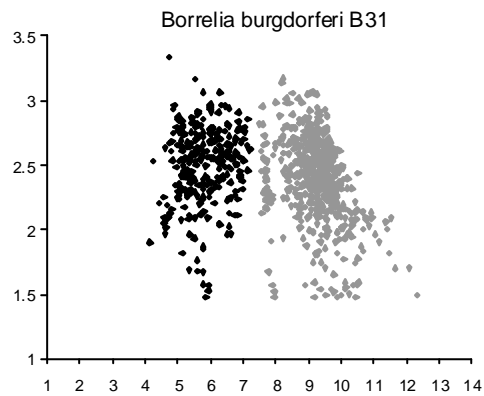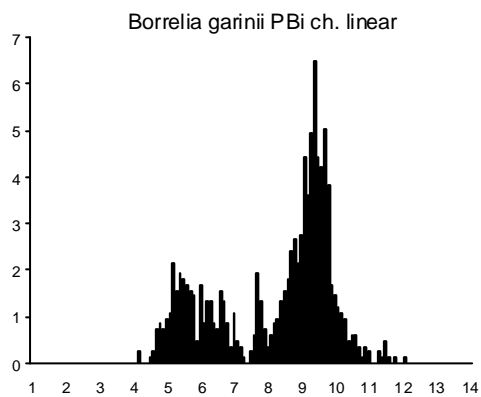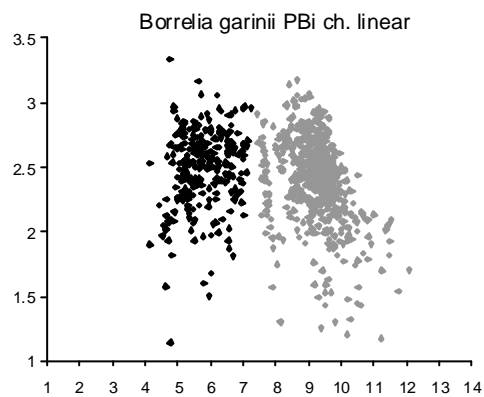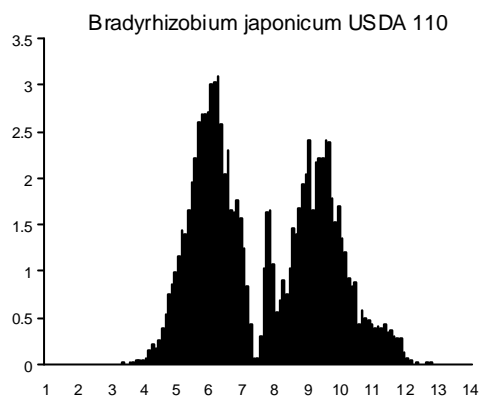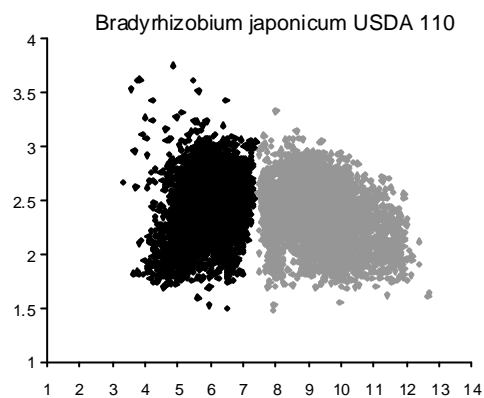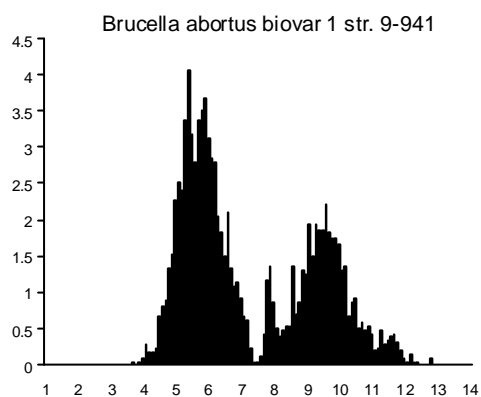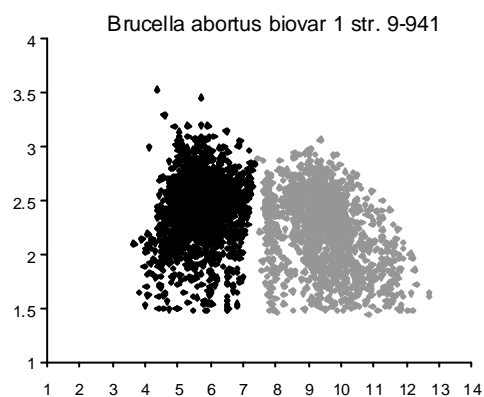

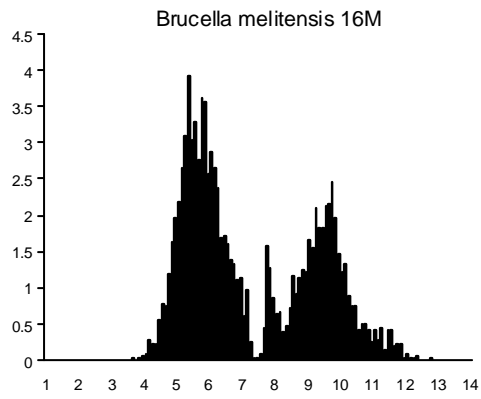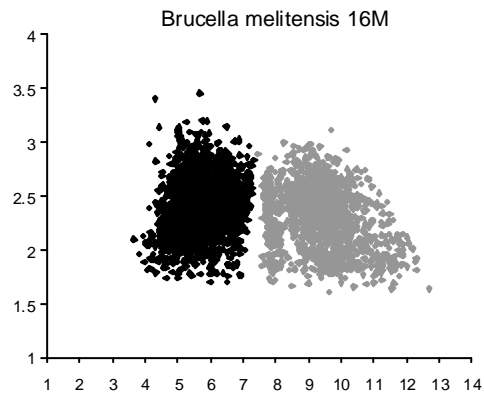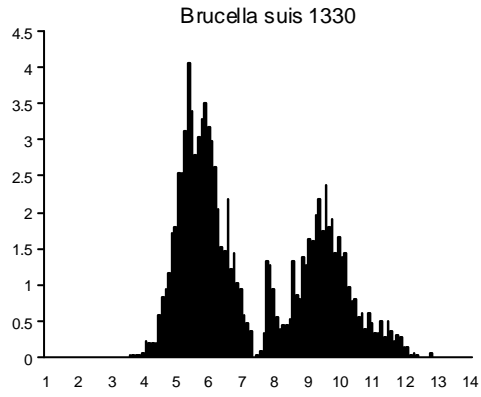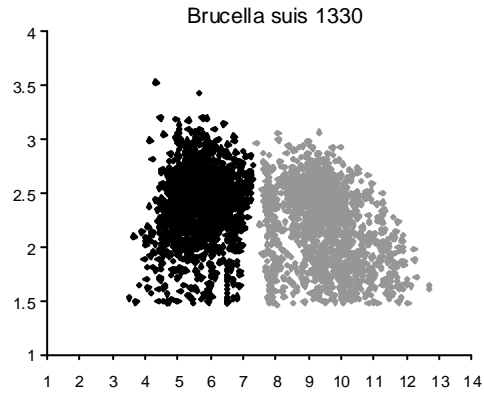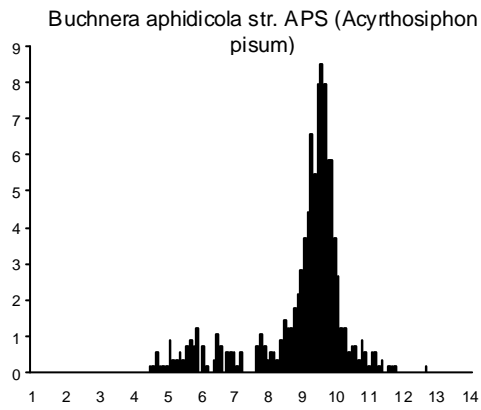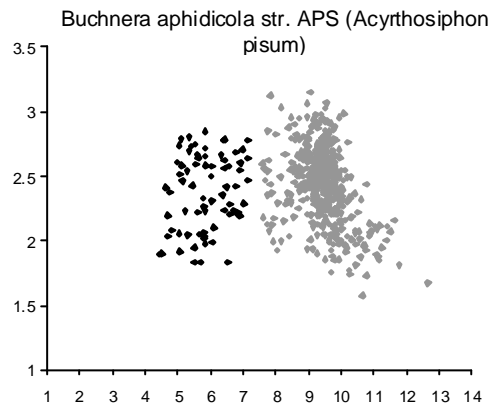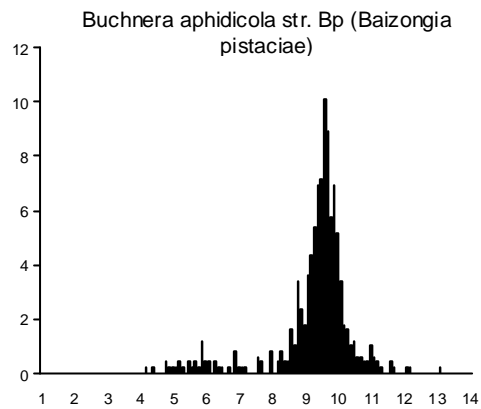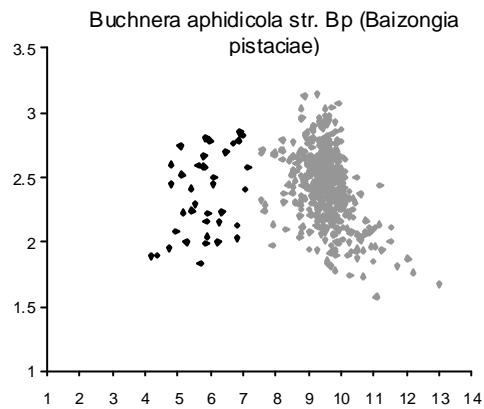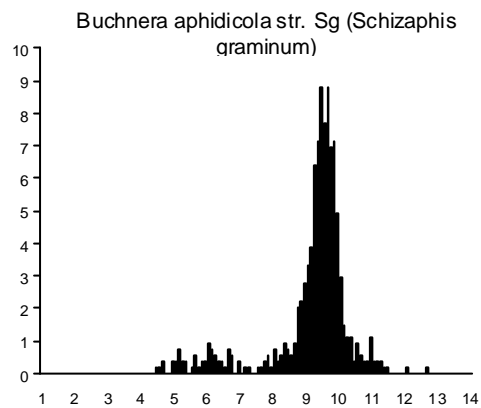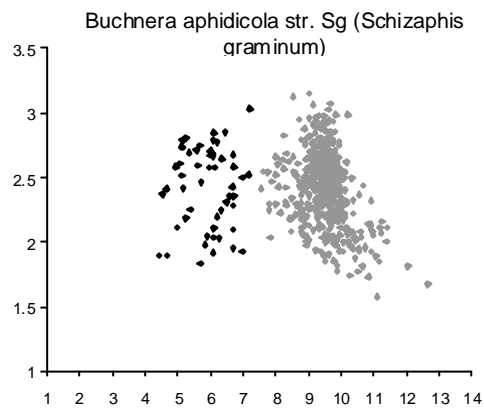

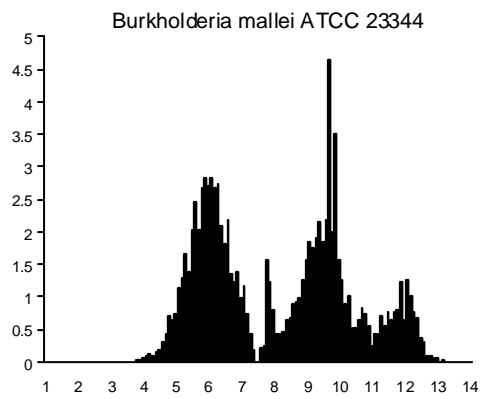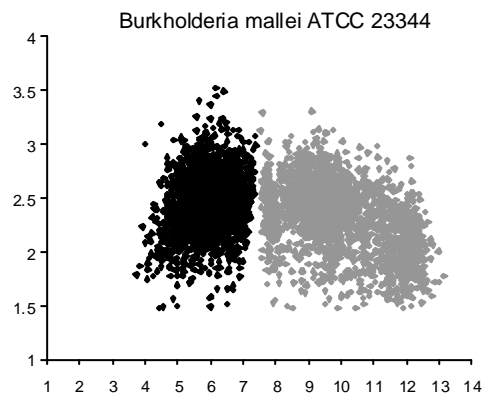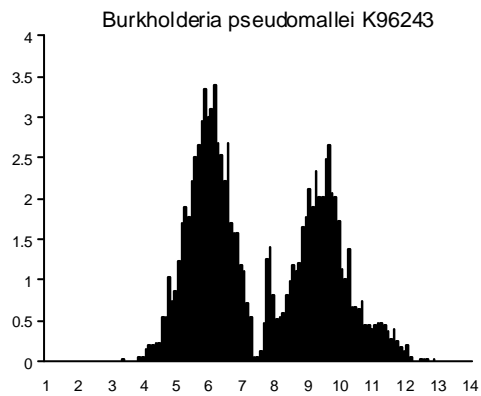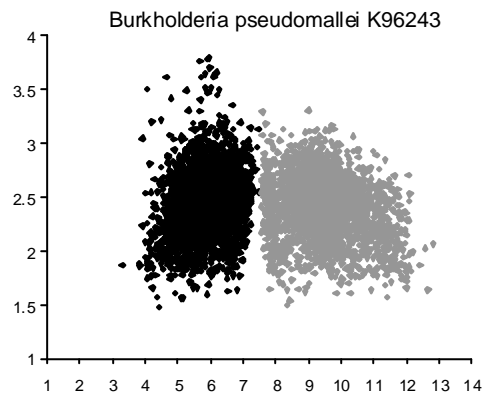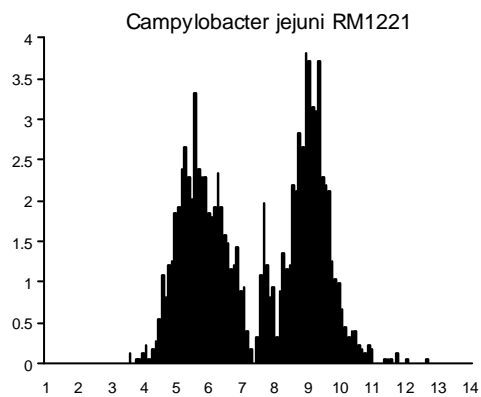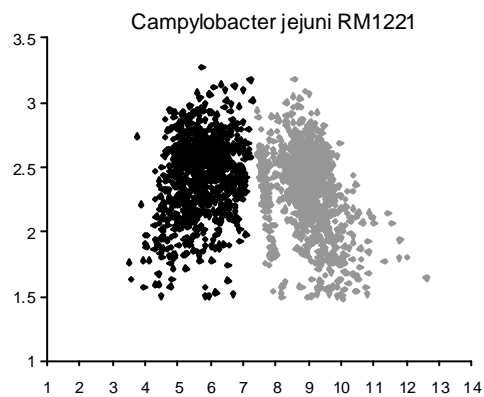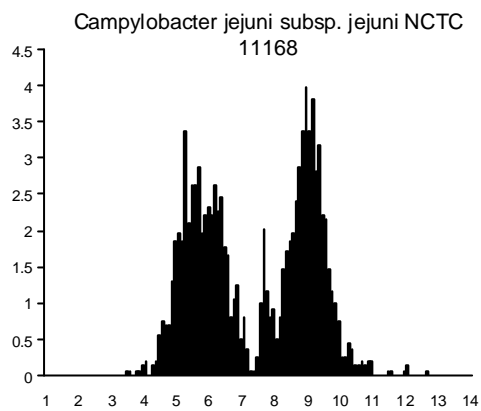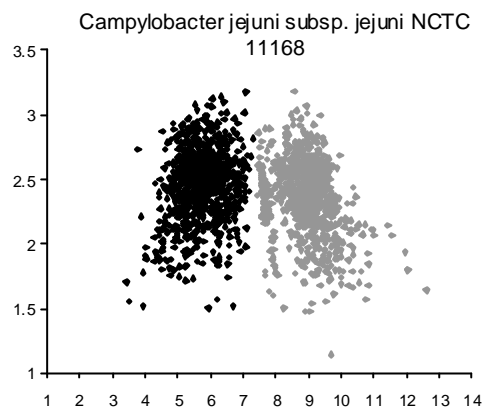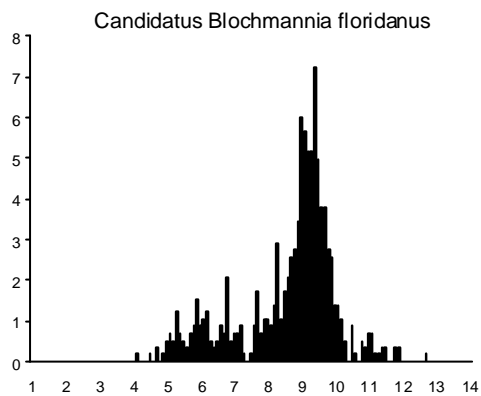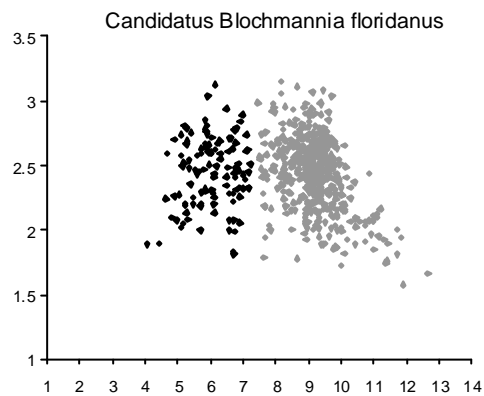

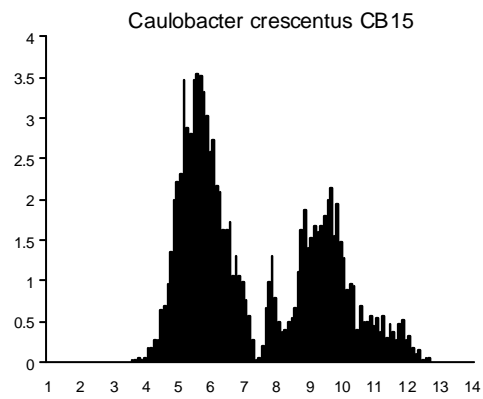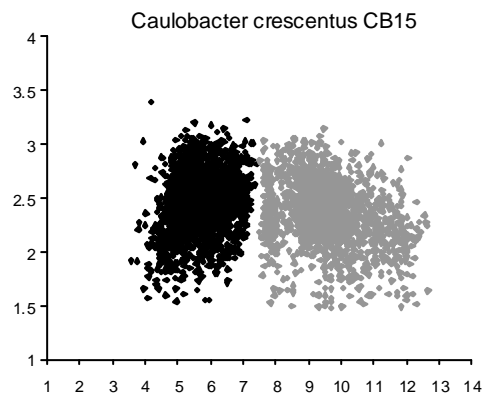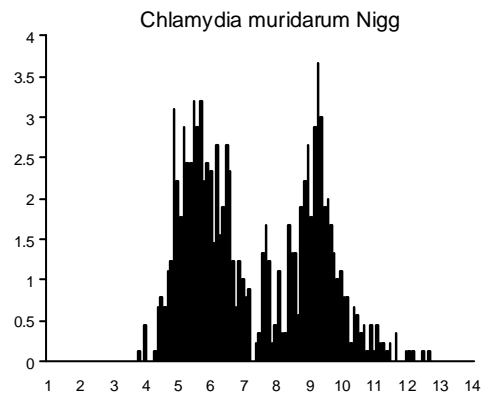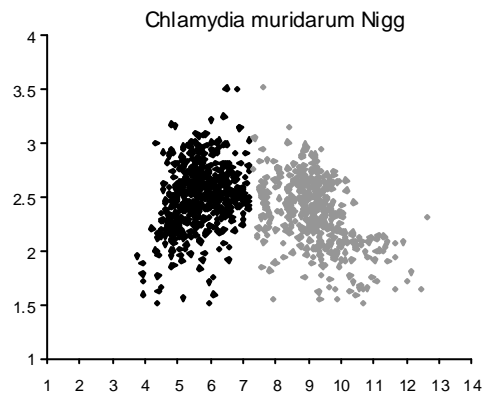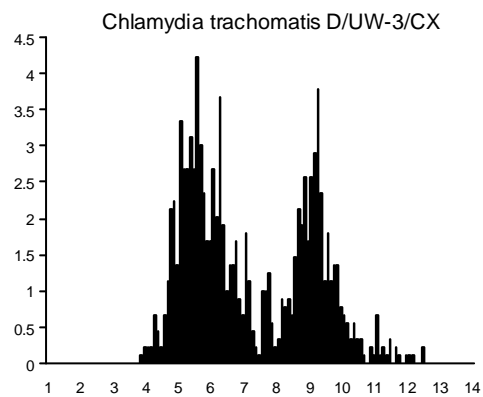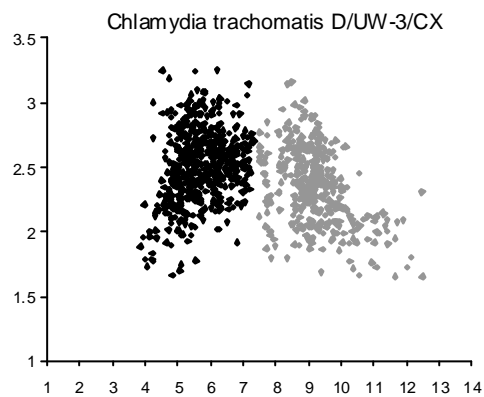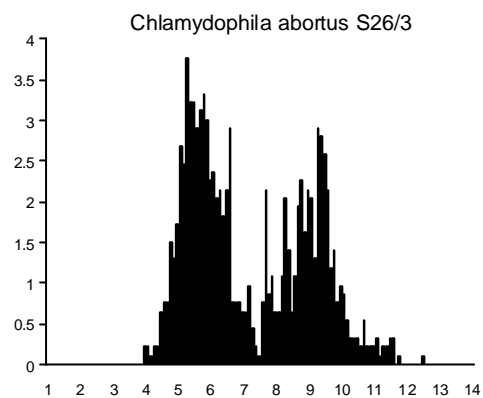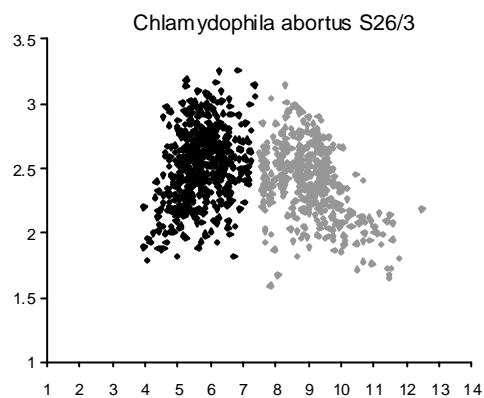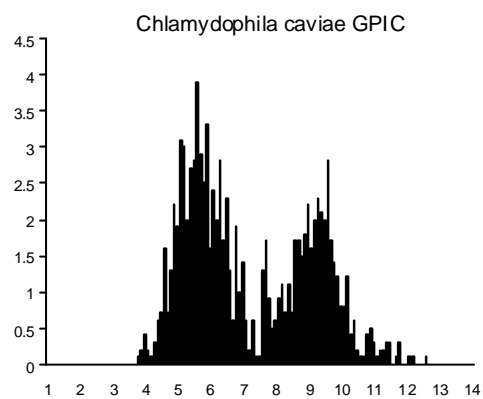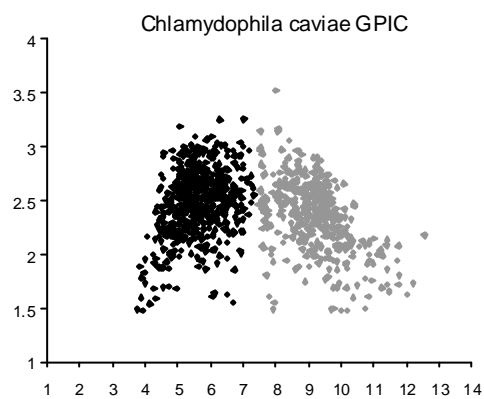

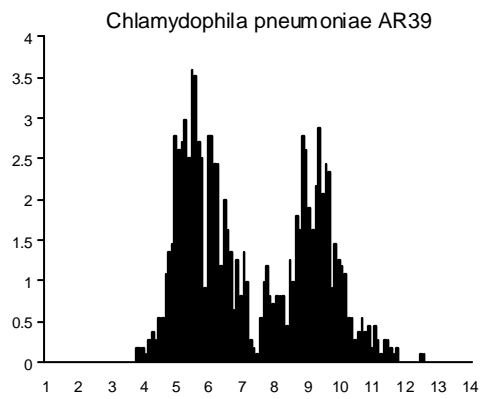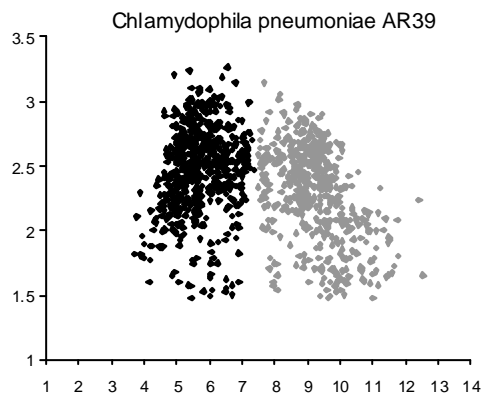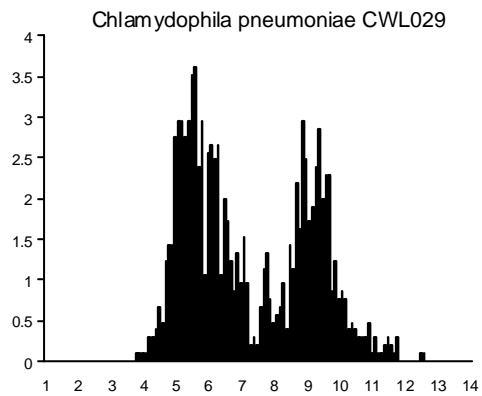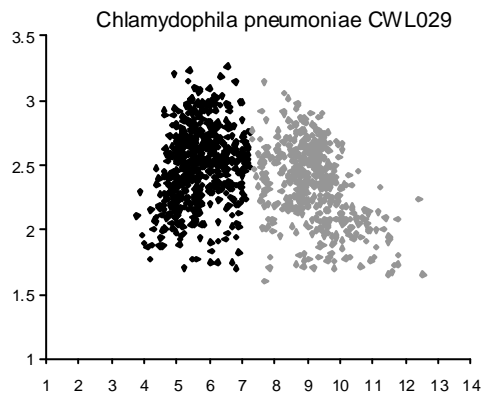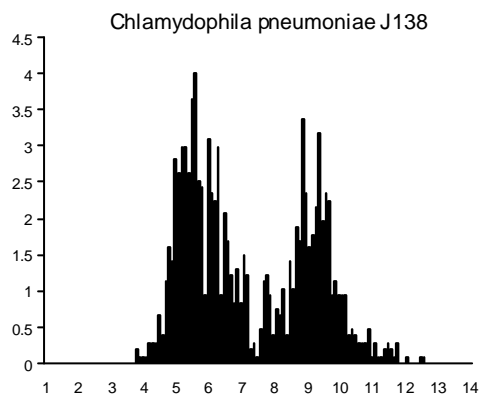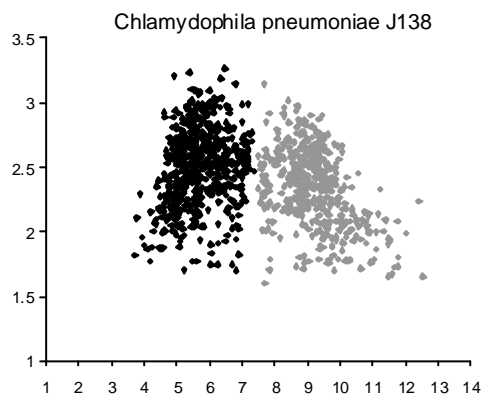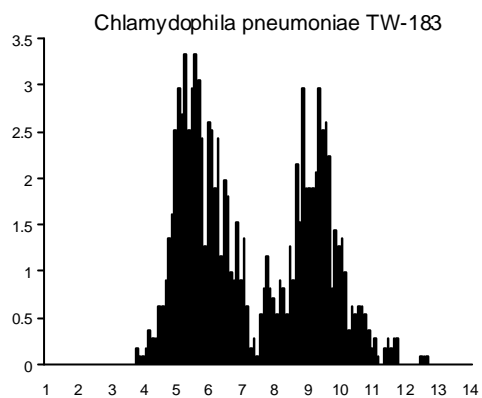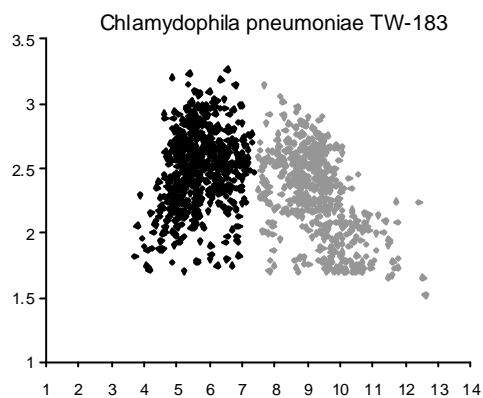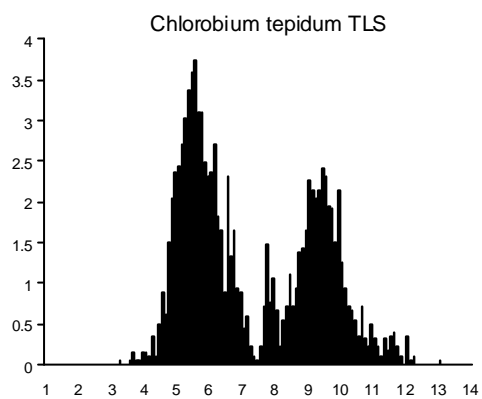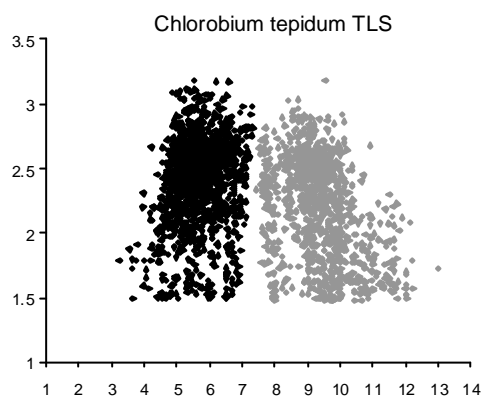

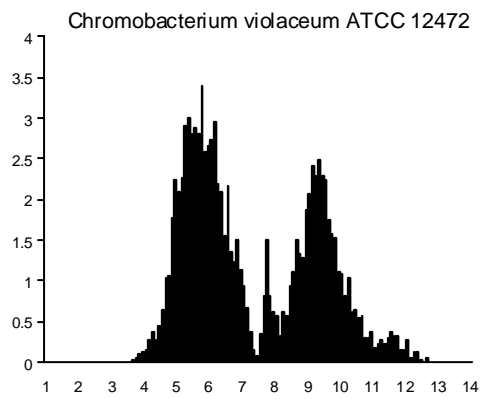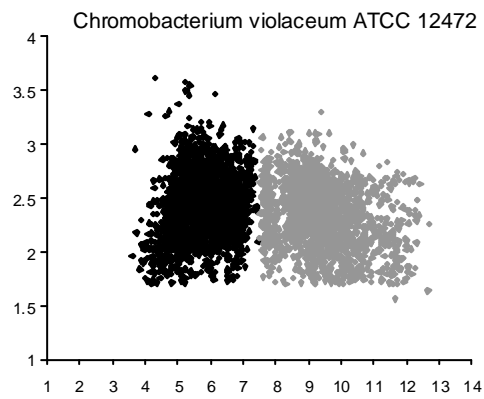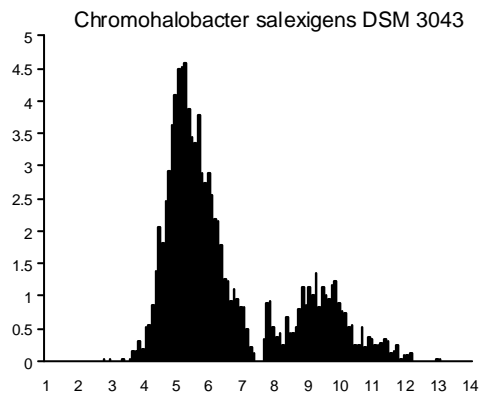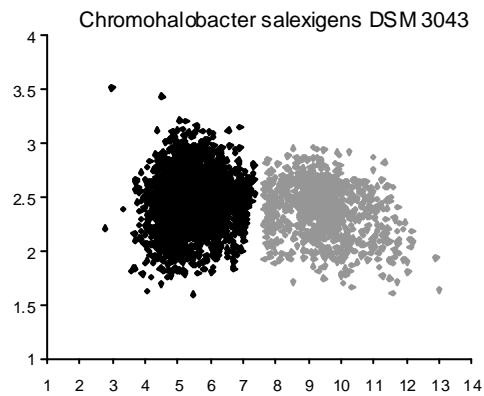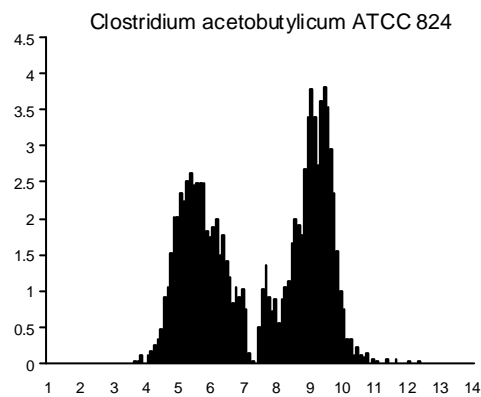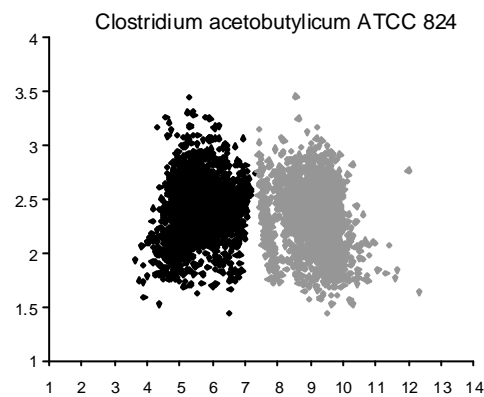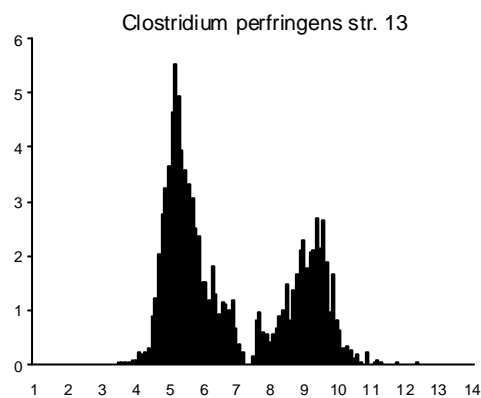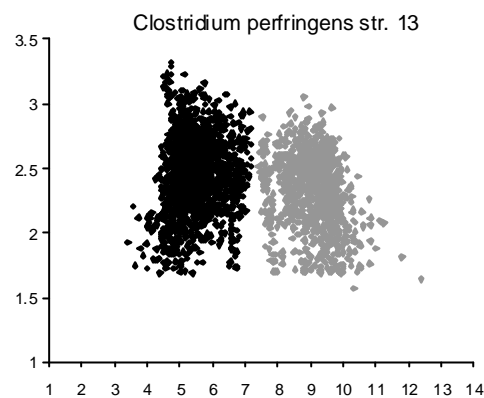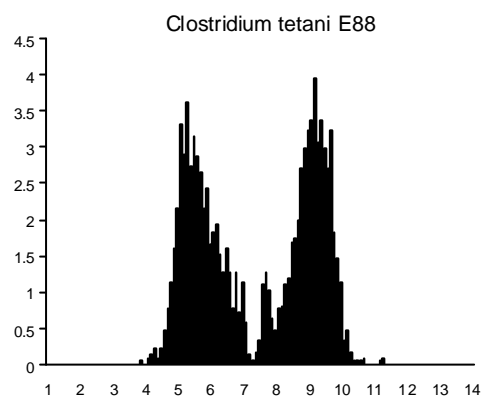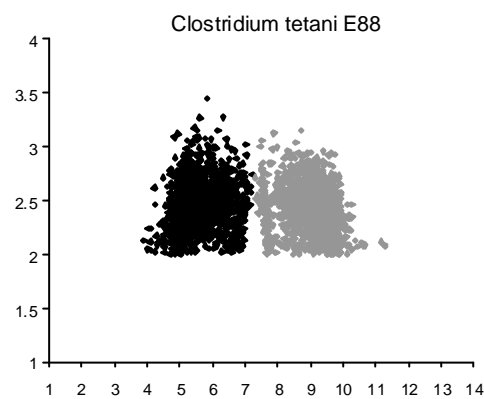

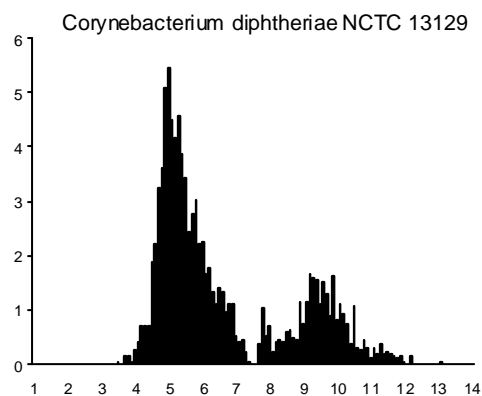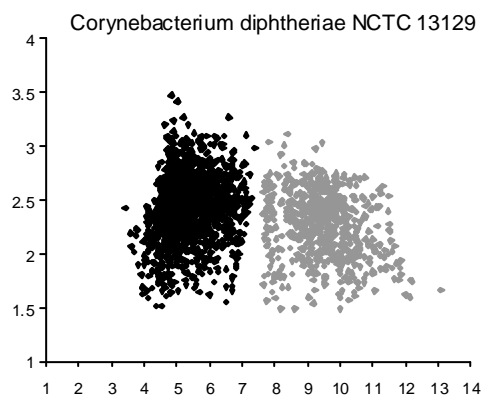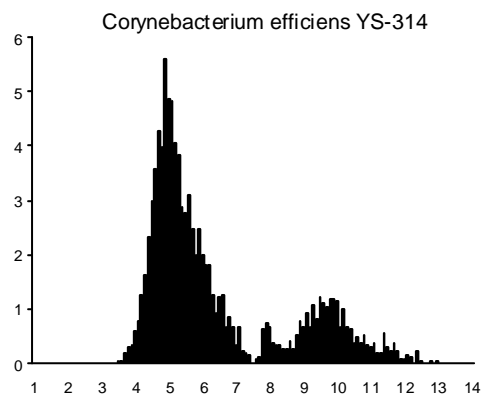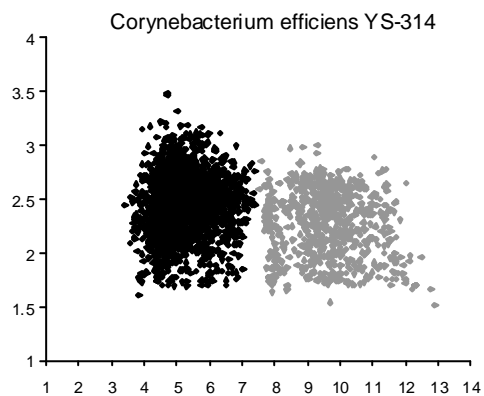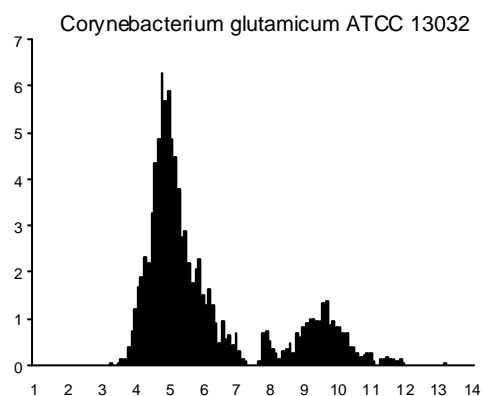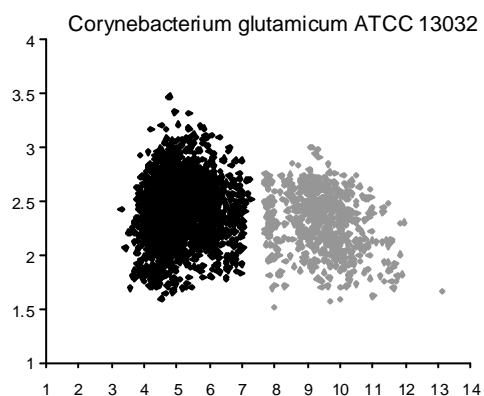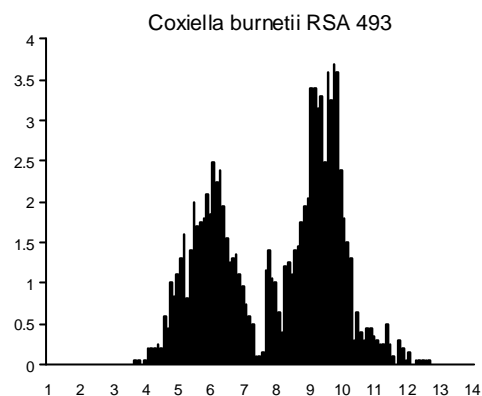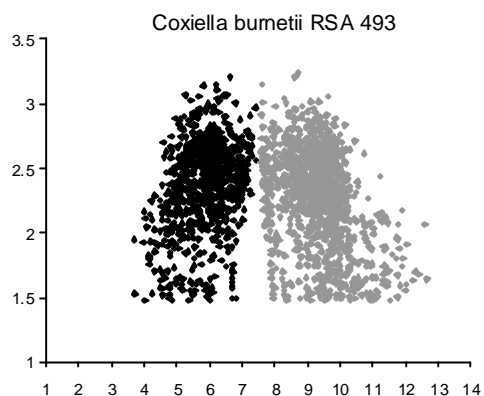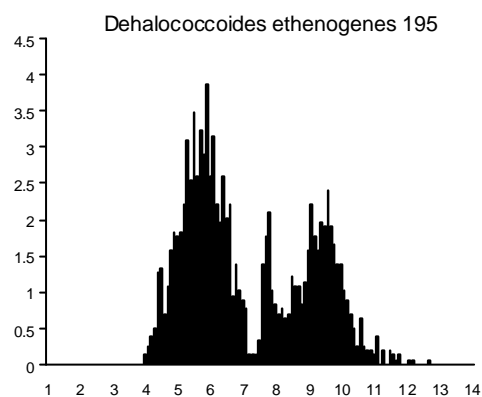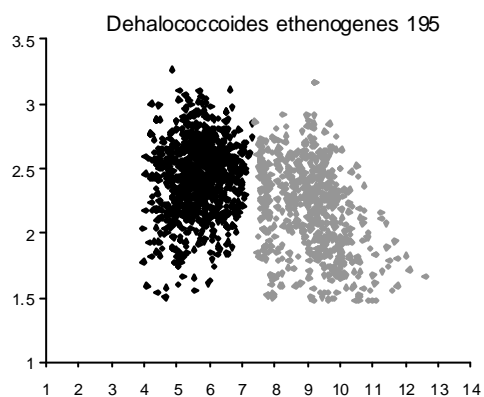

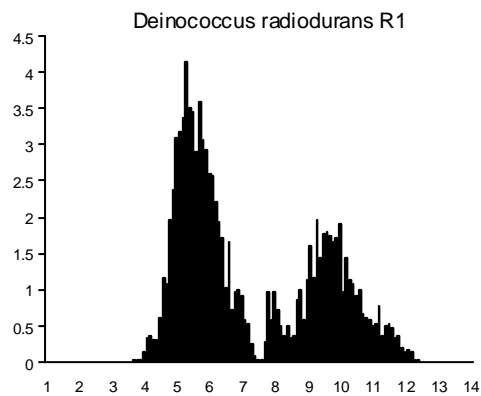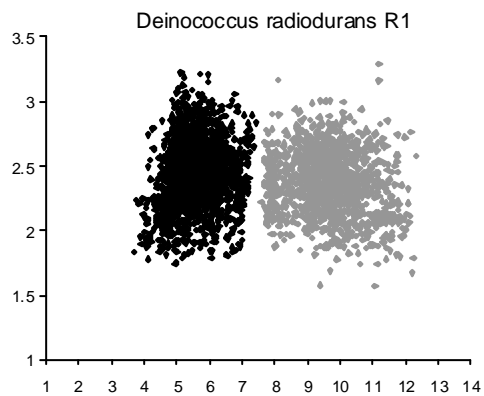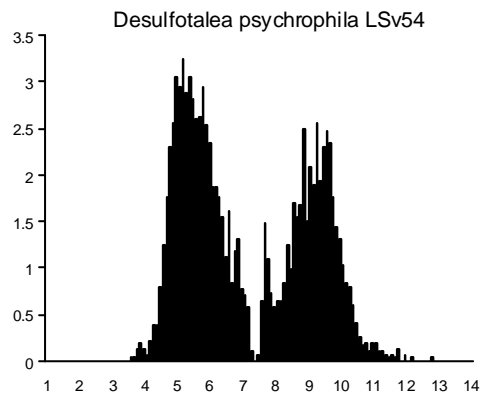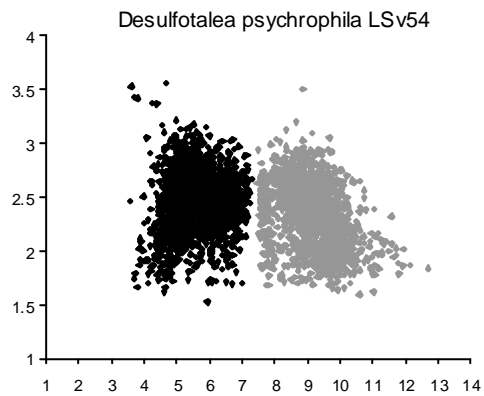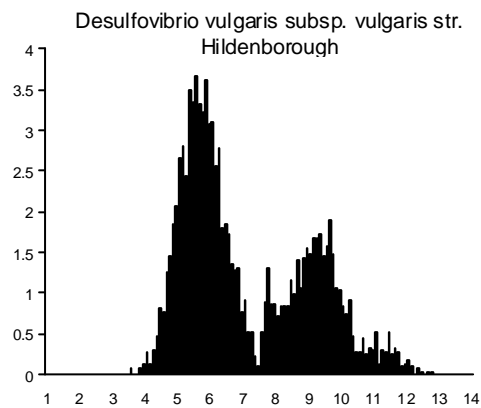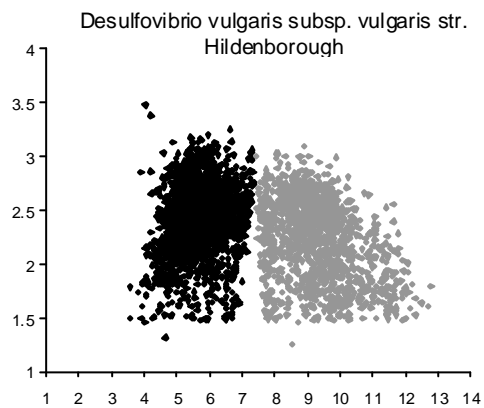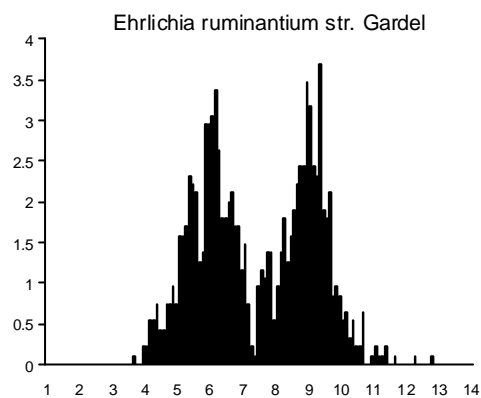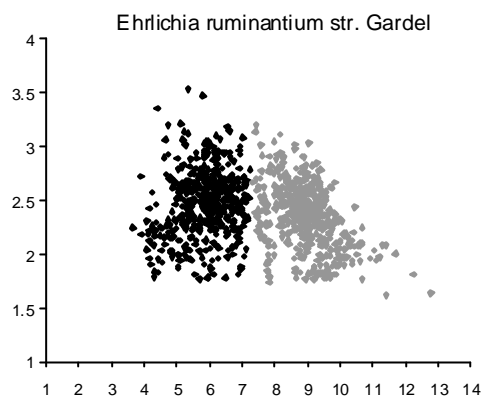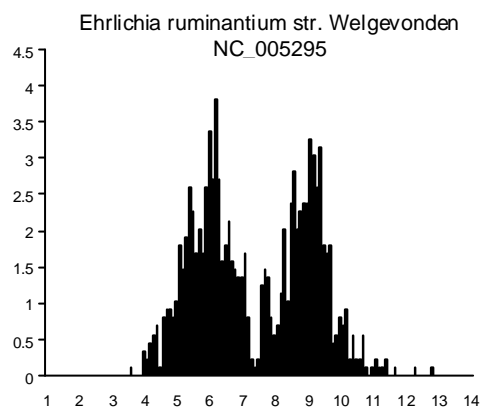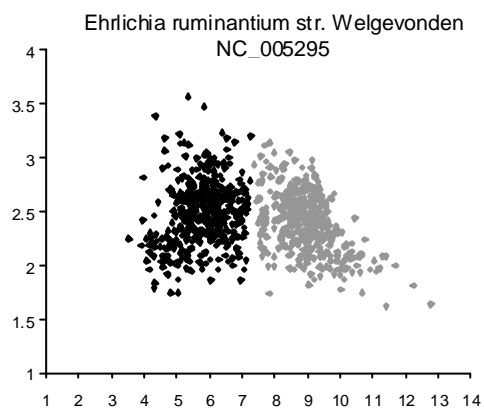

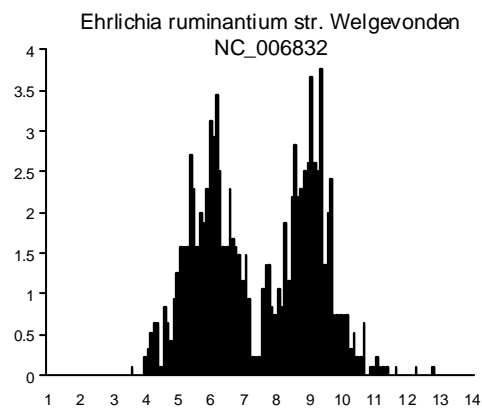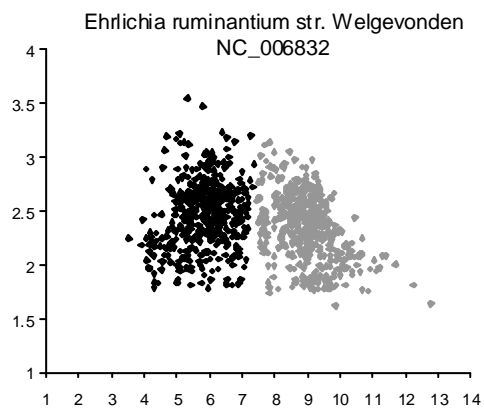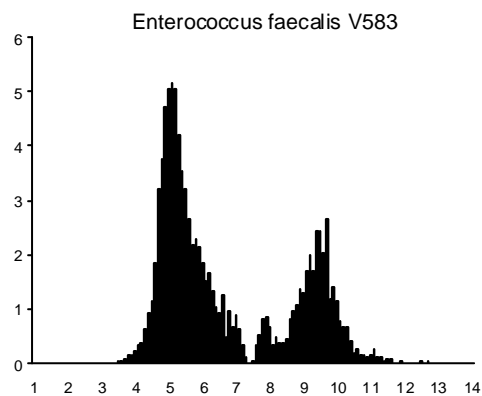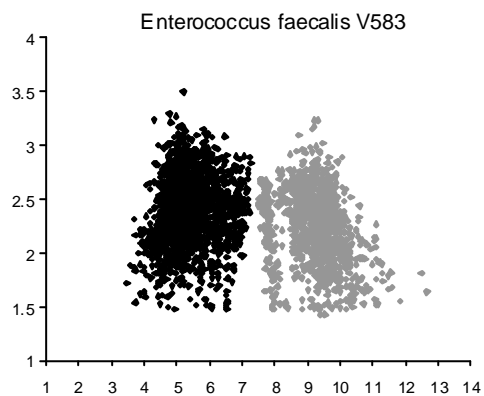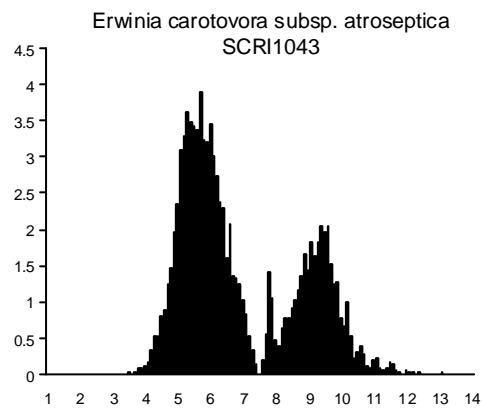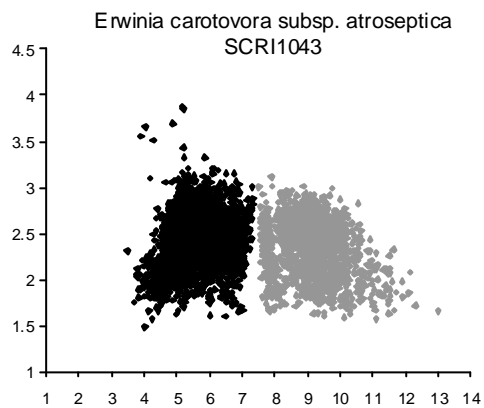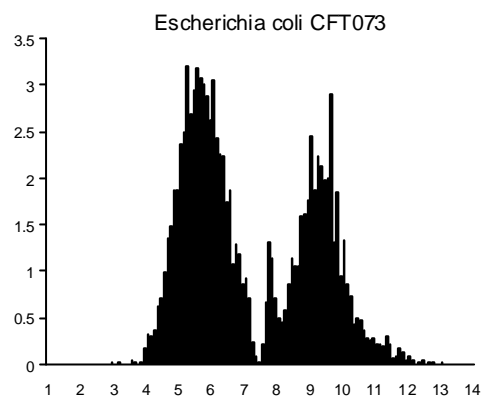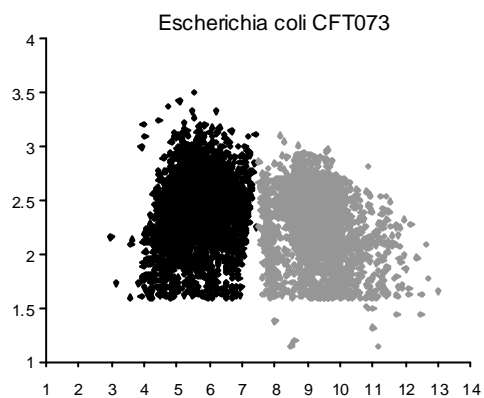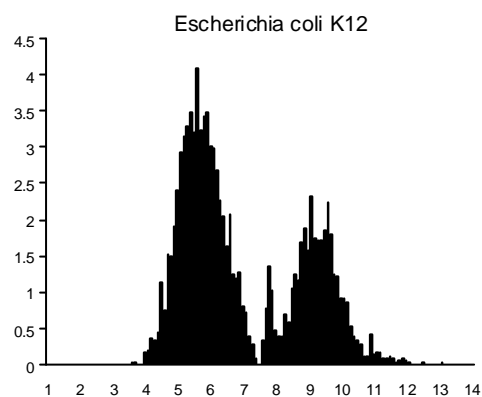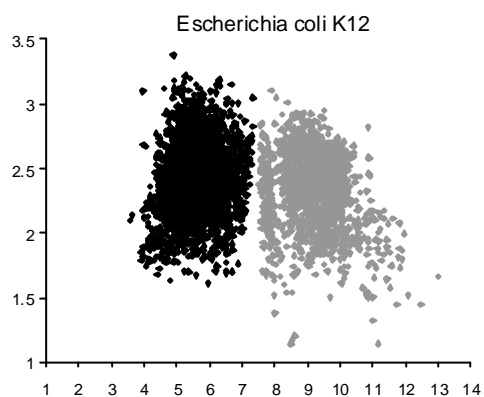

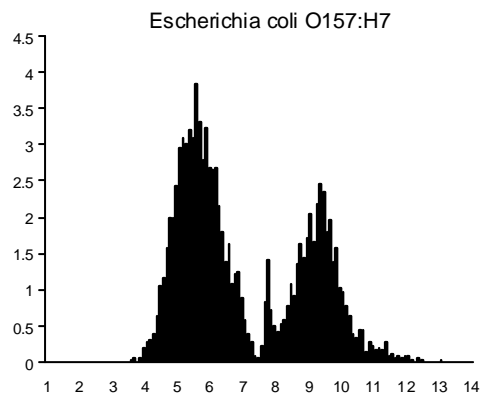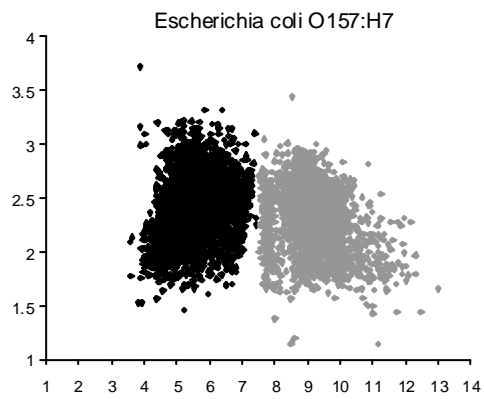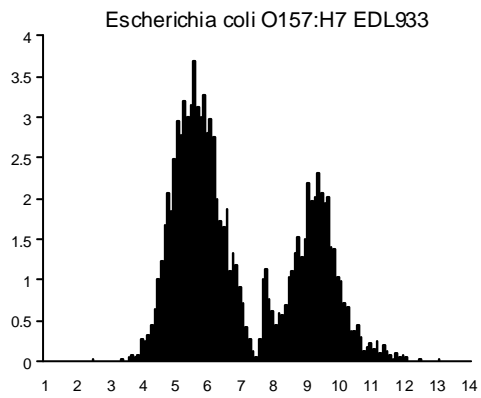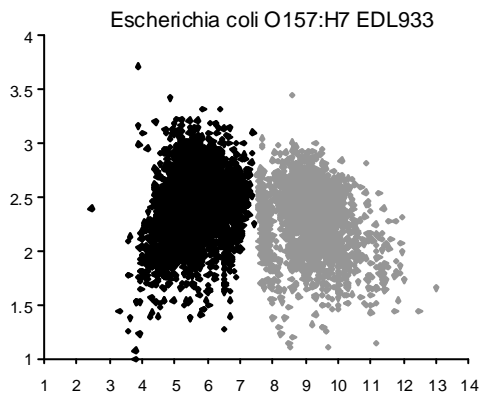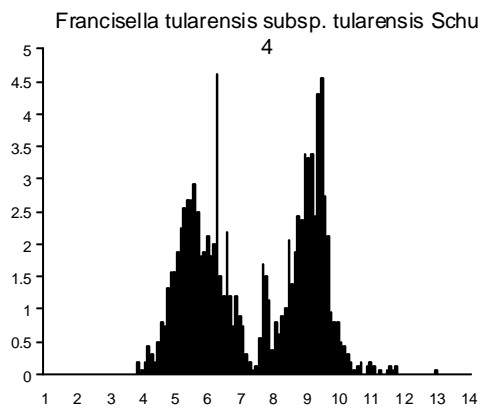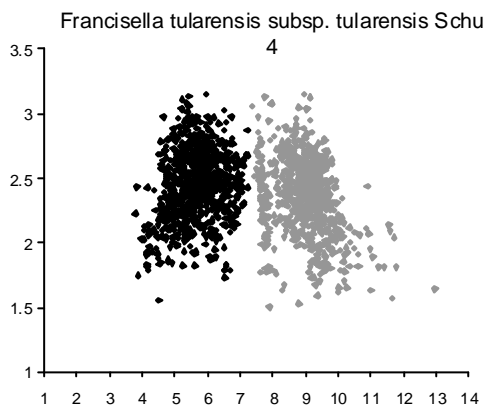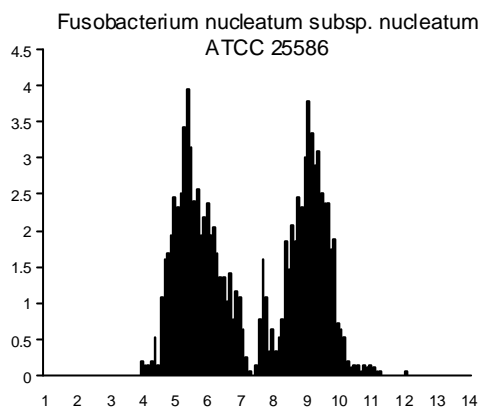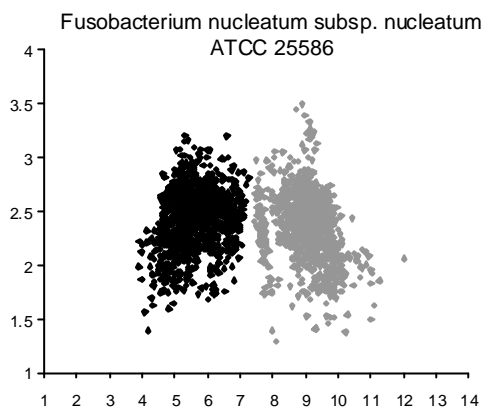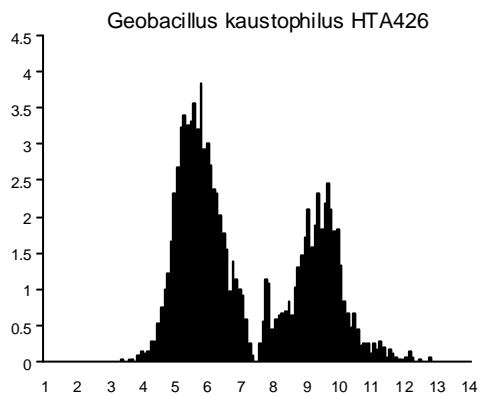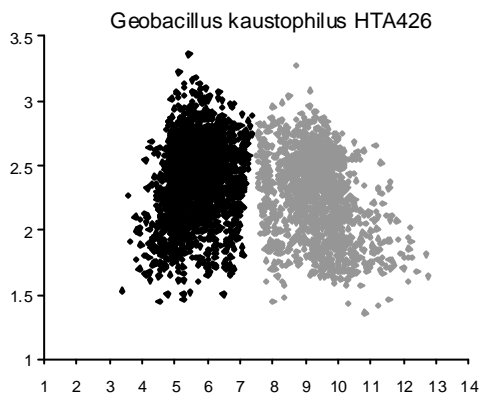

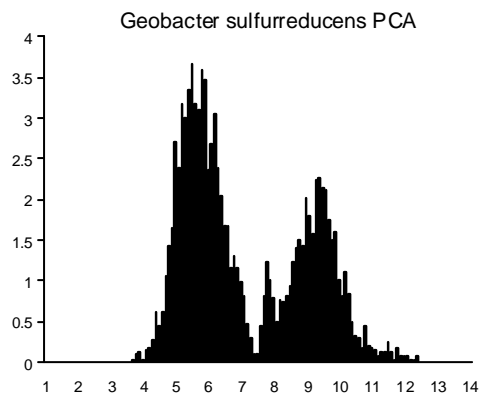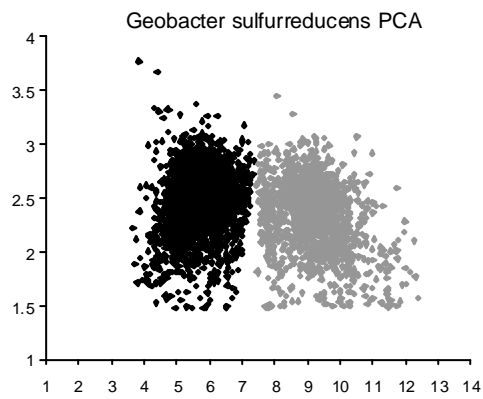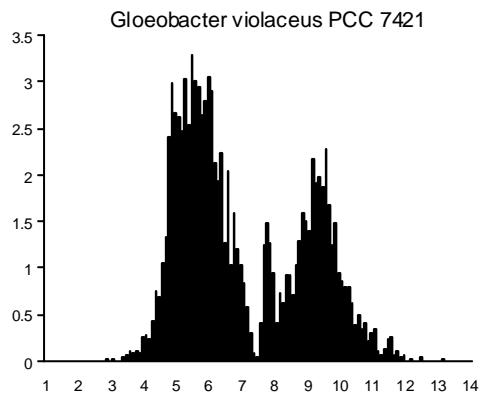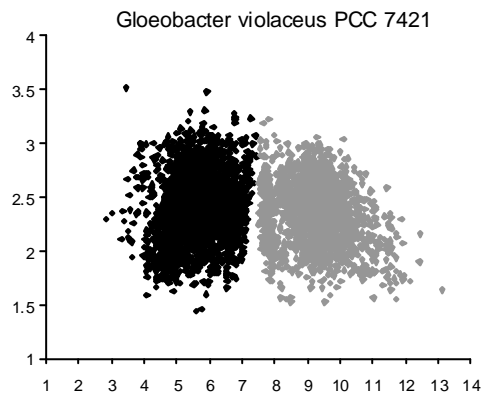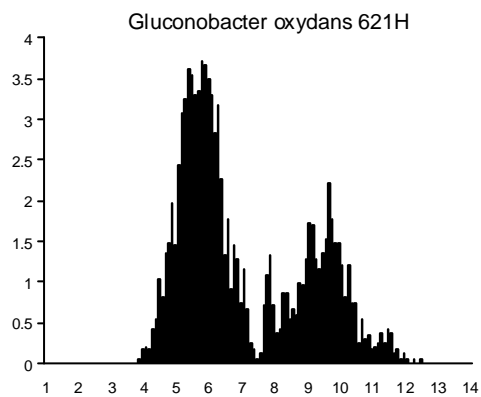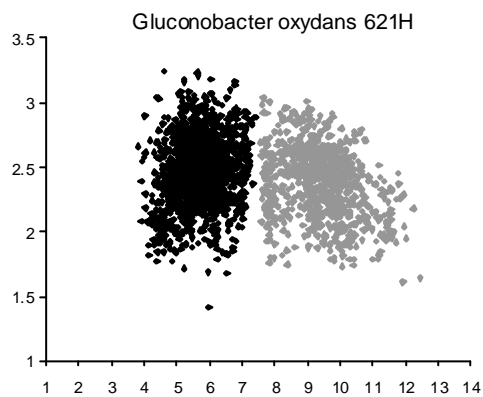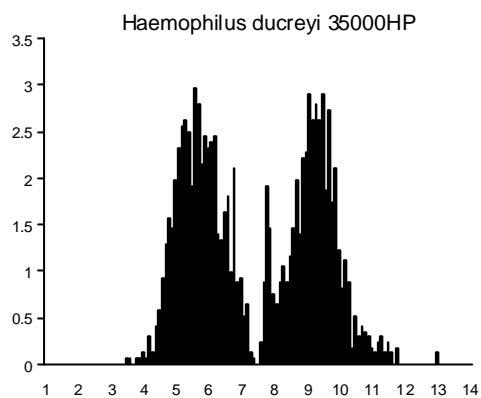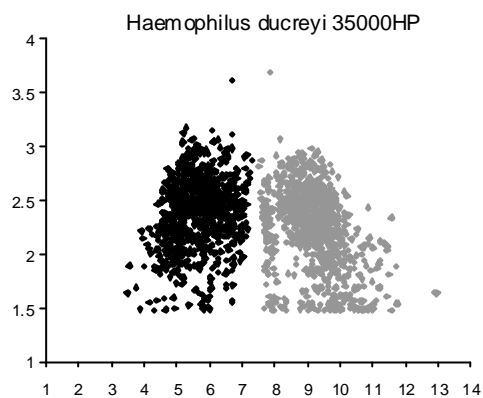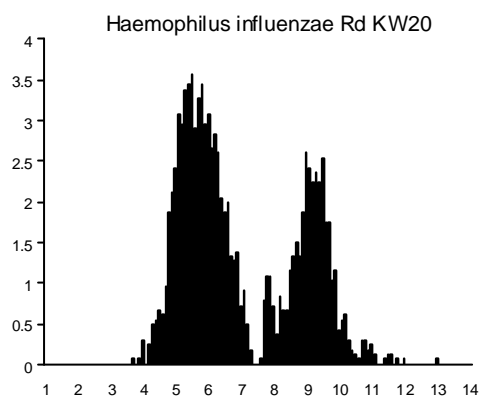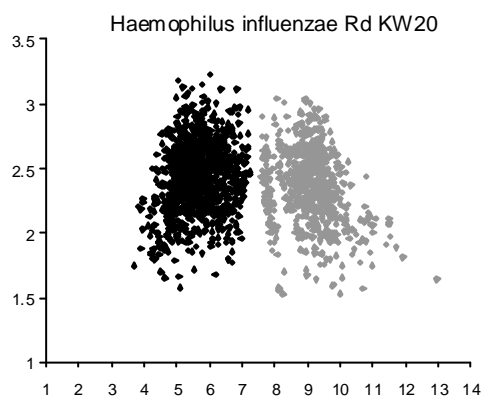

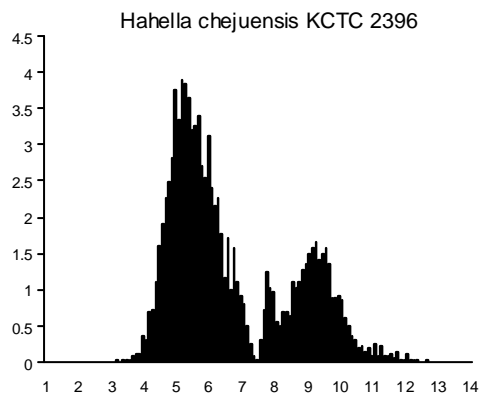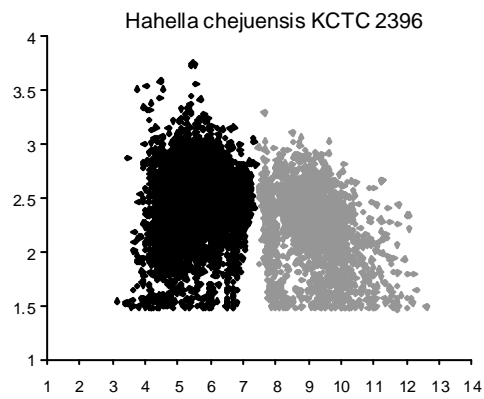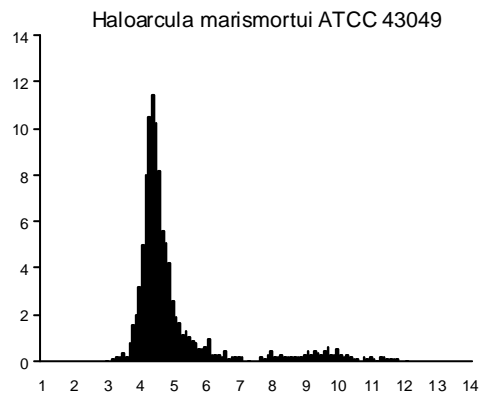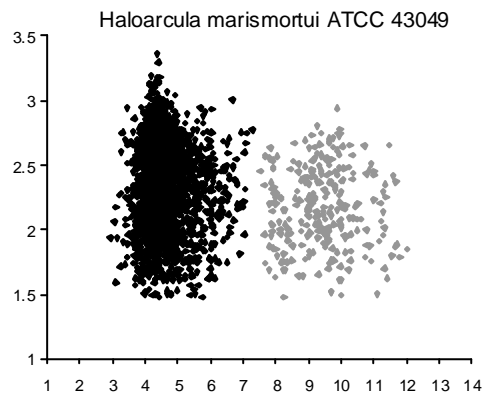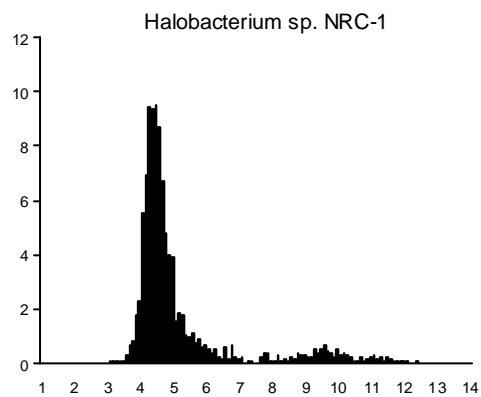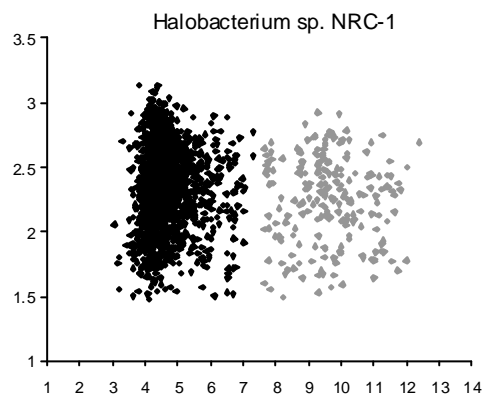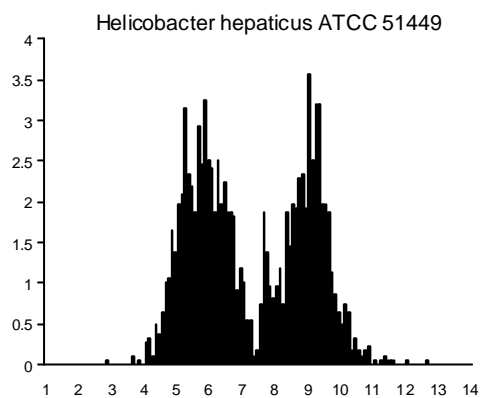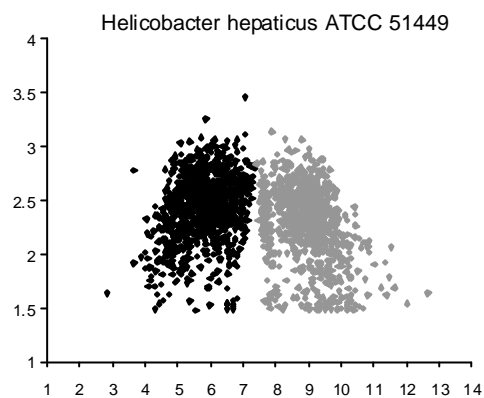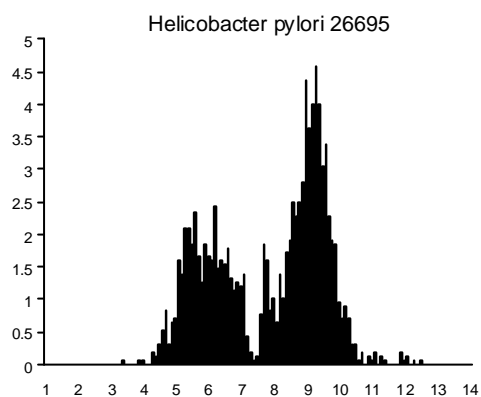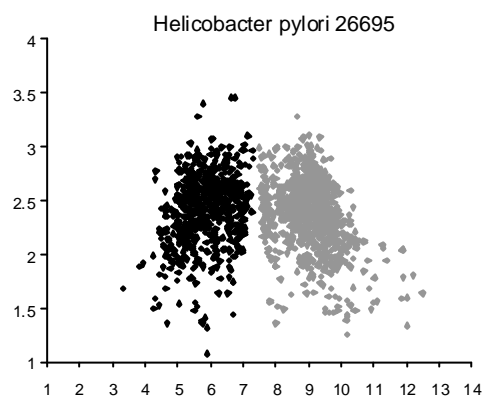

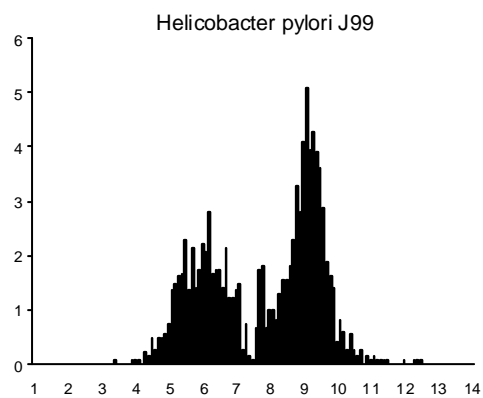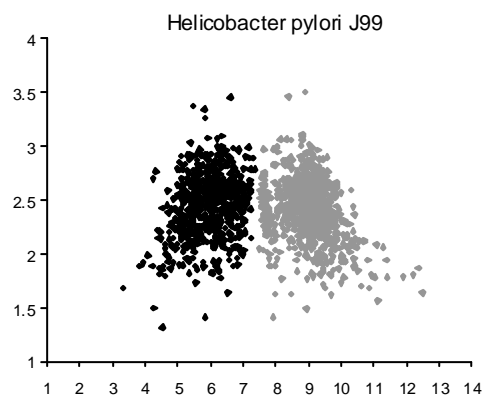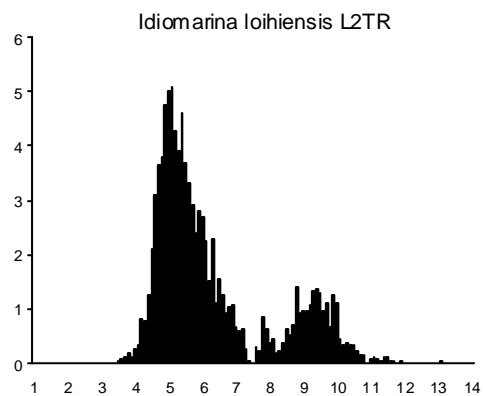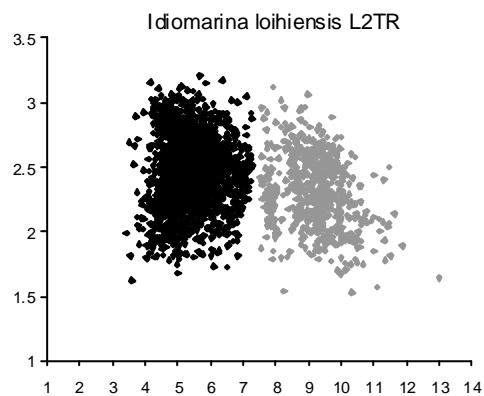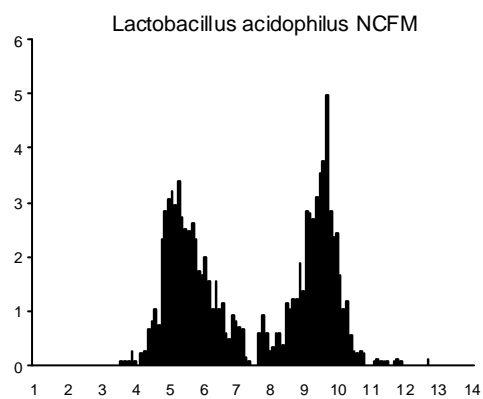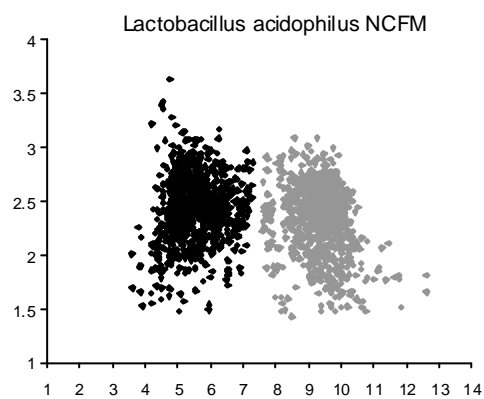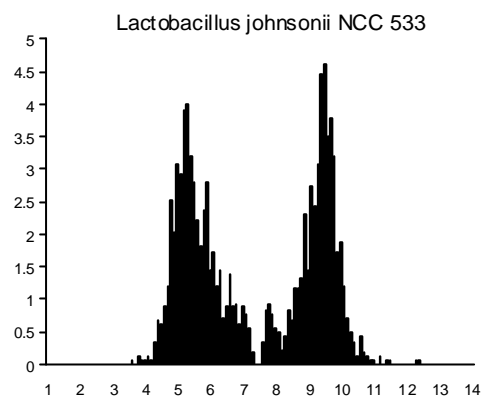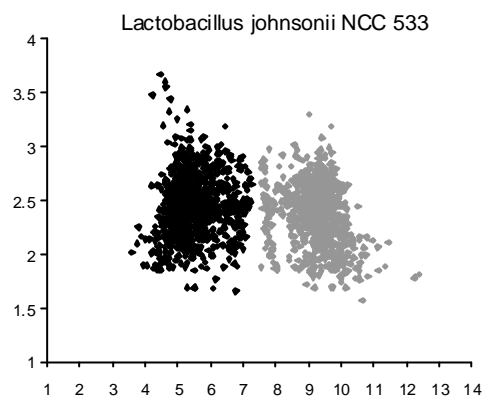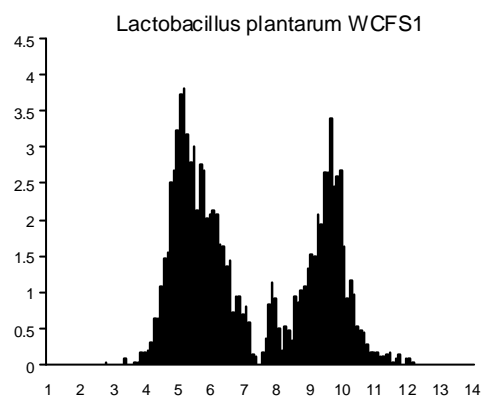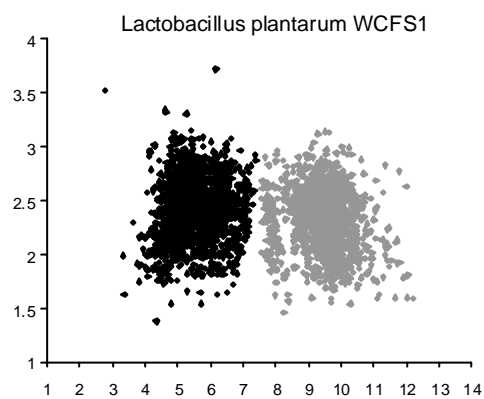

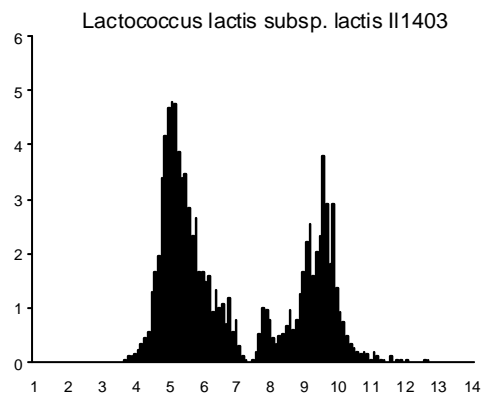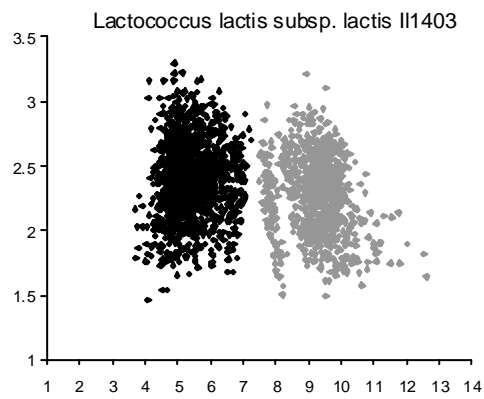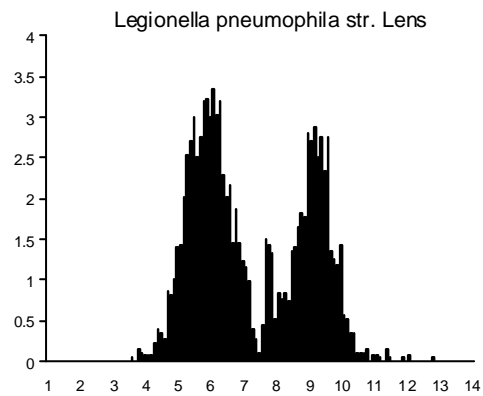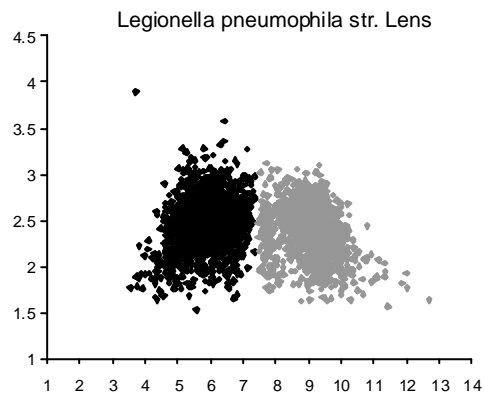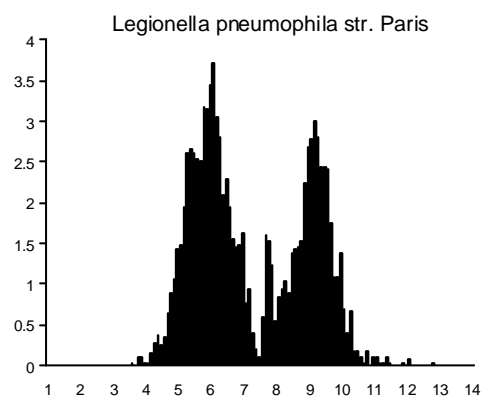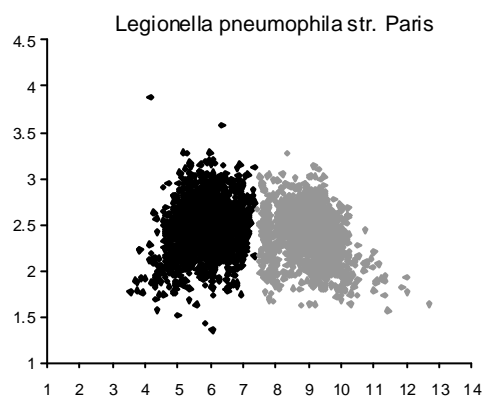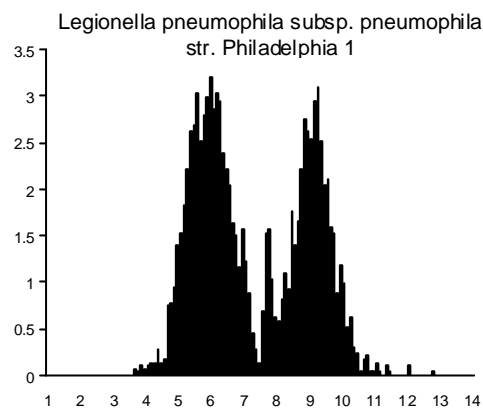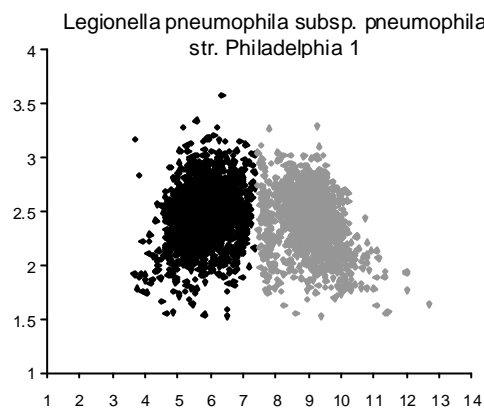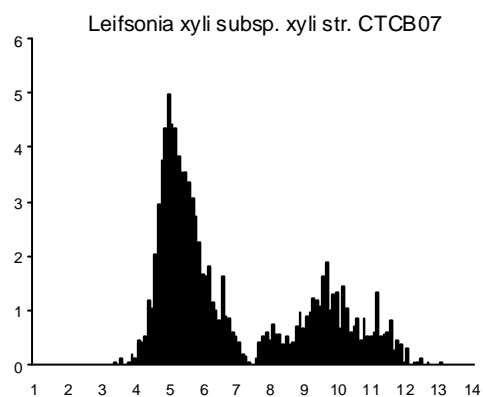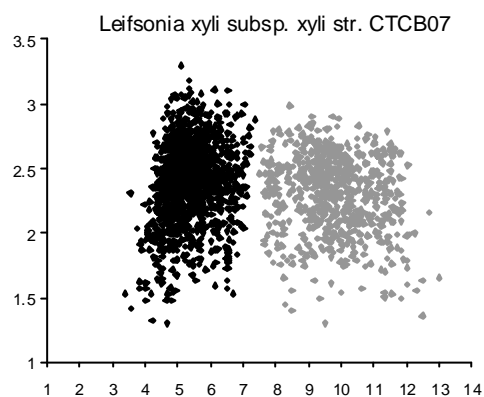

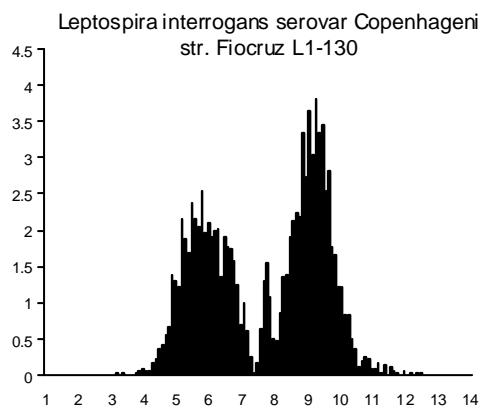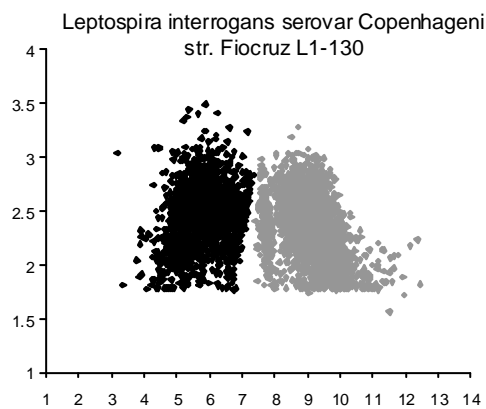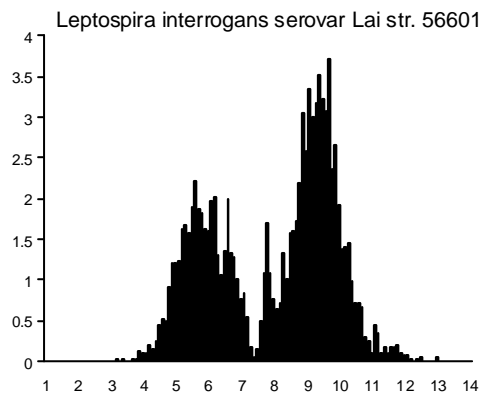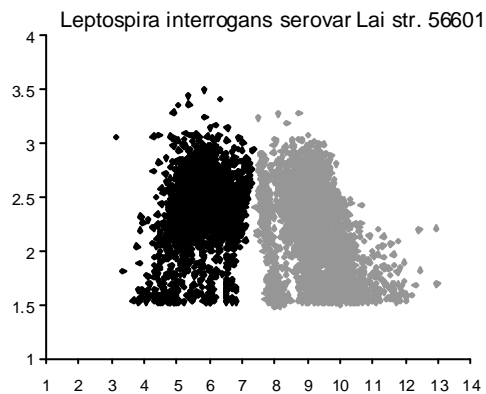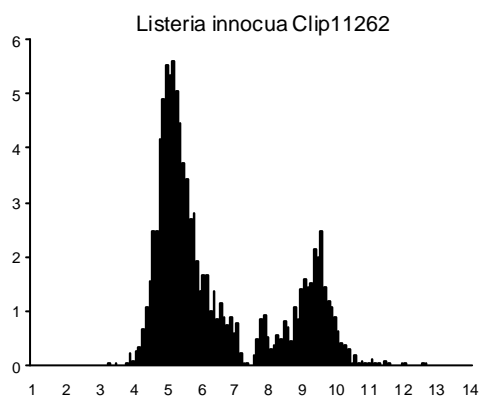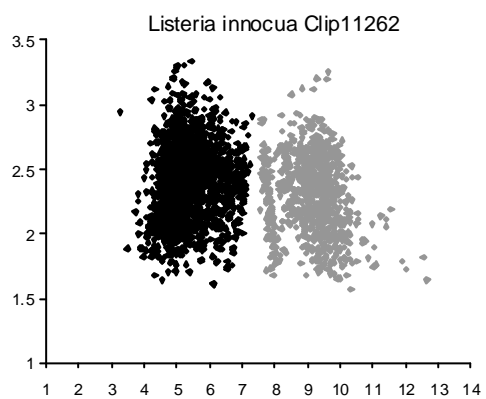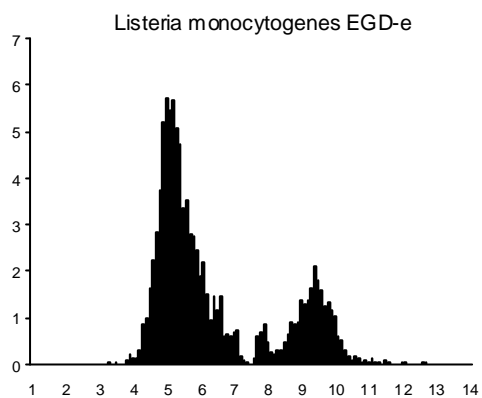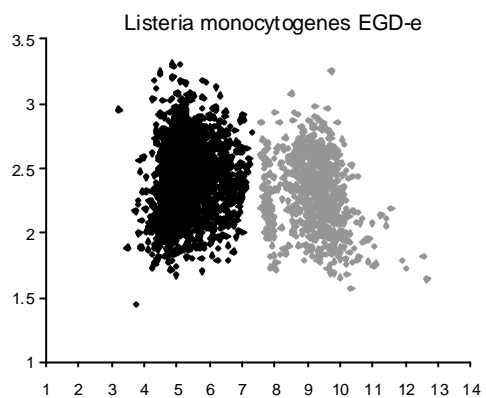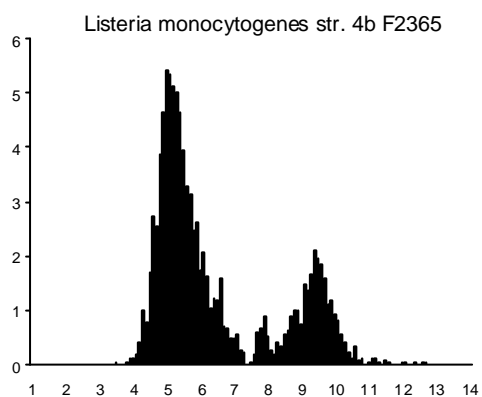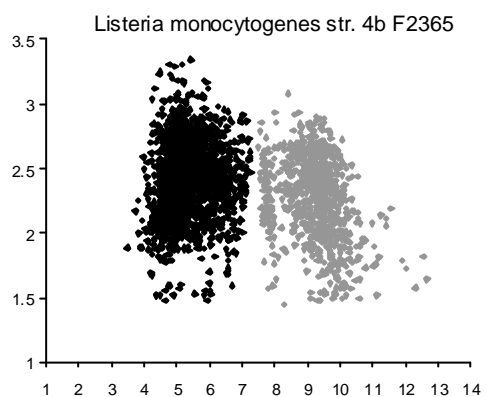

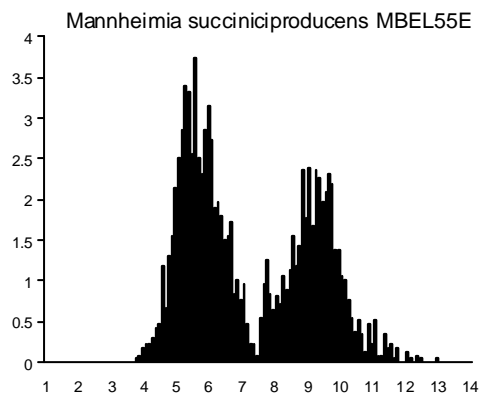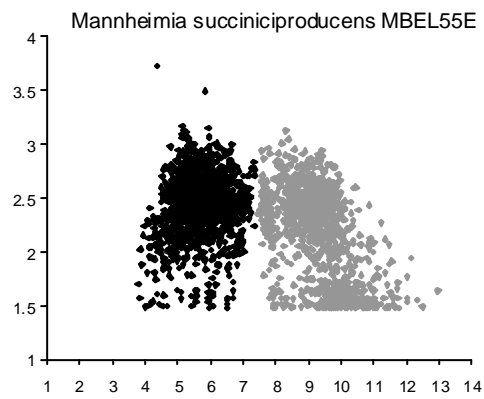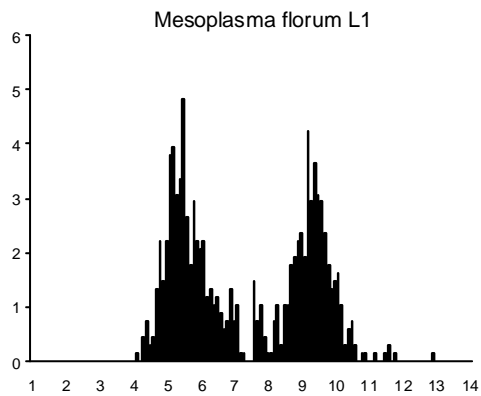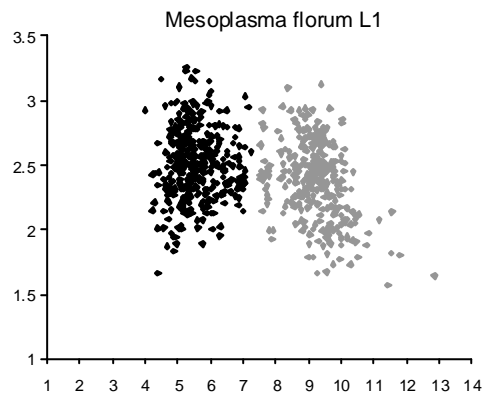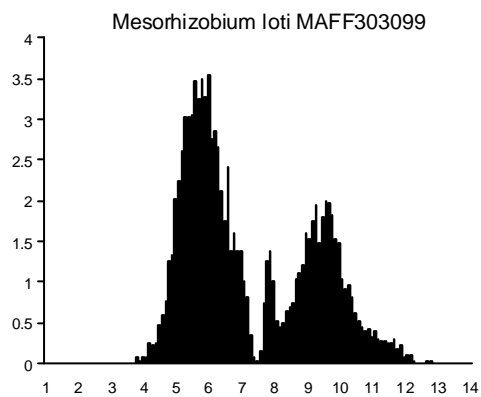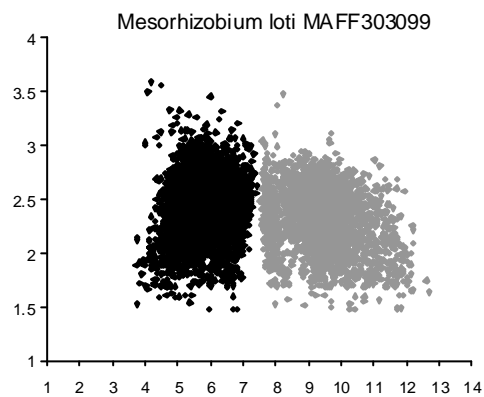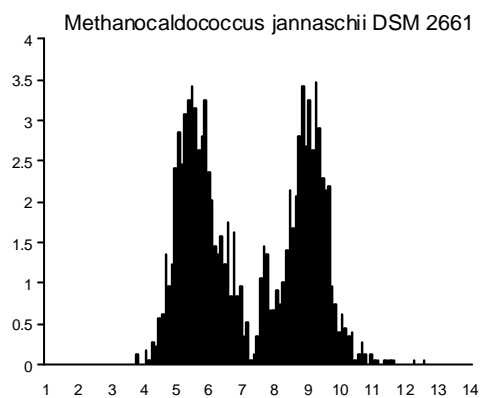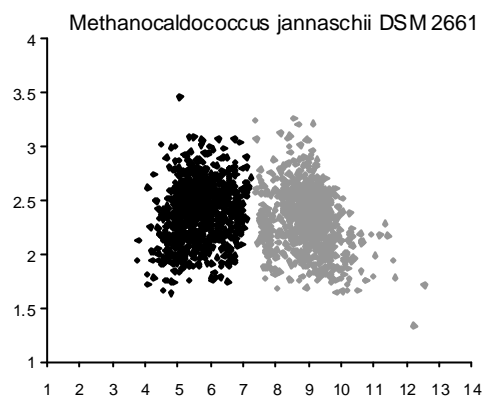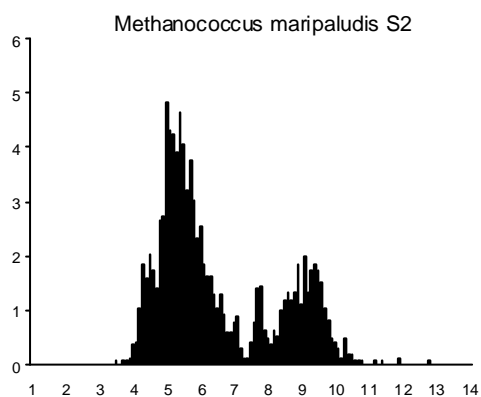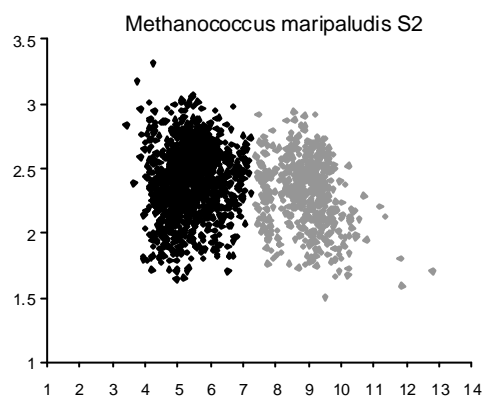

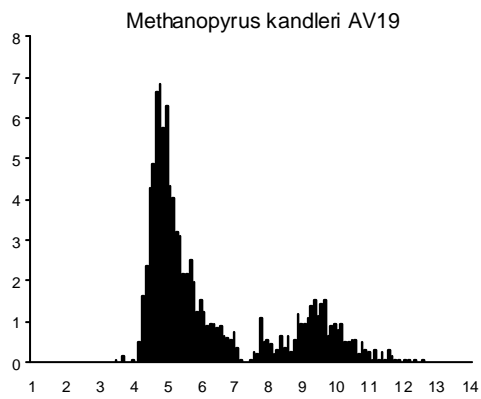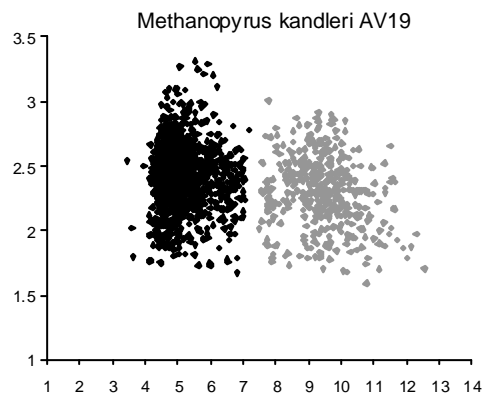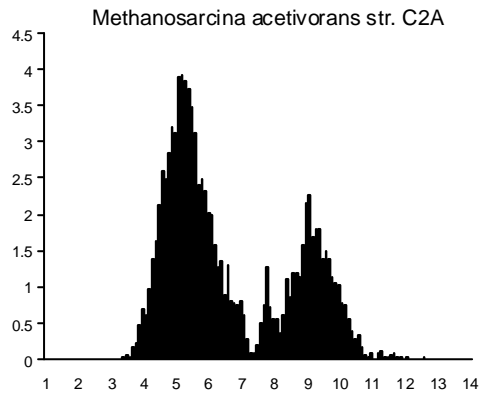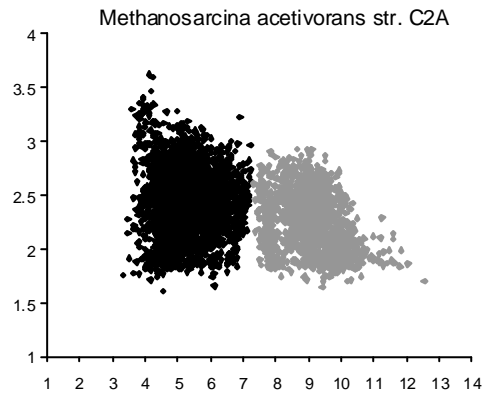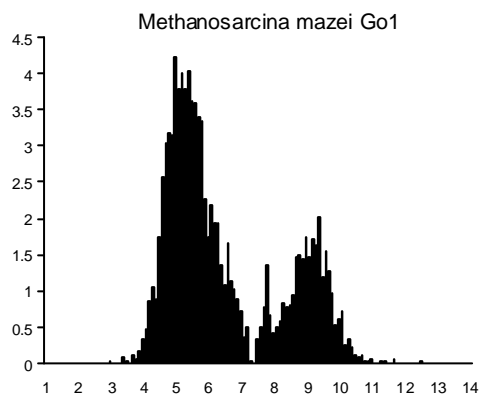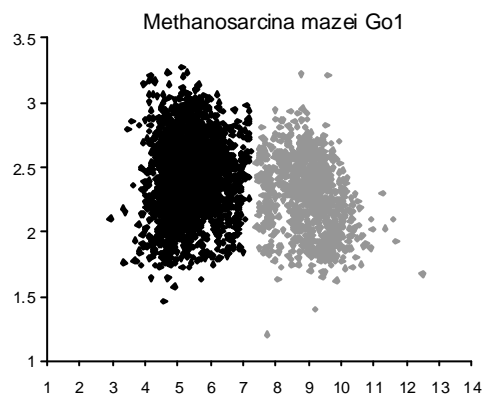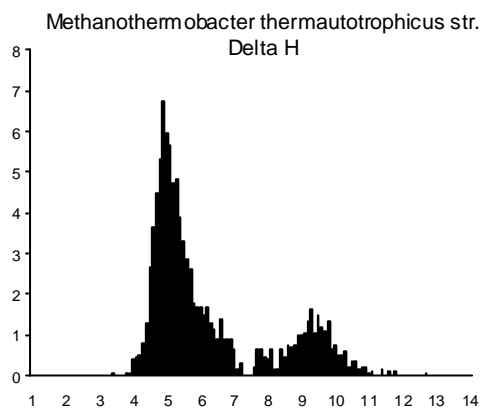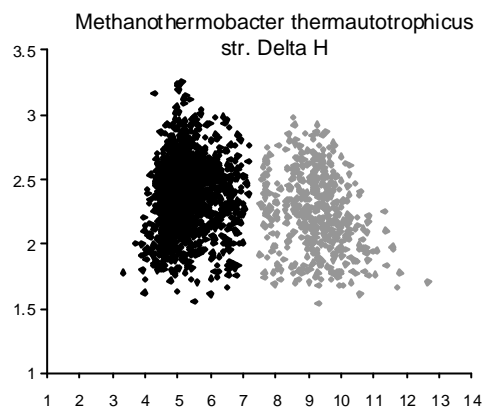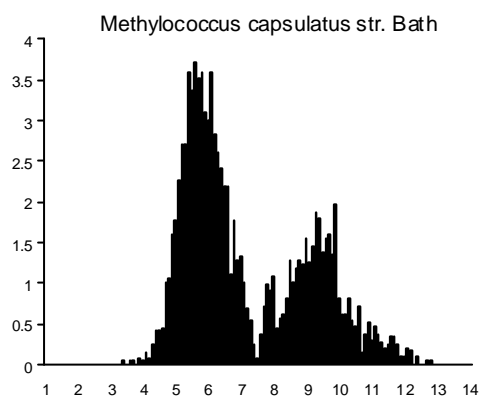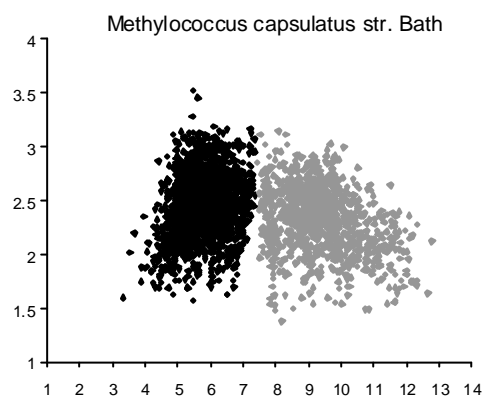

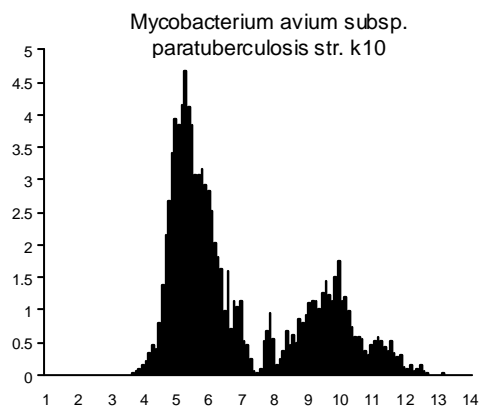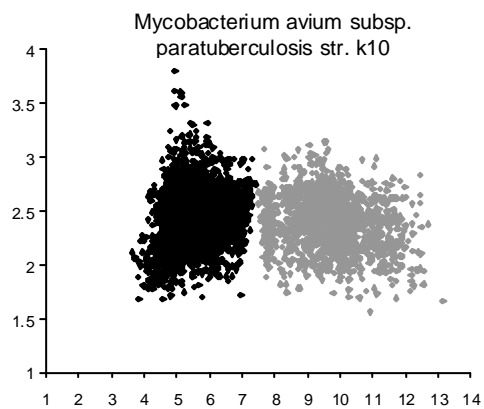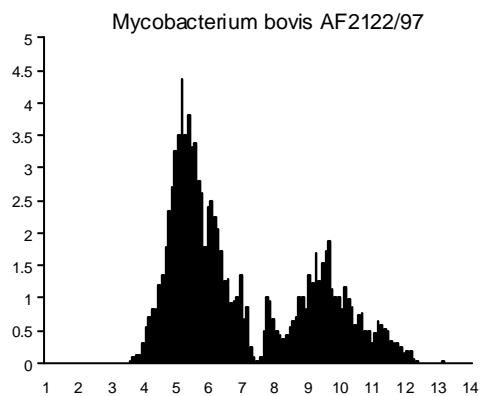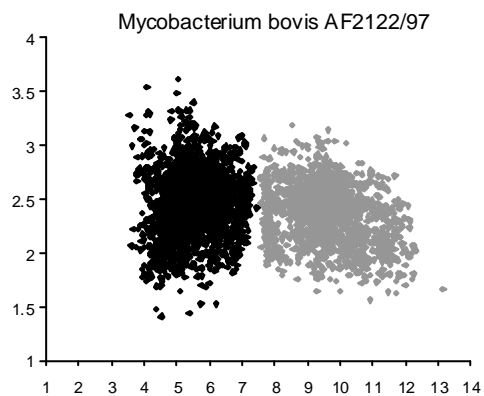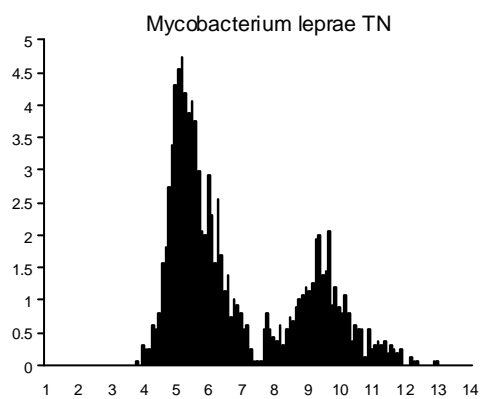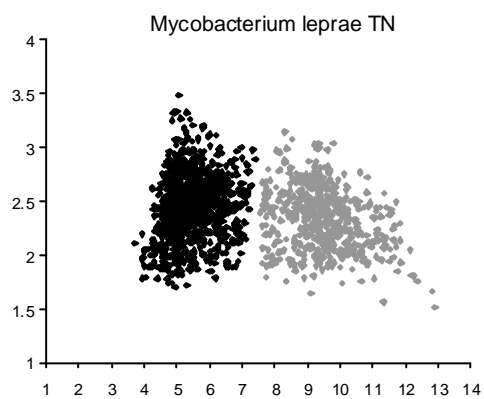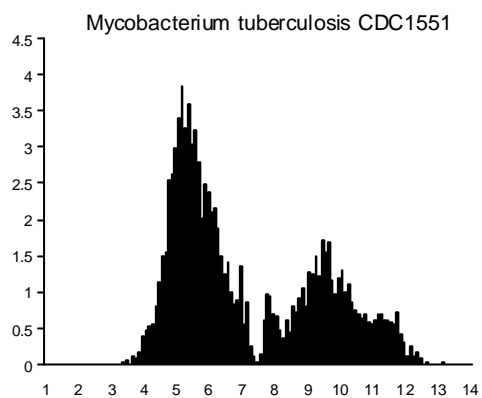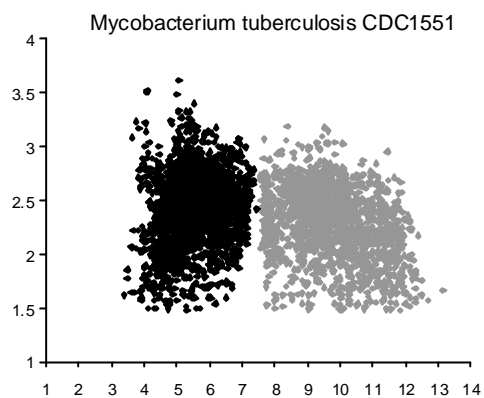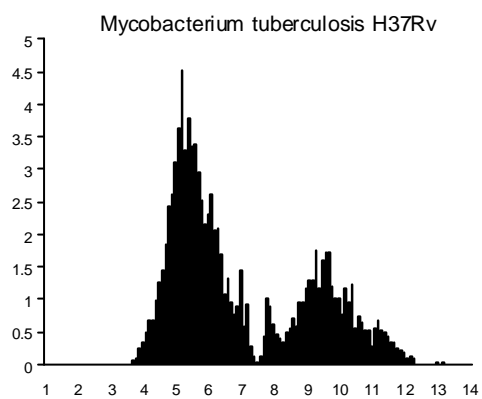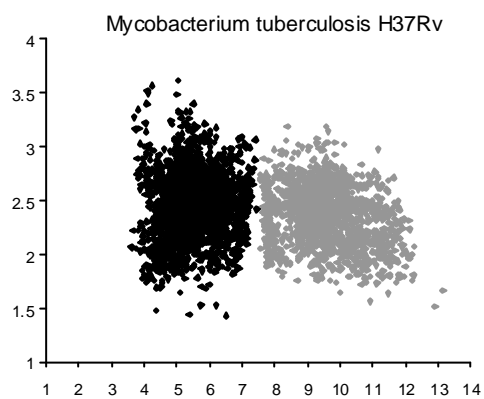

Supplement: Additional file 2 — PI distribution for prokaryotic proteomes. Left panel: histograms of pI values at 0.1 unit intervals (X axis: class of pI; Y axis: percent); right panel: relationships between the logarithm of length of proteins (Y axis) and their pI (Y axis). Black points represent the set of acidic proteins while grey ones – the set of basic proteins. [file 1471-2164-8-163-S2.pdf]

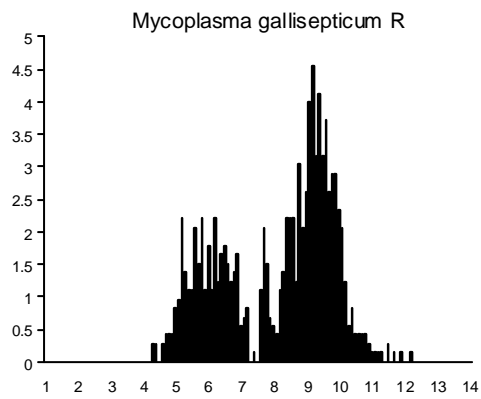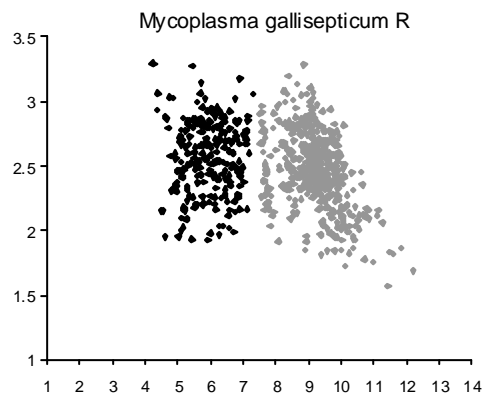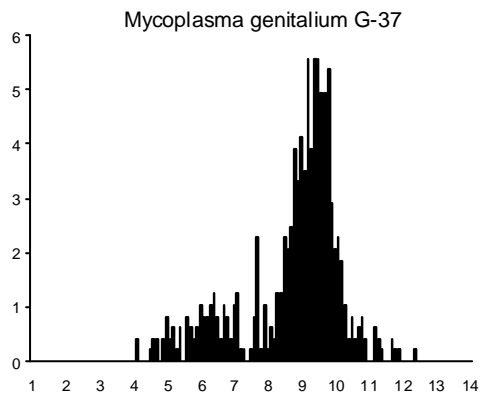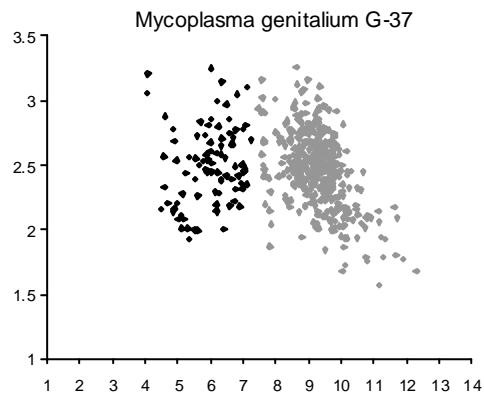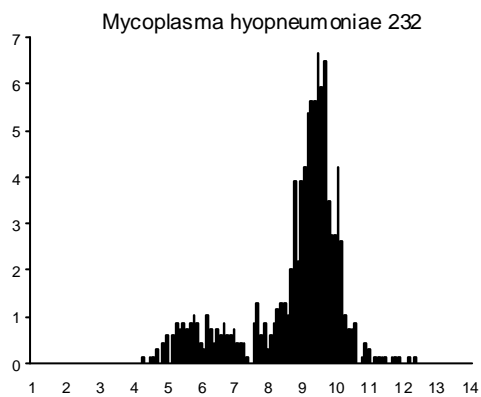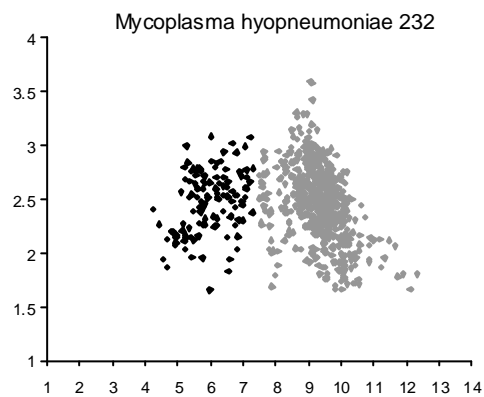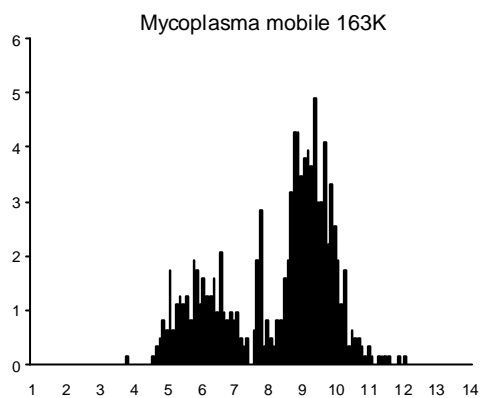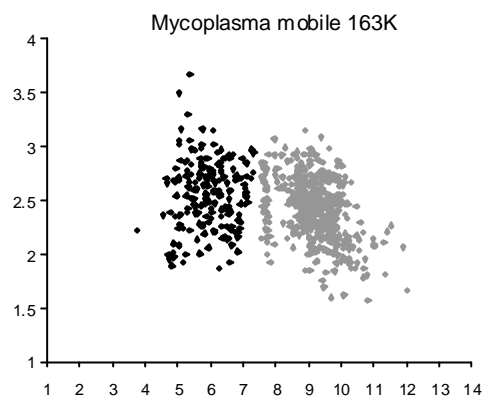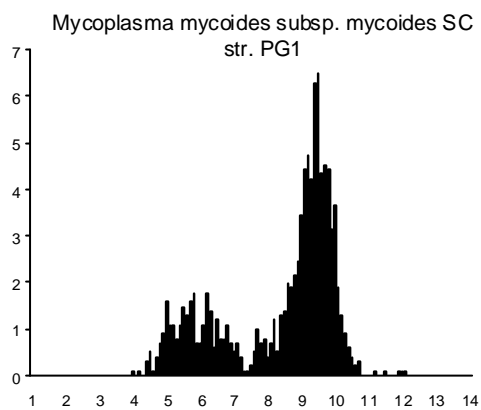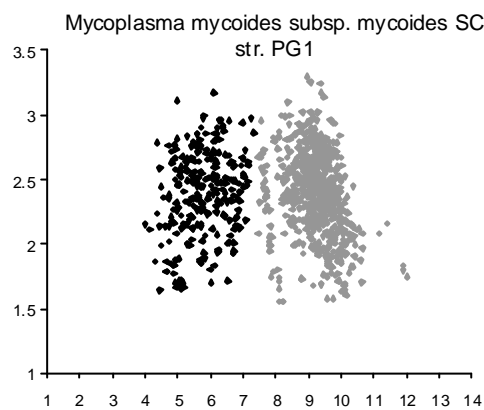

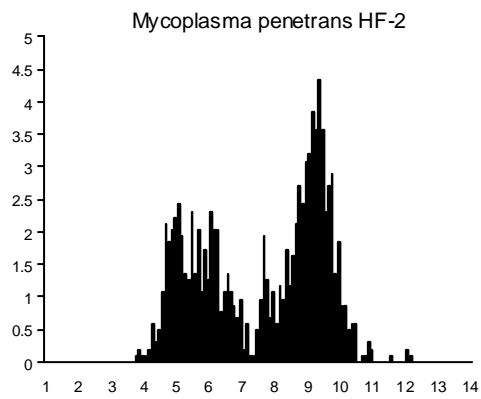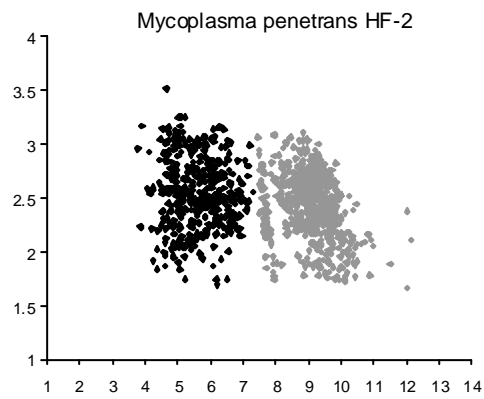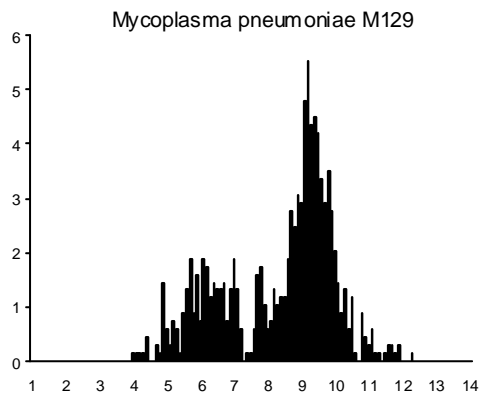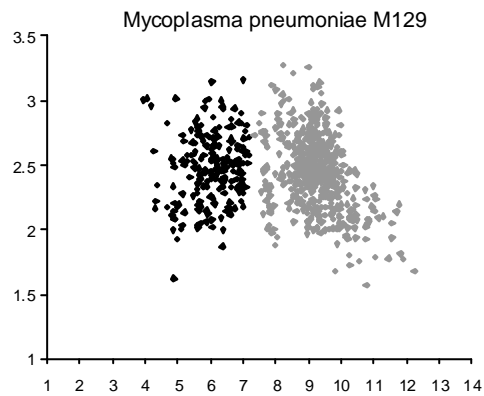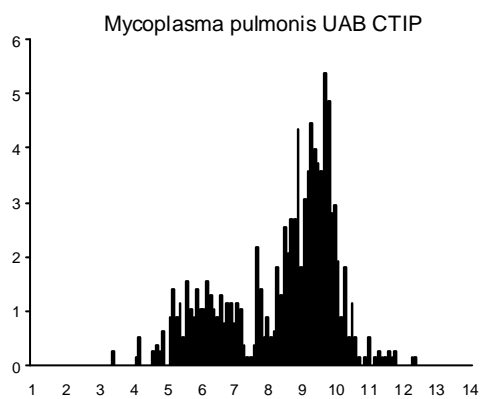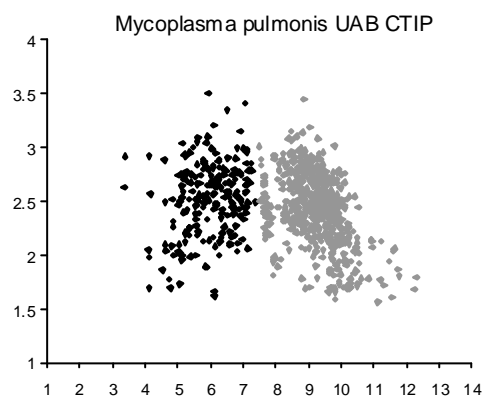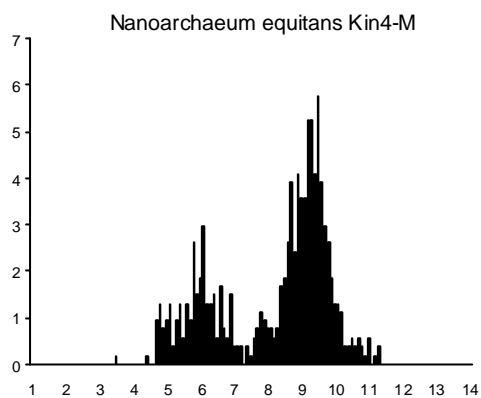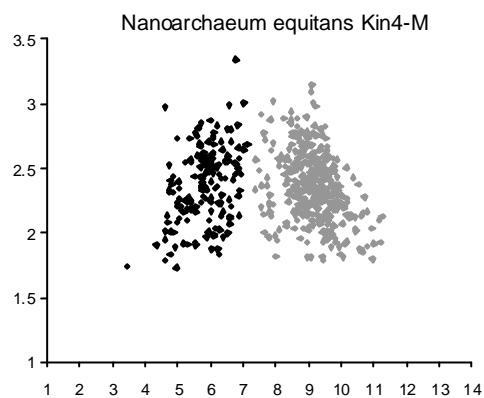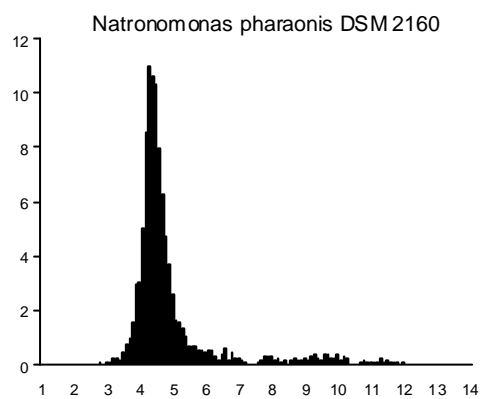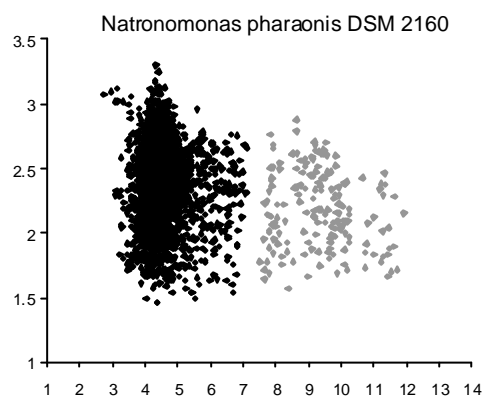

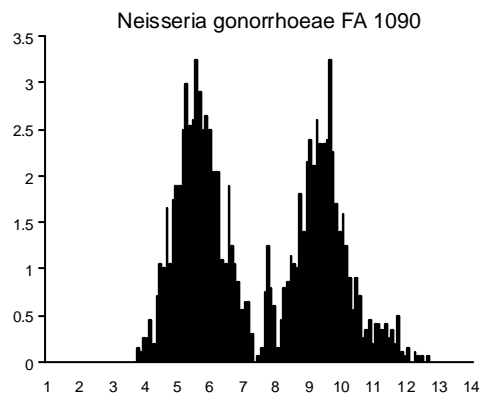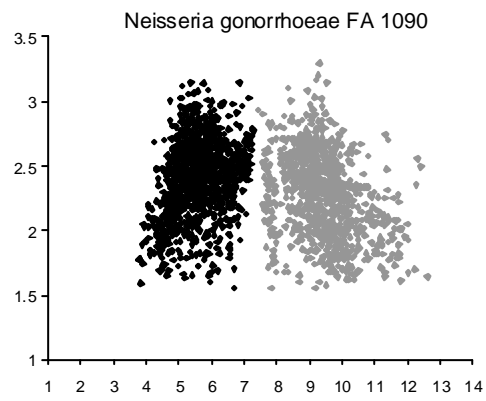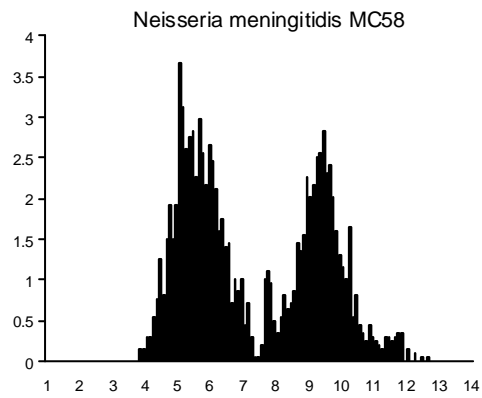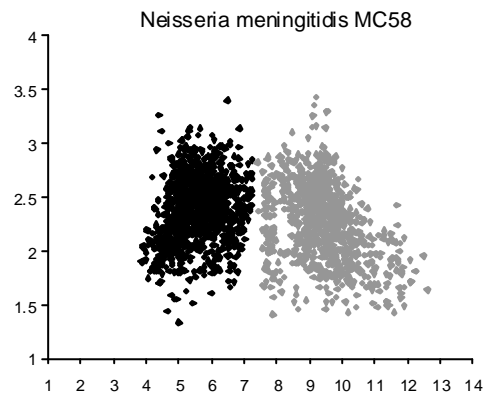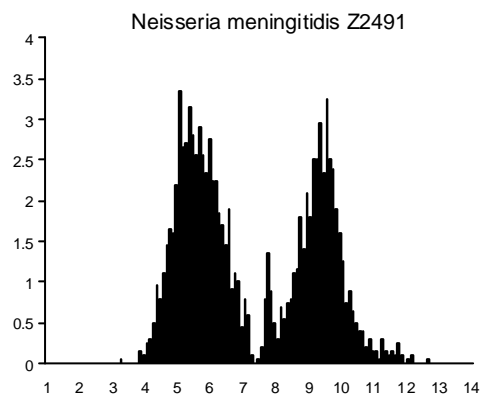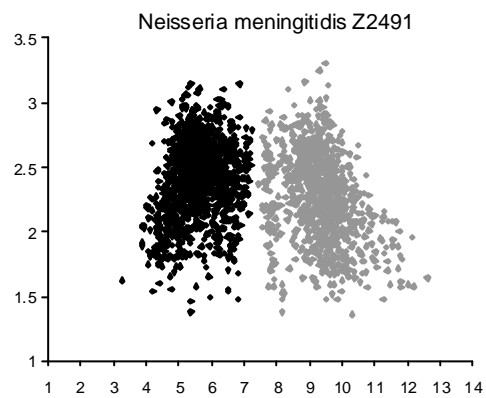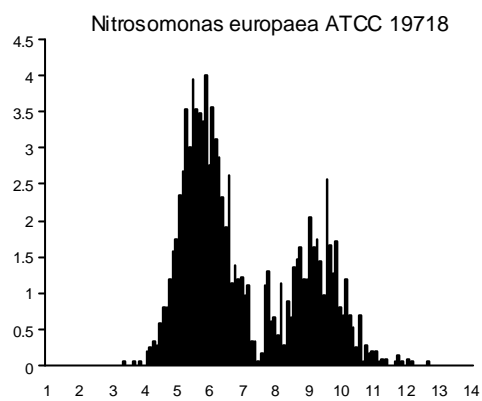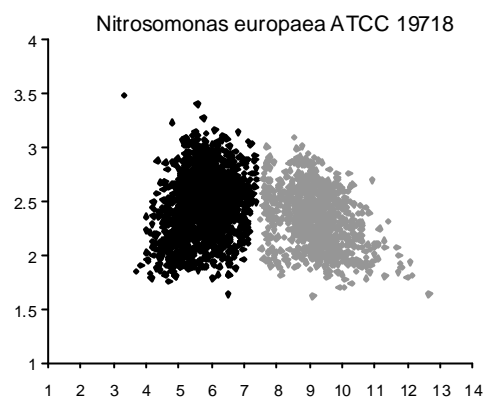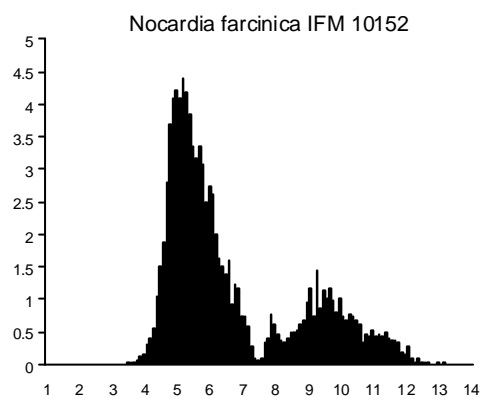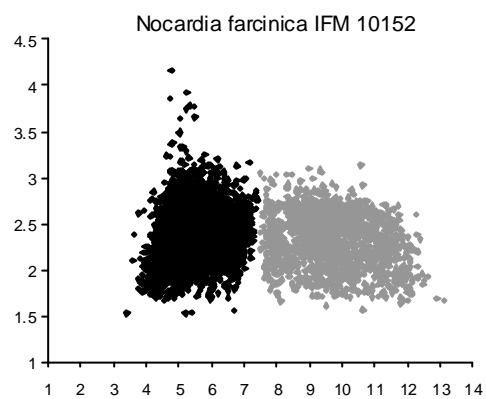

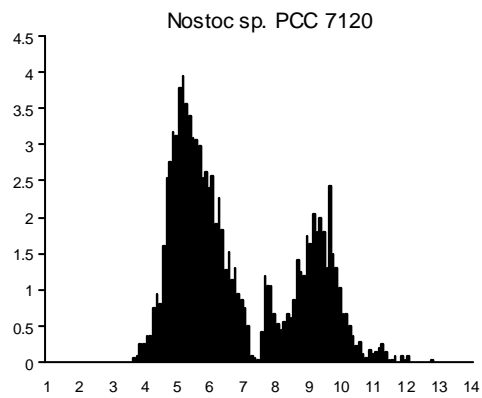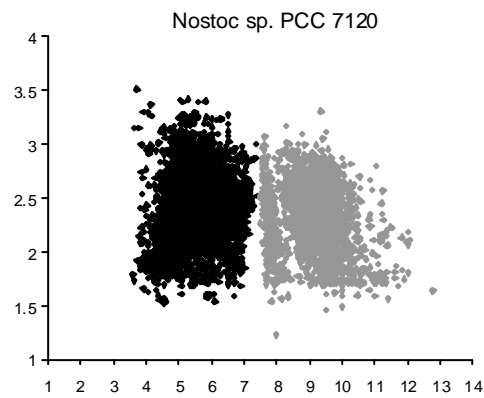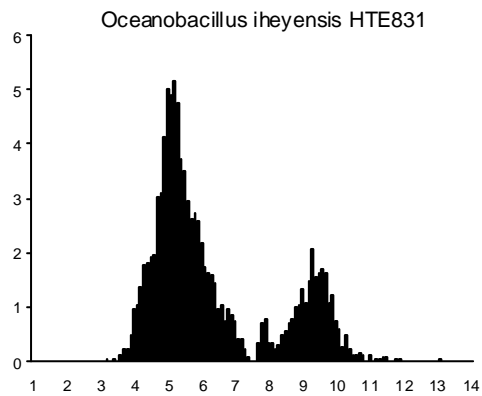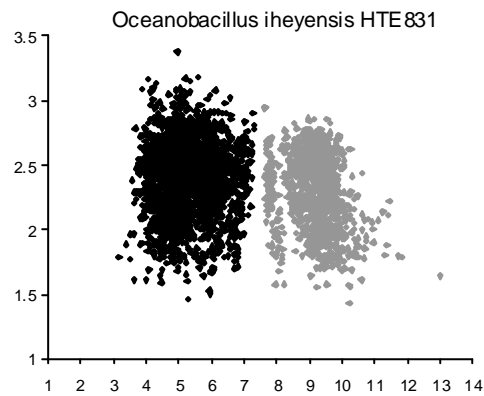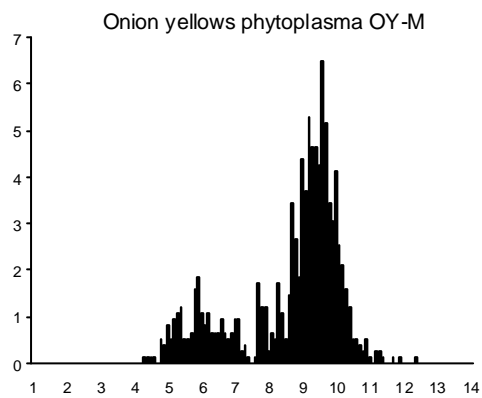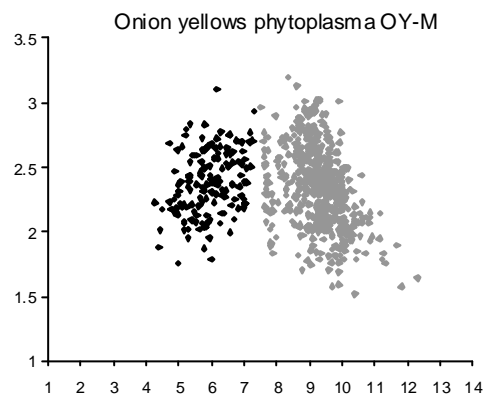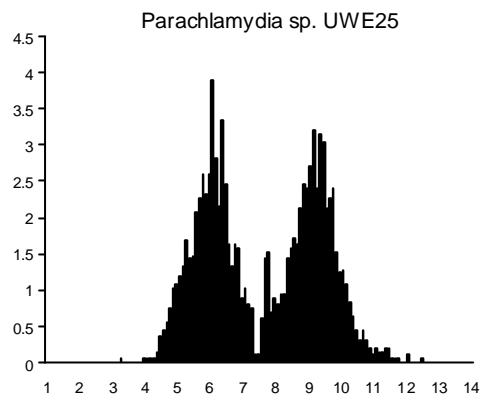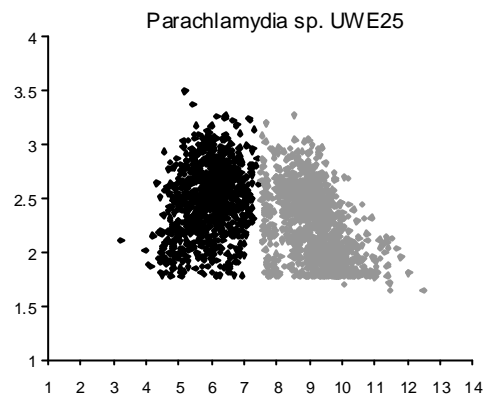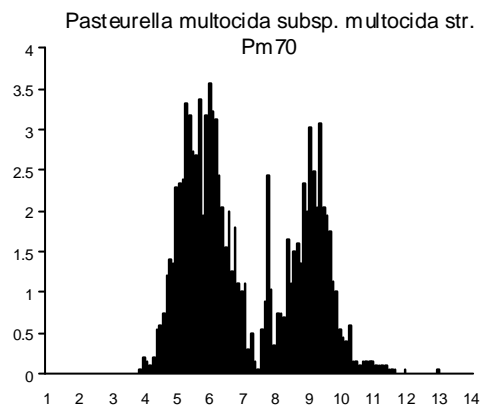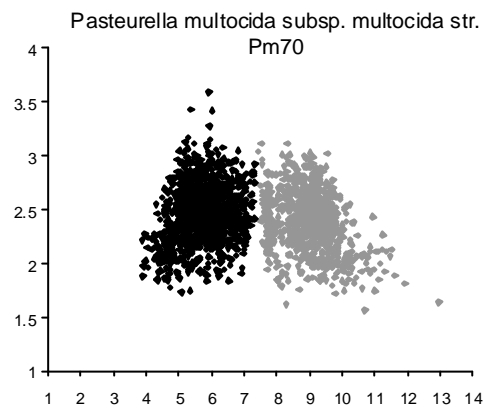

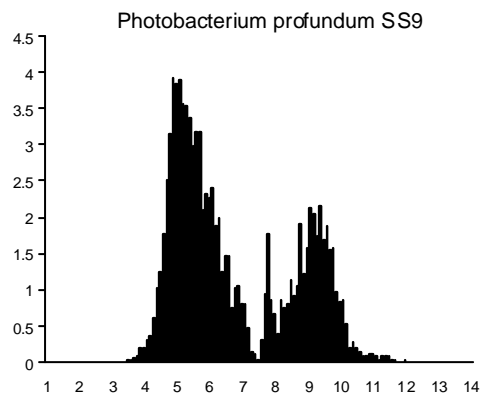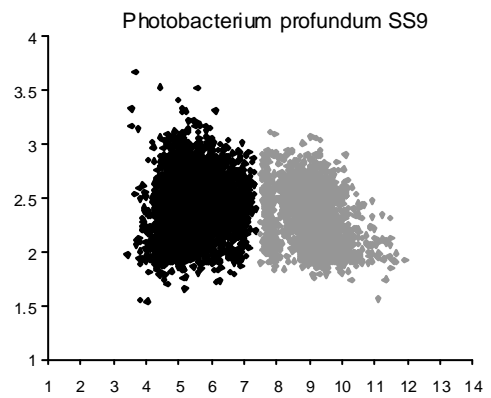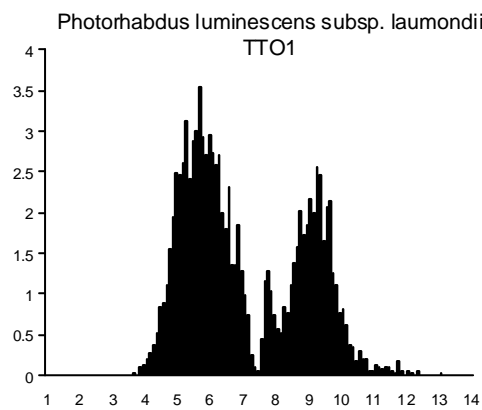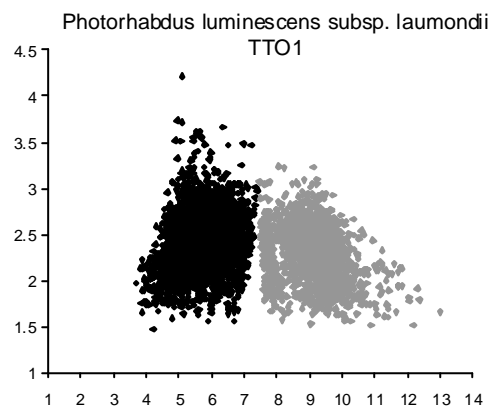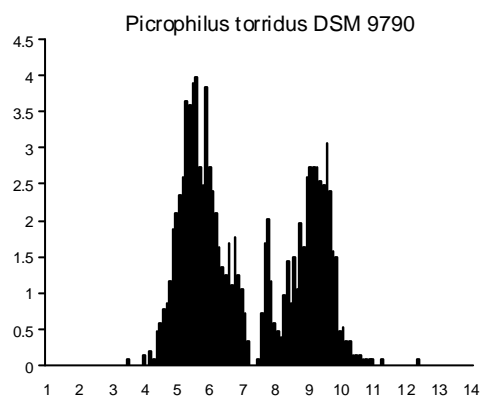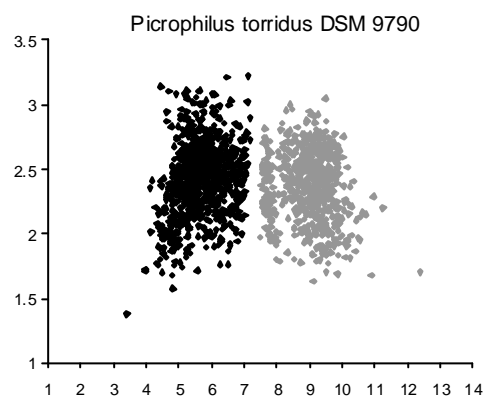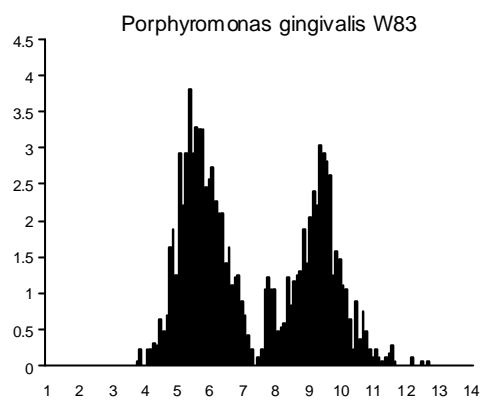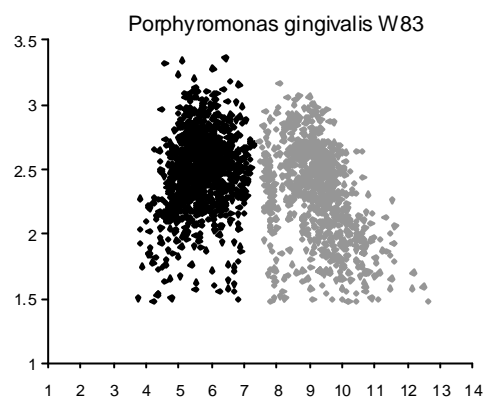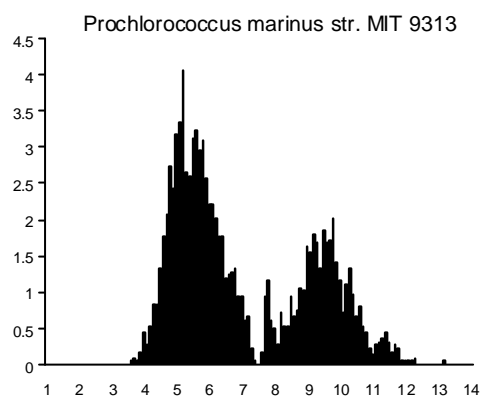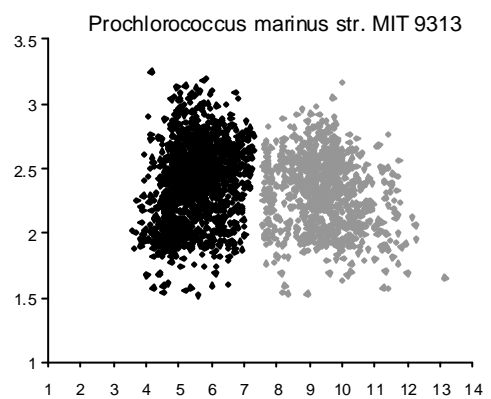

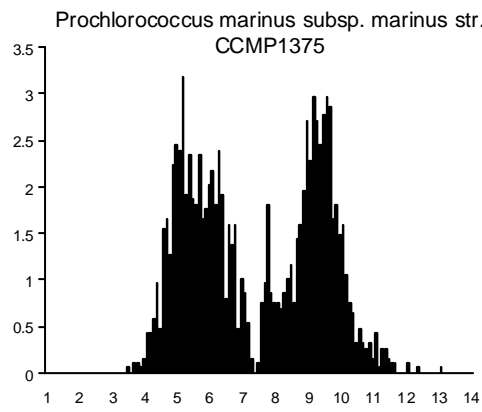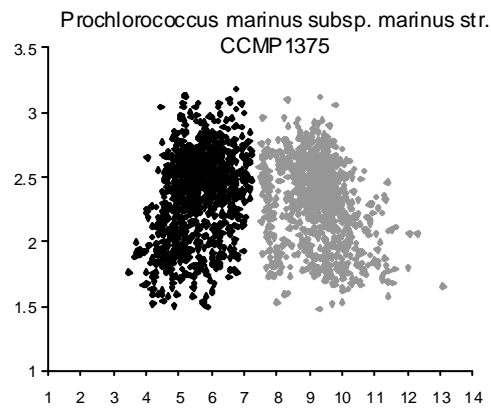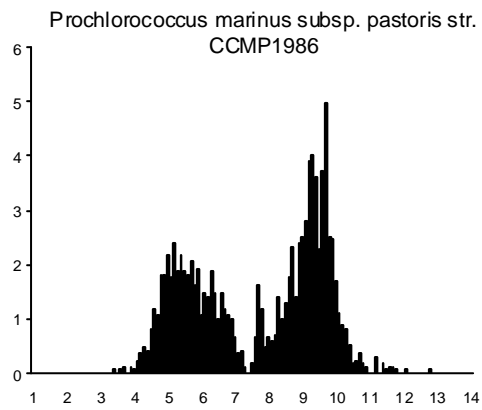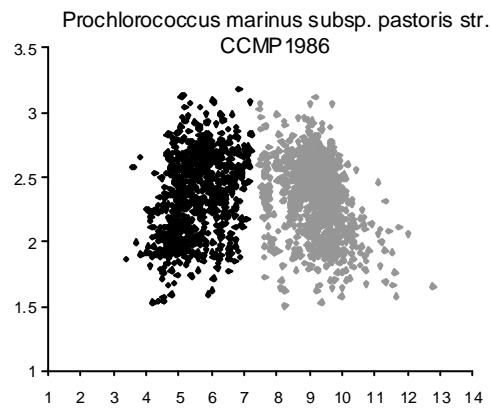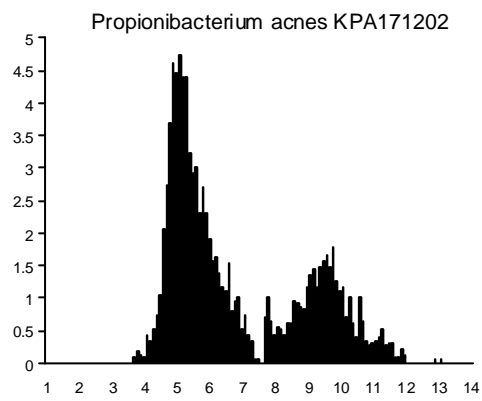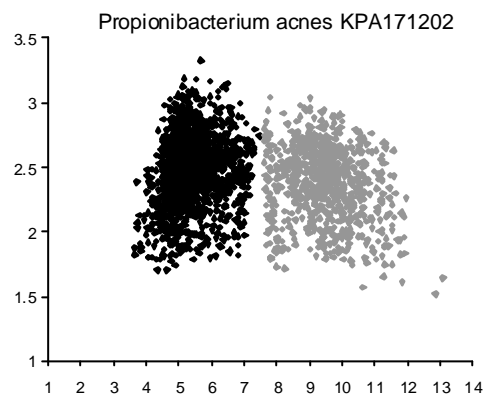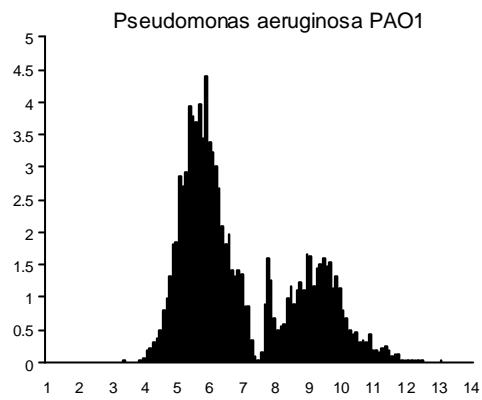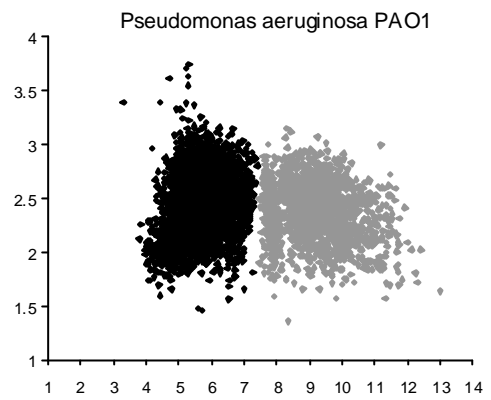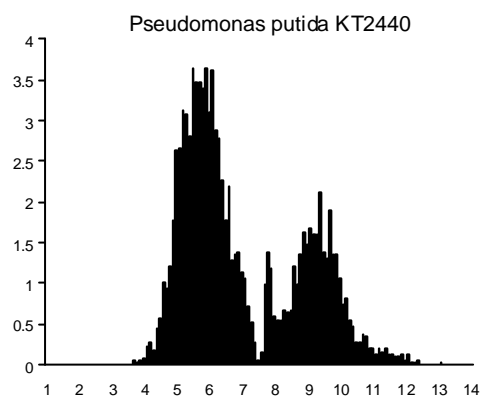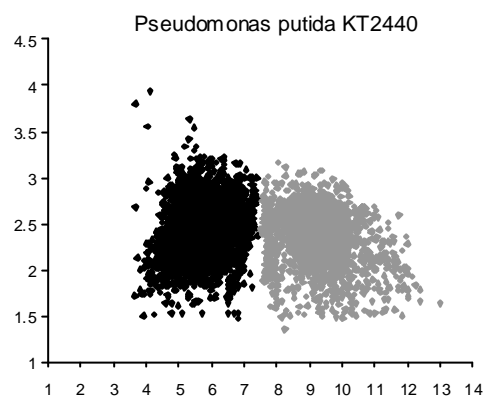

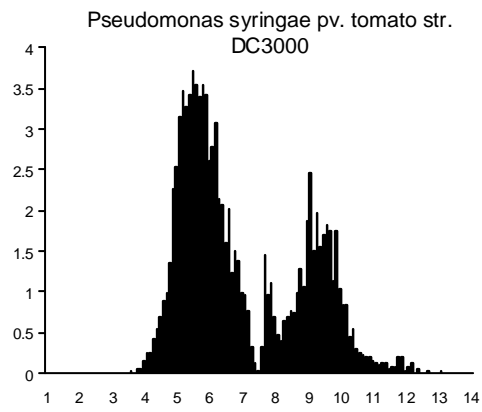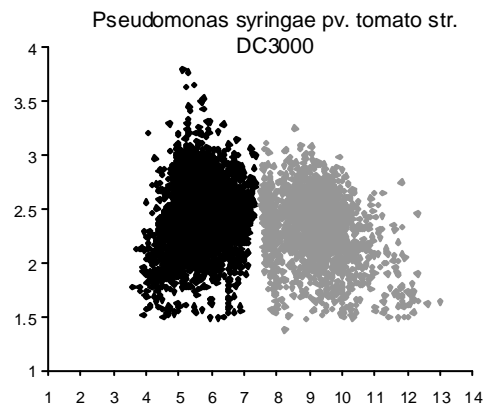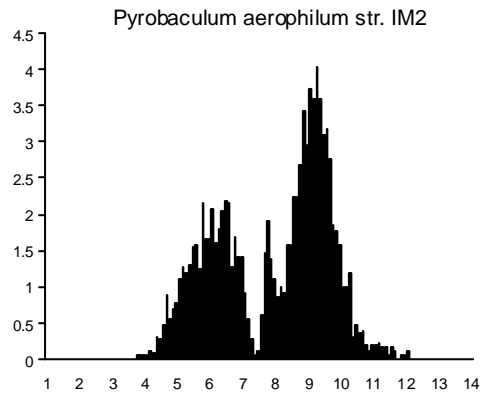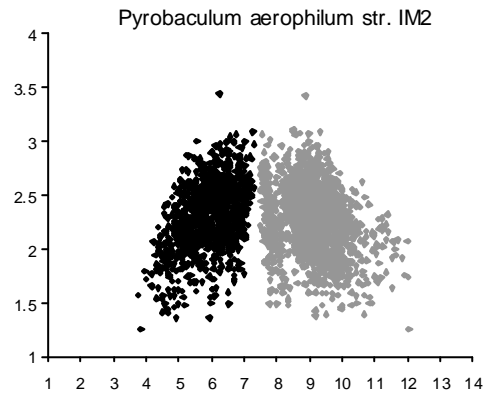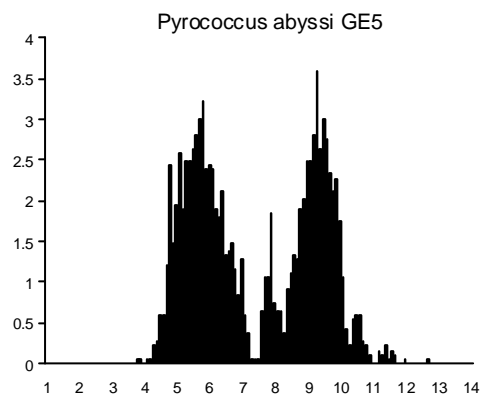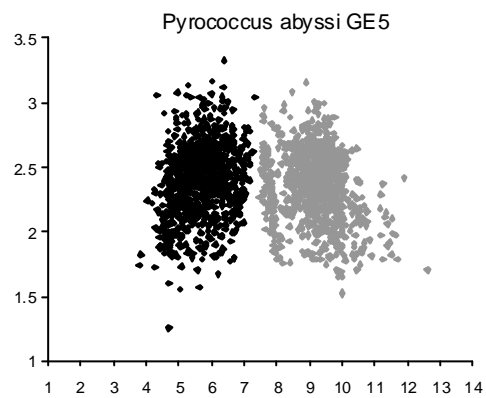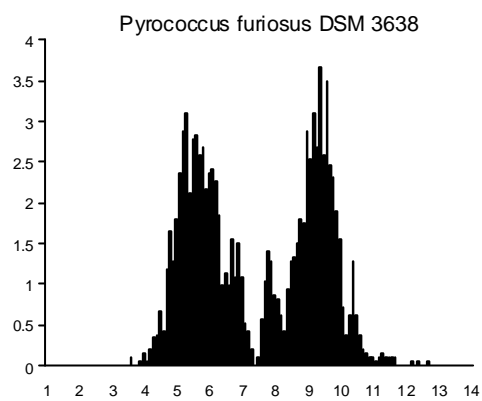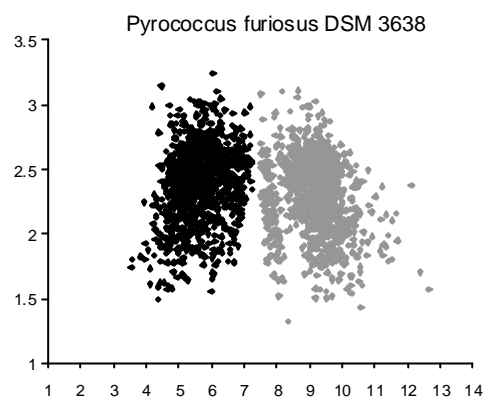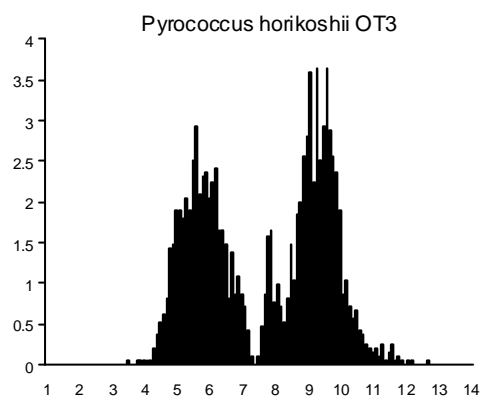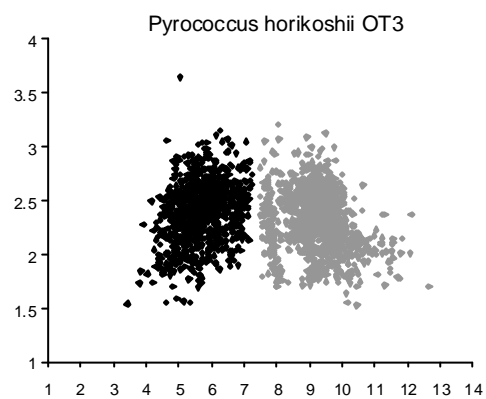

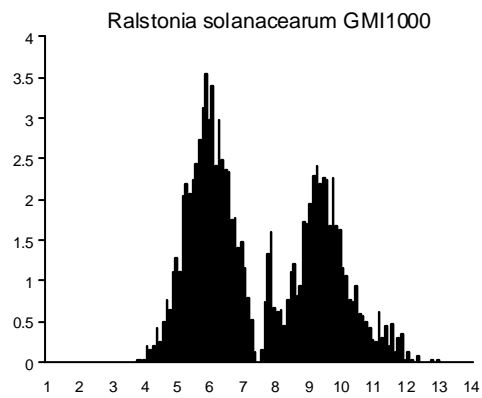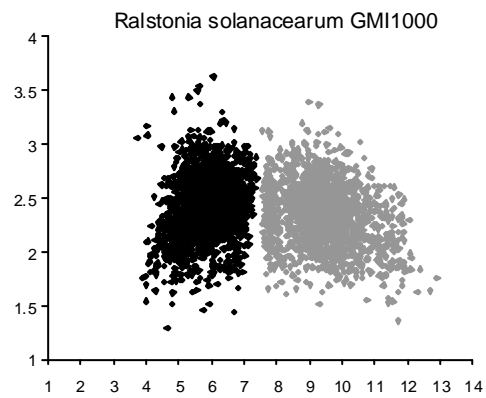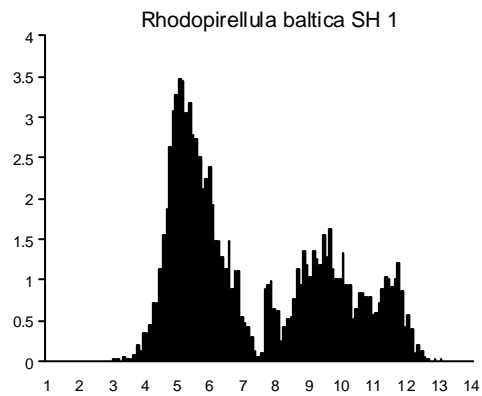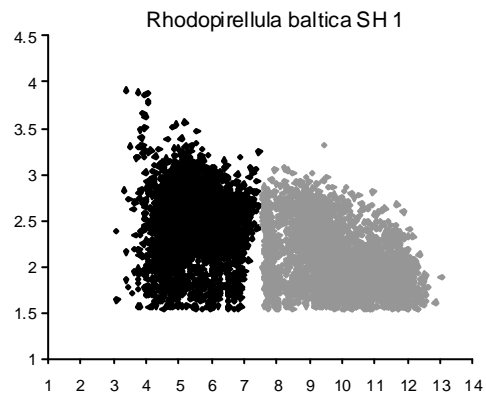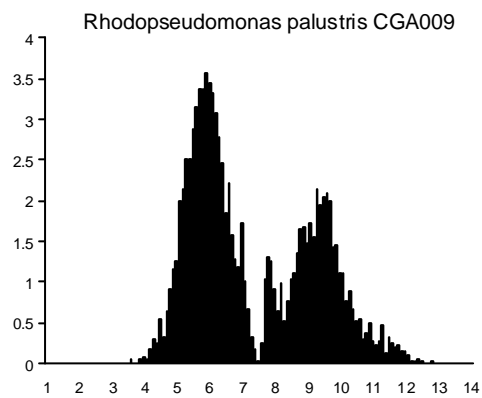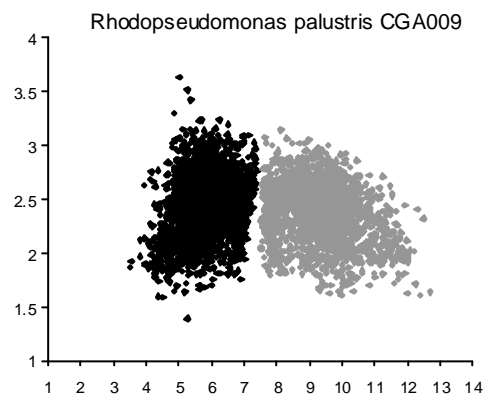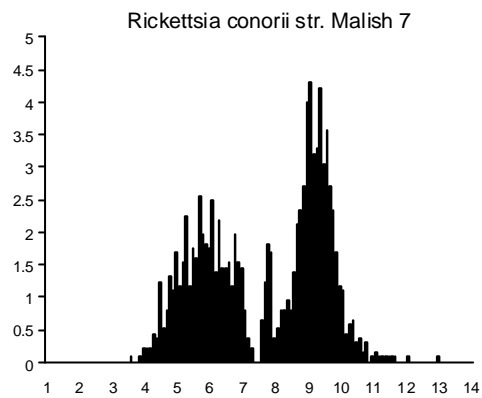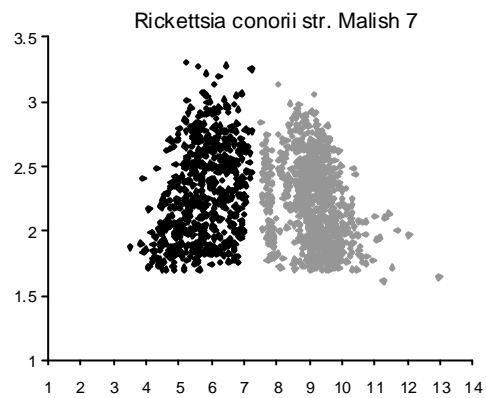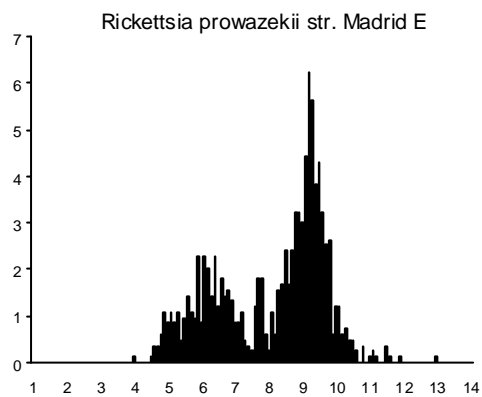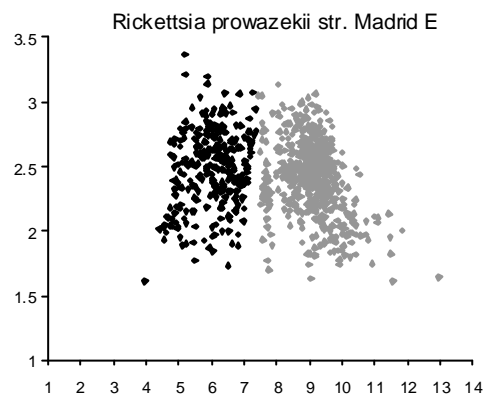

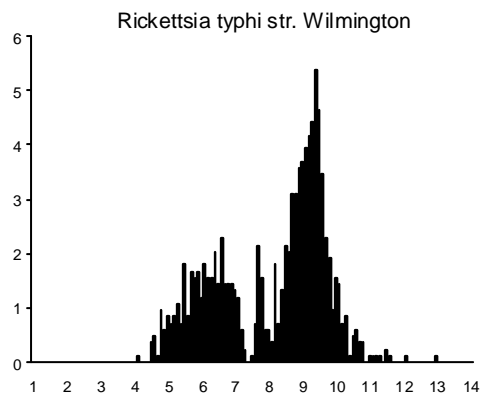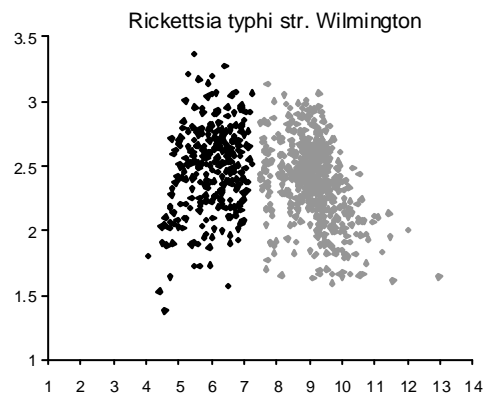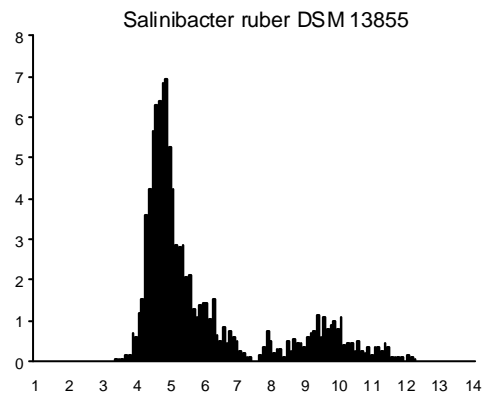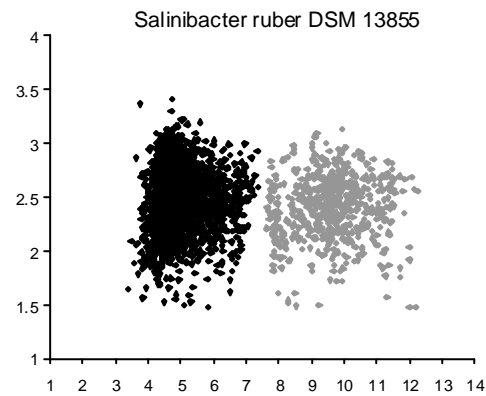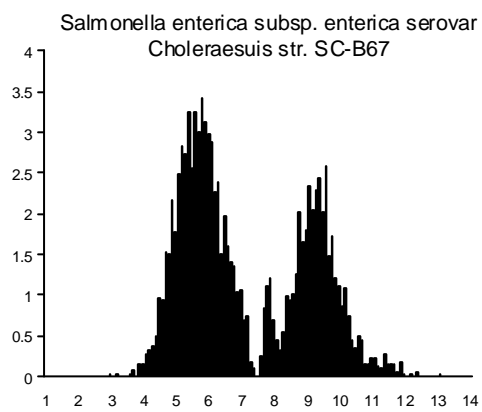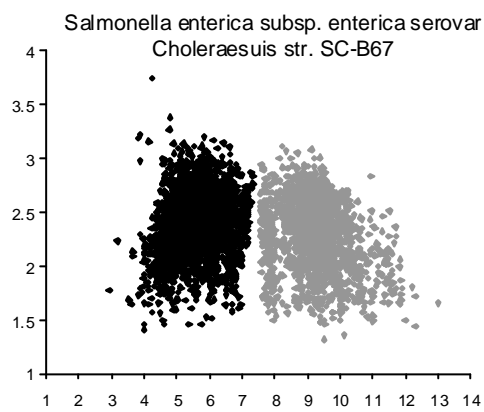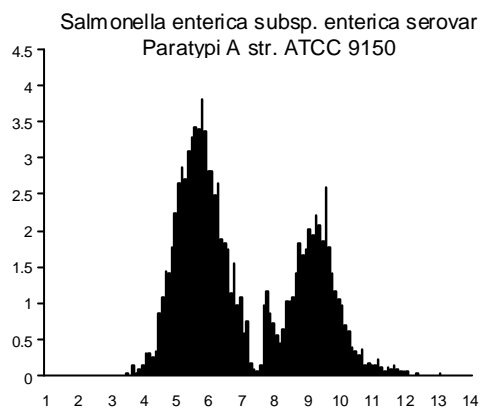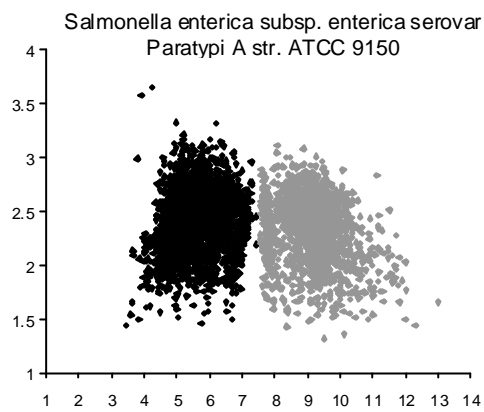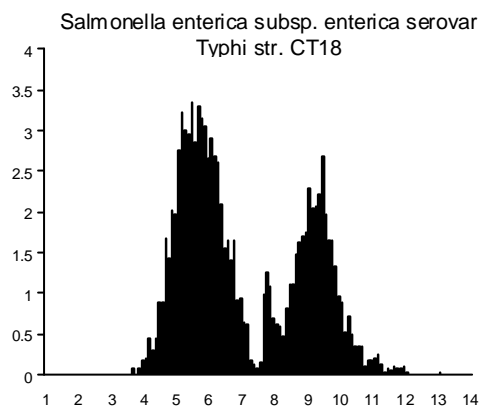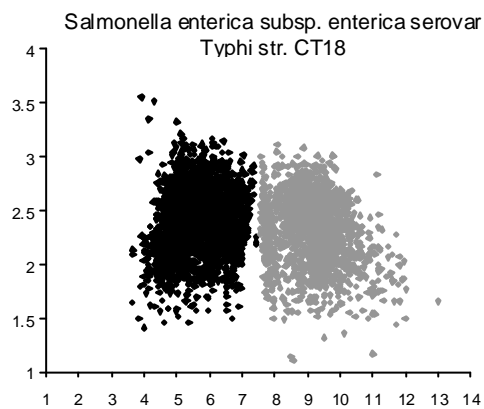

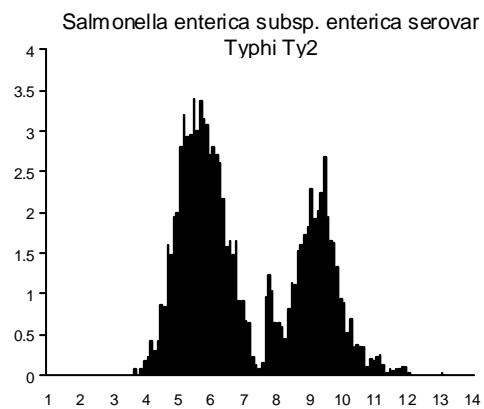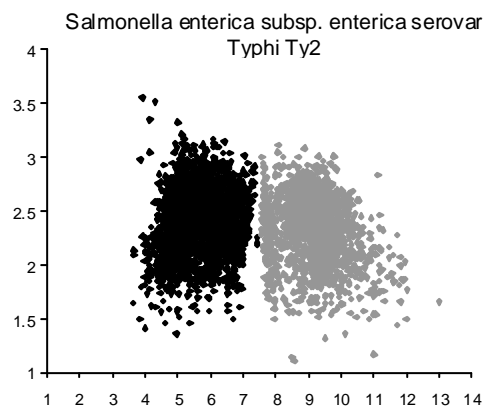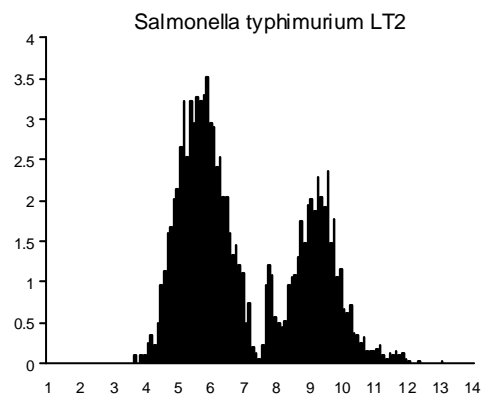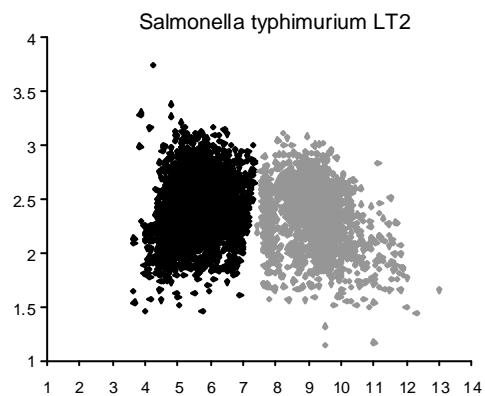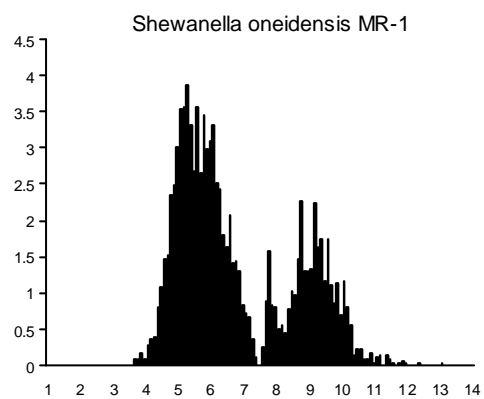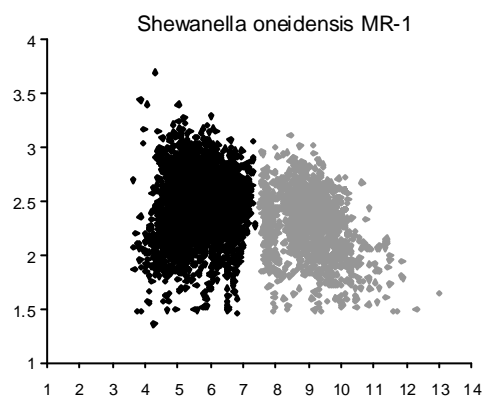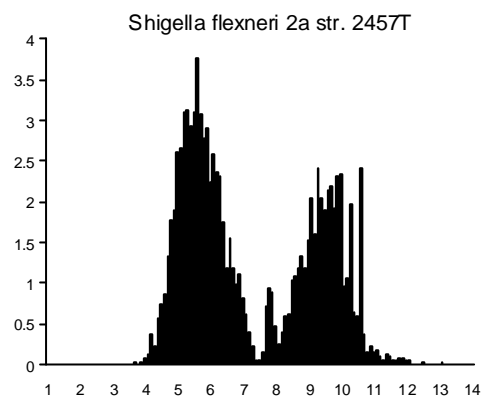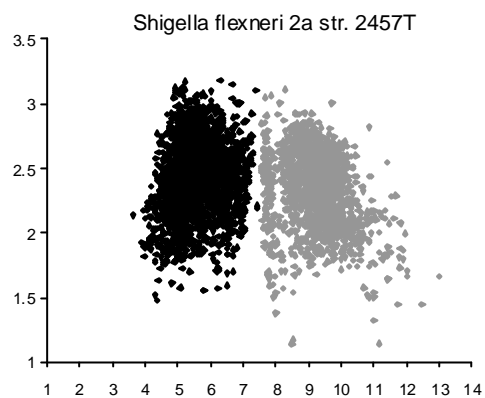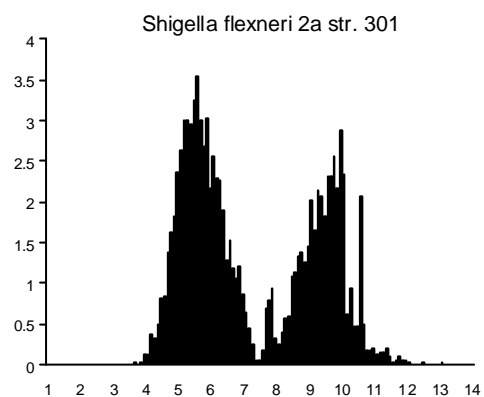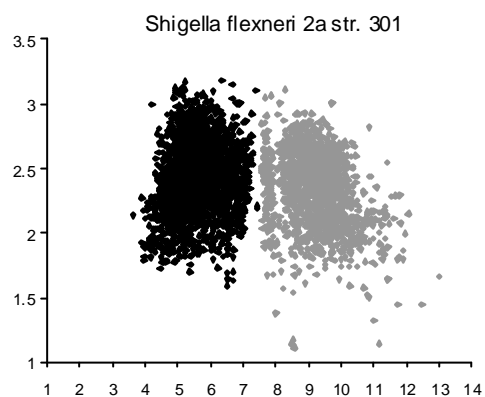

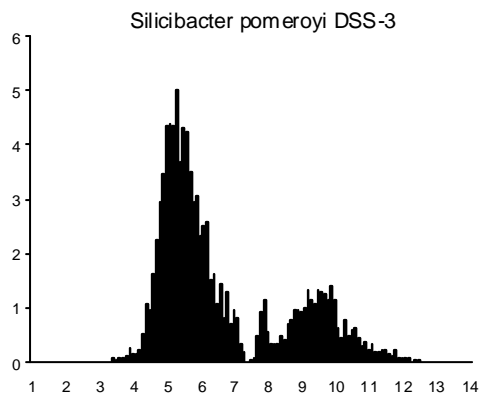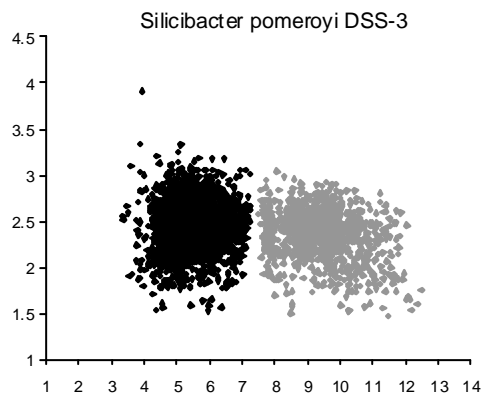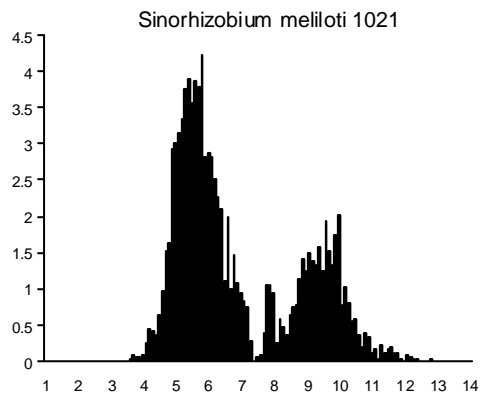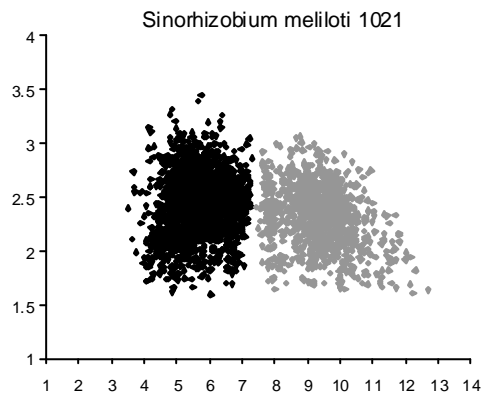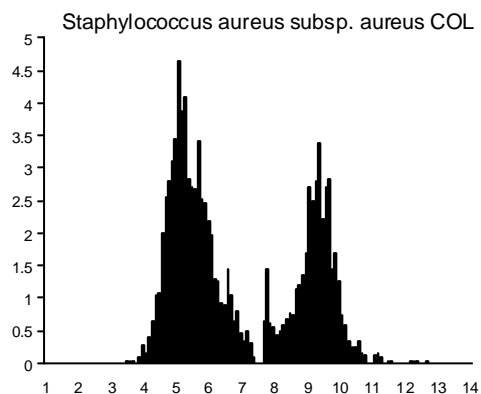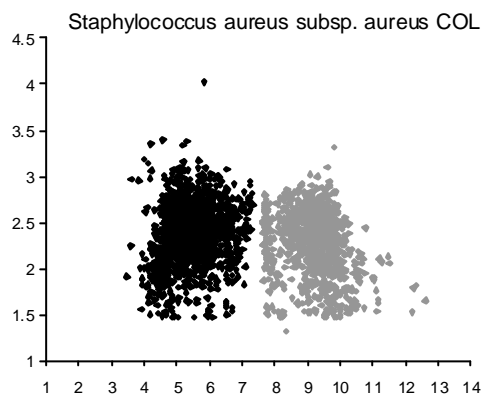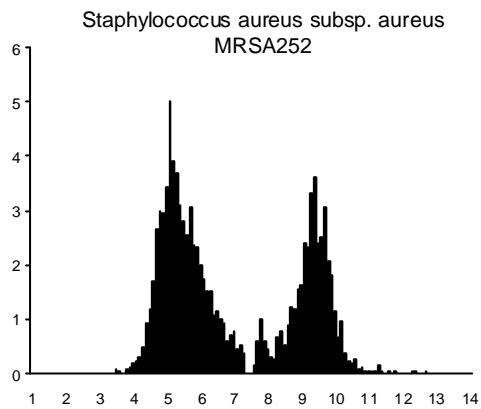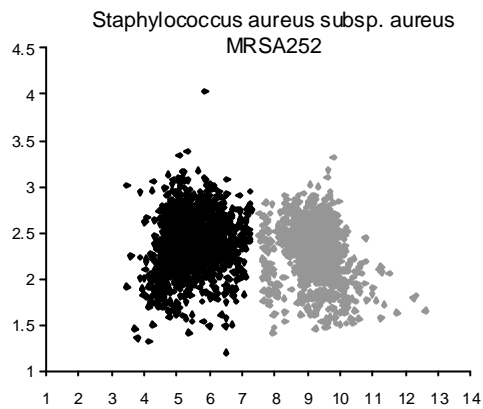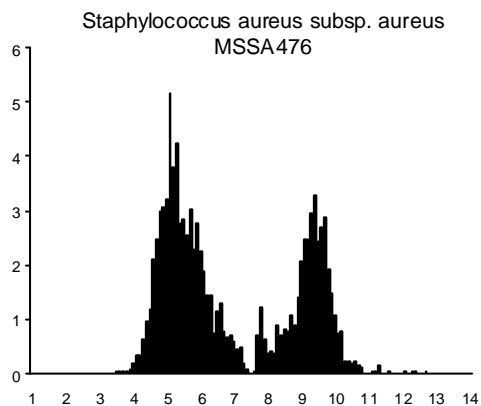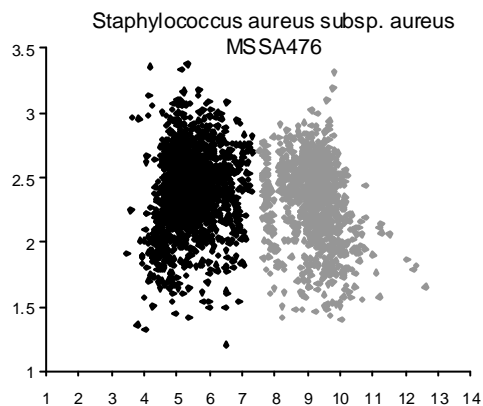

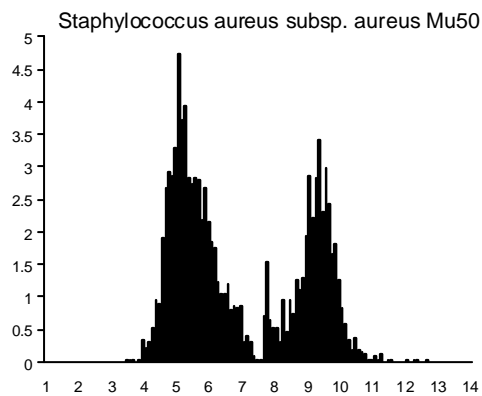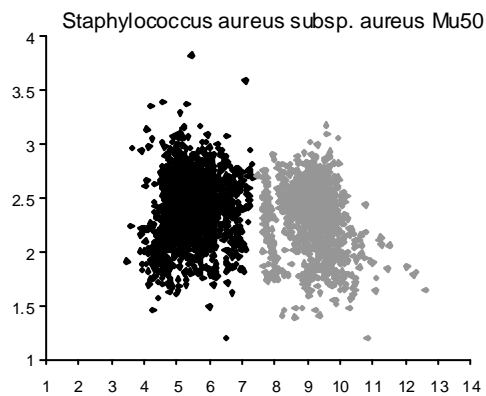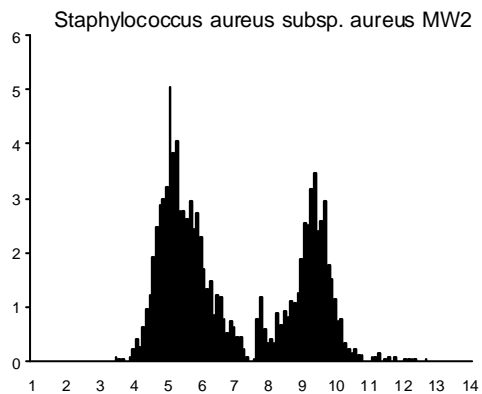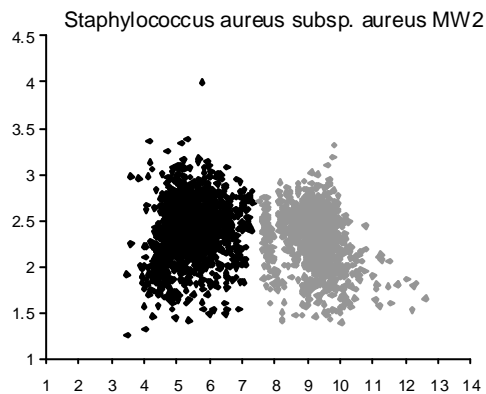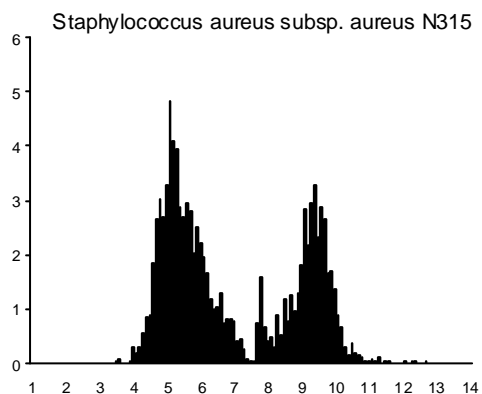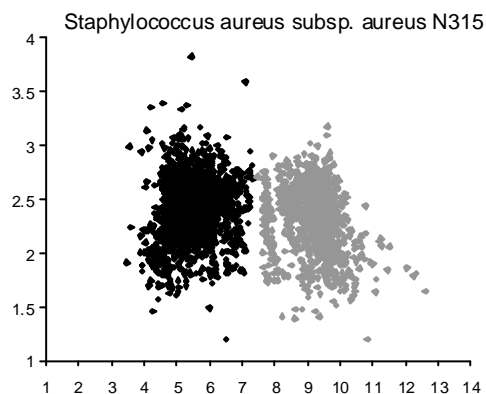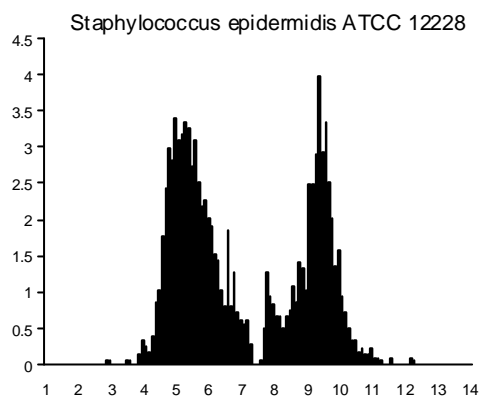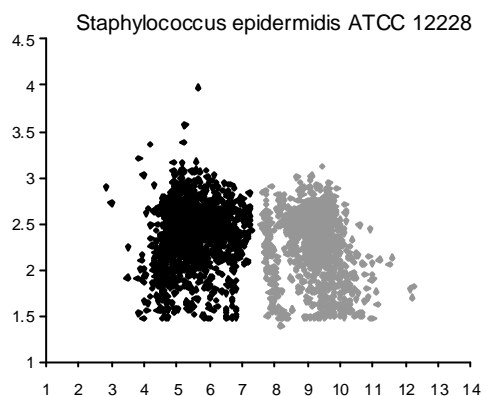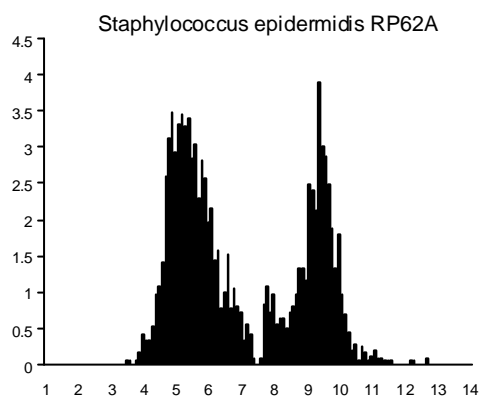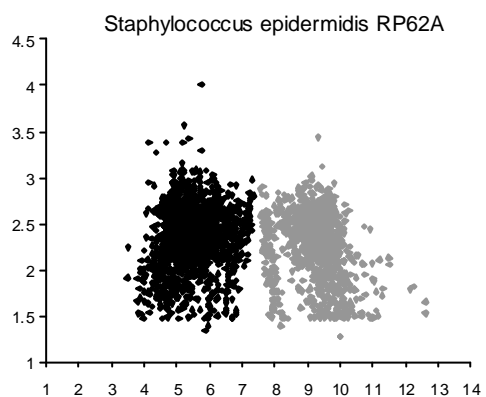

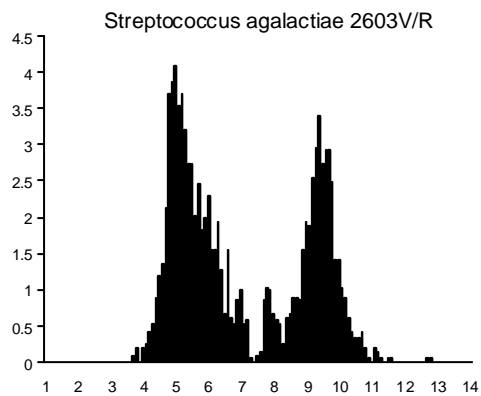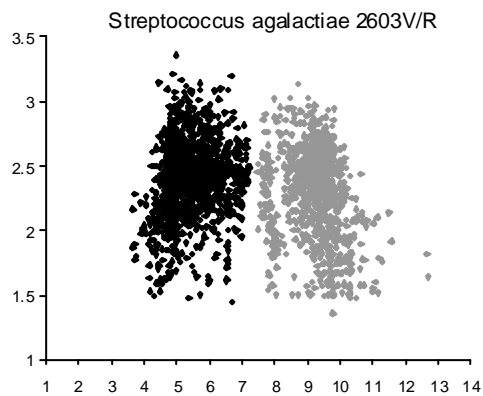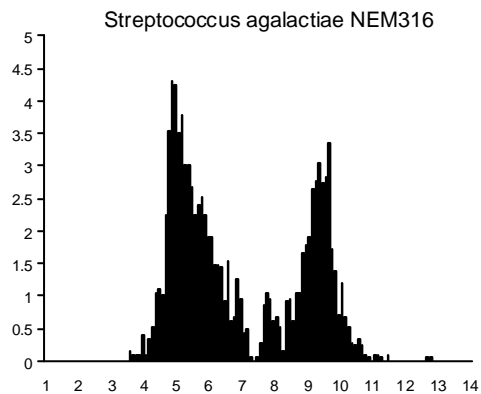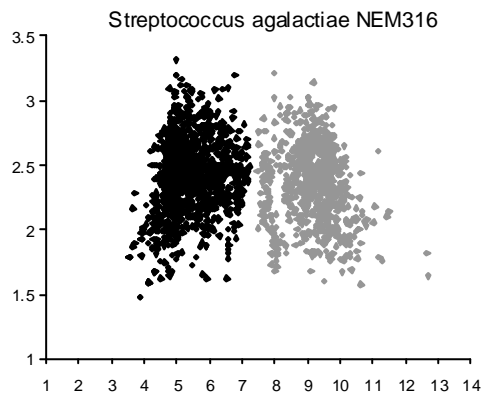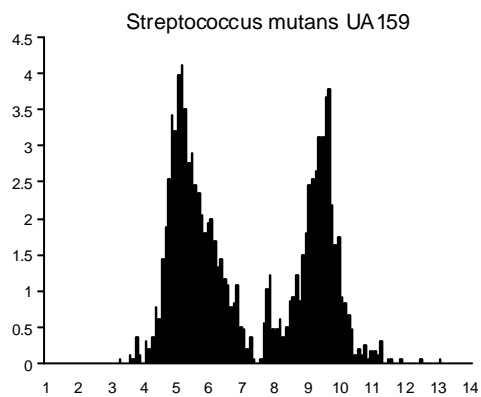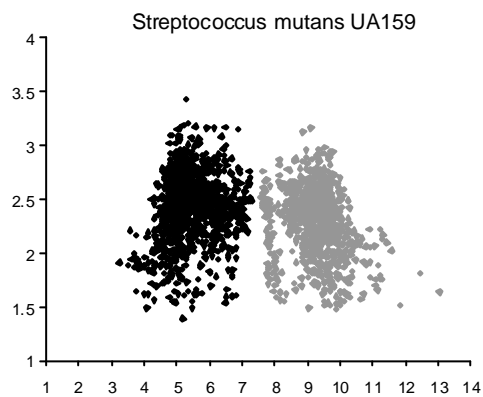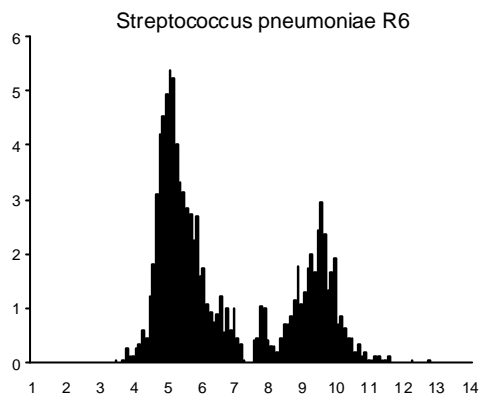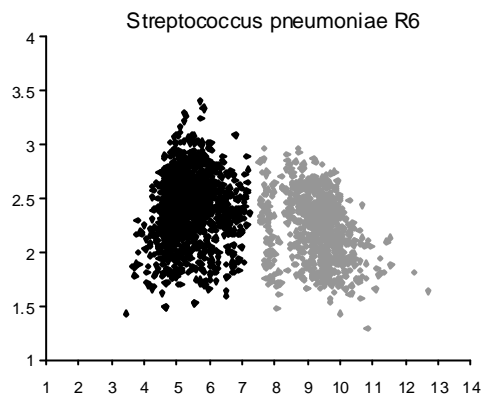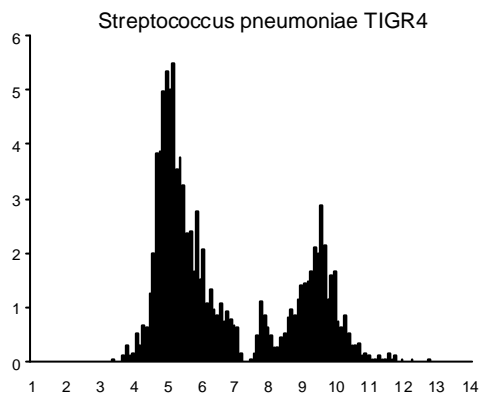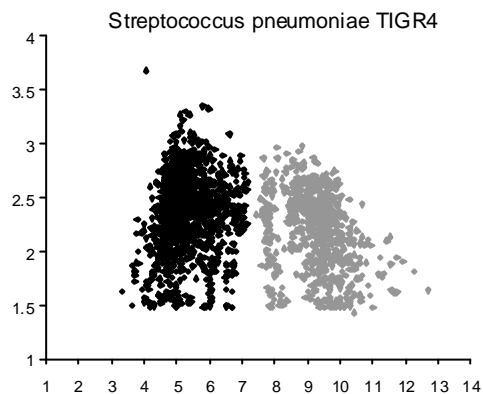

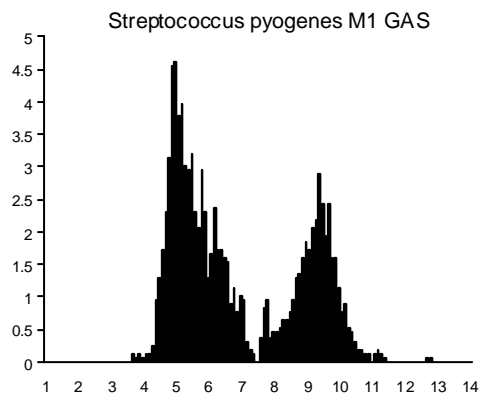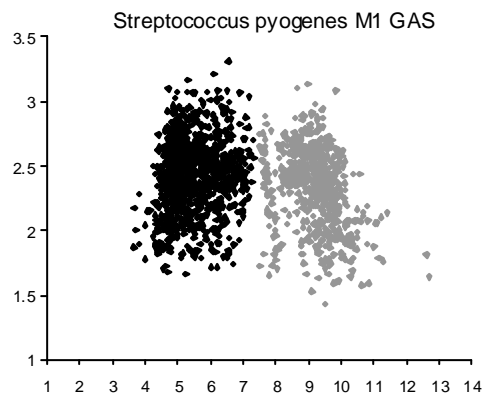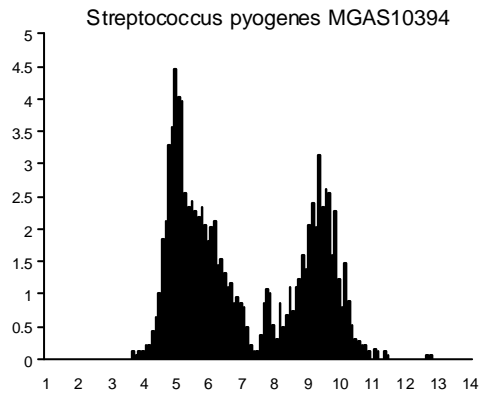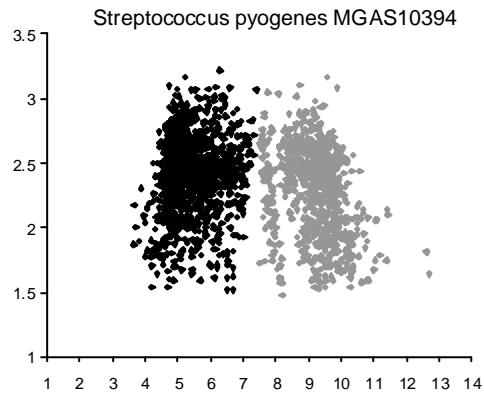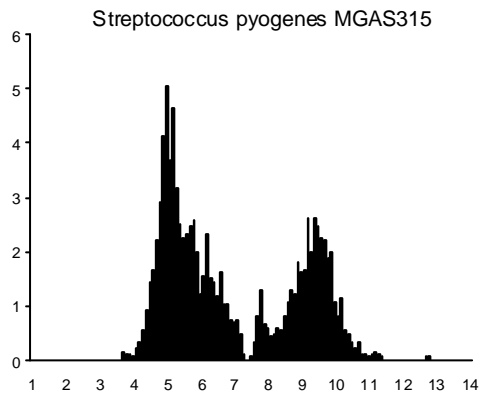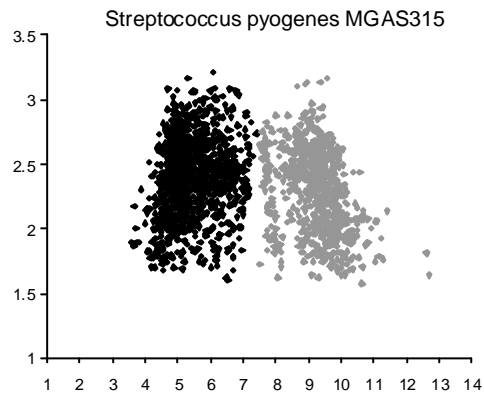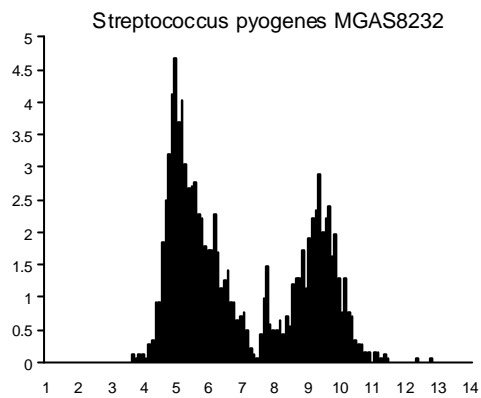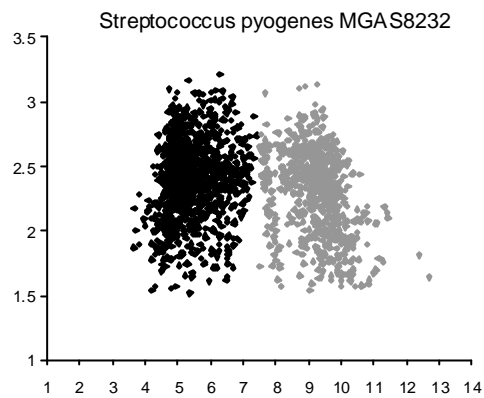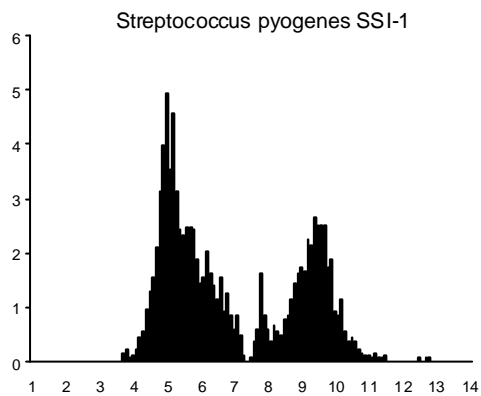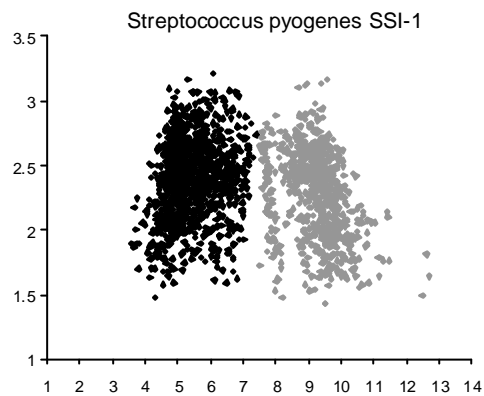

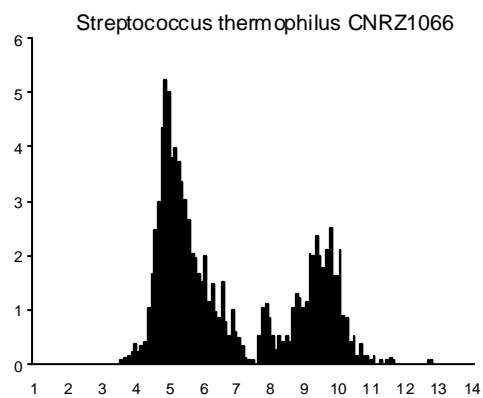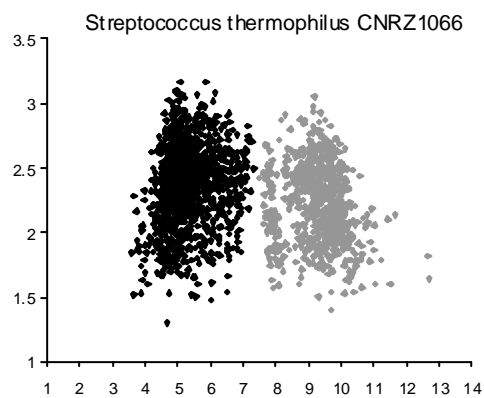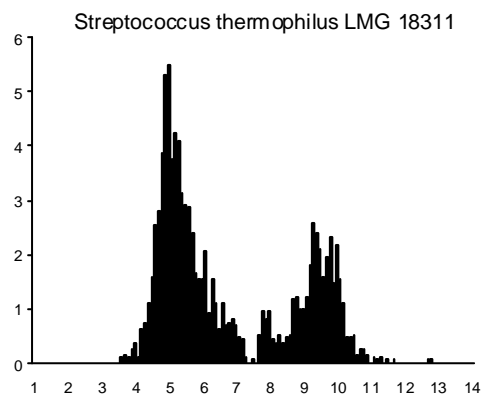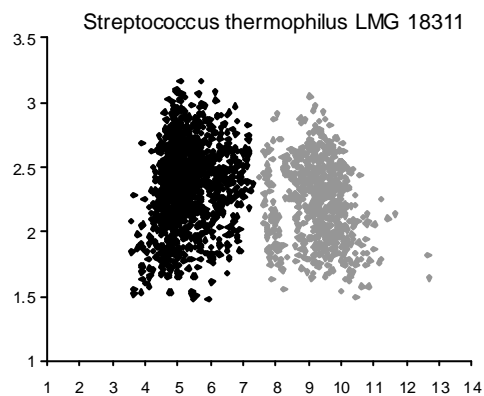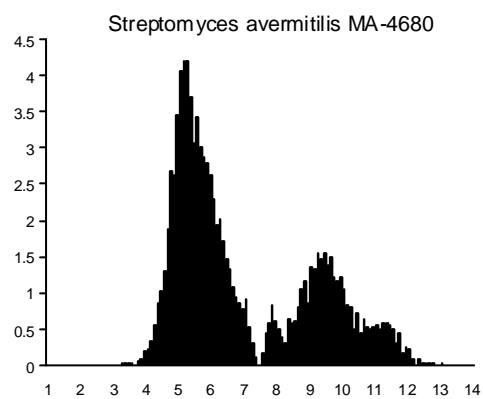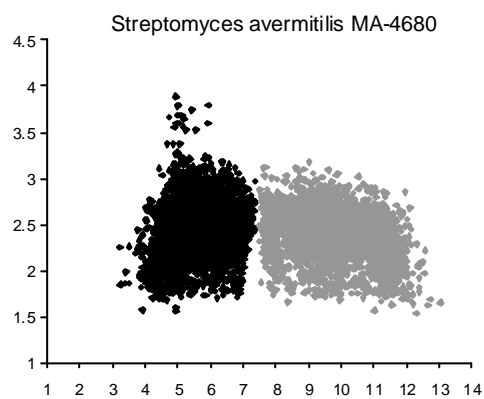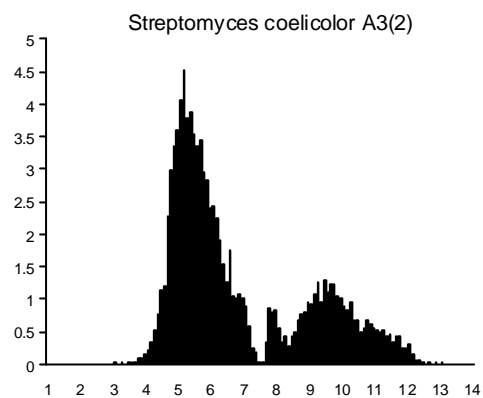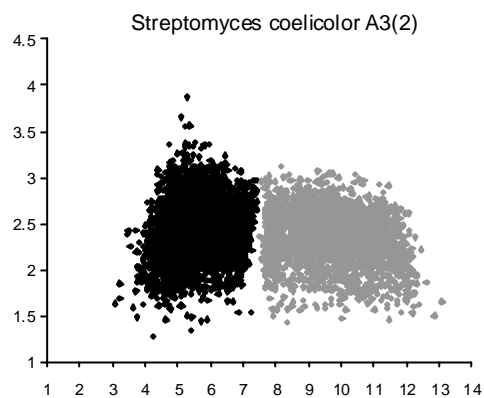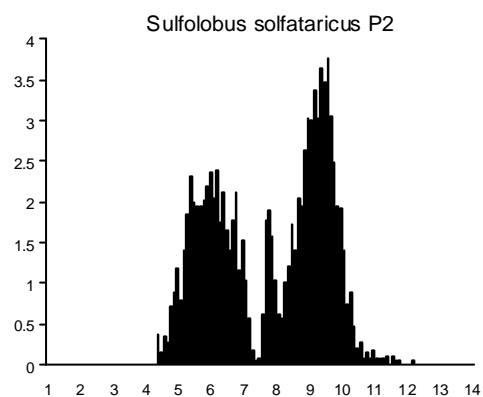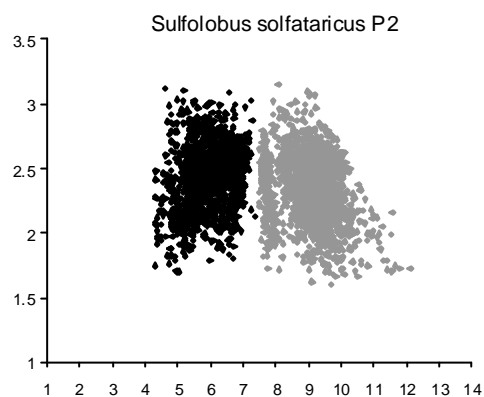

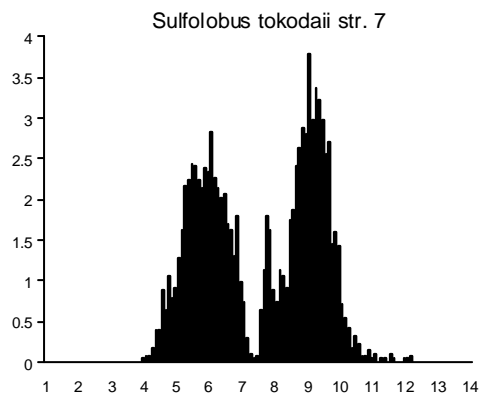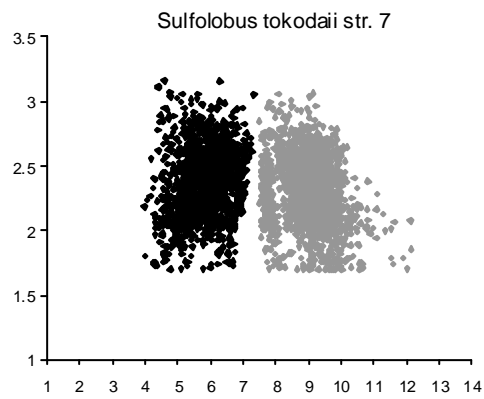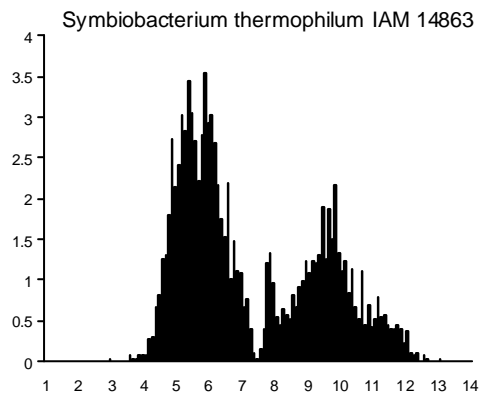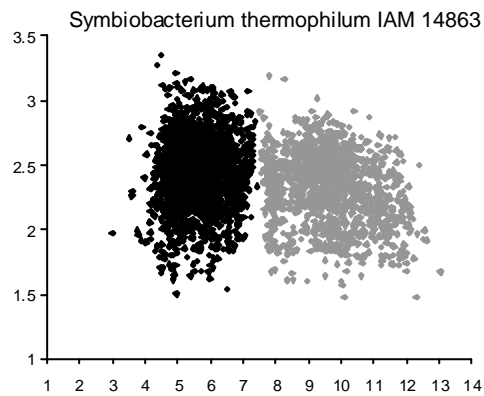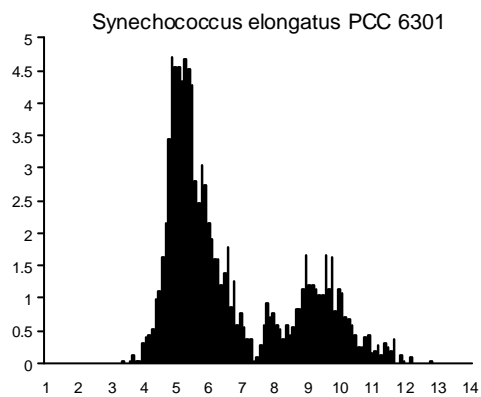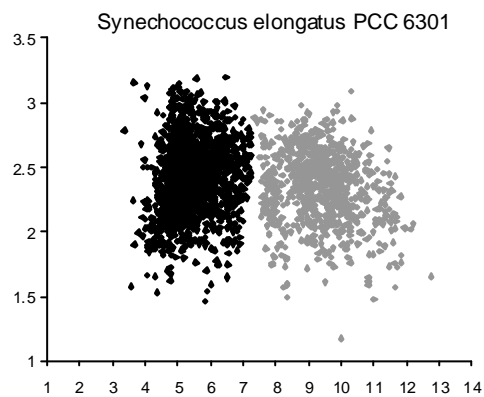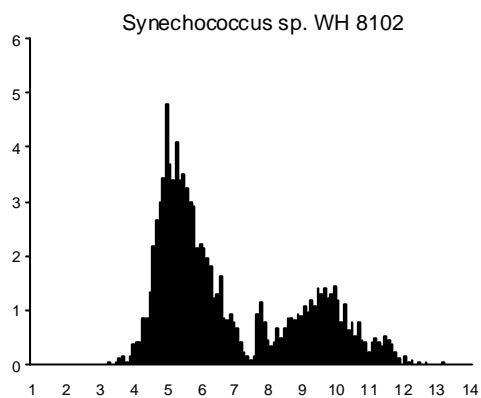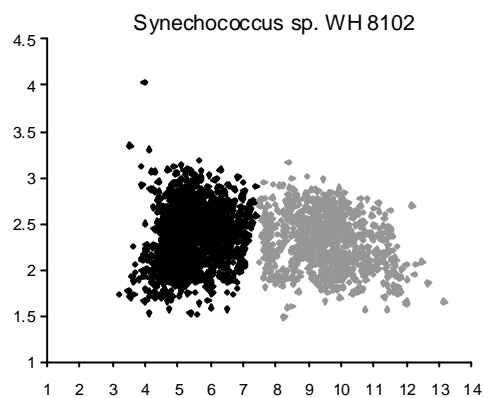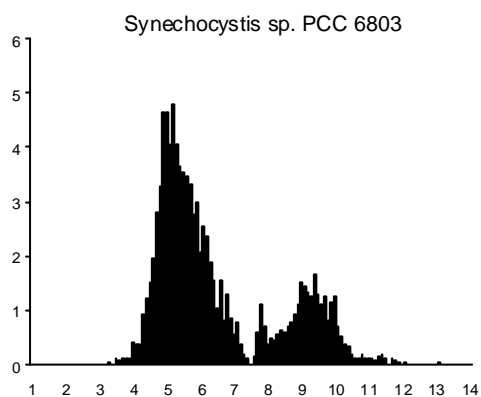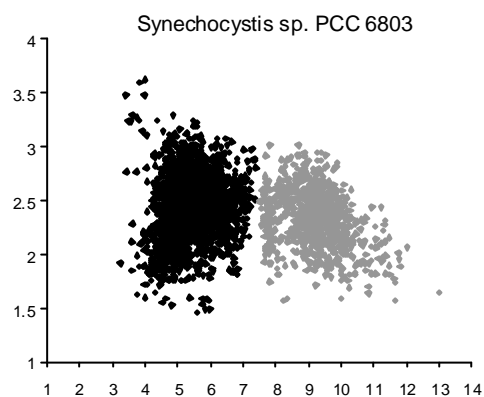

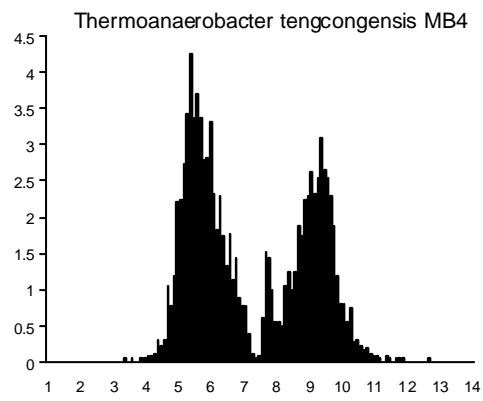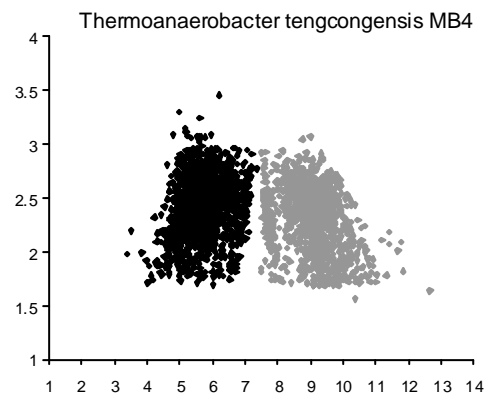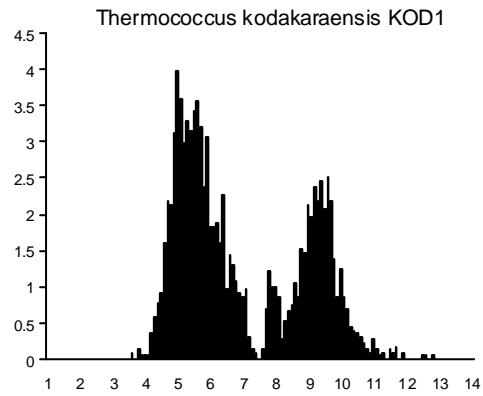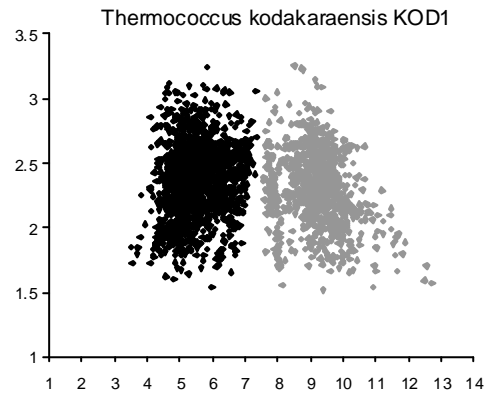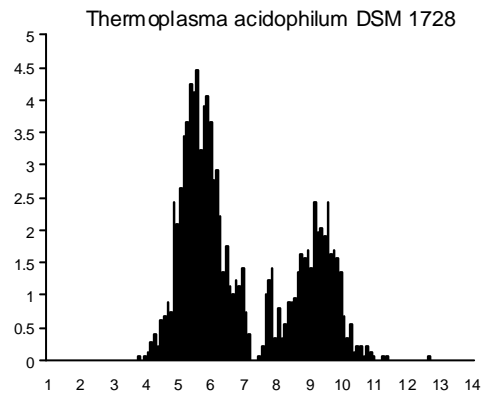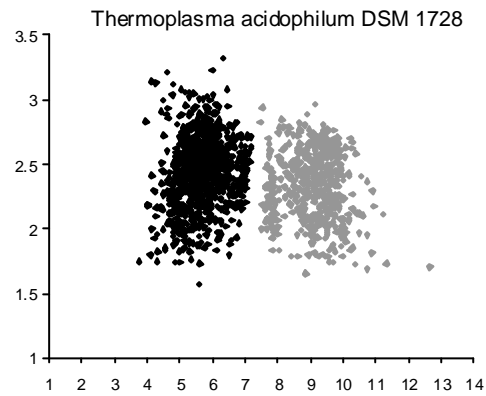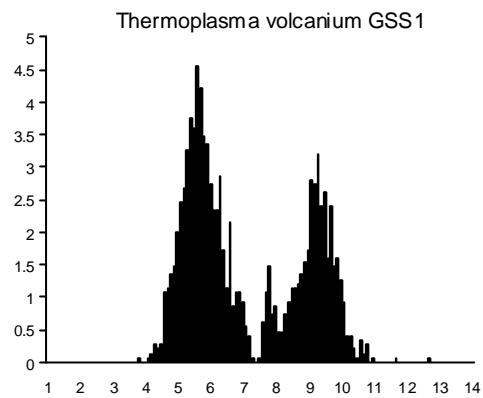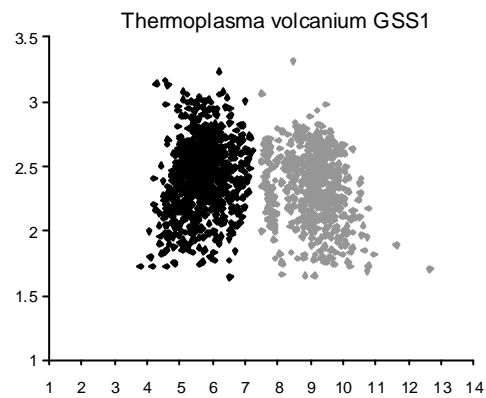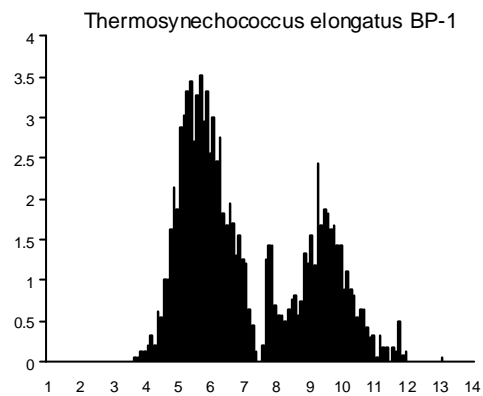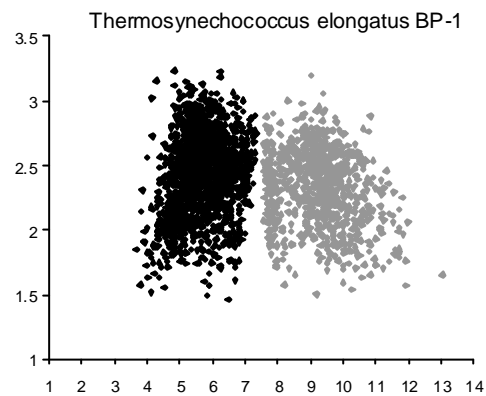

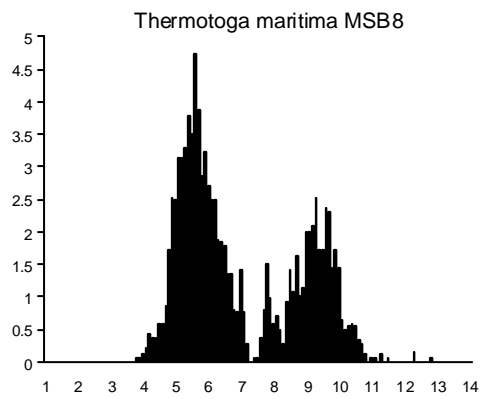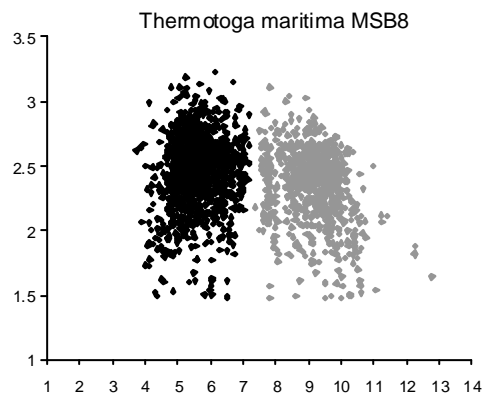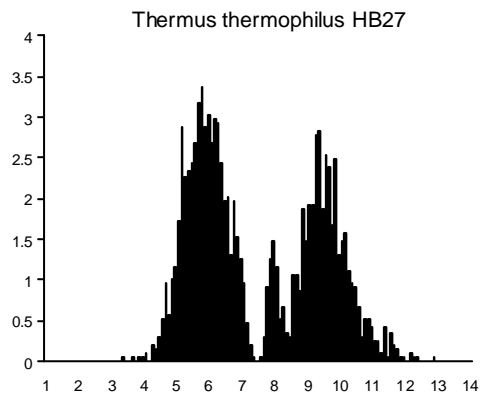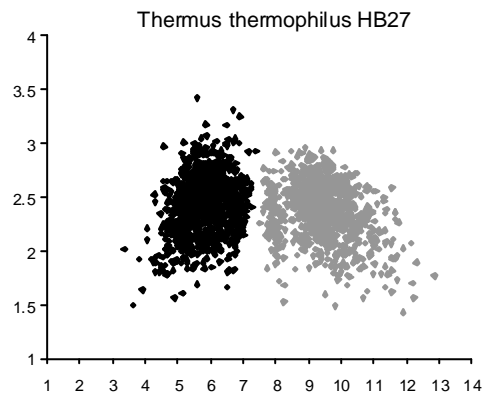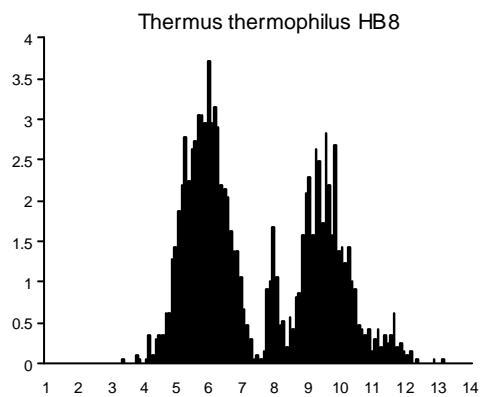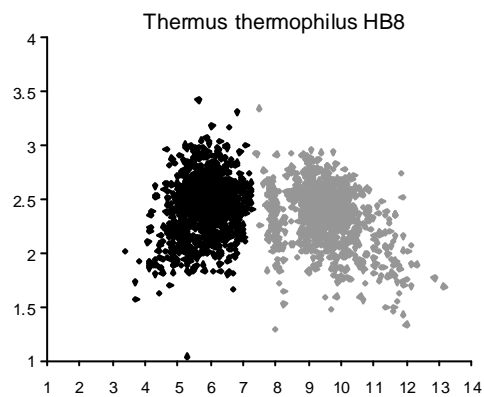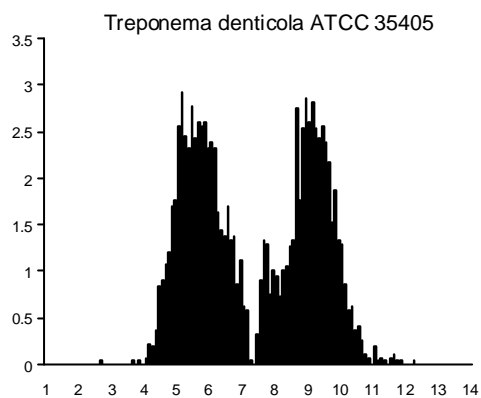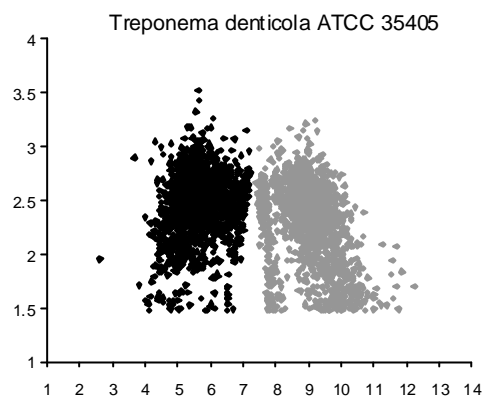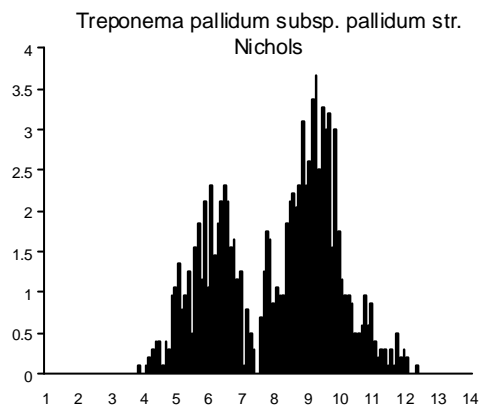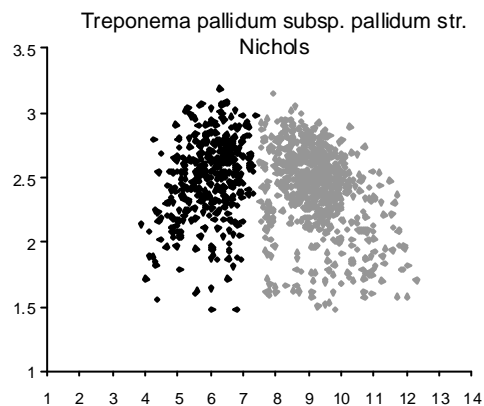

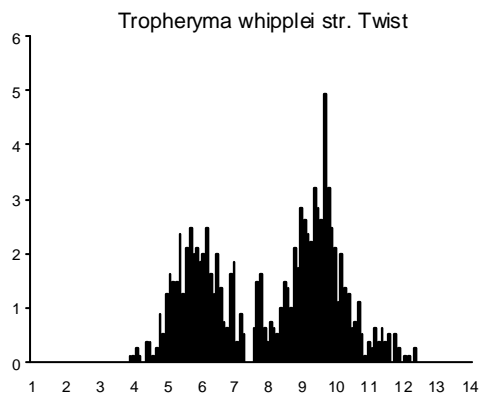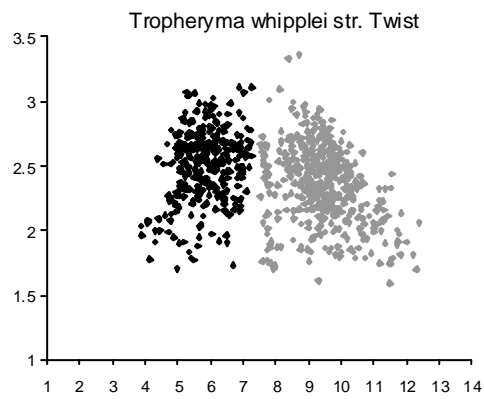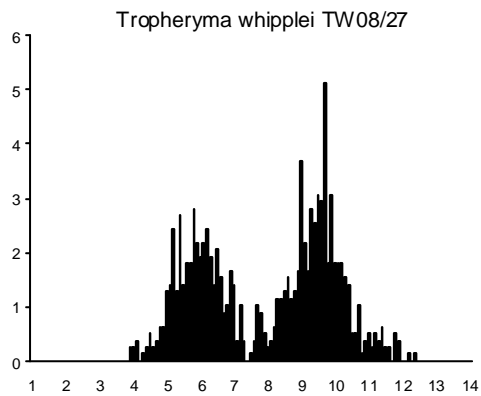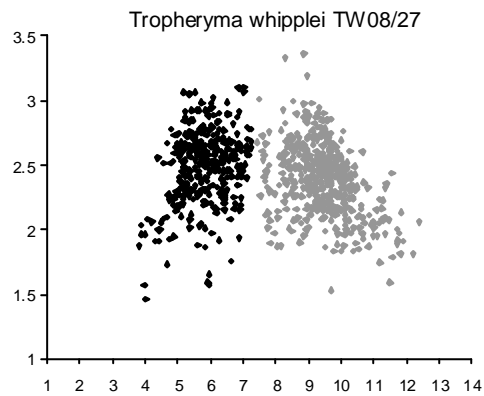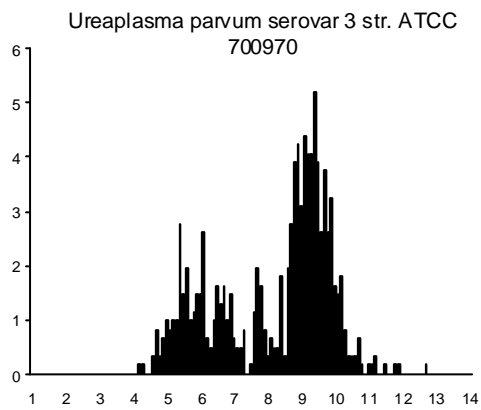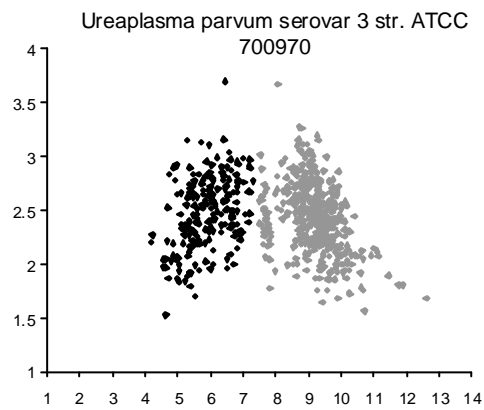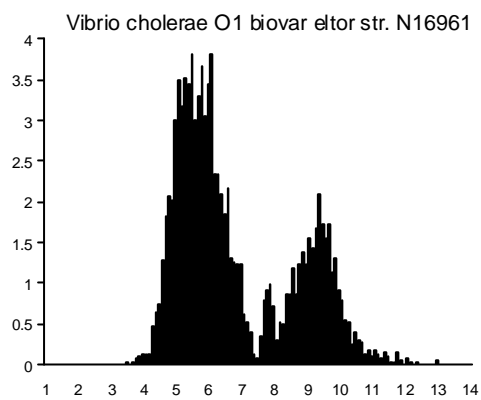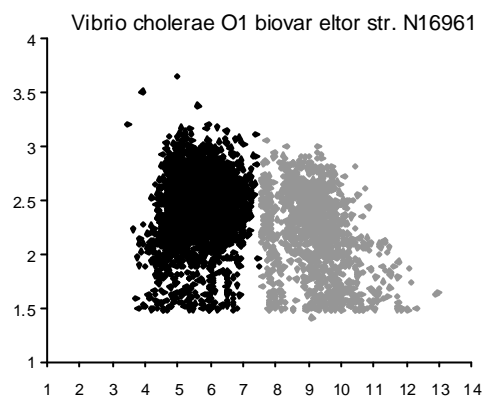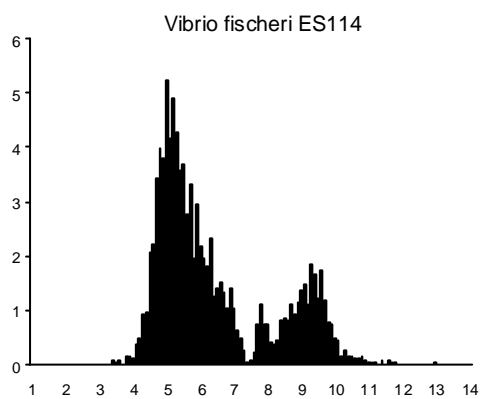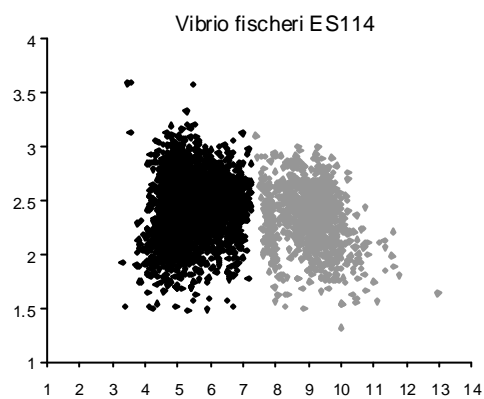

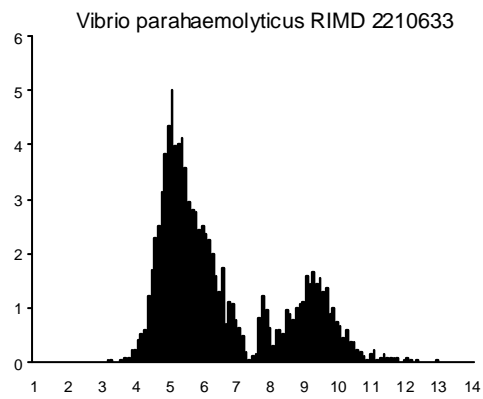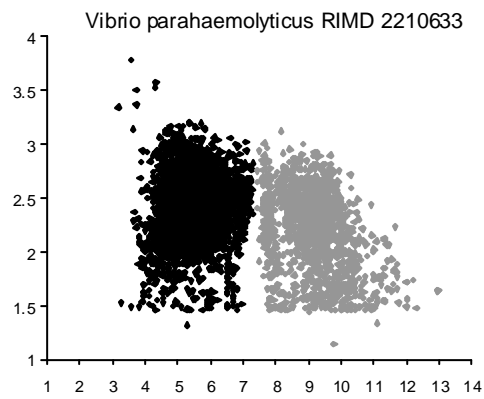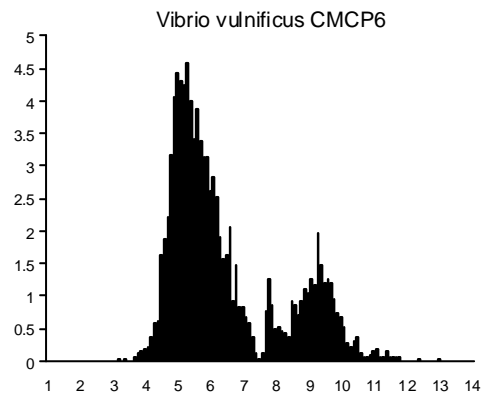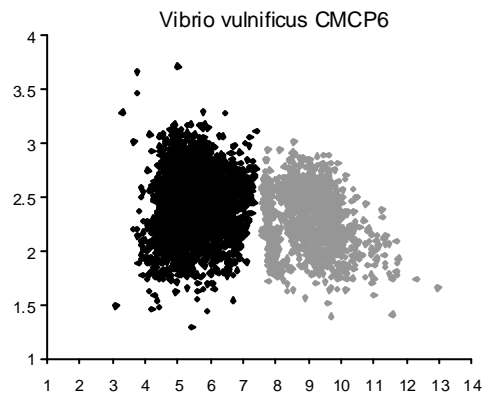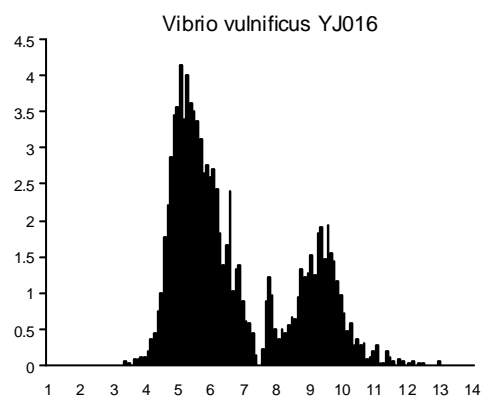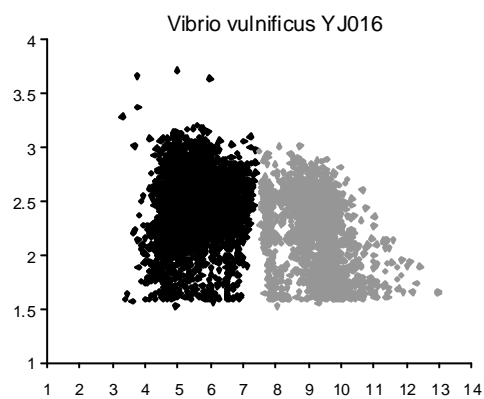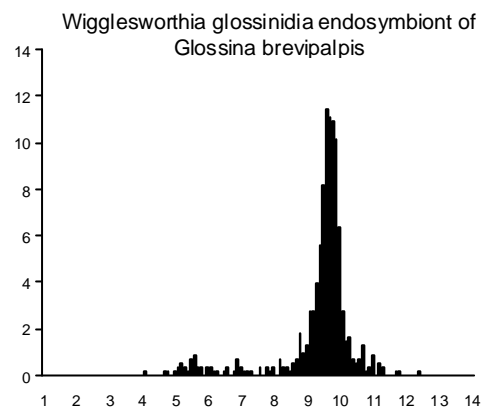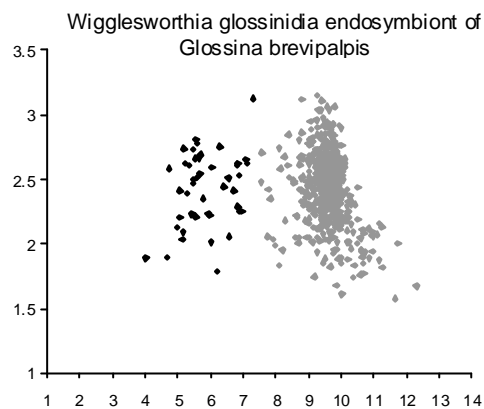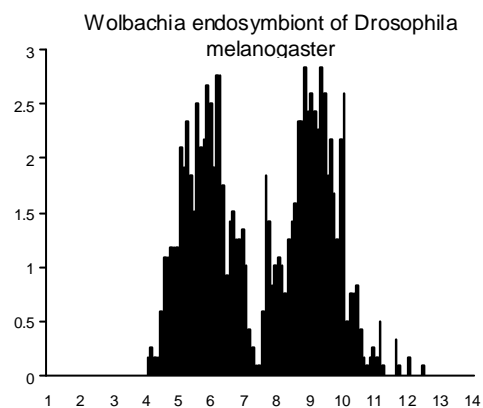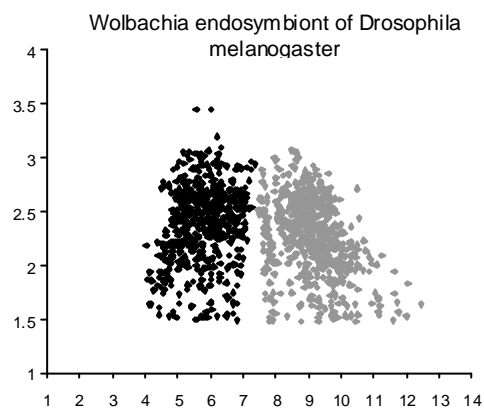

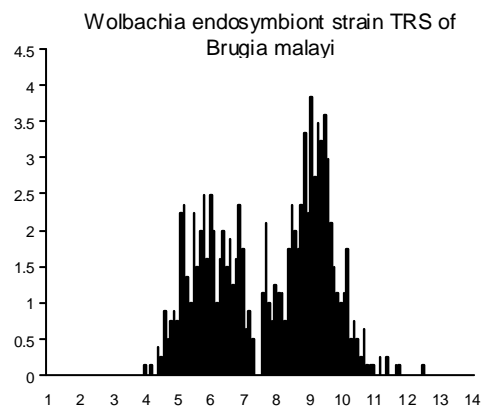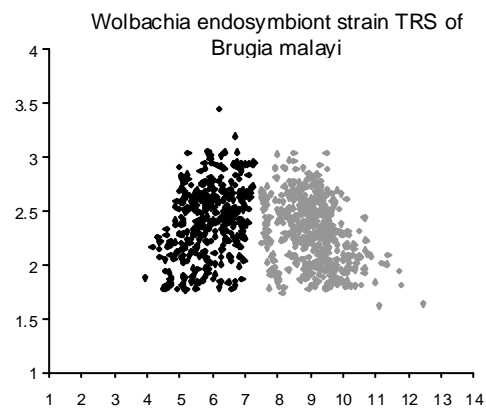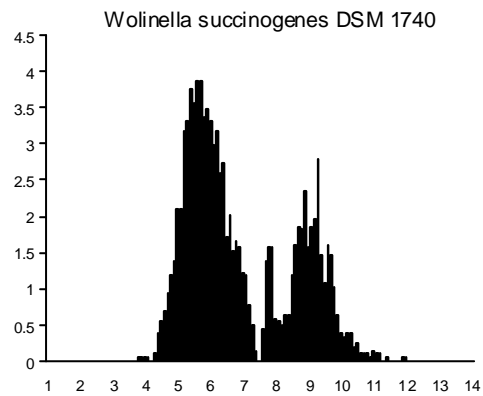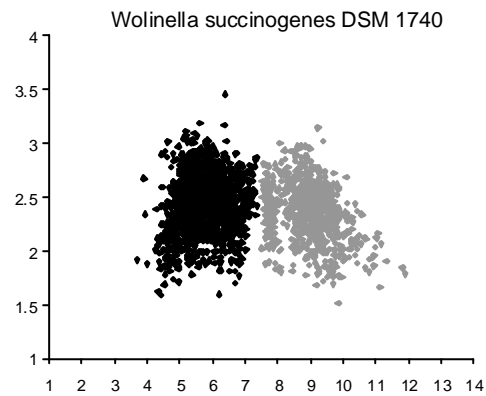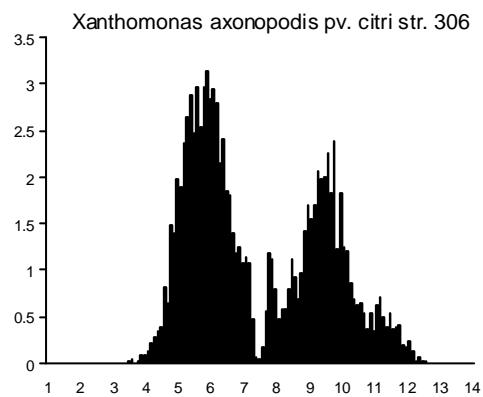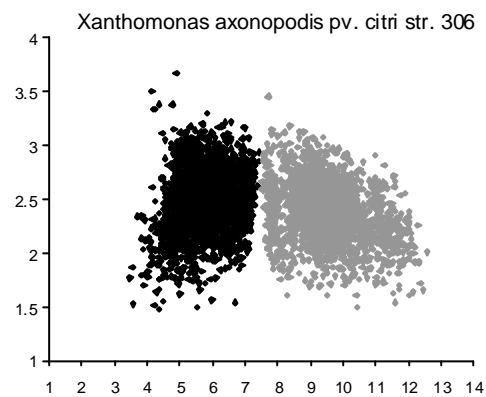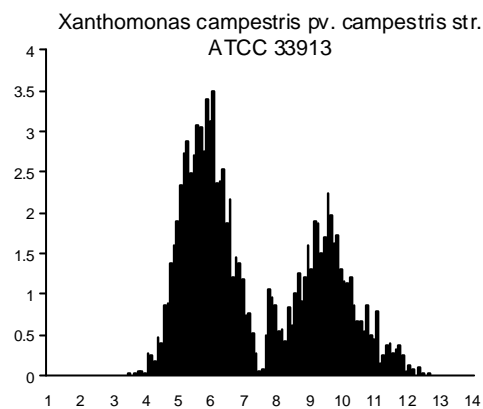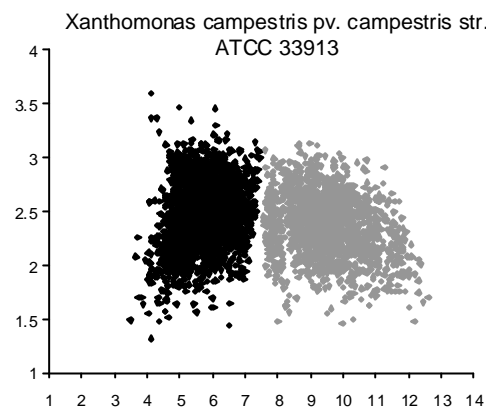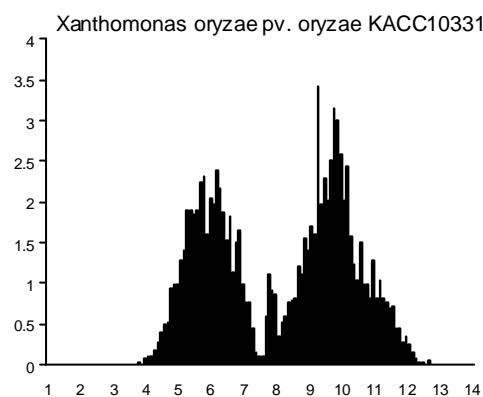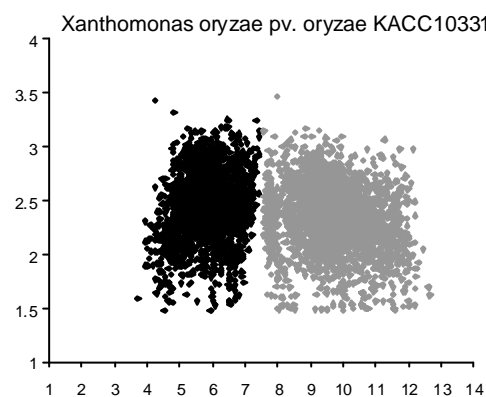

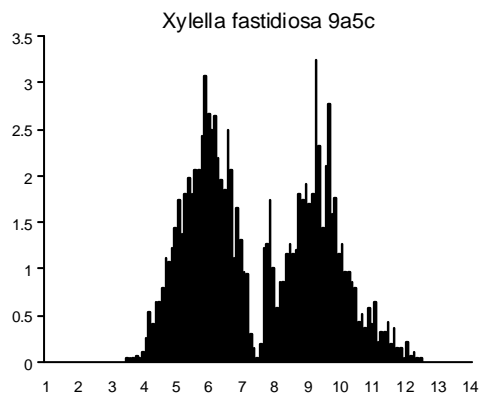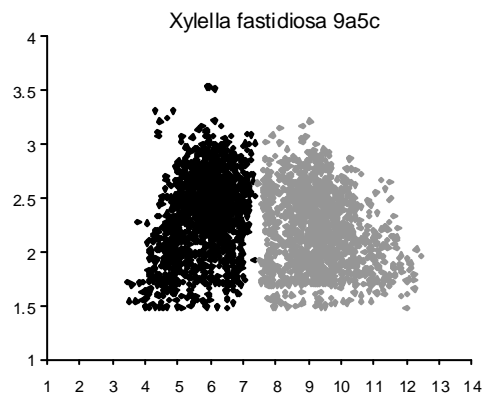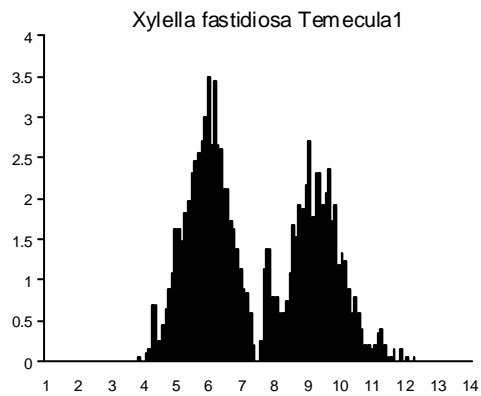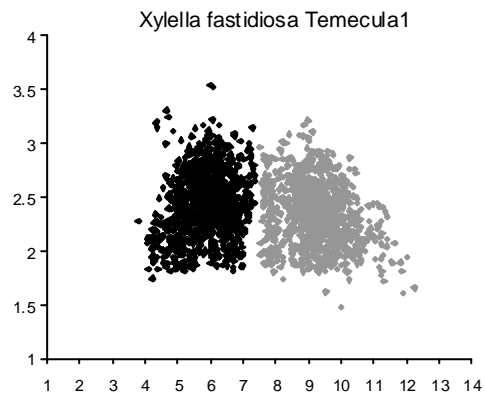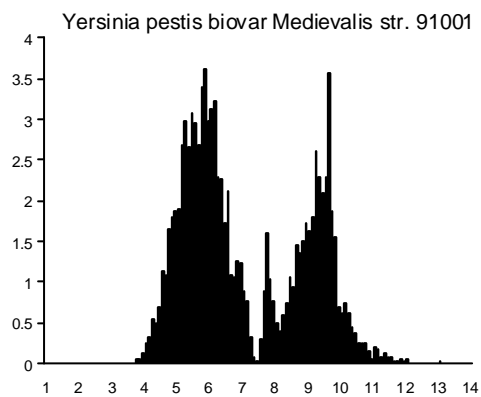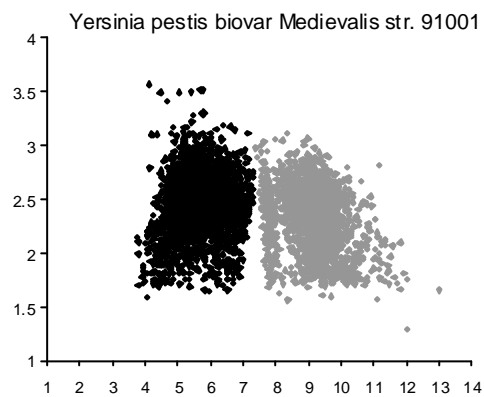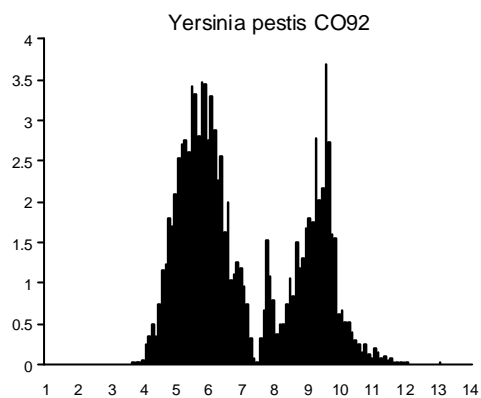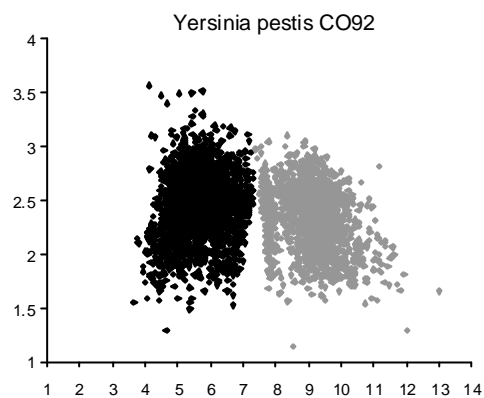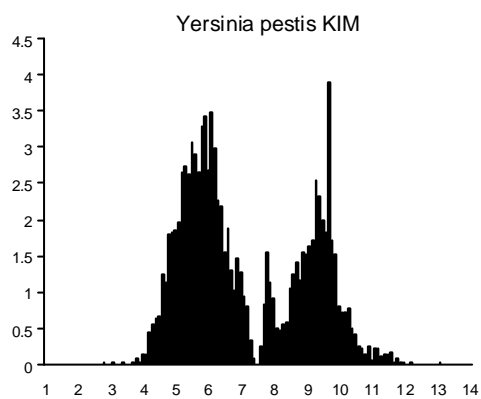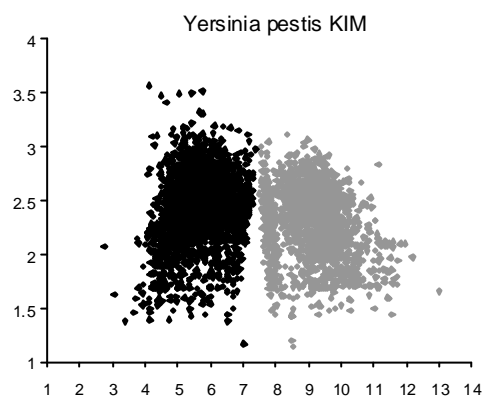

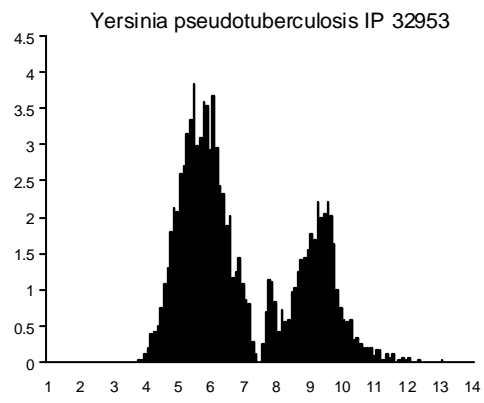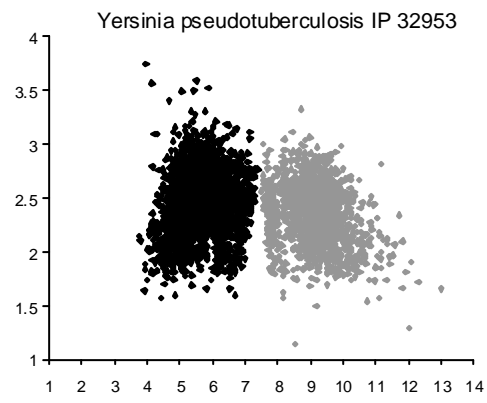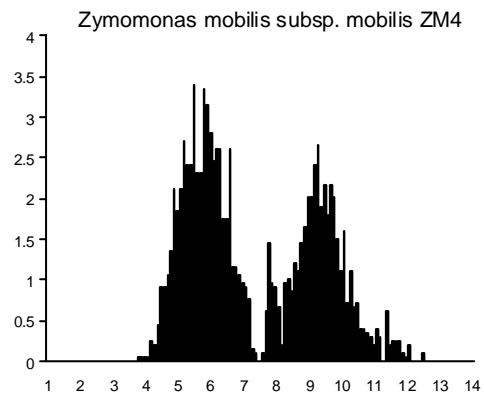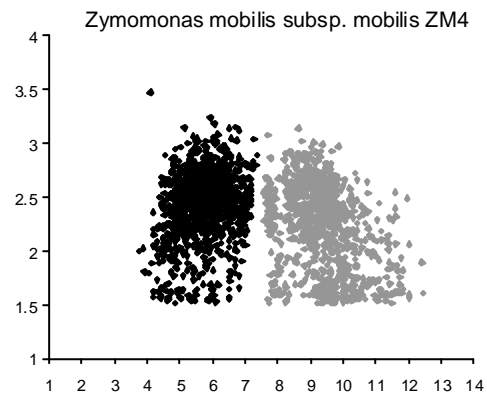

Supplement: Additional file 3 — PI distribution for prokaryotic proteomes. Left panel: histograms of pI values at 0.1 unit intervals (X axis: class of pI; Y axis: percent); right panel: relationships between the logarithm of length of proteins (Y axis) and their pI (Y axis). Black points represent the set of acidic proteins while grey ones – the set of basic proteins. [file 1471-2164-8-163-S3.pdf]

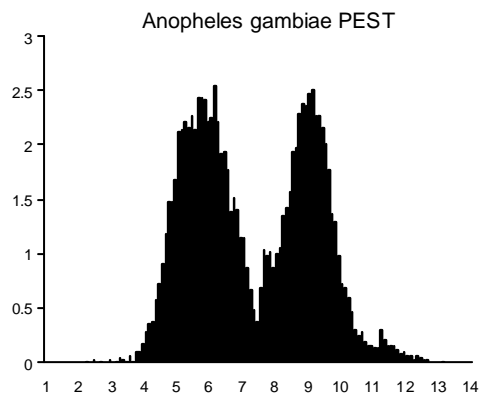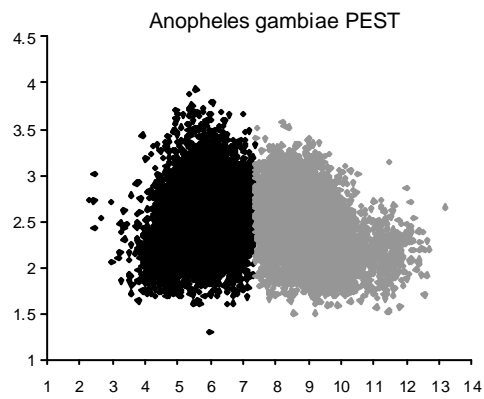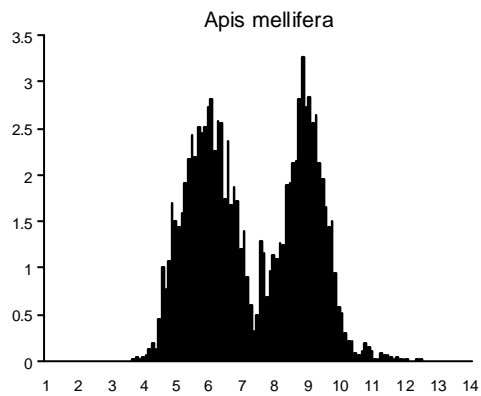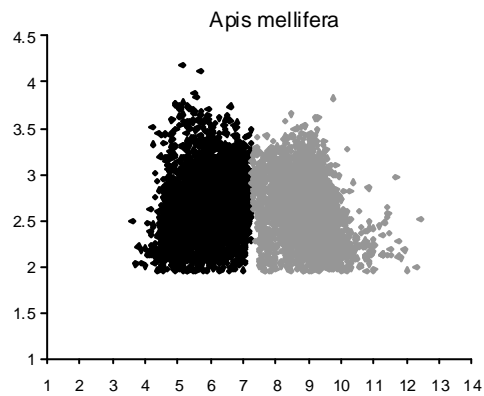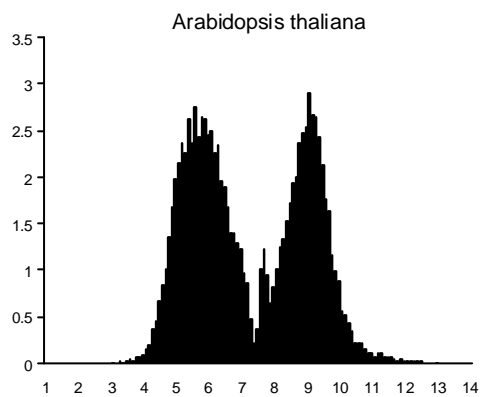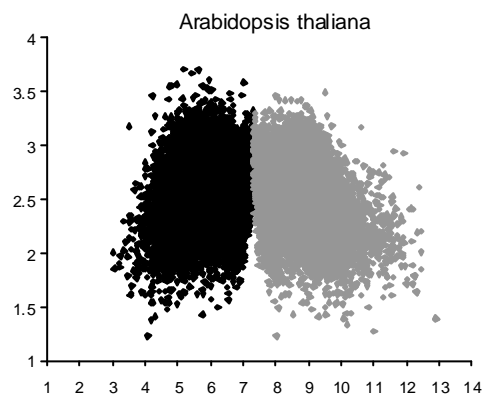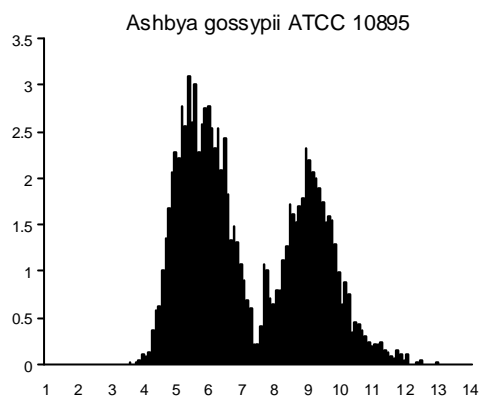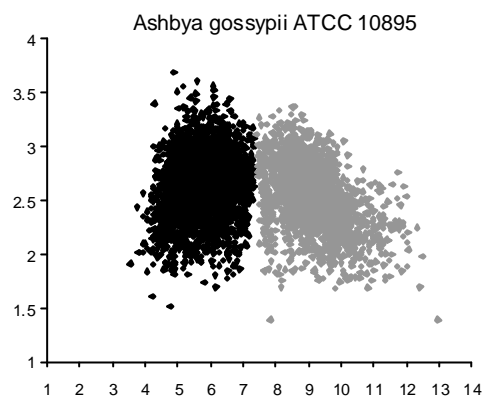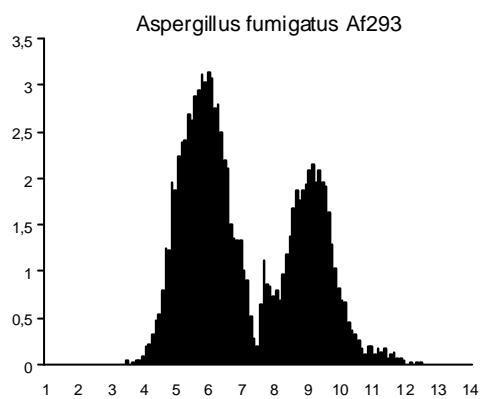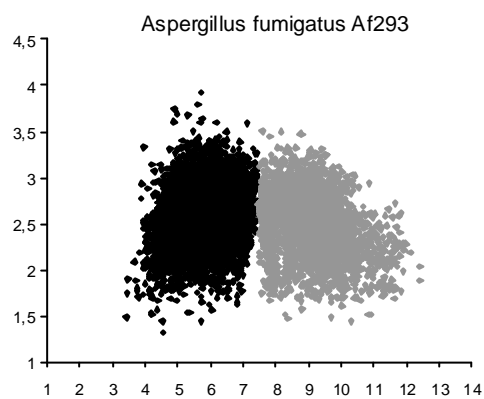

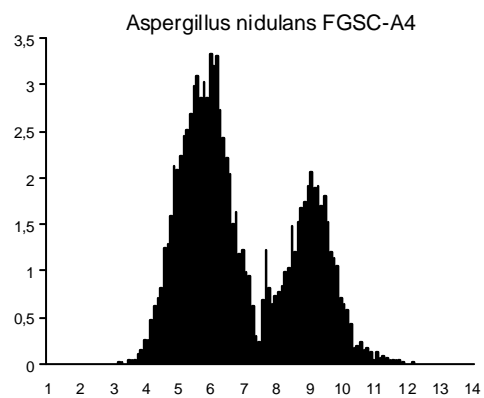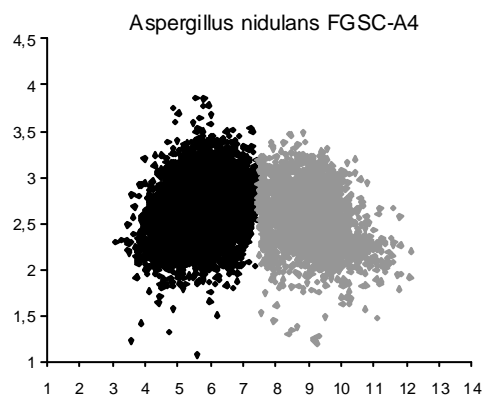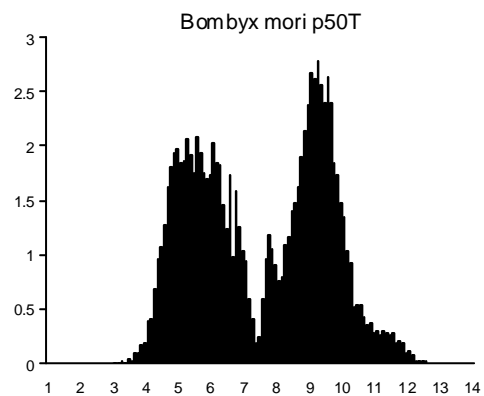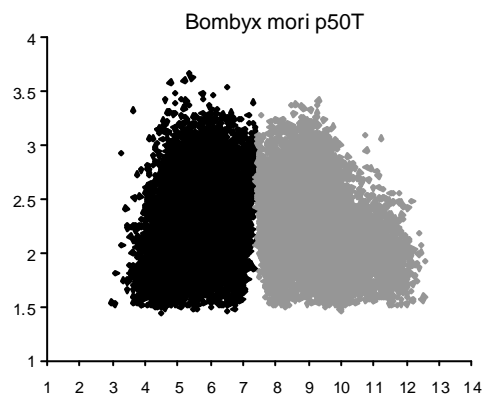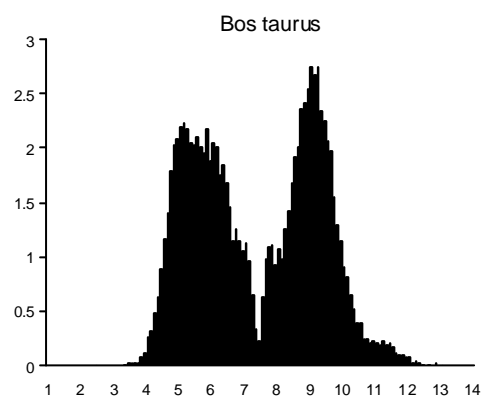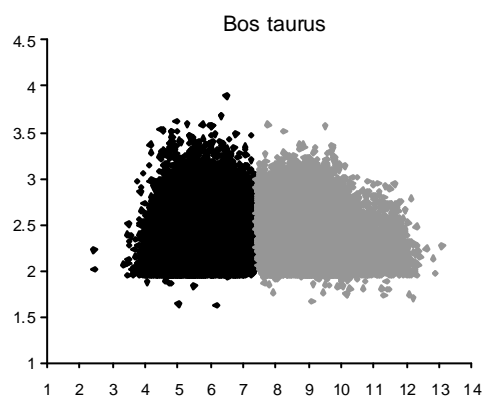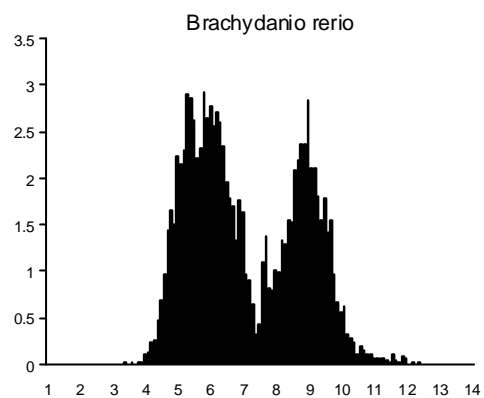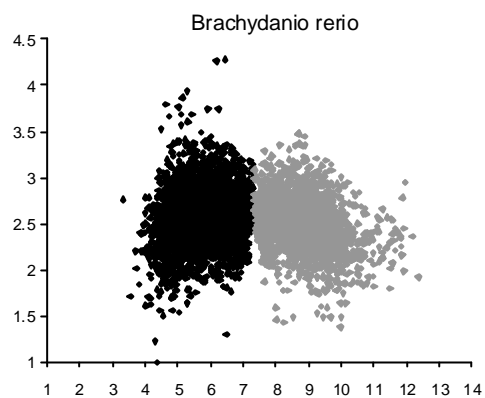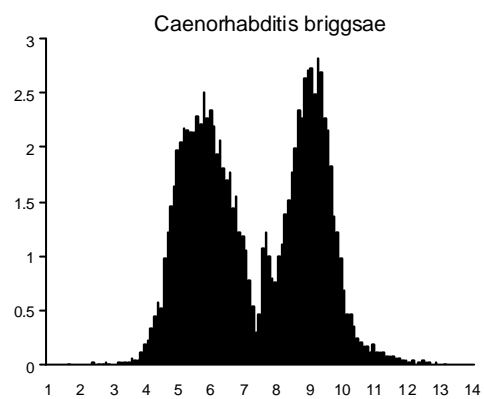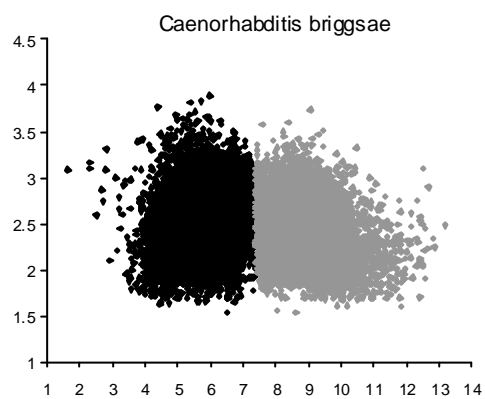

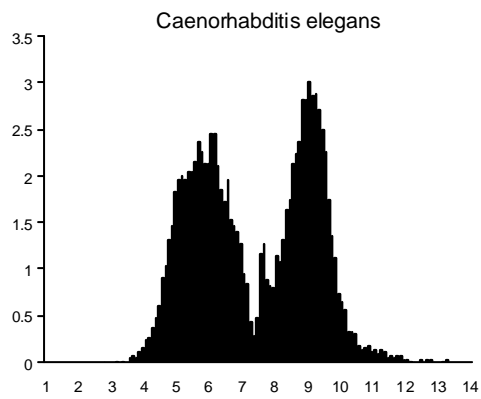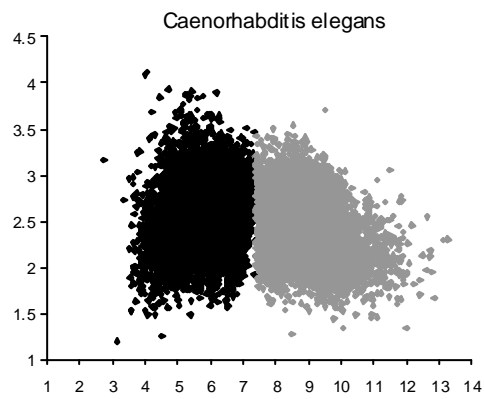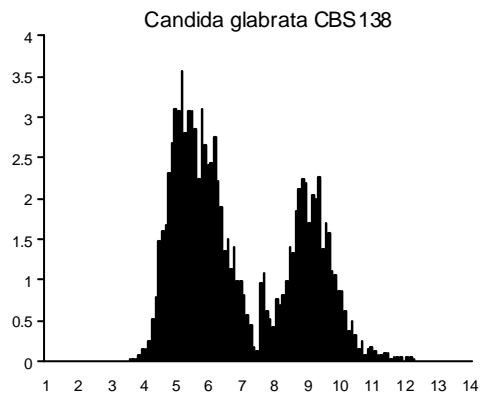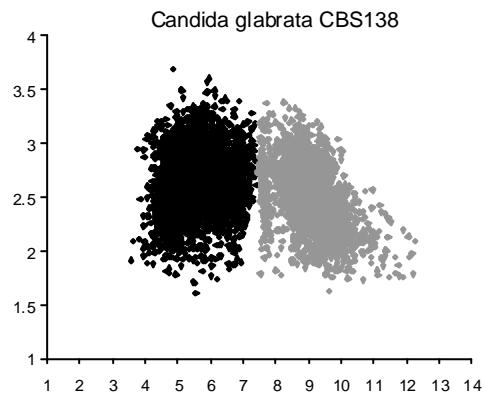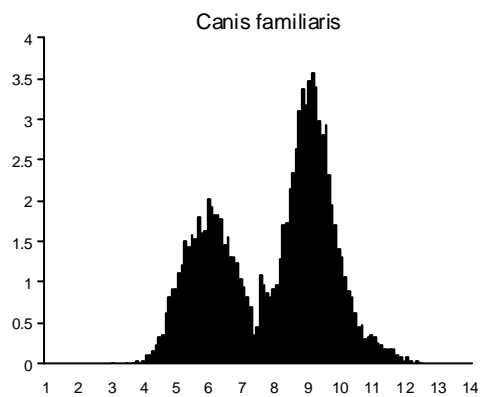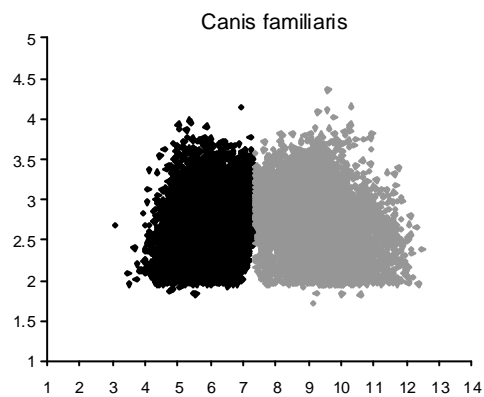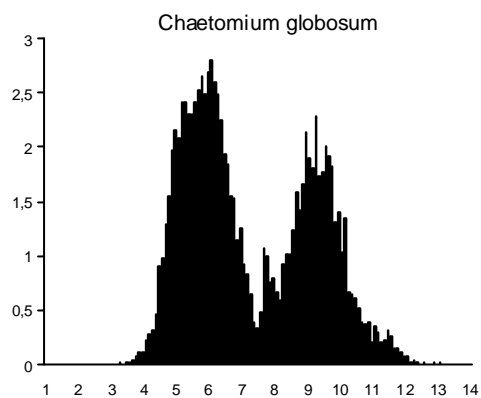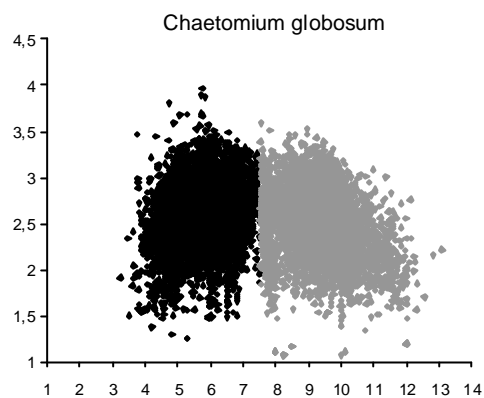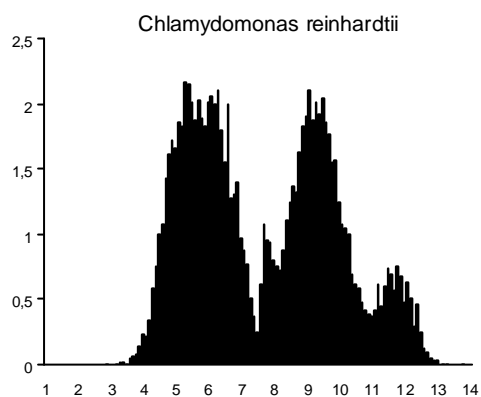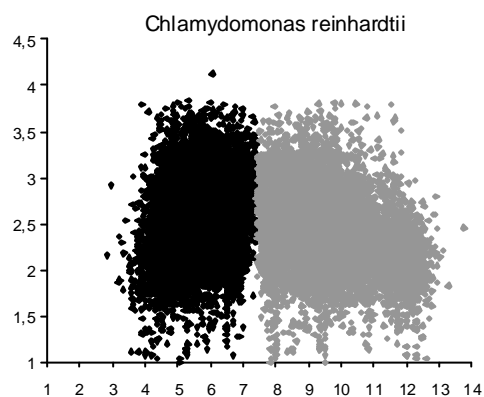

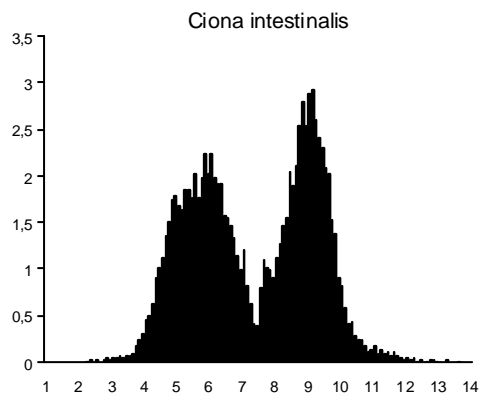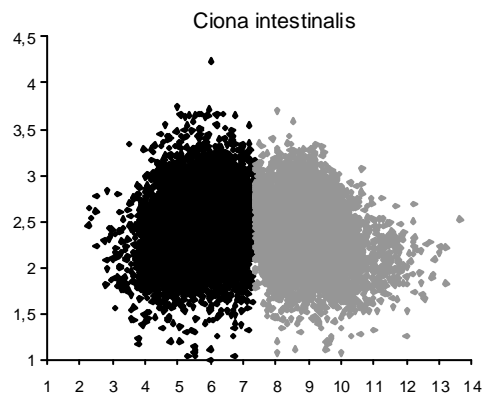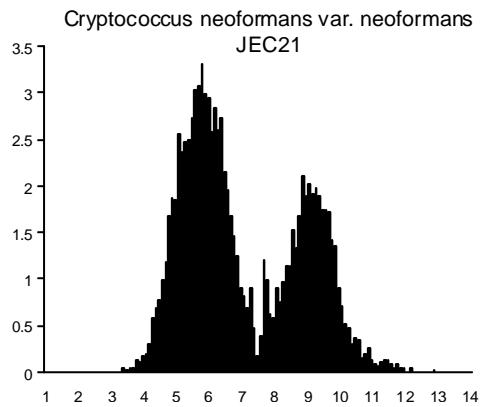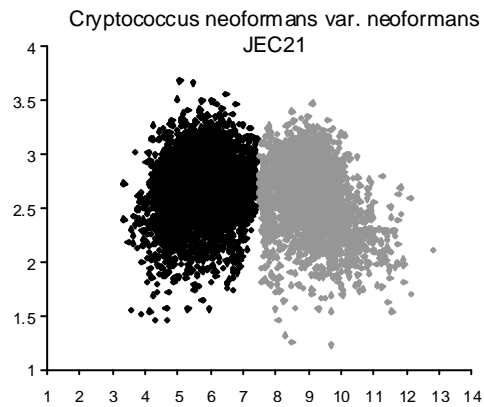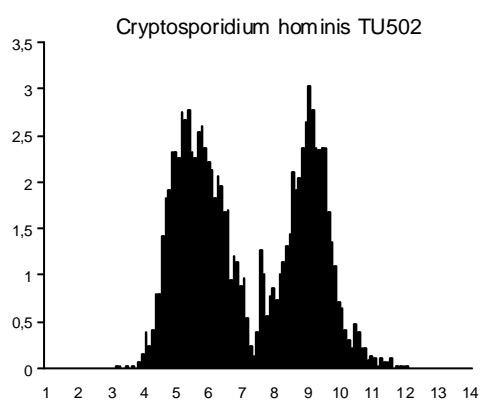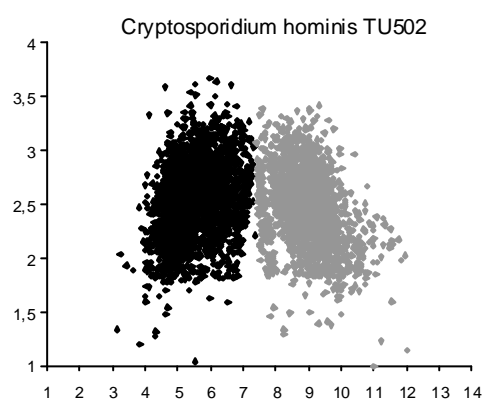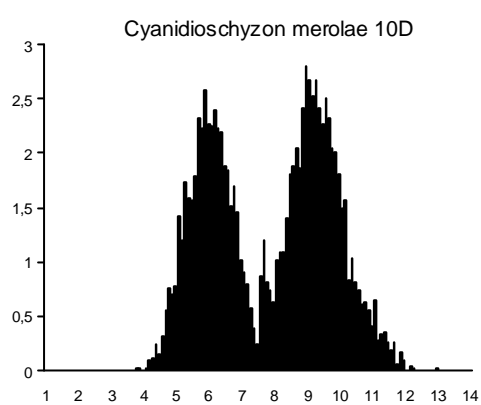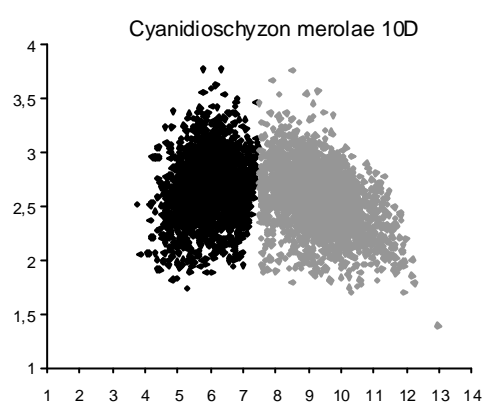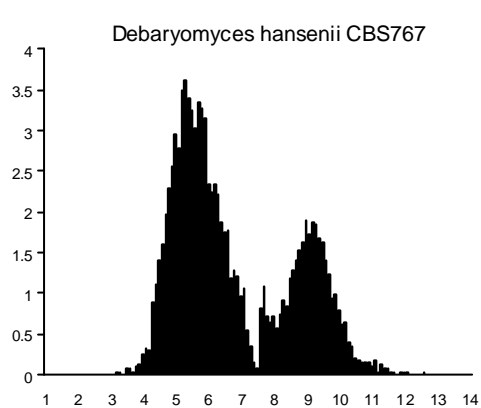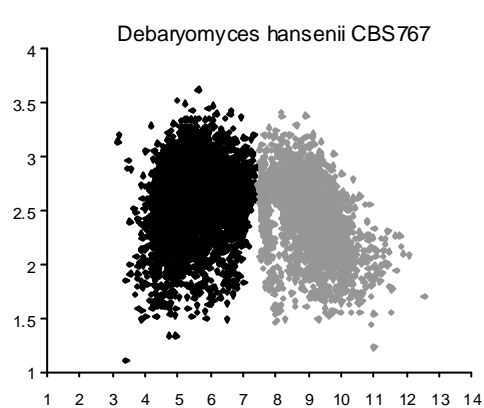

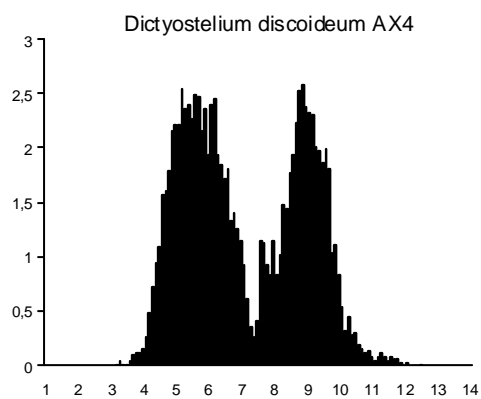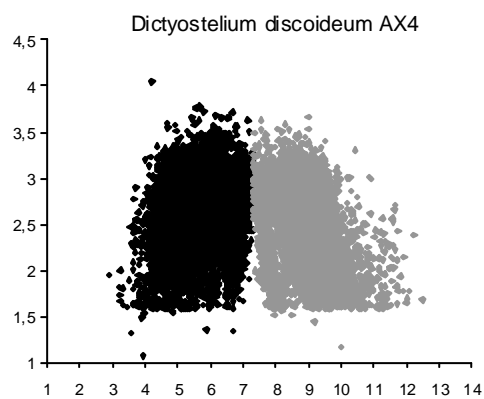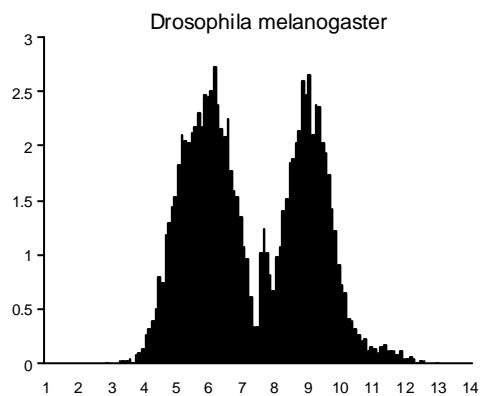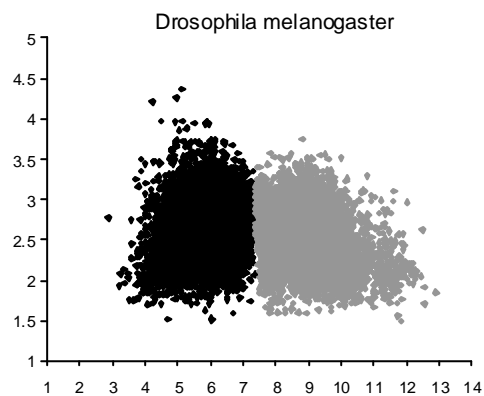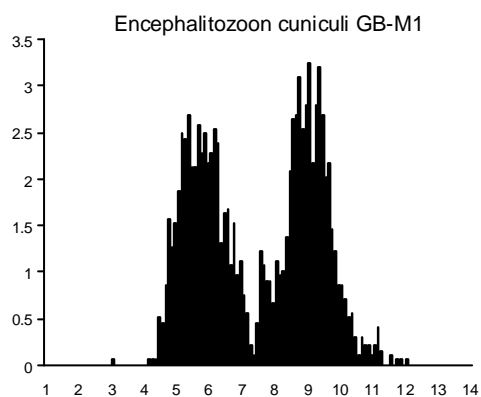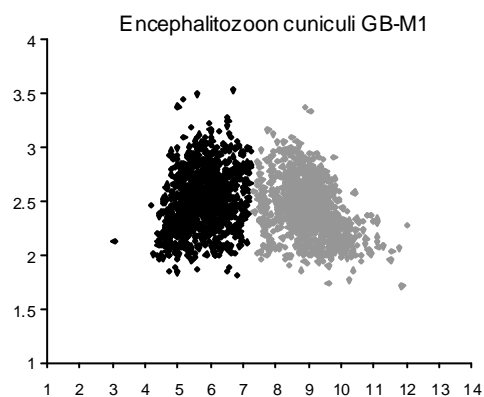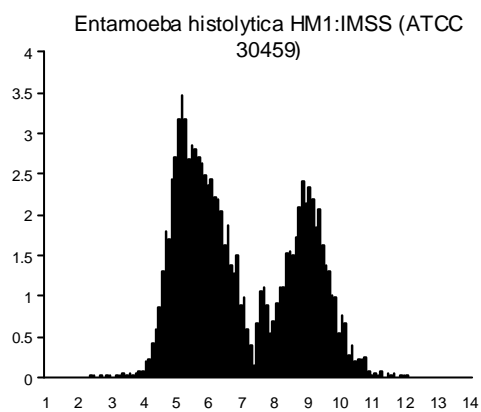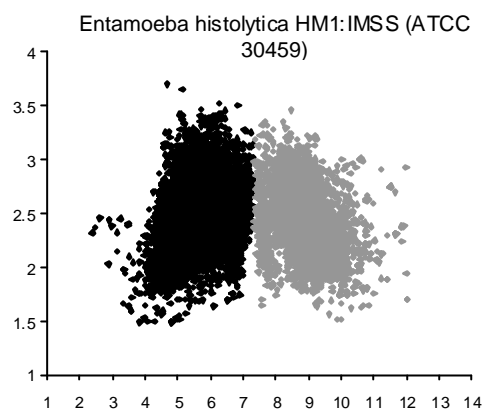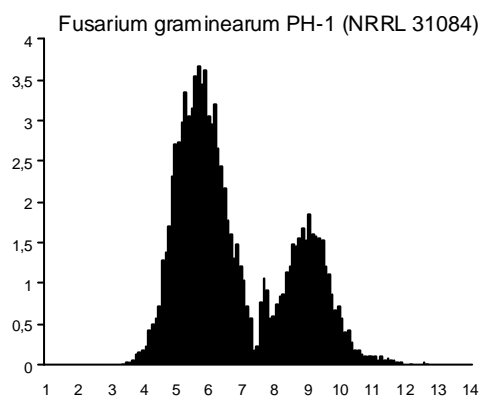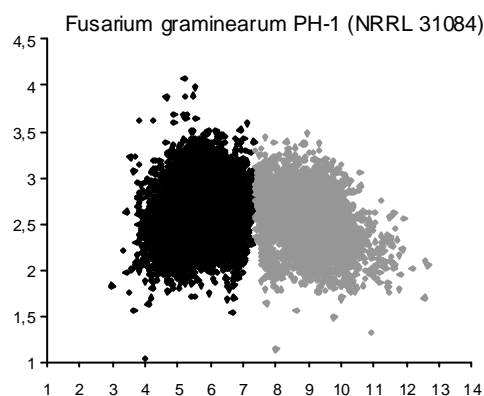

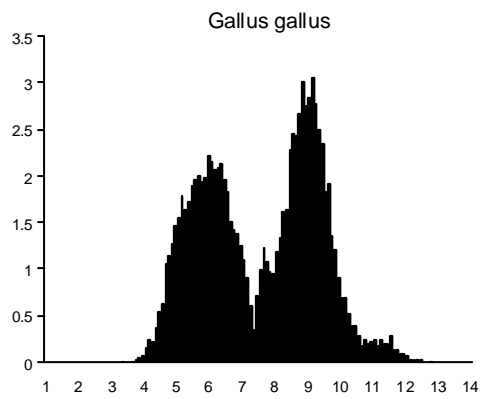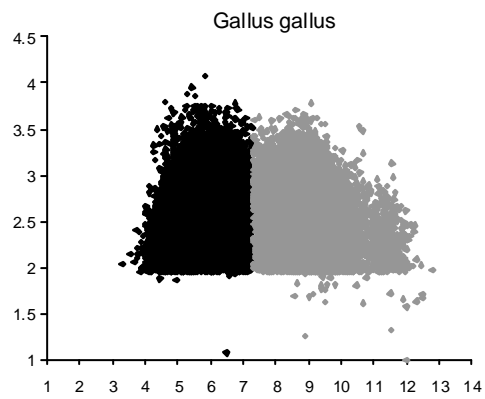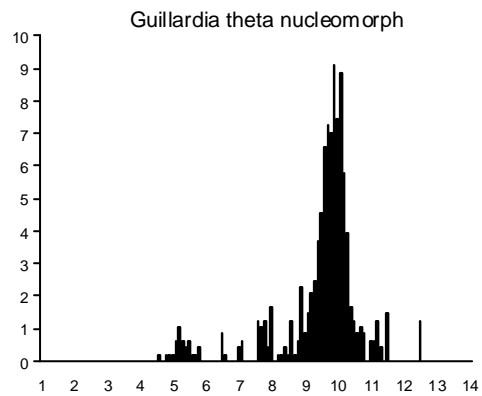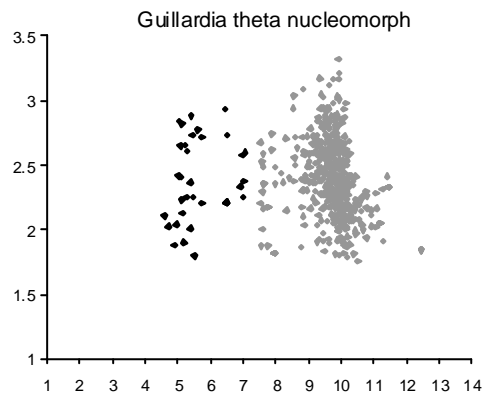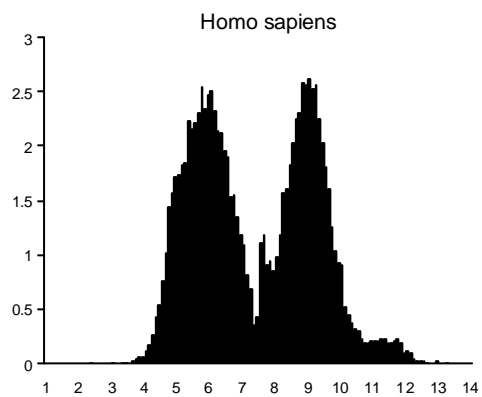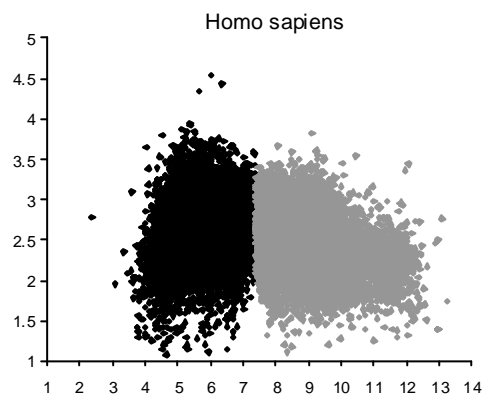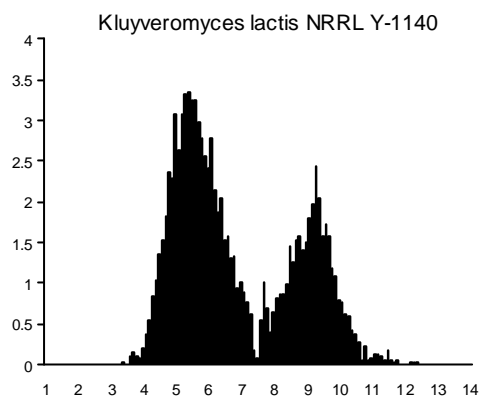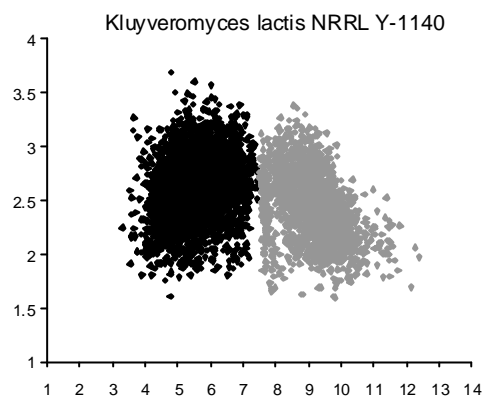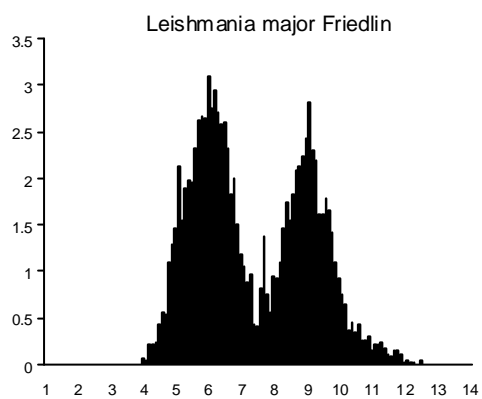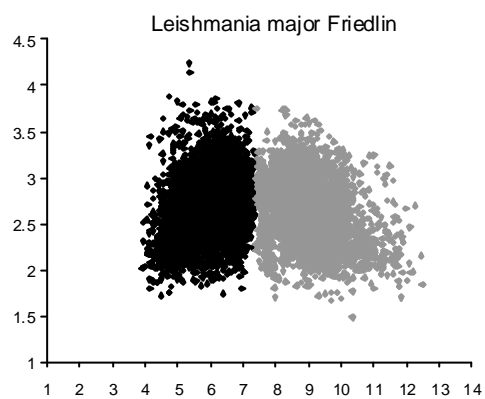

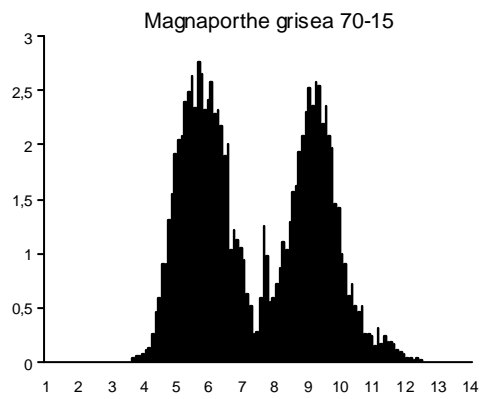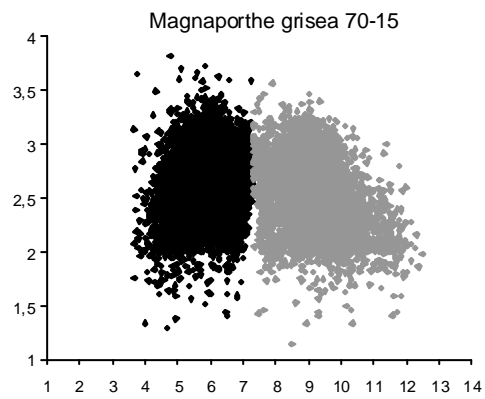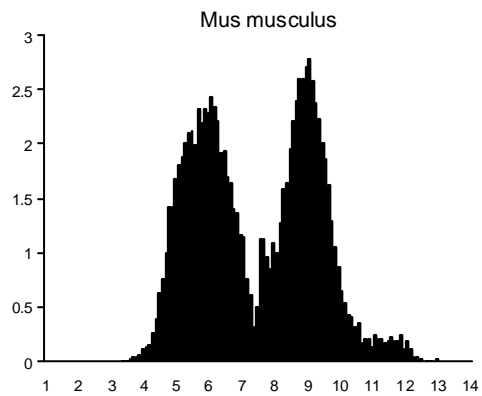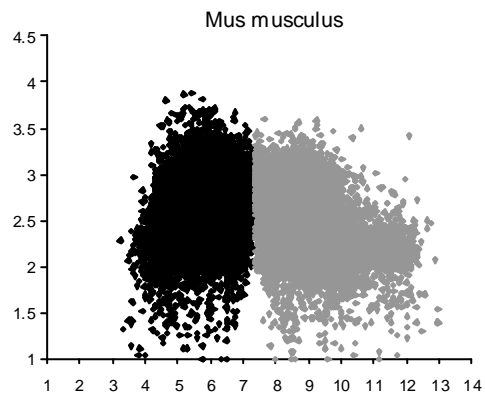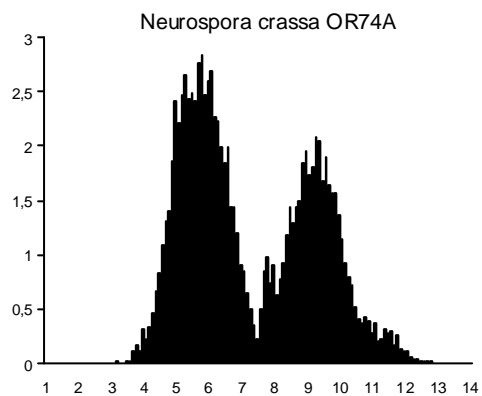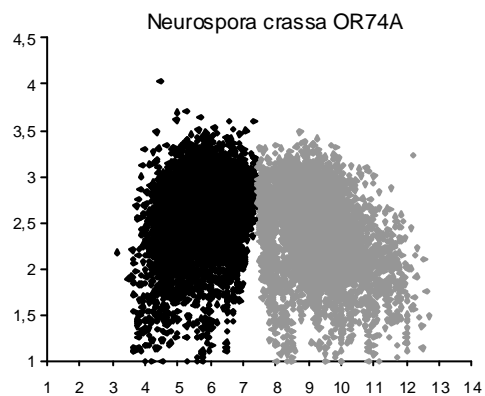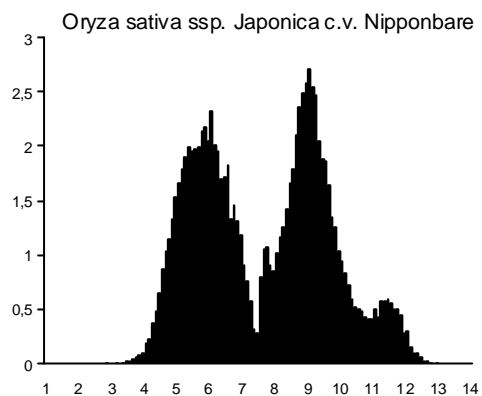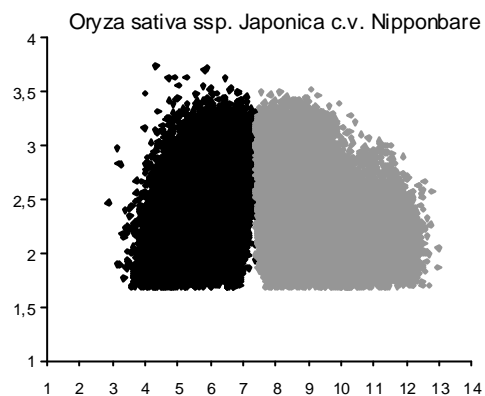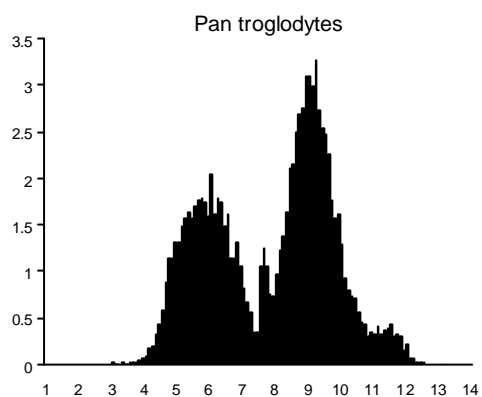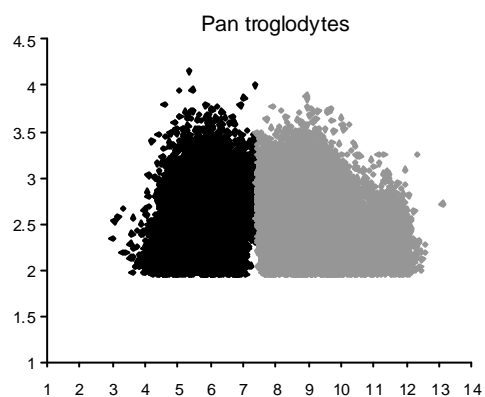

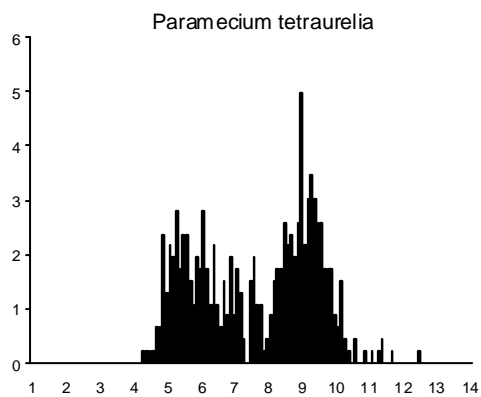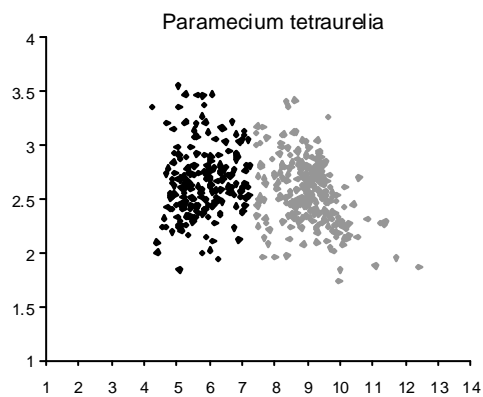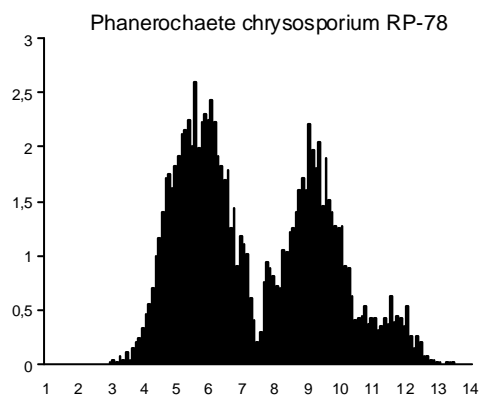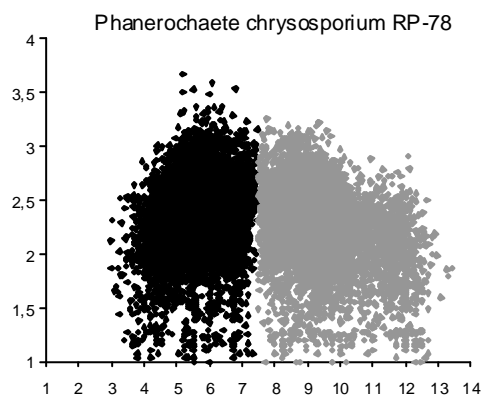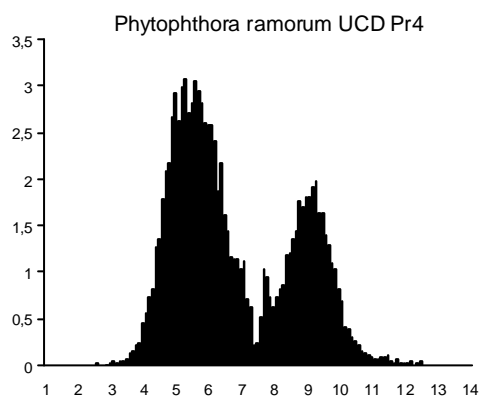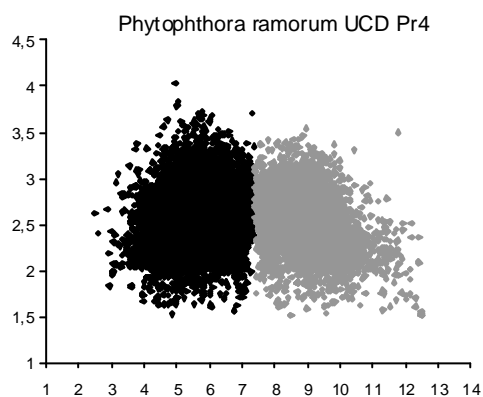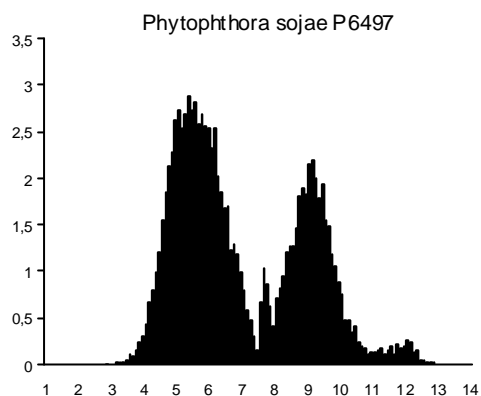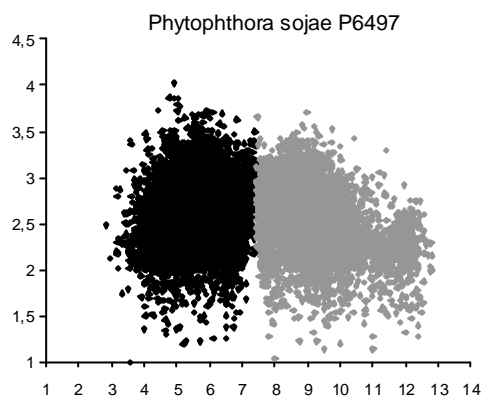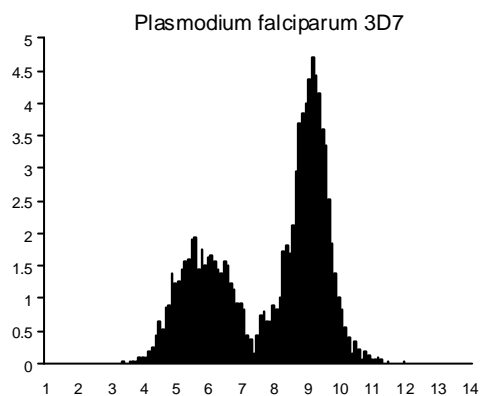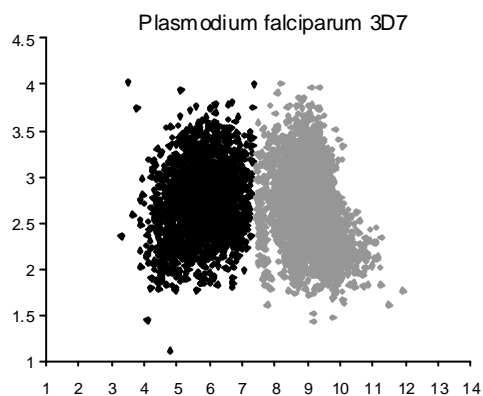

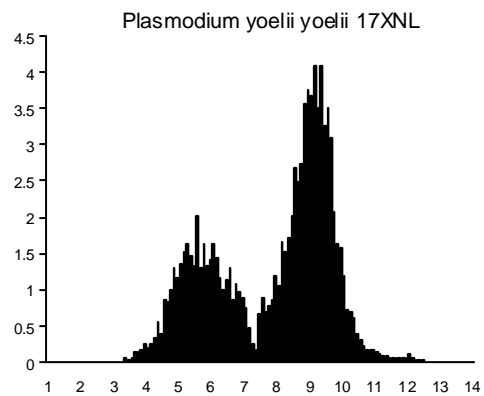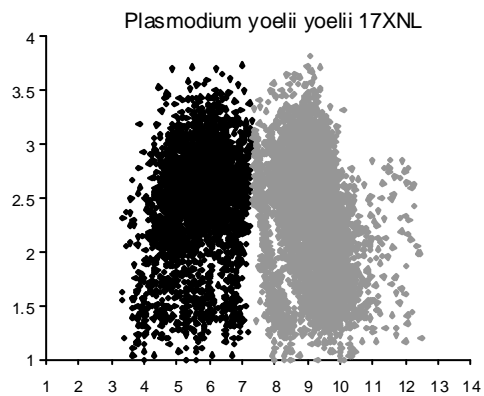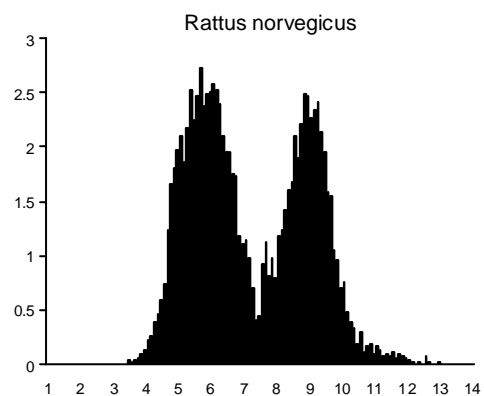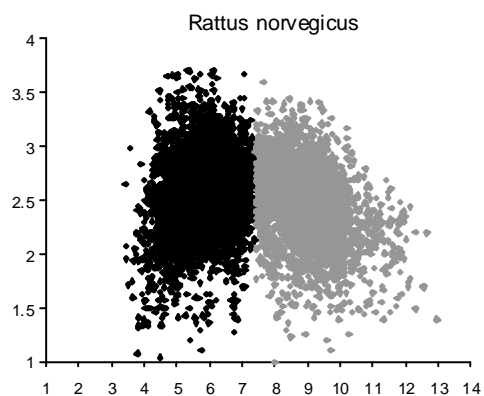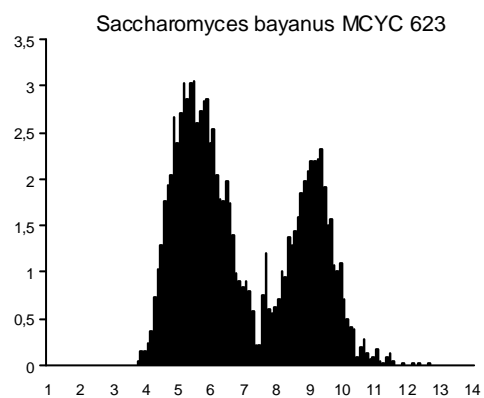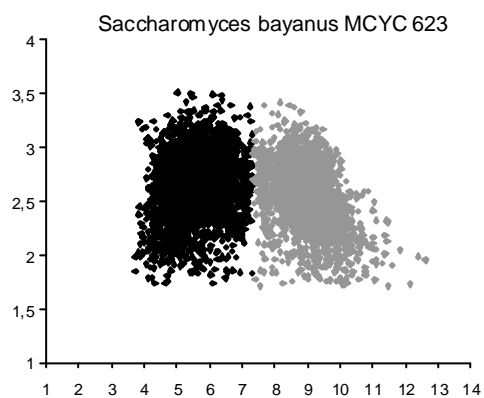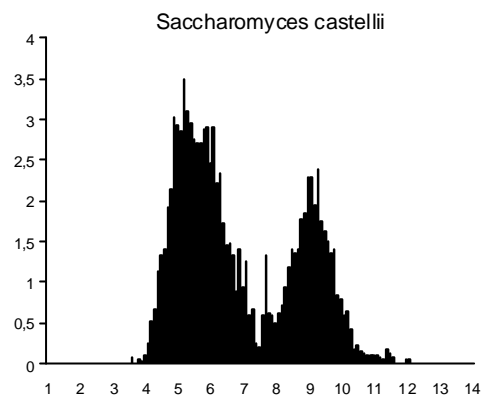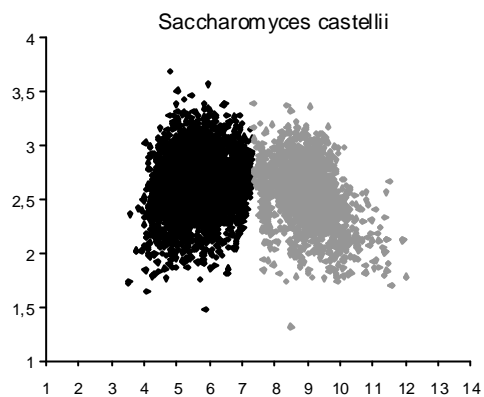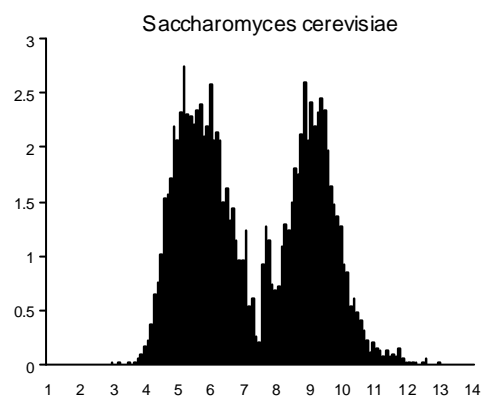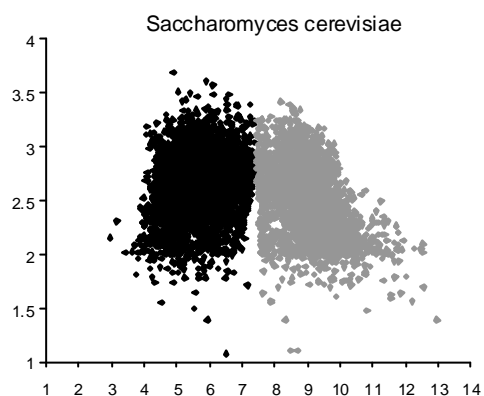

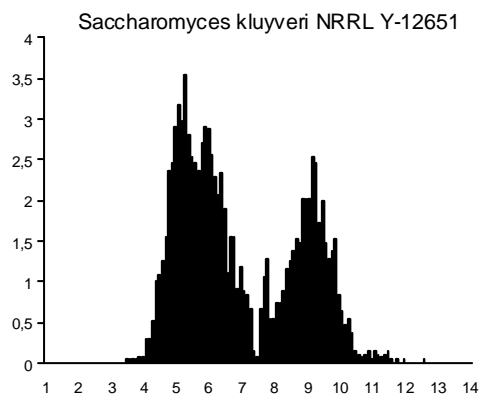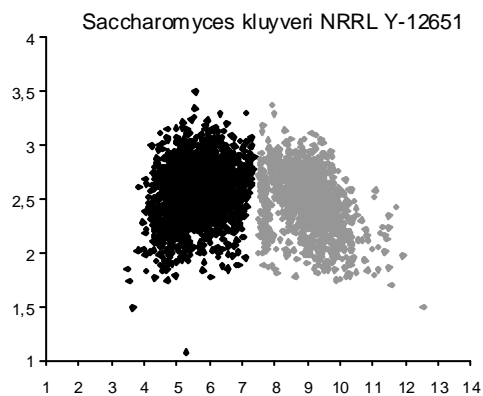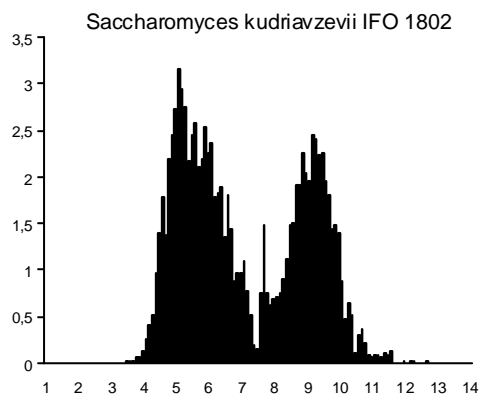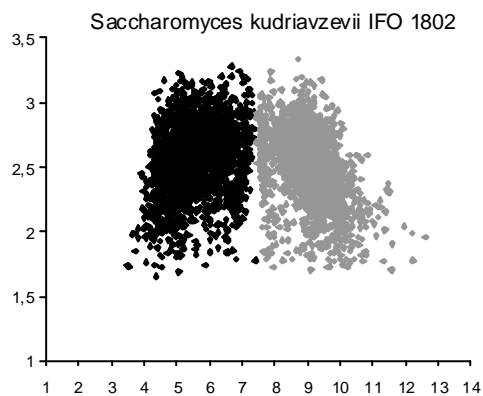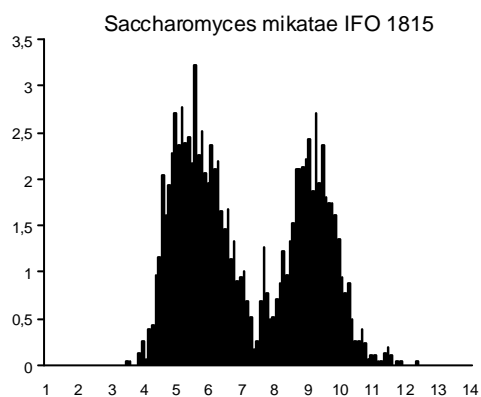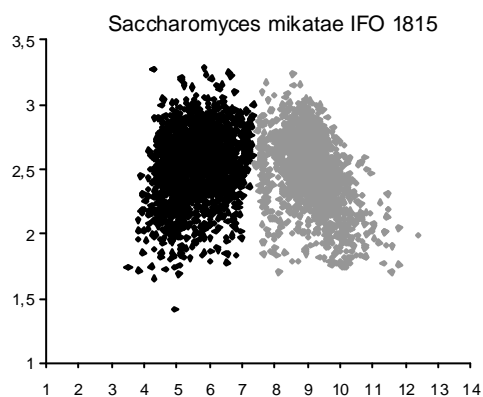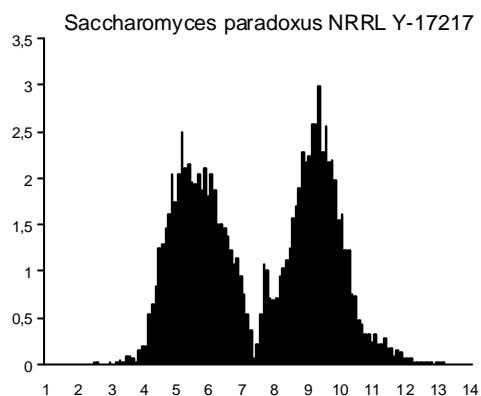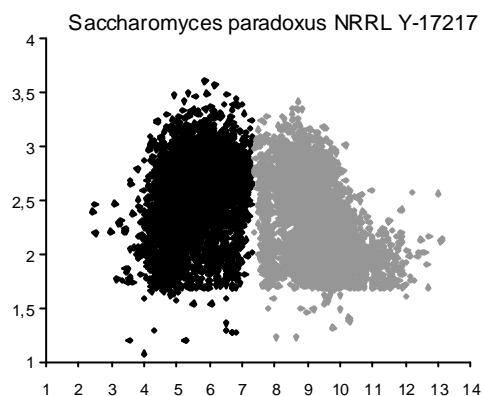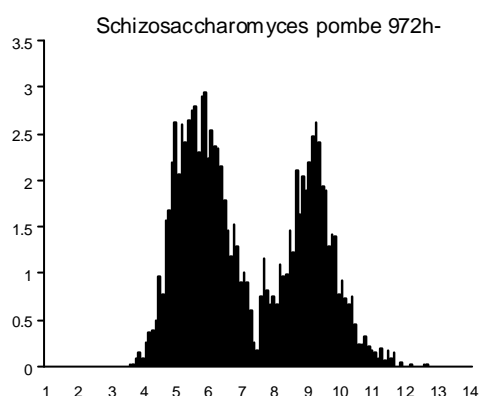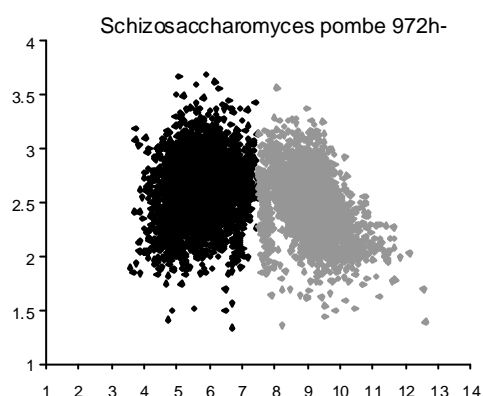

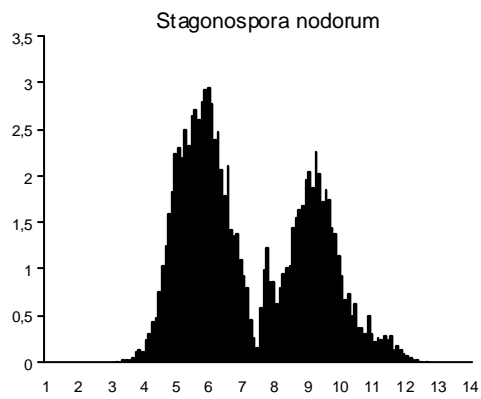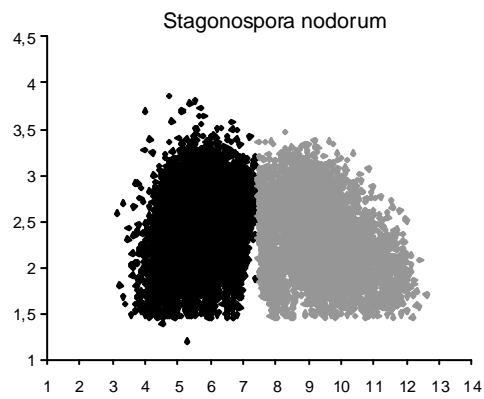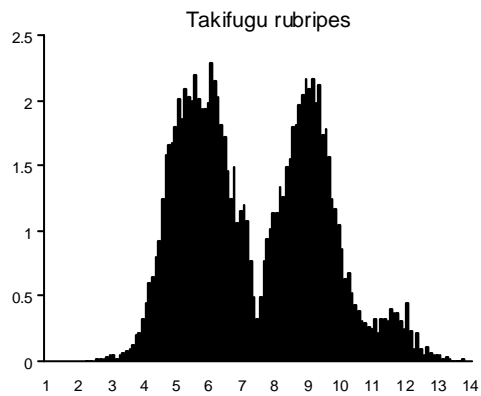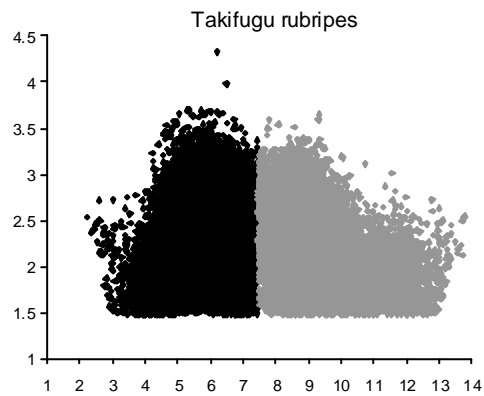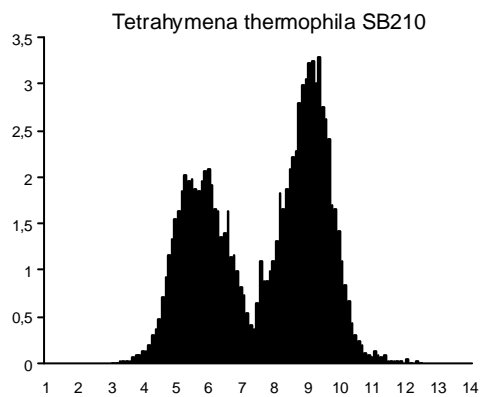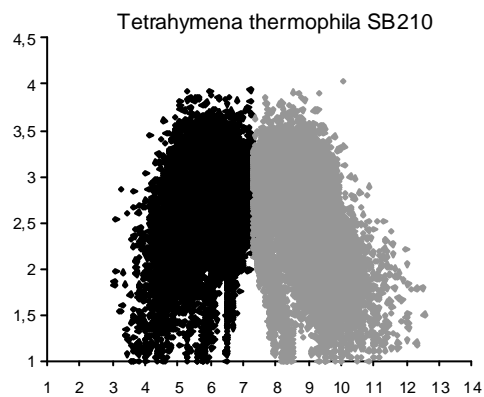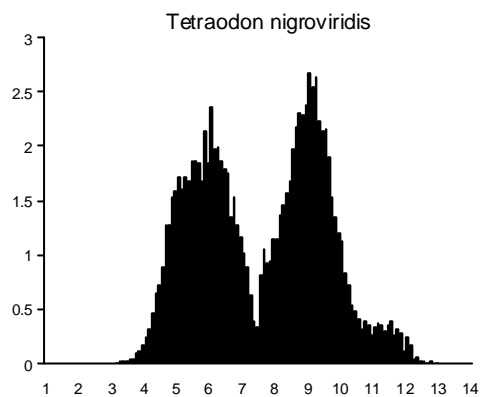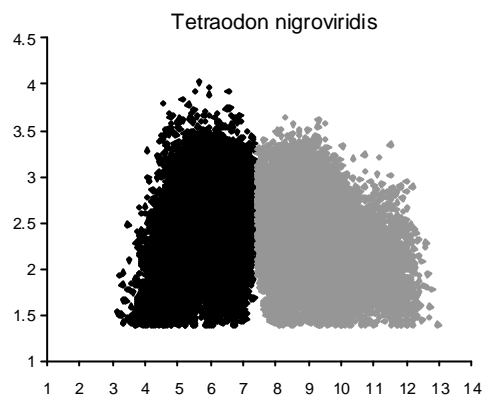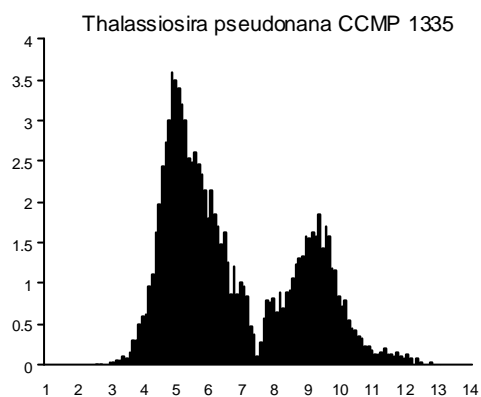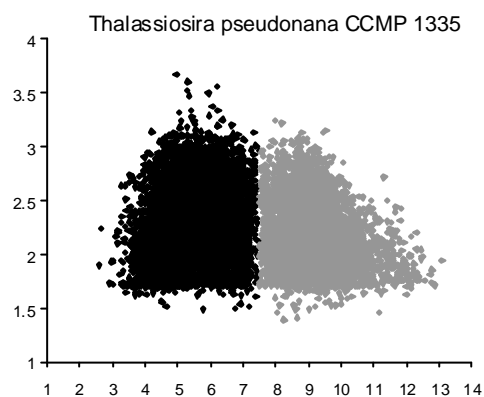

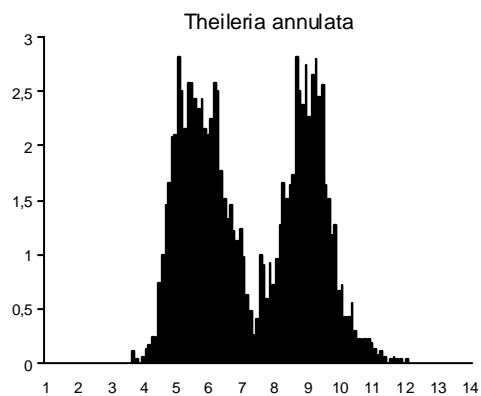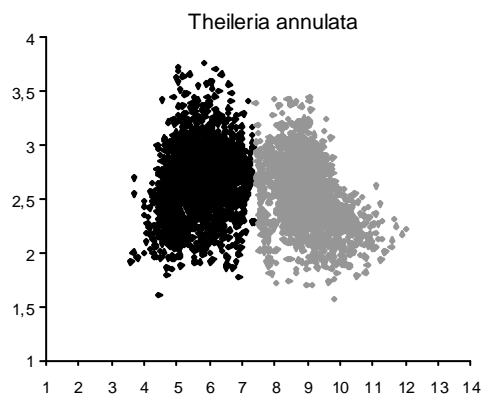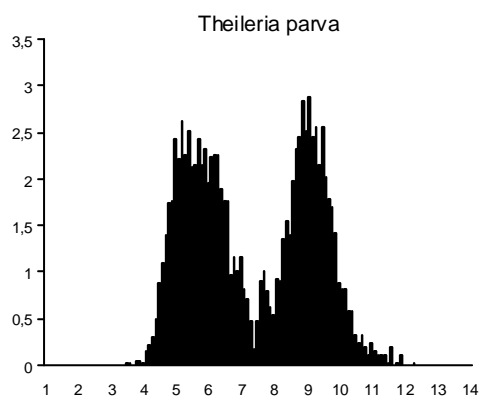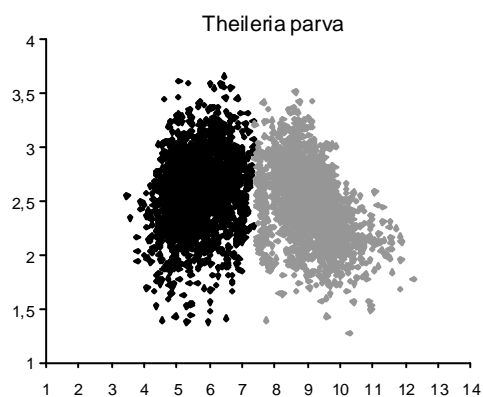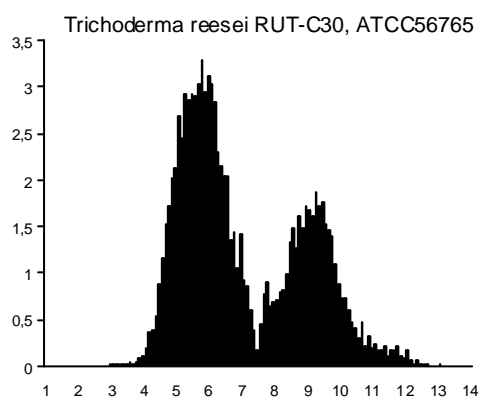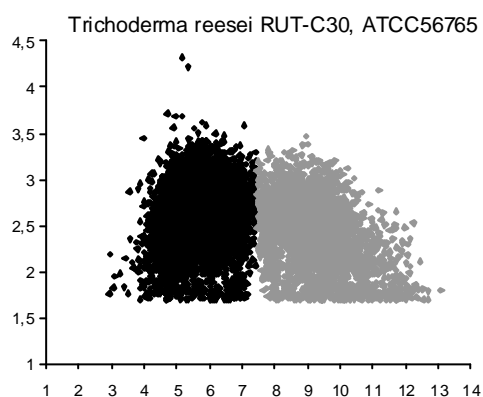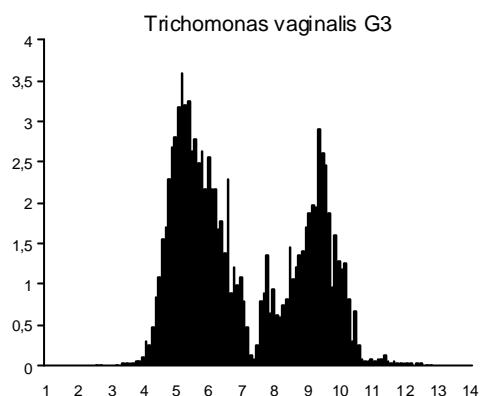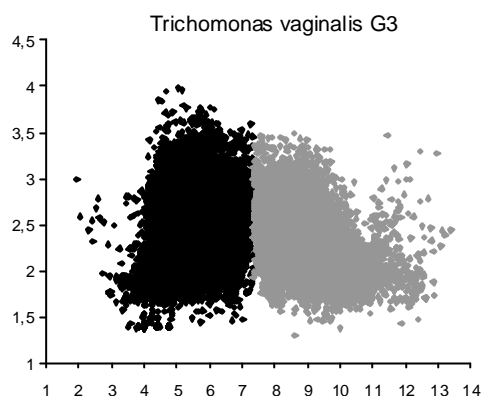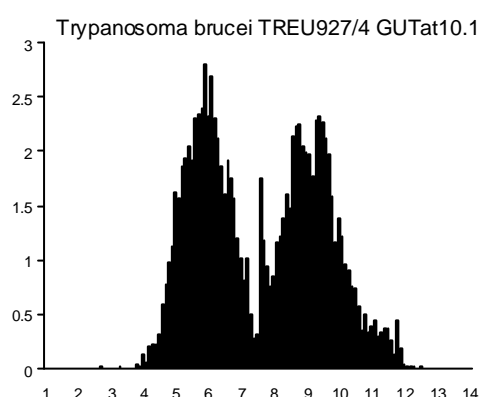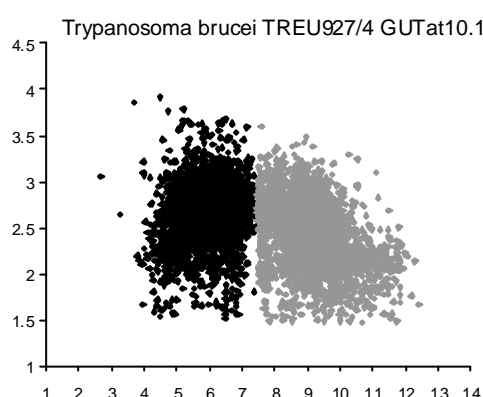

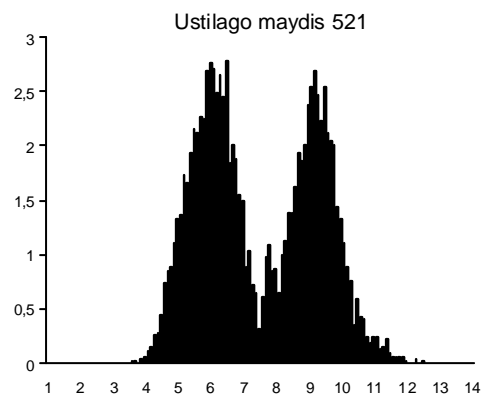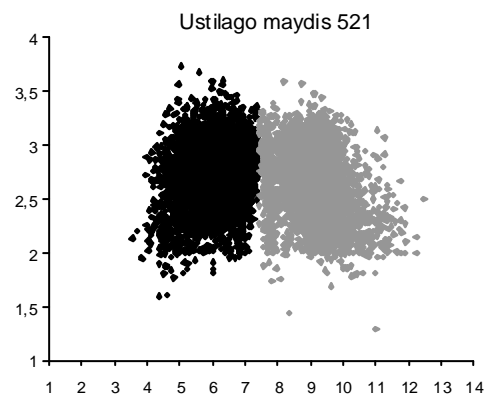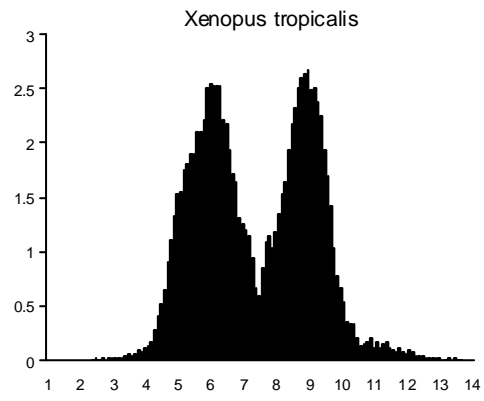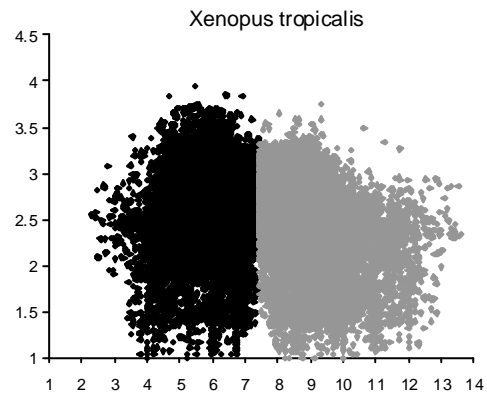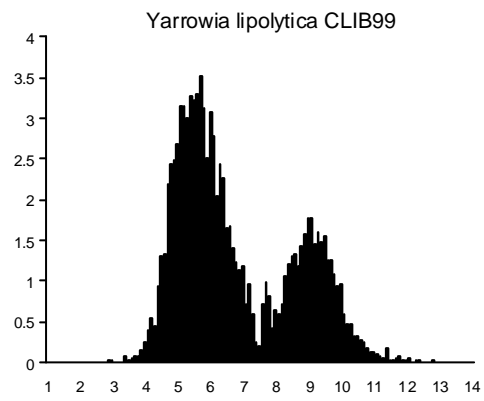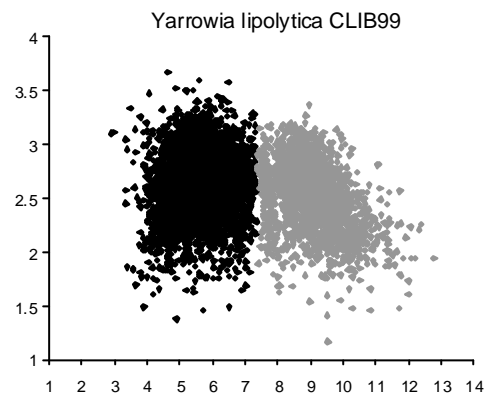

Supplement: Additional file 4 — PI distribution for eukaryotic proteomes. Left panel: histograms of pI values at 0.1 unit intervals (X axis: class of pI; Y axis: percent); right panel: relationships between the logarithm of length of proteins (Y axis) and their pI (Y axis). Black points represent the set of acidic proteins while grey ones – the set of basic proteins. [file 1471-2164-8-163-S4.pdf]

**A**

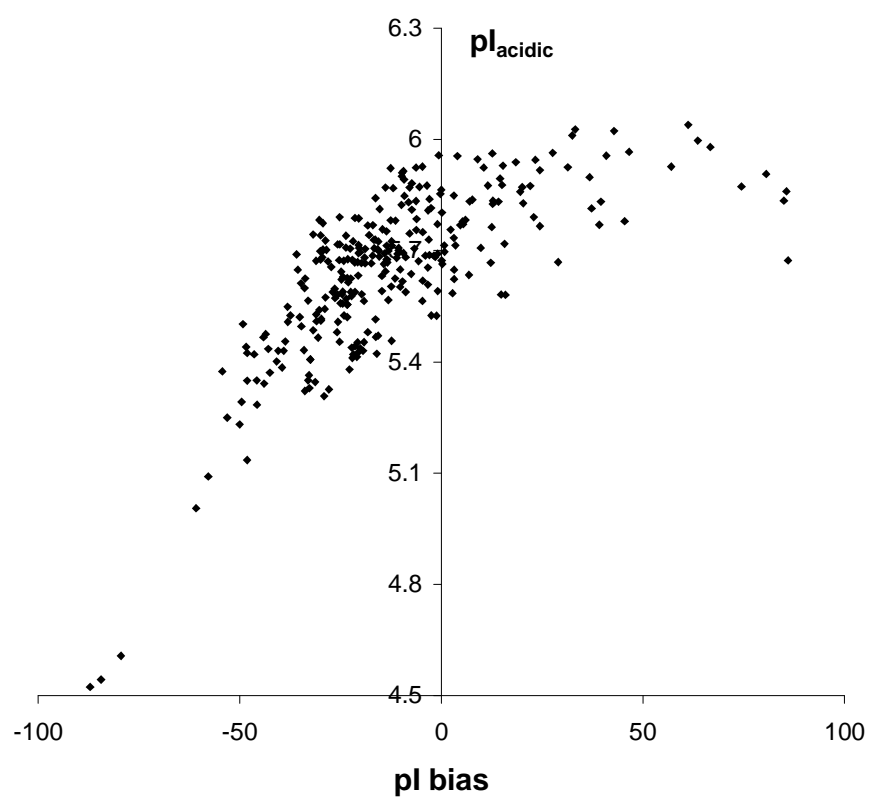

**B**

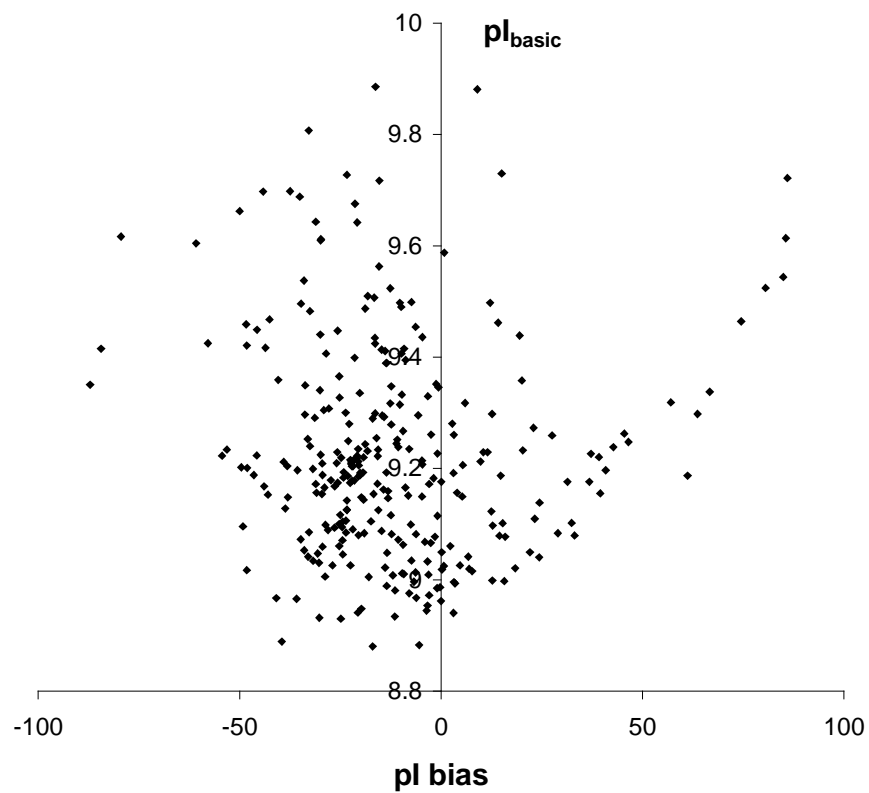

Supplement: Additional file 5 — Relationship between the average pI of proteins and the pI bias for acidic (A) and basic sets (B). [file 1471-2164-8-163-S5.pdf]

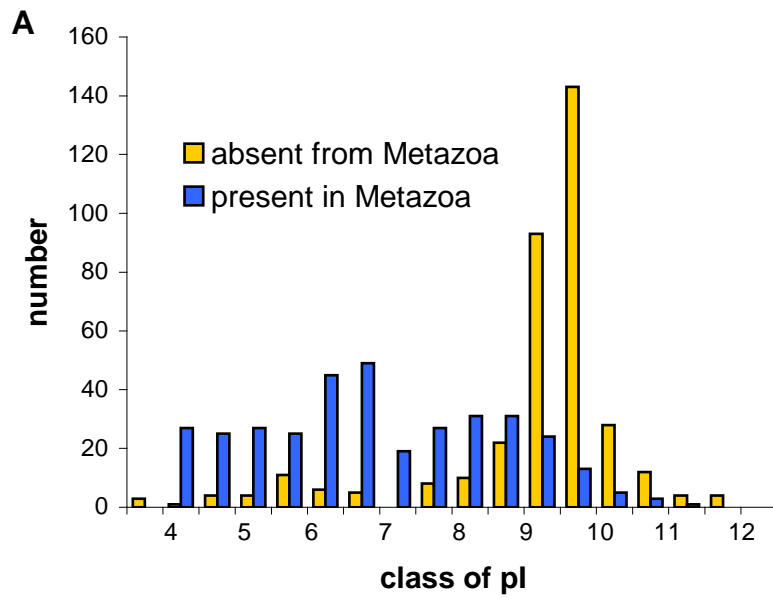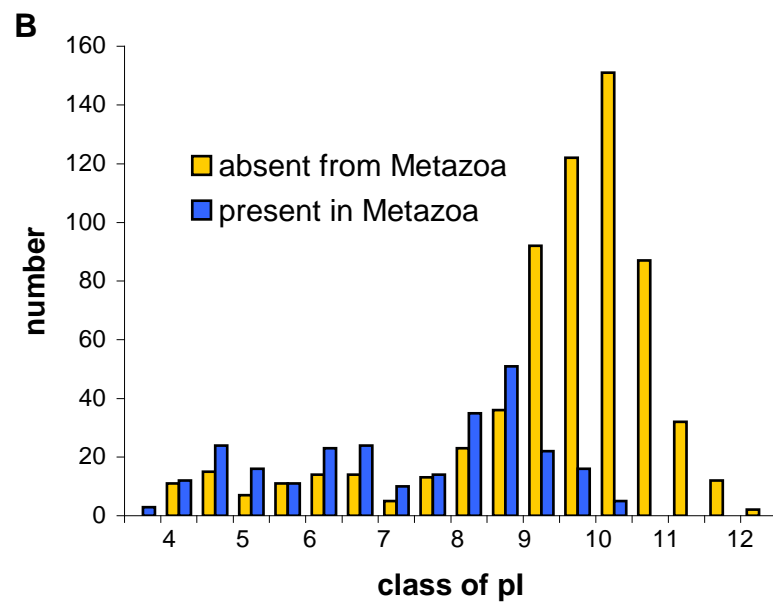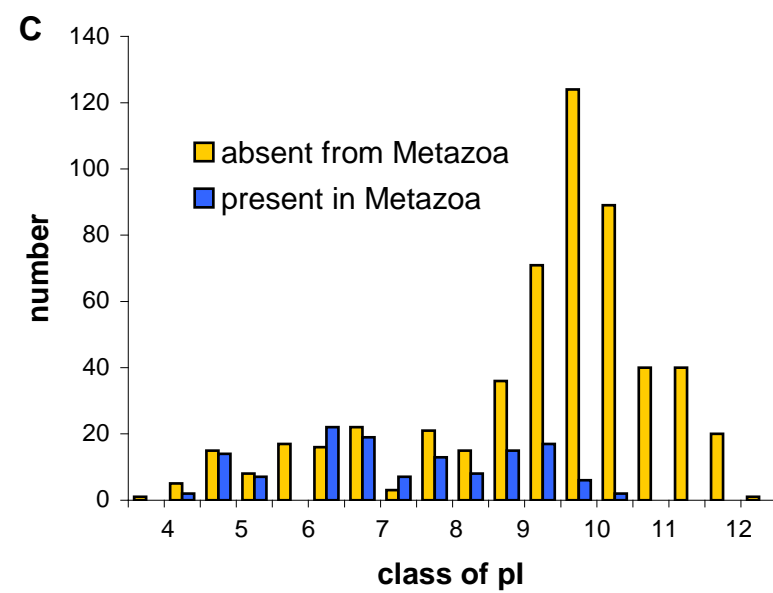

Supplement: Additional file 7 — PI distribution of mitochondrial proteomes belonging to Fungi (A), Protista (B) and Viridiplantae (C) prepared separately for proteins common for Metazoa and the analyzed group (present in Metazoa) and absent from Metazoa. [file 1471-2164-8-163-S7.pdf]

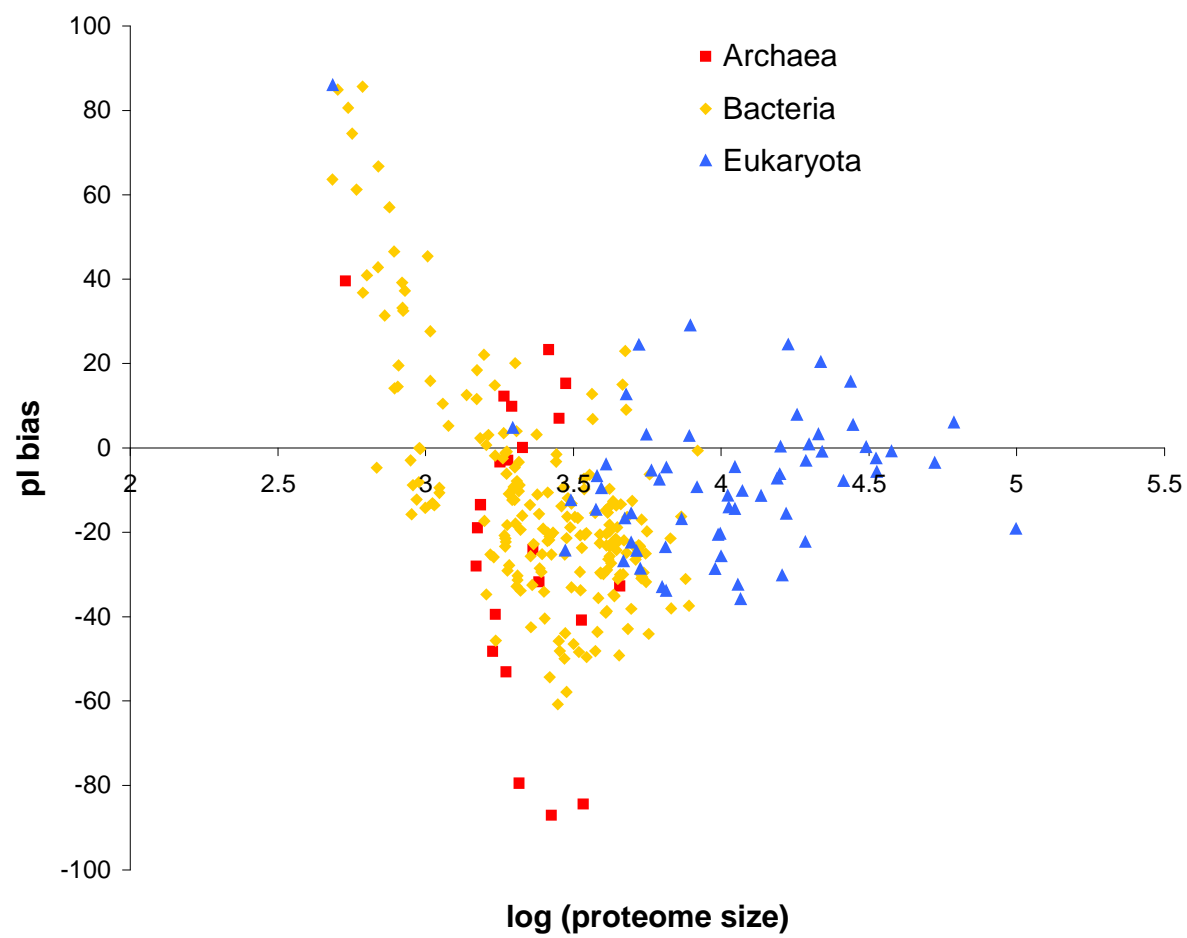

Supplement: Additional file 11 — Relationship between the pI bias and logarithm of proteome size for archaeal, bacterial and eukaryotic organisms. [file 1471-2164-8-163-S11.pdf]
