# Supplementary material for: The relationships between the isoelectric point and: length of proteins, taxonomy and ecology of organisms
Source: BMC Genomics. 2007 Jun 12;8:163. doi: 10.1186/1471-2164-8-163 (PMC1905920; doi:10.1186/1471-2164-8-163)

The real proteome of *Escherichia coli* K12

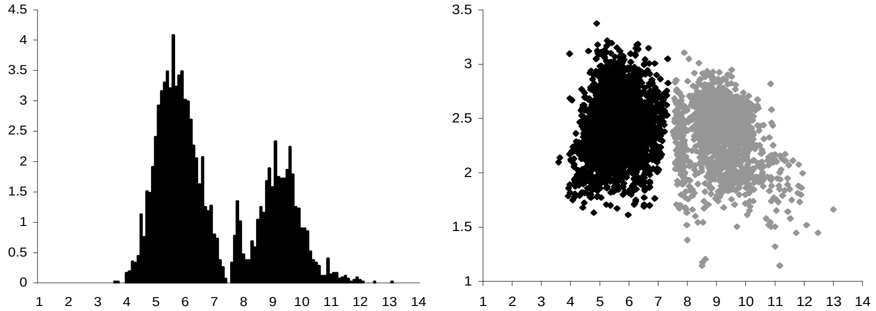

Artificial proteome: average amino acid composition of *E. coli* proteins; length of *E. coli* proteins

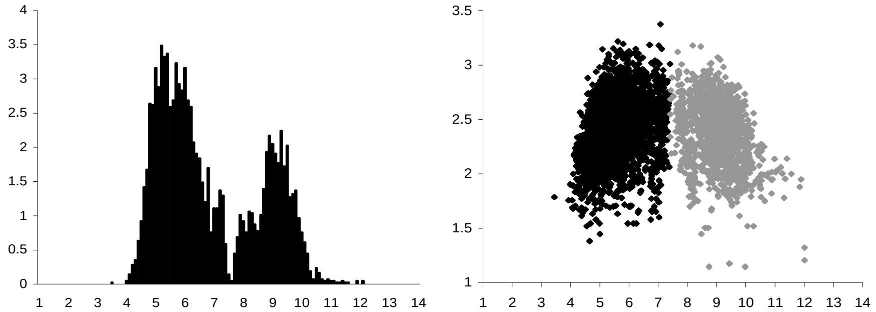

Artificial proteome: equal frequencies of amino acids; length of *E. coli* proteins

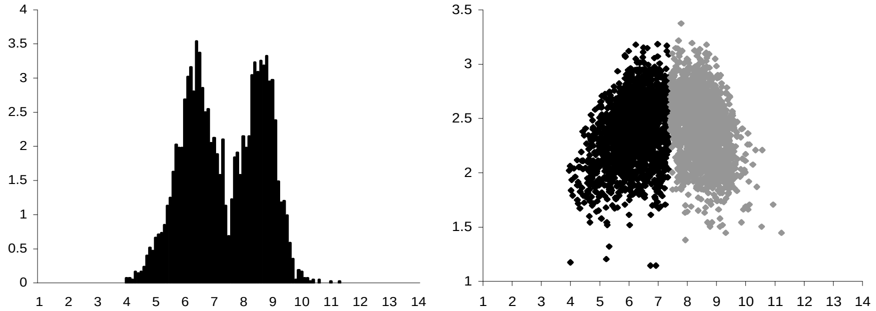

Artificial proteome: average amino acid composition of *E. coli* proteins; uniform length distribution in the range of *E. coli* proteins

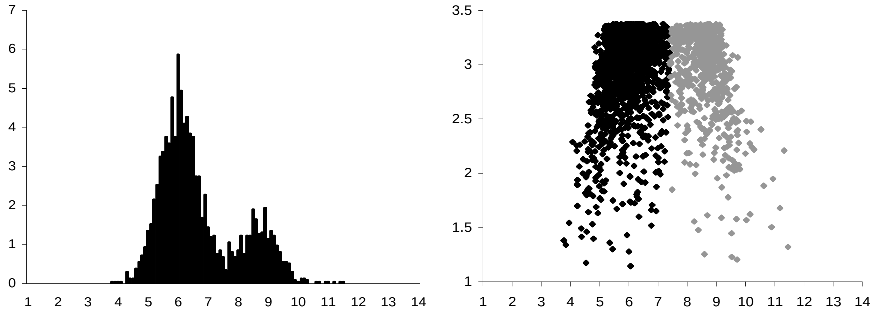

Artificial proteome: amino acid composition calculated for the base composition of the *E. coli* genome; length of *E. coli* proteins

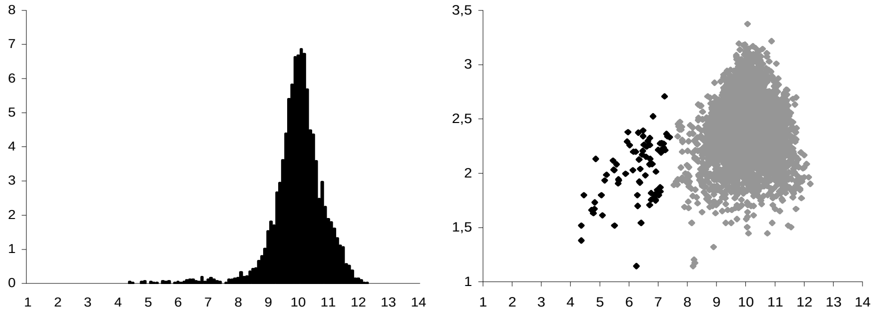

Supplement: Additional file 6 — The real and artificial proteomes of Escherichia coli K12. Left panel: histograms of pI values at 0.1 unit intervals (X axis: class of pI; Y axis: percent); right panel: relationships between the logarithm of length of proteins (Y axis) and their pI (Y axis). [file 1471-2164-8-163-S6.pdf]
