# Supplementary material for: The relationships between the isoelectric point and: length of proteins, taxonomy and ecology of organisms
Source: BMC Genomics. 2007 Jun 12;8:163. doi: 10.1186/1471-2164-8-163 (PMC1905920; doi:10.1186/1471-2164-8-163)

Nuclear-coded proteins targeted to plastid

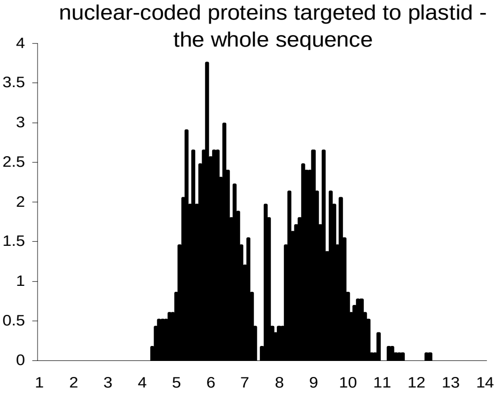

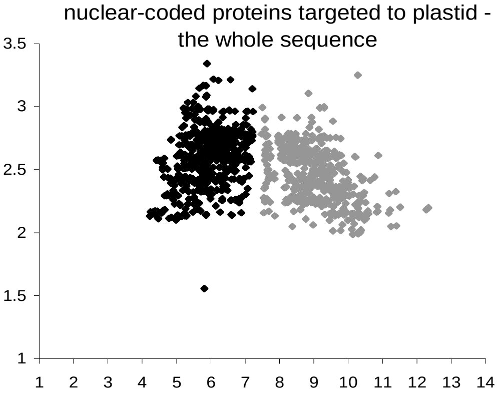

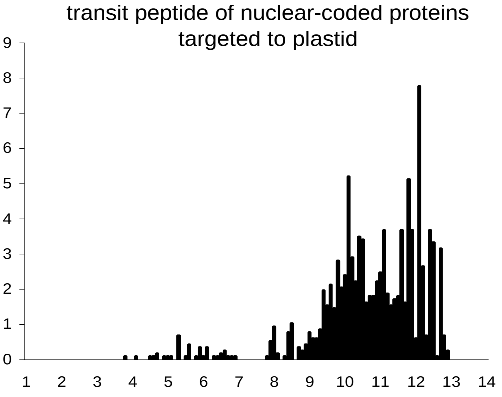

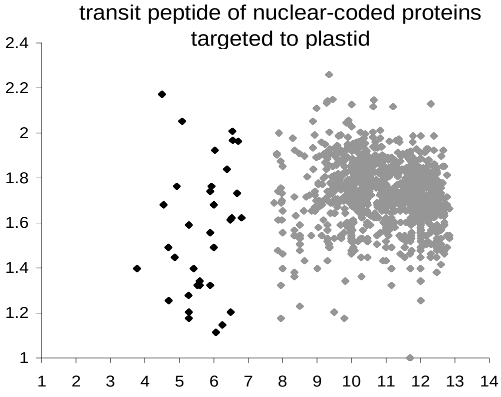

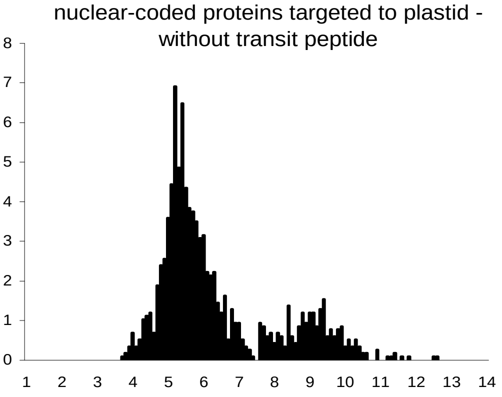

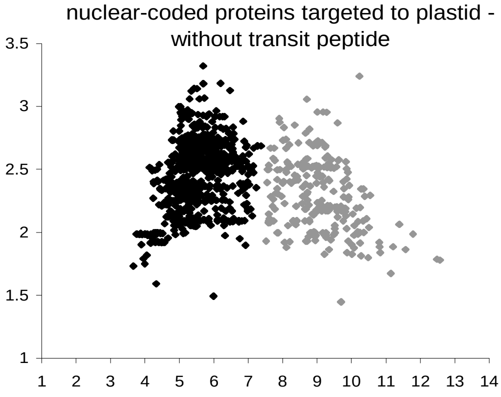

Nuclear-coded proteins targeted to mitochondrion

all groups

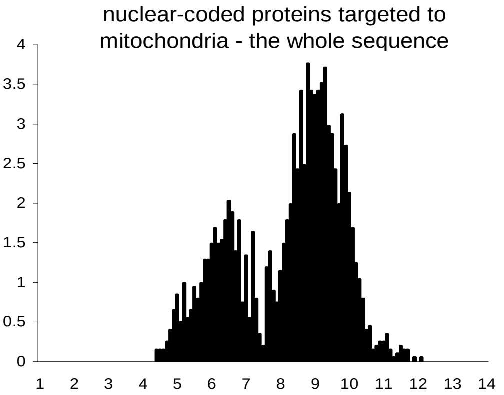

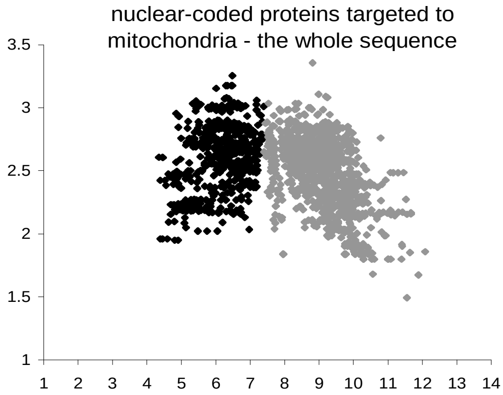

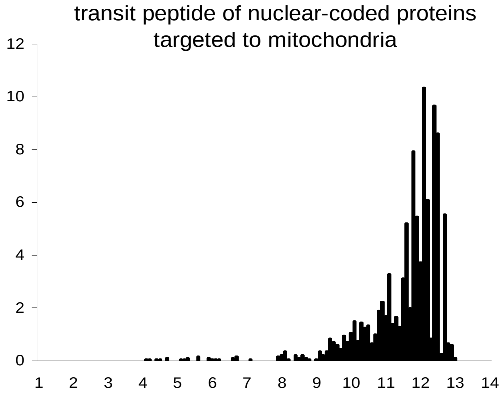

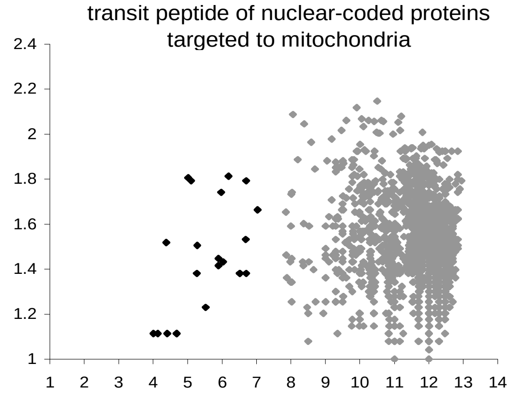

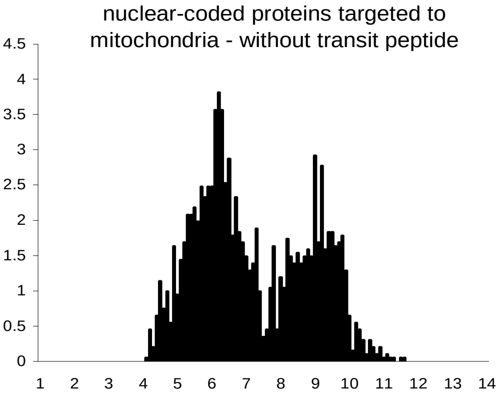

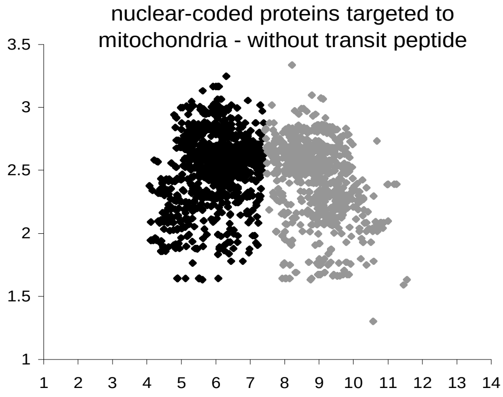

Nuclear-coded proteins targeted to mitochondrion

Protista

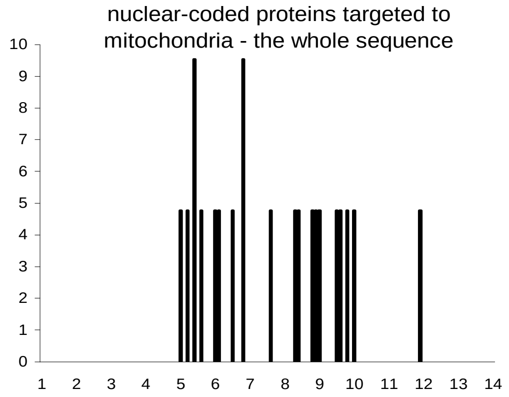

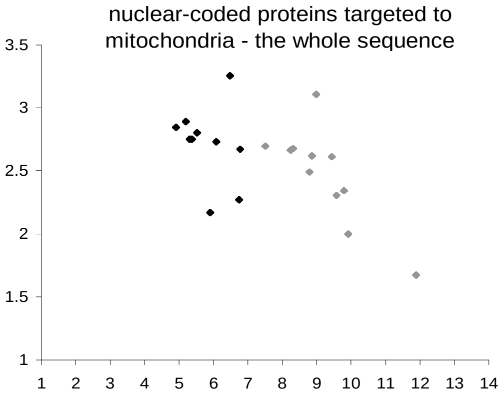

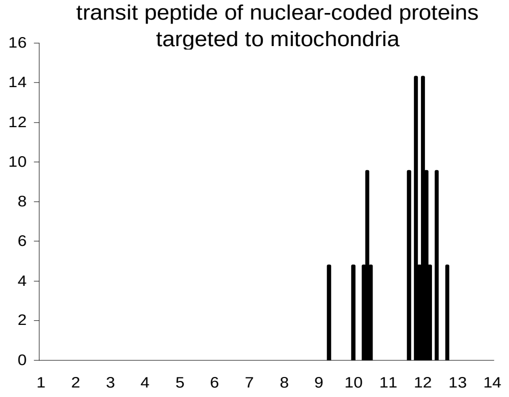

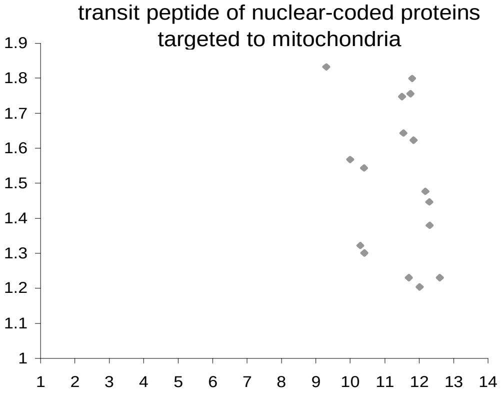

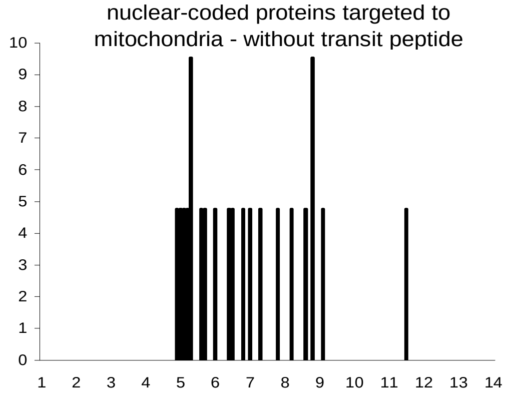

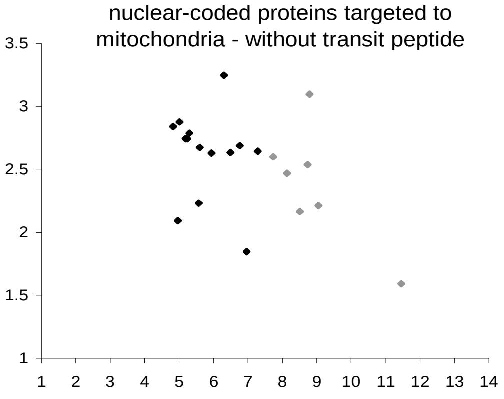

Nuclear-coded proteins targeted to mitochondrion

Viridiplantae

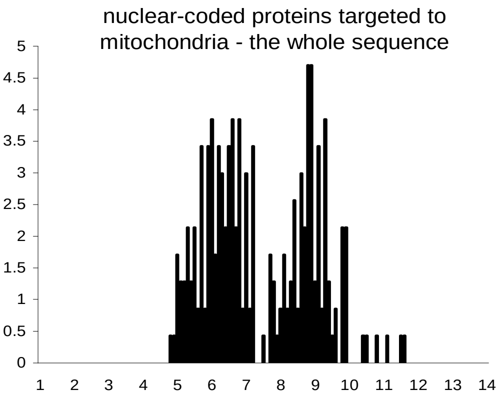

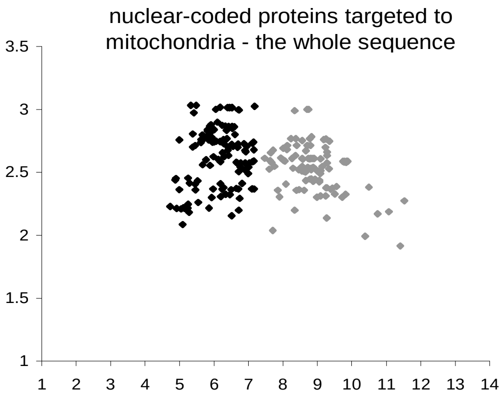

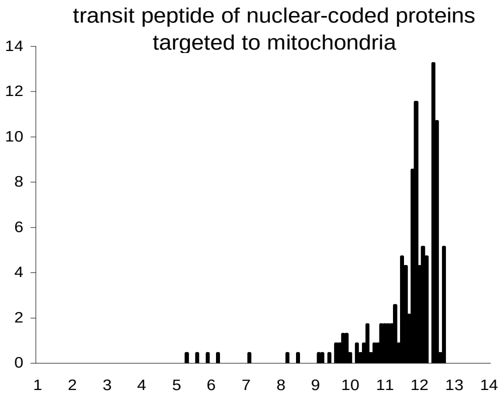

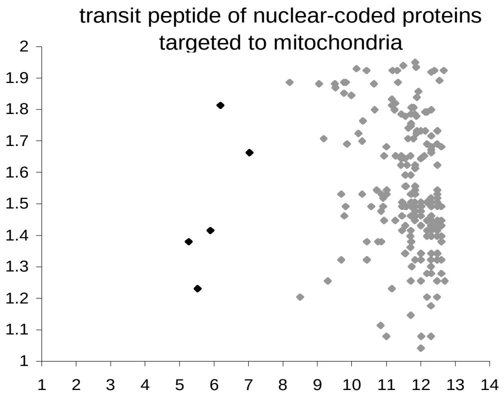

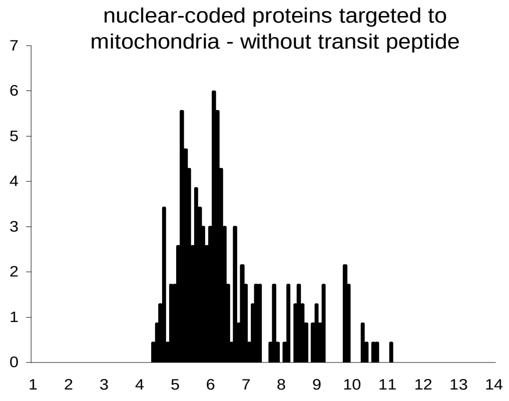

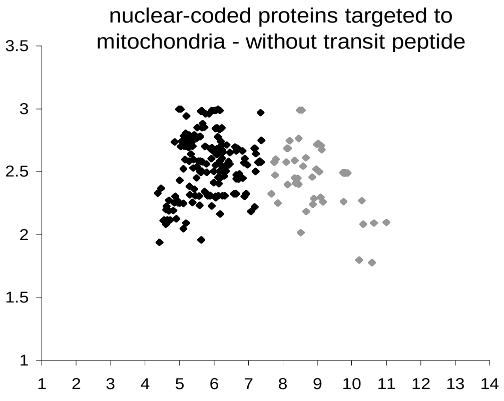

Nuclear-coded proteins targeted to mitochondrion

Fungi

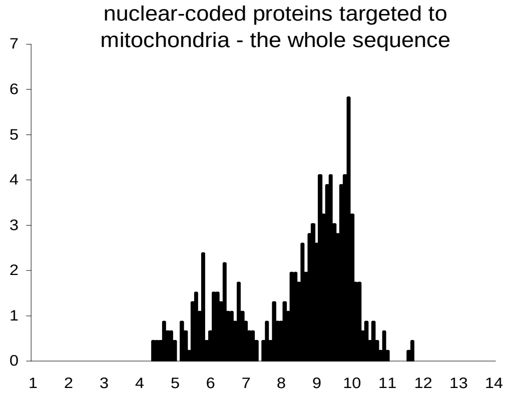

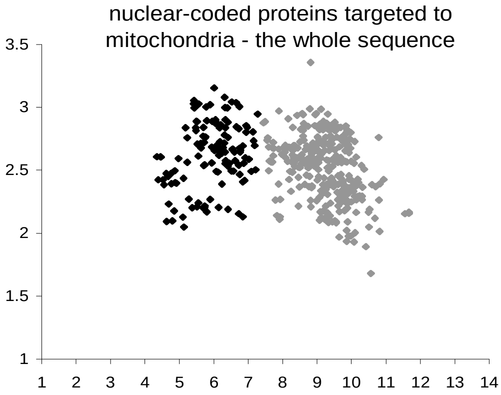

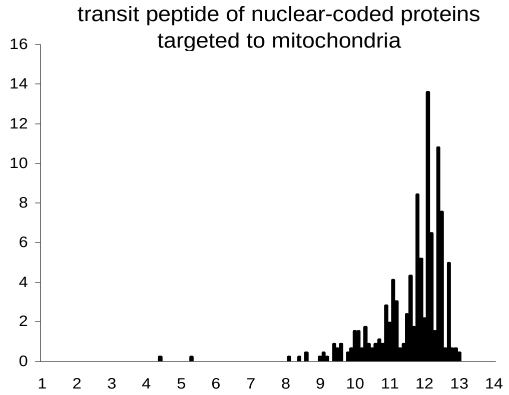

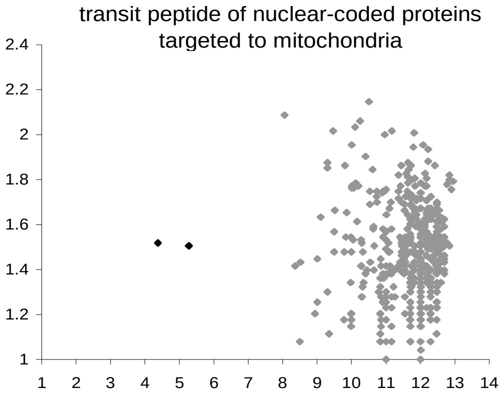

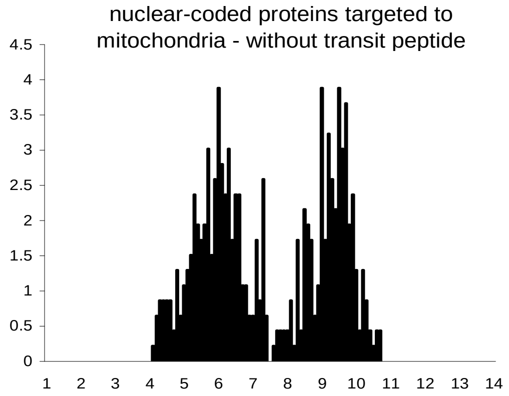

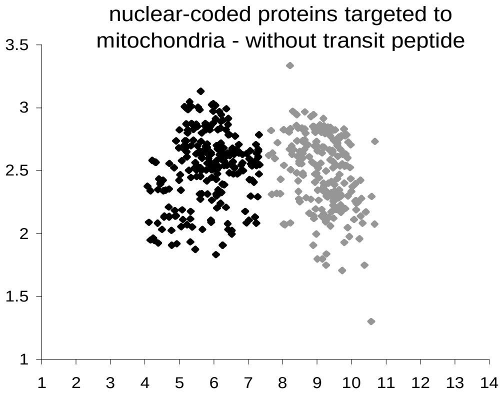

Nuclear-coded proteins targeted to mitochondrion

non-Chordata

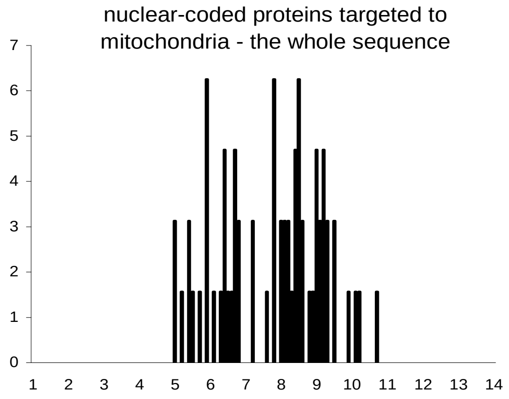

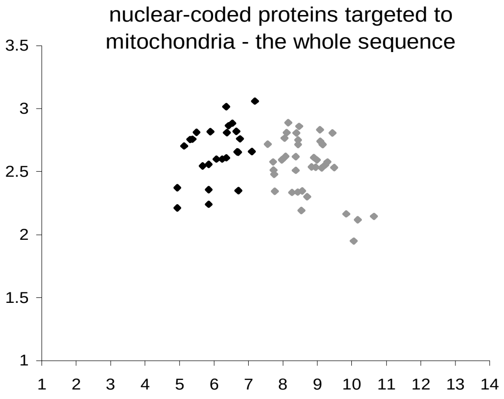

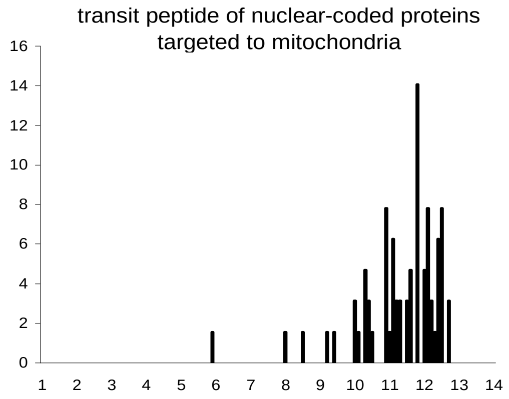

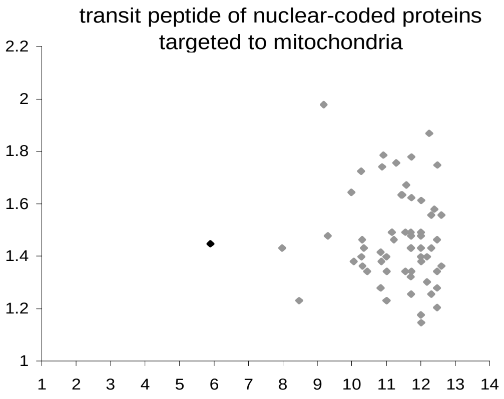

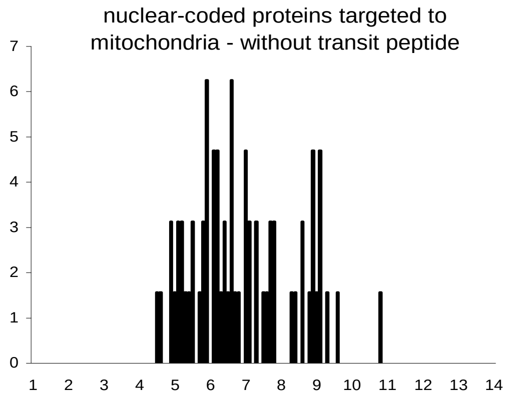

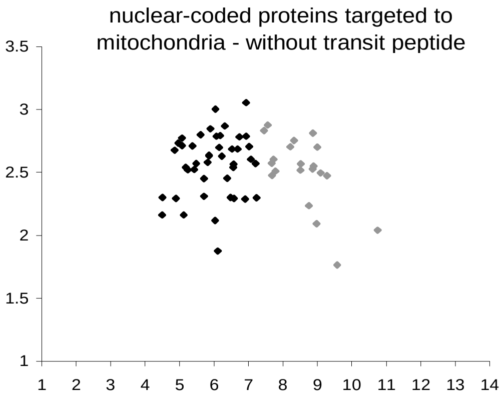

Nuclear-coded proteins targeted to mitochondrion

Chordata

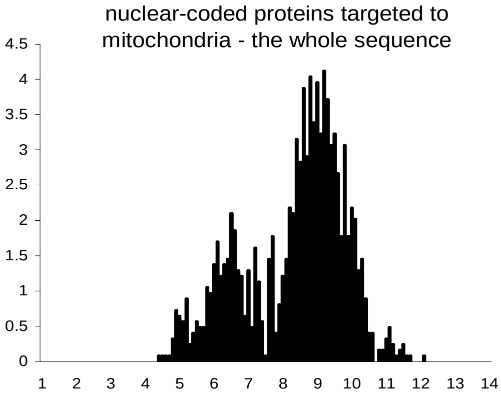

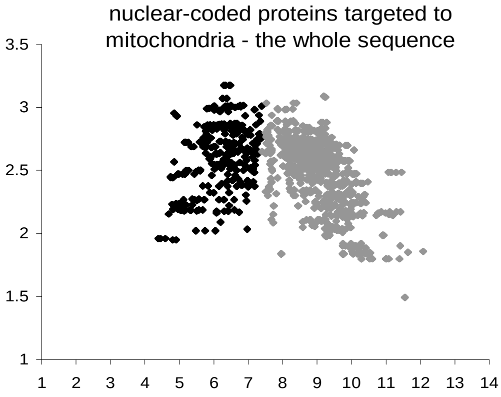

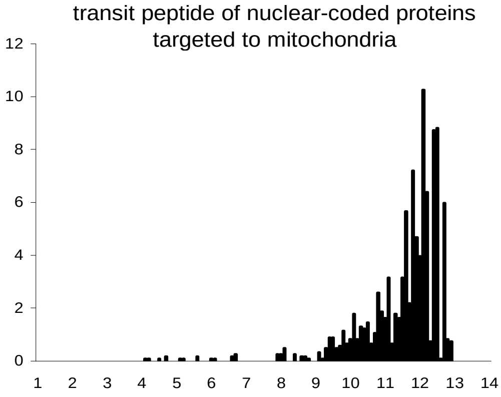

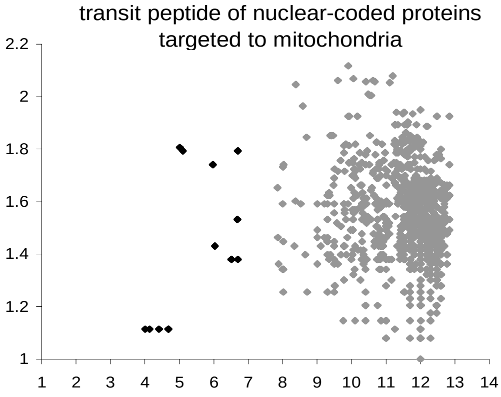

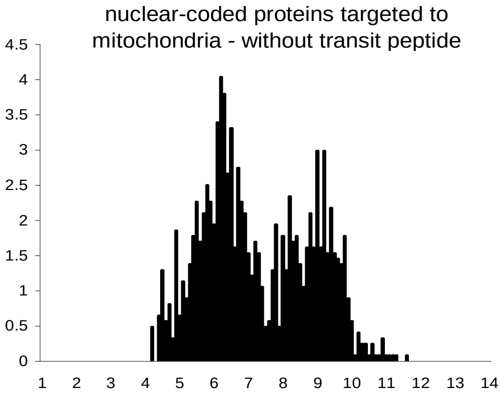

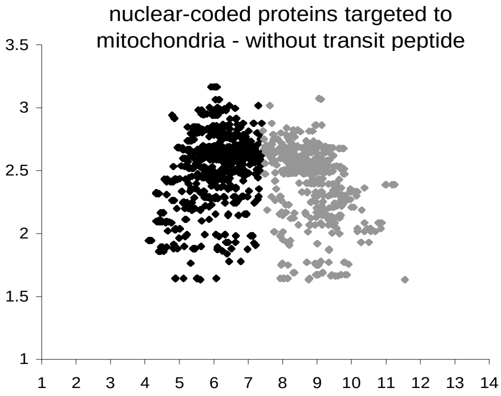

Supplement: Additional file 8 — PI distribution for nuclear-encoded proteins targeted to organelles: plastid and mitochondrion. Left panel: histograms of pI values at 0.1 unit intervals (X axis: class of pI; Y axis: percent); right panel: relationships between the logarithm of length of proteins (Y axis) and their pI (Y axis). Black points represent the set of acidic proteins while grey ones – the set of basic proteins. Transit peptides, premature proteins (i.e. the whole sequence) and mature proteins (i.e. without transit peptide) were analysed separately. Mitochondrial proteomes were divided to various taxonomical groups. [file 1471-2164-8-163-S8.pdf]
