# Supplementary material for: The relationships between the isoelectric point and: length of proteins, taxonomy and ecology of organisms
Source: BMC Genomics. 2007 Jun 12;8:163. doi: 10.1186/1471-2164-8-163 (PMC1905920; doi:10.1186/1471-2164-8-163)
Supplement: Additional file 9 — PI distribution for proteomes of different subcellular localization. Left panel: histograms of pI values at 0.1 unit intervals (X axis: class of pI; Y axis: percent); right panel: relationships between the logarithm of length of proteins (Y axis) and their pI (Y axis). Black points represent the set of acidic proteins while grey ones – the set of basic proteins. [file 1471-2164-8-163-S9.pdf]

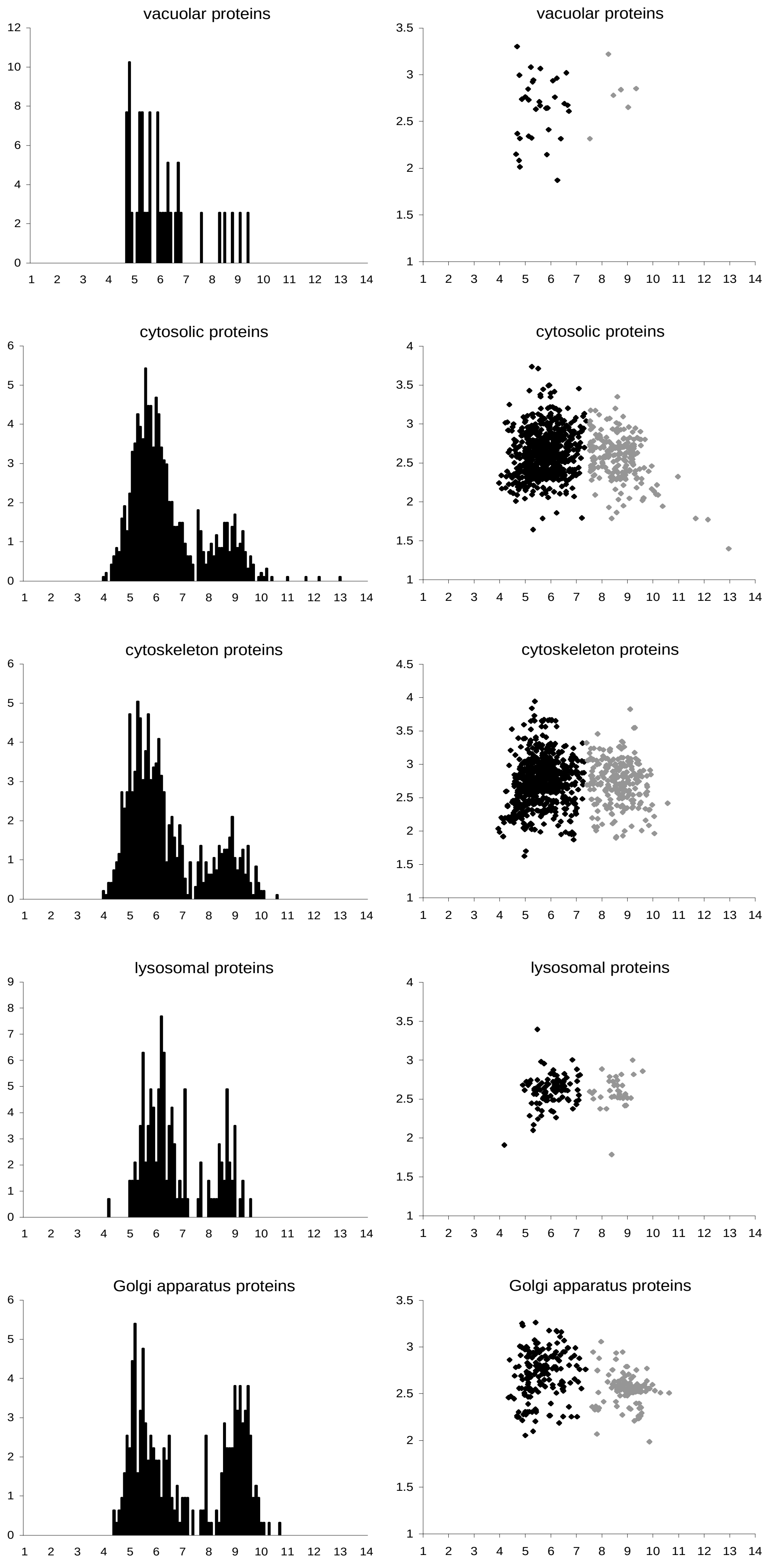
vacuolar proteins
0 2 4 6 8 10 12
1 2 3 4 5 6 7 8 9 10 11 12 13 14
vacuolar proteins
1 1.5 2 2.5 3 3.5
1 2 3 4 5 6 7 8 9 10 11 12 13 14
cytosolic proteins
0 1 2 3 4 5 6
1 2 3 4 5 6 7 8 9 10 11 12 13 14
cytosolic proteins
1 1.5 2 2.5 3 3.5 4
1 2 3 4 5 6 7 8 9 10 11 12 13 14
cytoskeleton proteins
0 1 2 3 4 5 6
1 2 3 4 5 6 7 8 9 10 11 12 13 14
cytoskeleton proteins
1 1.5 2 2.5 3 3.5 4 4.5
1 2 3 4 5 6 7 8 9 10 11 12 13 14
lysosomal proteins
0 1 2 3 4 5 6 7 8 9
1 2 3 4 5 6 7 8 9 10 11 12 13 14
lysosomal proteins
1 1.5 2 2.5 3 3.5 4
1 2 3 4 5 6 7 8 9 10 11 12 13 14
Golgi apparatus proteins
0 1 2 3 4 5 6
1 2 3 4 5 6 7 8 9 10 11 12 13 14
Golgi apparatus proteins
1 1.5 2 2.5 3 3.5
1 2 3 4 5 6 7 8 9 10 11 12 13 14

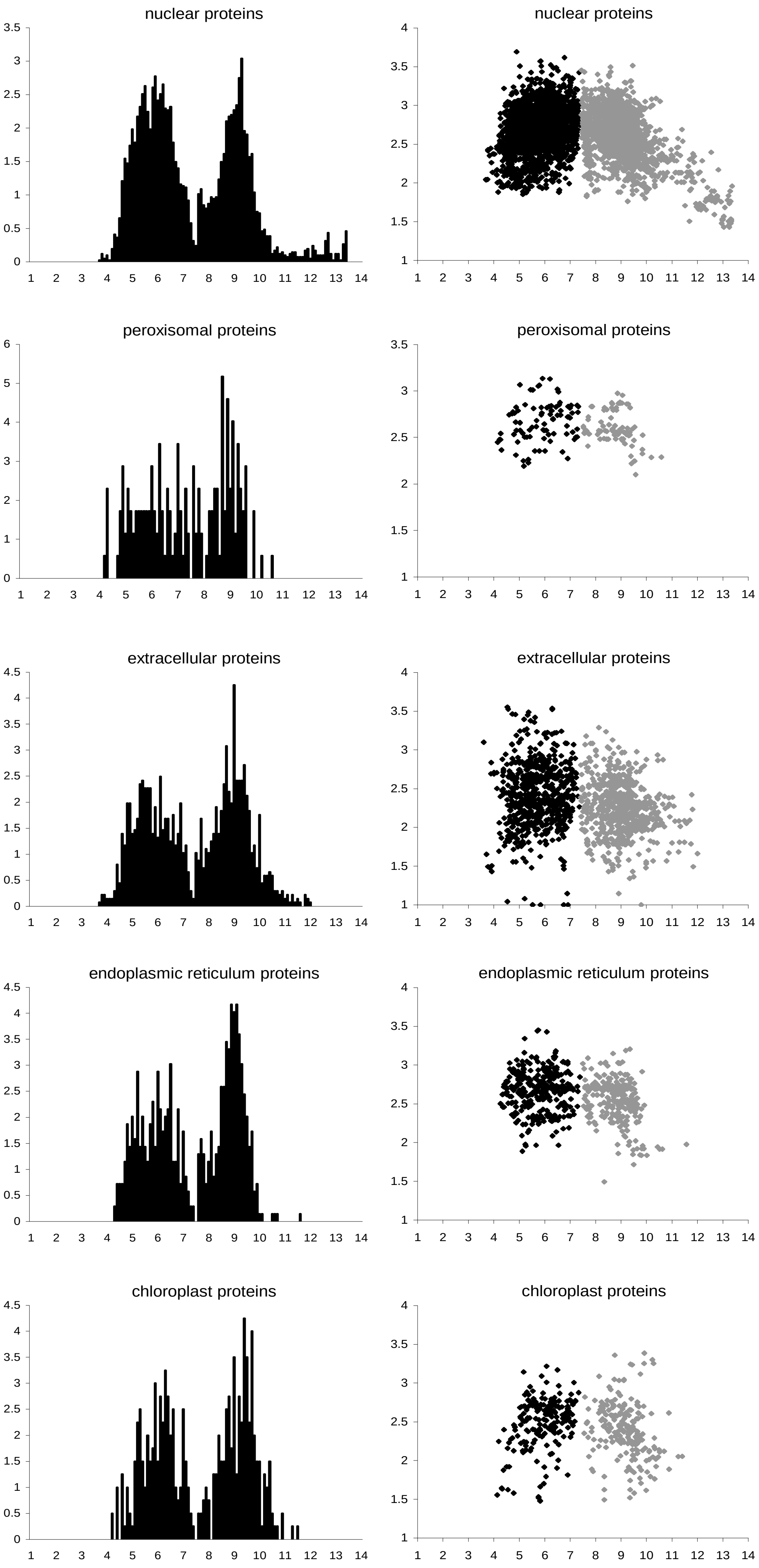
nuclear proteins
nuclear proteins
peroxisomal proteins
peroxisomal proteins
extracellular proteins
extracellular proteins
endoplasmic reticulum proteins
endoplasmic reticulum proteins
chloroplast proteins
chloroplast proteins

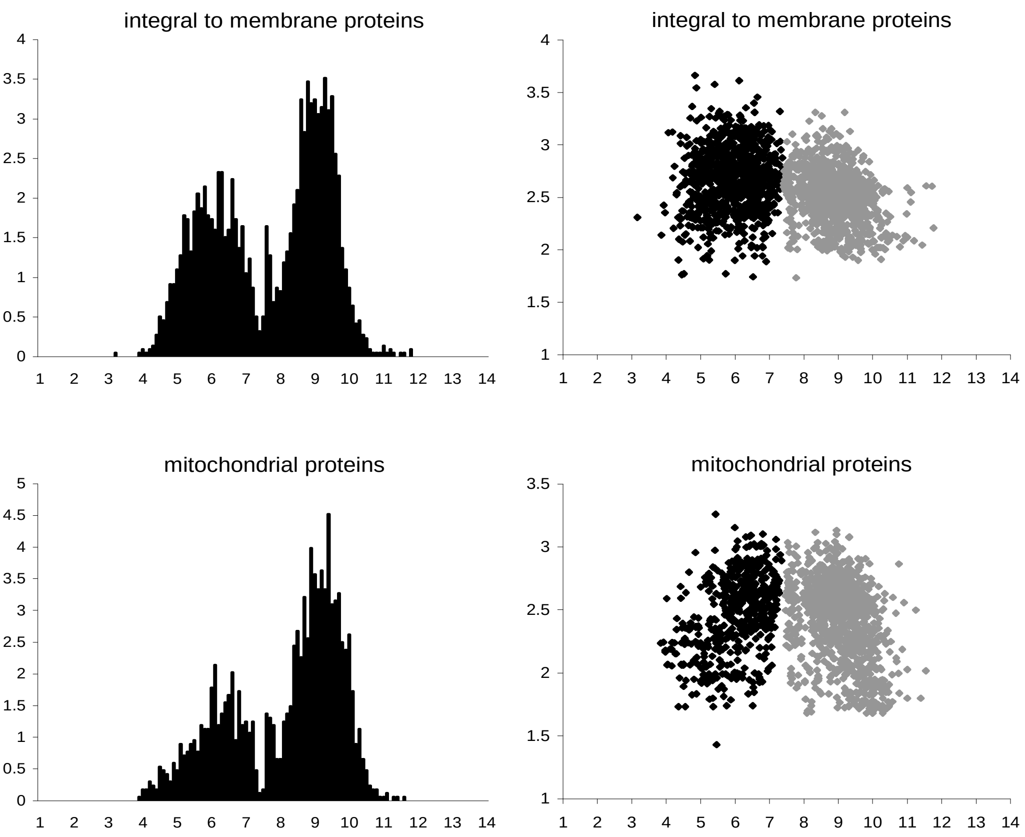
integral to membrane proteins
4
3.5
3
2.5
2
1.5
1
0.5
0
1 2 3 4 5 6 7 8 9 10 11 12 13 14
integral to membrane proteins
4
3.5
3
2.5
2
1.5
1
1 2 3 4 5 6 7 8 9 10 11 12 13 14
mitochondrial proteins
5
4.5
4
3.5
3
2.5
2
1.5
1
0.5
0
1 2 3 4 5 6 7 8 9 10 11 12 13 14
mitochondrial proteins
3.5
3
2.5
2
1.5
1
1 2 3 4 5 6 7 8 9 10 11 12 13 14
