# Supplementary material for: The relationships between the isoelectric point and: length of proteins, taxonomy and ecology of organisms
Source: BMC Genomics. 2007 Jun 12;8:163. doi: 10.1186/1471-2164-8-163 (PMC1905920; doi:10.1186/1471-2164-8-163)
Supplement: Additional file 10 — Ecological changes and their relationships with the pI bias of proteomes in closely related organisms. [file 1471-2164-8-163-S10.doc]

Tab. 1. Ecological changes and their relationships with the pI bias of proteomes in closely related organisms.

| Organism | pI bias | Ecological property |
| --- | --- | --- |
| Bacillus |  |  |
| *B. cereus* ATCC 10987 | -19.8 | non-halophilic |
| *B. cereus* ATCC 14579 | -24.4 | non-halophilic |
| *B. cereus* ZK | -26.5 | non-halophilic |
| *B. anthracis* 'Ames Ancestor' | -23.3 | non-halophilic |
| *B.* *anthracis* Sterne | -24.6 | non-halophilic |
| *B. anthracis* A2012 | -25.0 | non-halophilic |
| *B. thuringiensis* konkukian 97-27 | -23.6 | non-halophilic |
| *B. licheniformis* DSM 13 | -25.7 | non-halophilic |
| *B. licheniformis* ATCC 14580 | -26.3 | non-halophilic |
| *B. subtilis* *subtilis* 168 | -28.9 | non-halophilic |
| *B. clausii* KSM-K16 | -38.7 | mesohalophilic |
| *B. halodurans* C-125 | -39.1 | mesohalophilic |
| Burkholderia |  |  |
| *B. pseudomallei* K96243 | -6.3 | terrestrial habitat |
| *B.* *mallei* ATCC 23344 | 9.0 | host-associated |
| Corynebacterium |  |  |
| *C. glutamicum* ATCC 13032 | -57.8 | multiple habitat |
| *C. efficiens* YS-314 | -50.0 | multiple habitat |
| *C. diphtheriae* NCTC 13129 | -42.5 | host-associated |
| Mycobacterium |  |  |
| *M. avium* *paratuberculosis* k10 | -35.1 | multiple habitat |
| *M.* *leprae* TN | -34.7 | host-associated |
| *M.* *bovis* AF2122/97 | -29.8 | host-associated |
| *M.* *tuberculosis* H37Rv | -29.8 | host-associated |
| *M.* *tuberculosis* CDC1551 | -23.3 | host-associated |
| ***Streptococcus*** |  |  |
| *S. pneumoniae* TIGR4 | -33.8 | multiple habitat |
| *S. pneumoniae* R6 | -31.3 | multiple habitat |
| *S. thermophilus* LMG 18311 | -29.1 | multiple habitat |
| *S. thermophilus* CNRZ1066 | -27.8 | multiple habitat |
| *S. pyogenes* M1 GAS | -25.9 | host-associated |
| *S. pyogenes* MGAS315 | -22.3 | host-associated |
| *S. pyogenes* SSI-1 | -21.4 | host-associated |
| *S. pyogenes* MGAS8232 | -20.8 | host-associated |
| *S. pyogenes* MGAS10394 | -18.2 | host-associated |
| *S. agalactiae* NEM316 | -19.4 | host-associated |
| *S. agalactiae* 2603V/R | -16.0 | host-associated |
| *S. mutans* UA159 | -12.3 | host-associated |
| Vibrio |  |  |
| *V. cholerae* O1 biovar eltor N16961 | -35.6 | non-halophilic |
| *V.* *parahaemolyticus* RIMD 2210633 | -42.9 | mesohalophilic |
| *V.* *fischeri* ES114 | -48.2 | mesohalophilic |
| *V.* *vulnificus* YJ016 | -38.2 | halophilic |
| *V.* *vulnificus* CMCP6 | -49.2 | halophilic |
