# Supplementary material for: The relationships between the isoelectric point and: length of proteins, taxonomy and ecology of organisms
Source: BMC Genomics. 2007 Jun 12;8:163. doi: 10.1186/1471-2164-8-163 (PMC1905920; doi:10.1186/1471-2164-8-163)
Supplement: Additional file 12 — Characteristics of 39 sets of conserved orthologous proteins present in all selected 100 prokaryotic organisms. The correlation coefficient was calculated between the pI of proteins and the pI bias of the whole proteomes. [file 1471-2164-8-163-S12.doc]

Tab. 2. Characteristics of 39 sets of conserved orthologous proteins present in all selected 100 prokaryotic organisms. The correlation coefficient was calculated between the pI of proteins and the pI bias of the whole proteomes.

| name of protein set | short description | average  pI | correlation  coefficient | p value |
| --- | --- | --- | --- | --- |
| fusA | translation elongation factor | 5.26 | 0.64 | < 0.001 |
| nusA | transcription termination factor | 5.41 | 0.21 | 0.03 |
| ychF | GTP-binding protein | 5.45 | 0.76 | < 0.001 |
| efp | translation elongation factor | 5.55 | 0.59 | < 0.001 |
| leuS | leucyl-tRNA synthetase | 5.98 | 0.88 | < 0.001 |
| proS | prolyl-tRNA synthetase | 6.02 | 0.87 | < 0.001 |
| valS | valyl-tRNA synthetase | 6.06 | 0.88 | < 0.001 |
| thrS | threonyl-tRNA synthetase | 6.12 | 0.81 | < 0.001 |
| metG | methionyl-tRNA synthetase | 6.13 | 0.89 | < 0.001 |
| serS | seryl-tRNA synthetase | 6.19 | 0.85 | < 0.001 |
| argS | arginyl-tRNA synthetase | 6.24 | 0.88 | < 0.001 |
| pheS | phenylalanyl-tRNA synthetase | 6.29 | 0.80 | < 0.001 |
| hisS | histidyl-tRNA synthetase | 6.31 | 0.85 | < 0.001 |
| tyrS | tyrosyl-tRNA synthetase | 6.50 | 0.90 | < 0.001 |
| dnaX | gamma/tau DNA polymerase III | 6.70 | 0.61 | < 0.001 |
| rpoC | RNA polymerase beta' subunit | 6.95 | 0.74 | < 0.001 |
| trpS | tryptophanyl-tRNA synthetase | 7.04 | 0.82 | < 0.001 |
| infB | translation initiation factor 2 | 7.10 | 0.46 | < 0.001 |
| rpsB | 30S ribosomal protein S2 | 7.34 | 0.56 | < 0.001 |
| rplK | 50S ribosomal protein L11 | 9.17 | 0.27 | 0.006 |
| rplA | 50S ribosomal protein L1 | 9.35 | 0.49 | < 0.001 |
| rplE | 50S ribosomal protein L5 | 9.43 | 0.51 | < 0.001 |
| rplF | 50S ribosomal protein L6 | 9.58 | 0.41 | < 0.001 |
| secY | preprotein translocase subunit | 9.59 | 0.37 | < 0.001 |
| rpsH | 30S ribosomal protein S8 | 9.60 | 0.41 | < 0.001 |
| rplW | 50s ribosomal protein L23 | 9.76 | 0.37 | < 0.001 |
| rpsQ | 30S ribosomal protein S17 | 9.87 | 0.50 | < 0.001 |
| rpsC | 30S ribosomal protein S3 | 9.88 | 0.33 | < 0.001 |
| rplC | 50S ribosomal protein L3 | 9.93 | 0.43 | < 0.001 |
| rpsD | 30S ribosomal protein S4 | 9.95 | 0.32 | < 0.001 |
| rpsE | 30S ribosomal protein S5 | 10.03 | 0.32 | 0.001 |
| rpsG | 30S ribosomal protein S7 | 10.06 | 0.30 | 0.002 |
| rpsS | 30S ribosomal protein S19 | 10.30 | 0.32 | 0.001 |
| rplV | 50S ribosomal protein L22 | 10.37 | 0.30 | 0.002 |
| rpsM | 30S ribosomal protein S13 | 10.55 | 0.31 | 0.001 |
| rpsI | 30S ribosomal protein S9 | 10.57 | 0.50 | < 0.001 |
| rplB | 50S ribosomal protein L2 | 10.74 | -0.12 | 0.249 |
| rpsK | 30S ribosomal protein S11 | 10.85 | 0.04 | 0.726 |
| rpsL | 30S ribosomal protein S12 | 11.04 | 0.14 | 0.149 |
