# Supplementary material for: The relationships between the isoelectric point and: length of proteins, taxonomy and ecology of organisms
Source: BMC Genomics. 2007 Jun 12;8:163. doi: 10.1186/1471-2164-8-163 (PMC1905920; doi:10.1186/1471-2164-8-163)
Supplement: Additional file 13 — Discussion about relationships between pI and other phenomena [file 1471-2164-8-163-S13.doc]

**Relationships between pI and other phenomena**

Even much conserved proteins are subject to pI changes, but it does not mean that some special adaptive proteins involved in interactions with the environment are not under selection for their pI. Such selection acts probably on proteomes of halophilic microorganisms. As it was already mentioned their proteins are rich in acidic residues, better hydrated, which influences the organization of salt ion network and can build salt bridges. It makes these proteins more stable and soluble in a high salt concentration environment so they can maintain their function [1-7]. Genomes of halophiles are GC-rich [e.g. 8] which may influence the observed negative correlation between pI bias and GC content (Fig. 8B). However, the content is rather an adaptation to minimization of DNA damage caused by solar UV radiation [8,9] to which halophiles are usually exposed but it is not the result of selection for encoding acidic residues. The high GC content should rather generate basic proteomes (see Fig. 10).

It was argued that basic proteomes may be the result of adaptation to acidic environment because they could buffer the proton excess especially in the case of *Coxiella* *burnetti* (pI bias = 20%) living in acidic phagosomes and *Helicobacter* *pylori* residing in low-pH gastric environment [10]. Actually, the pI bias of proteomes of two *H.* *pylori* strains equals 18% and 22% whereas the proteome of non-acidophilic *H.* *hepaticus* is slightly acidic (–6%). However, there are exceptions to this rule. For example, bacteria with acidic proteomes such as Anaplasma, Brucella, Chlamydiae and Ehrlichia reside and replicate in vacuoles or phagosomes that usually have low pH. Probably, different species have used various strategies to adapt to acidic environment, not necessary connected with changes in pI of their proteins.

It has been also suggested that the pI distribution biased towards basicity is also connected with thermophily in Archaea that has been explained by minimization of protein aggregation due to the increase of the net charge [11]. However, we have not observed a significant correlation between pI and temperature requirements. Probably such a feature is characteristic for specific bacteria living in high temperatures (> 60^o^C) and is obscured by other relations when groups of microorganisms living in much wider range of temperatures are considered. Assuming that the high-temperature environment exerts strong mutagenic effect, the basicity of proteomes of the extreme thermophiles may result from the increased mutational pressure as in the intracellular bacteria.

## References

1. Bonnete F, Madern D, Zaccai G: **Stability against denaturation mechanisms in halophilic malate dehydrogenase "adapt" to solvent conditions.** *J Mol Biol* 1994, **244:**436-447.
2. Rao JK, Argos P: **Structural stability of halophilic proteins.** *Biochemistry* 1981, **20:**6536–6543.
3. Dym O, Mevarech JL, Sussman L: **Structural features that stabilize halophilic malate dehydrogenase from an archaebacterium.** *Science* 1995, **267:**1334-1346.
4. Eisenberg H: **Life in unusual environments: progress in understanding the structure and function of enzymes from extreme halophilic bacteria*.*** *Arch Biochem Biophys* 1995, **318:**1–5.
5. Madern D, Zaccai G: **Stabilisation of halophilic malate dehydrogenase from Haloarcula marismortui by divalent cations – effects of temperature, water isotope, cofactor and pH.** *Eur J Biochem* 1997, **249:**607–611.
6. Elcock AH, McCammon JA: **Electrostatic contributions to the stability of halophilic proteins.** *J Mol Biol* 1998, **280:**731-748.
7. Oren A, Mana L: **Amino acid composition of bulk protein and salt relationships of selected enzymes of *Salinibacter ruber*, an extremely halophilic Bacterium.** *Extremophiles* 2002, **6:**217-223.
8. Goo YA, Roach J, Glusman G, Baliga NS, Deutsch K, Pan M, Kennedy S, DasSarma S, Ng WV, Hood L: **Low-pass sequencing for microbial comparative genomics.** *BMC Genomics* 2004, **5:**3.
9. Kennedy SP, Ng WV, Salzberg SL, Hood L, DasSarma S: **Understanding the adaptation of Halobacterium species NRC-1 to its extreme environment through computational analysis of its genome sequence.** *Genome Res* 2001, **11:**1641-1650.
10. Seshadri R, Paulsen IT, **Eisen JA** , Read TD, Nelson KE, Nelson WC, Ward NL, Tettelin H, Davidsen TM, Beanan MJ, Deboy RT, Daugherty SC, Brinkac LM, Madupu R, Dodson RJ, Khouri HM, Lee KH, Carty HA, Scanlan D, Heinzen RA, Thompson HA, Samuel JE, Fraser CM, Heidelberg JF: **Complete genome sequence of the Q-fever pathogen Coxiella burnetii.** *Proc Nat Acad Sci USA* 2003, **100:**5455-5460.
11. Kawashima T, Amano N, Koike H, Makino S, Higuchi S, Kawashima-Ohya Y, Watanabe K, Yamazaki M, Kanehori K, Kawamoto T, Nunoshiba T, Yamamoto Y, Aramaki H, Makino K, Suzuki M: **Archaeal adaptation to higher temperatures revealed by genomic sequence of *Thermoplasma volcanium*.** *Proc Natl Acad Sci USA* 2000, **97:**14257-14262.
